# Characterization of the microbiome and polyphenolic compounds in the medicinal plant *Dracocephalum tanguticum*

**DOI:** 10.7717/peerj.21626

**Published:** 2026-07-29

**Authors:** Erhao Zhang, Xiu Yin, Yazhou Lu, Hong Quan, Liang Li, Zhongbin Wang, Xiaozhong Lan

**Affiliations:** 1 The Provincial and Ministerial Co-founded Collaborative Innovation Center for R&D in Xizang Characteristic Agricultural and Animal Husbandry Resources, Xizang Agriculture and Animal Husbandry University, Linzhi Xizang China; 2 Key Laboratory of Traditional Zang’s Medicine Resources Conservation and Utilization of Xizang Autonomous Region, Xizang Agriculture and Animal Husbandry University, Linzhi, China; 3 Resources and Environment College, Xizang Agriculture and Animal Husbandry University, Linzhi, China

**Keywords:** *Dracocephalum tanguticum*, Various organs, Endophytes, Polyphenol, Correlation

## Abstract

*Dracocephalum tanguticum* (Maxim) is rich in various chemical constituents and is widely used in traditional Zang medicine. Endophytes play a direct or indirect role in the biosynthesis of active compounds and plant growth. However, little is known about the characteristics of endophytes and polyphenolic compounds in the various organs of *D. tanguticum*. In this study, high-throughput sequencing and polyphenol-targeted metabolomics were employed to analyze endophytic community diversity and assembly processes, polyphenolic compound content, and their correlations. The results showed that the endophytic compositions of the leaf and stem organs were similar, and significantly different from that in the root organs; however, the endophytic diversity did not differ significantly across the various organs. Actinobacteriota and Pseudomonadota were the dominant bacterial phyla, Ascomycota and Basidiomycota were the dominant fungal phyla in the various organs, while the dominant endophytic genera were significantly different. The endophytic community assembly was influenced mainly by stochastic processes in the various organs. A total of 75 polyphenolic compounds were identified, and the contents of the polyphenolic compounds in the various organs of *D. tanguticum* differed significantly. The correlation analysis revealed varying degrees of positive and negative correlation between endophytes and polyphenolic compounds. These findings clarify the characteristics of the endophytes and polyphenolic compounds, and lay a theoretical foundation for the identification and application functional microbiomes in the *D. tanguticum*.

## Introduction

*Dracocephalum tanguticum* (Maxim) is a perennial herb that belongs to the Lamiaceae family, and is mainly distributed on Qinghai Xizang Plateau. *D. tanguticum*, as a traditional Xizang medicine, has been recorded in the Xizang medicine standard (Li et al., 2016). *D. tanguticum* is rich in various chemical constituents, such as flavonoids, terpenoids, polysaccharides and volatile components (Zeng et al., 2010). The major active ingredients are flavonoids and terpenes (Chen et al., 2022b), which are commonly used to treat hepatitis, dizziness, gastritis, ulcers, and arthritis (Li et al., 2016; Liu et al., 2009; Wang et al., 2010). Previous studies have employed various methods to isolate and identify flavonoids from *D. tanguticum*. For instance, Li et al. (2016) utilized high-performance liquid chromatographic fingerprints combined with tandem mass spectrometry and chemometrics to identify 33 flavonoids. Additionally, Chen et al. (2022a); Chen et al. (2022b) employed three different high-speed counter-current chromatography modes to separate five flavone glycosides. However, due to the inherent complexity of the herbal medicines, which contain a plethora of primary and secondary metabolites, these methods exhibit certain limitations (Qian et al., 2023). Metabolomics technology presents a promising approach for investigating functional secondary metabolites and metabolic pathways. It has been successfully applied to study the medicinal constituents of *Bistorta vivipara* (He et al., 2024), bisbenzylisoquinoline alkaloids (BIAs) in *Phellodendron amurense* Rupr Zhang et al. (2024b), and volatile flavor compounds in cigar tobacco (Wang et al., 2024). Consequently, metabolomics technology is anticipated to significantly advance research on a wide range of medicinal plants.

Endophytes are microorganisms residing within plant organs such as leaves, stems, and roots without causing harm to the host (Huang et al., 2023; Zhang et al., 2023). They are associated with host growth, development and metabolic synthesis (Golinska et al., 2015; Chen et al., 2019). Endophytes, such as *Bacillus xiamenensis* NIBSM_SgR3 and *Bacillus velezensis* NIBSM_SgL59, isolated from *Rauvolfia serpentina* L. possess the potential to promote plant growth and exhibit antibacterial properties (Anisha et al., 2025). Endophytes, *Bacillus subtilis* IGPEB 34 and *Bacillus pumilis* IGPEB 37, significantly increased the growth of ginger (Jabborova et al., 2025). Endophytes play a direct or indirect role in the biosynthesis of active compounds in medicinal plants (Ling et al., 2022). Previous studies have shown that the exopolysaccharides produced by the endophytic *Penicillium javanicum* have anti-inflammatory activity (Lai et al., 2023). Additionally, the extracts from endophytic bacteria, such as *Enterobacter*, *Bacillus*, *Erwinia*, *Stenotrophomonas*, and *Pantoea*, contain various pharmacologically active ingredients (Rahman et al., 2022). The endophytic fungi (*Chaetomium nozdrenkoae* and *Penicillium oxalicum*) isolated from *Paris polyphylla* var. *yunnanensis* had antibacterial and antitumour activity (Zhang et al., 2026). The colonization of *Polygonum* by endophytic fungi significantly affects the contents of active ingredients (Zhang et al., 2022). Furthermore, *Alternaria tenuissima* ZP28 and ZM148, endophytic fungi that produce flavonoids, demonstrate antioxidant and antibacterial properties (Zheng et al., 2022). Consequently, the quality of medicinal plants depends on the contents and quantity of medicinal components associated with endophytic microbes (Huang, Long & Lam, 2018). Research on the interactions between microbes and metabolites elucidates the roles of endophytic microbes in plants. Currently, there is significant interest in the functional microbes associated with medicinal components. For instance, utilizing microbiome and metabolome data, researchers have identified numerous medicinal components in the roots of *Polygonum viviparum* L (He et al., 2024) and have mapped complex interaction networks between root-associated microorganisms of *Phellodendron amurense* and their key chemical compounds (Zhang et al., 2024a; Zhang et al., 2024b). Nevertheless, the endophytic community and the primary active compounds in *D. tanguticum* remain largely unknown.

To address the relationship between endophytic microbiomes and polyphenolic compounds in *D. tanguticum*, we studied the microbial community and metabolites among different organs of *D. tanguticum*. Furthermore, the relationships between the polyphenolic compounds and endophytic microbial community was investigated. Our study aims to provide a practical strategy for identifying and applying functional microbiomes and enhancing the quality of *D. tanguticum* by employing a microbiological approach to manipulate the organs-associated microbiomes.

## Materials and Methods

### Sample collection and processing

Samples of fresh leaves, stems, and roots from healthy *D. tanguticum* were gathered in Xizang, China. The fresh leaf, stem and root samples from fifty healthy *D. tanguticum* (three years old) were cut off and collected as one sample at each point of sampling. A total of nine sampling points were selected, the root, stem, and leaf of each sampling point constitute an independent biological replicate, respectively. DtL, DtS, and DtR represented leaf, stem and root of wild *D. tanguticum*, respectively. All samples were carefully washed by running water and surface-sterilized by the following operations: 75% ethanol for 1 min, and then 2.5% sodium hypochlorite for 3 min, subsequently, rinsed 6 times with sterile distilled water and dried on sterilized paper (Zhang et al., 2023). The effectiveness of surface sterilization was assessed by plating the last rinse water on Luria–Bertani (LB) and Potato Dextrose Agar (PDA) medium to confirm no microbial growth (Lu et al., 2020). The different organs samples at each sampling point were split into two portions: one for examining the endophytic community and the other portion randomly selected six different organs samples for conducting phenolics-target metabolomics analysis.

### 16S and ITS sequencing analysis of *D. tanguticum* endophytes

Total DNA was extracted from three different organs of *D. tanguticum* using plant DNA extraction kit (Invitrogen™, Life Technologies, Carlsbad, United States) according to the manufacturer’s instructions. The quality and quantity of the extraction DNA were verified by 1% gel electrophoresis and NanoDrop 2000 ultraviolet spectrophotometry (Thermo Fisher Scientific Inc, USA). The V5–V7 region of the 16S rRNA gene was amplified with primers 799F (5′-AACMGGATTAGATACCCKG-3′) and 1193R (5′-ACGTCATCCCCACCTTCC-3′), and the ITS1 variable region was amplified with primers ITS1F (5′-CTTGGTCATTTAGAGGAAGTAA-3′) and ITS2 (5′-GCTGCGTTCTTCATCGATGC-3′). All PCR amplification was performed using TransStart Fastpfu DNA Polymerase. After being examined and purified, the PCR products underwent sequencing on the Illumina MiSeq platform at Majorbio, Bio-Pharm Technology Co. Ltd. in Shanghai, China.

### Sequencing analysis

Flash (1.2.11) was used to overlap and merge the raw data from the Illumina MiSeq platform, followed by quality-controlled processing with QIIME (Version 1.9.1) (Caporaso et al., 2010). At 97% similarity, the clean sequences were clustered into operational taxonomic units (OTUs: microbial sequences into the basic units of communities in molecular biology analysis) using Vsearch (Eevers et al., 2016). The annotation of the bacterial and fungal OTUs was performed with Greengenes database (Release 13.8, http://greengenes.secondgenome.com/) and the UNITE database (Release 8.0, http://unite.ut.ee/index.php) using the RDP classifier (v 2.13). Data normalization was performed using the sample with the fewest sequences. All subsequent analyses were performed using the normalized data. Alpha diversity was analyzed with Mothur software (version 1.30.2) and included the Shannon index, Simpson index, Chao index, Shannon evenness index and Good’s coverage; Beta diversity and principal coordinate analysis (PCoA) were performed with QIIME (Version 1.9.1); Heatmaps and Venn diagrams were drawn and analyzed using R software (version 3.3.1). The co-occurrence network analysis was conducted by calculating Spearman’s correlation coefficients with absolute correlation coefficients ≥ 0.7 and *P* < 0.05, and the resulting network was visualized with Gephi (0.9.7) (Chen et al., 2022a; Chen et al., 2022b).

### Polyphenol-targeted metabolomic analysis

Metabolites were extracted from subsamples as follows: 100 mg of freeze-dried sample was added to 600 µL of ice-cold methanol-water (2:1, v/v, containing an internal standard mixture: 0.02 mg/mL succinic acid-2,2,3,3-d4, and 2-hydroxybenzoic-3,4,5,6-d4 acid) and 400 µL of chloroform, ground at 60 Hz for 2 min, ultrasonicated for 20 min in an ice-water bath, and then centrifuged at 4 °C and 13,000 rpm for 10 min, after which the supernatant was decanted into sample vials. It was then evaporated under a gentle nitrogen stream and reconstituted in 350 µL of a methanol-water mixture (7:18, v/v, containing an internal standard: 0.02 mg/mL L-2-chlorophenylalanine). The solution was ultrasonicated for 5 min in an ice-water bath and subsequently passed through a 0.22 µm organic phase pinhole filter prior to the UPLC-ESI-MS/MS analysis (UPLC, AB ExionLC; MS, AB Sciex Qtrap 6500+). The composition of the mobile phase was 0.1% formic acid (A) and acetonitrile (B). The chromatographic separation was conducted using a waters UPLC HSS T3 column (100 × 2.1 mm, 1.8 µm) with a flow rate of 0.35 mL/min and a column temperature set to 40  °C. The gradient elution program was as follows: 0–2 min at 0% B; 2–30 min from 0% to 50% B; 30–32 min from 50% to 95% B; 32–34 min at 95% B; 34–34.1 min from 100% to 0% B; 34.1–35.5 min at 0% B. The standard information was shown in Table S1. Data acquisition and preliminary analysis utilized Analyst 1.6.3 software. All metabolites were quantified based on peak area and calibration curve (Fig. S2). The metabolite extraction, identification, and quantification were carried out by OE Biotech Co., Ltd., Shanghai, China.

### Correlation analysis of the metabolome and microbiome

The relationship between differential metabolites and endophytic genera across different *D. tanguticum* organs was analyzed, and the Spearman correlation analysis was calculated. The different metabolites were screened based on a *P*-value <0.05 and log 2 (fold change) ≥1, and the different endophytic genera was screened using the LEfSe method based on a *P*-value <0.05. The network map of the correlation analysis was drawn using R (version 3.3.1).

### Statistical analysis

The statistical analyses were performed with SPSS 22.0 software, and the data are expressed as the mean ± standard deviations (SDs). One-way ANOVA and Duncan’s multiple range test were used to determine significant mean differences at *P* < 0.05 and 0.001, after ANCOM-BC correction (FDR < 0.05).

## Results

### Analysis of sequencing data and endophytic diversity

After data processing and normalization to the lowest number of sequences, a total of 412,452 high-quality sequences were obtained from the bacterial sequencing results based on the normalized data (15,276), with an average length of 376 bp. A total of 1,084,968 high-quality sequences were obtained from fungal sequencing results based on the normalized data (40,184), with an average length of 243 bp (Table S2). The sequencing coverage of all the samples was greater than 97.60%, indicating that the libraries were sufficient to cover all the microbial groups.

**Figure 1:**
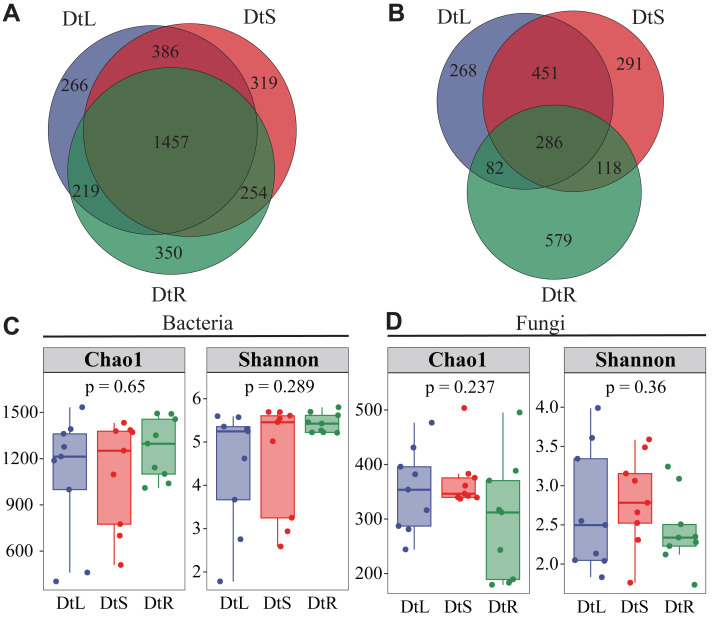
Venn diagram and alpha diversity of endophytic microbiome in leaf (DtL), stem (DtS), and root (DtR) organs of *D. tanguticum*. (A) Venn diagram of endophytic bacteria. (B) Veen diagram of endophytic fungi. (C) Chao1 and Shannon index of endophytic bacteria. (D) Chao1 and Shannon index of endophytic fungi.

As shown in Figs. 1A and 1B, 7,024 bacterial OTUs and 3,298 fungal OTUs were obtained from the organs of *D. tanguticum*. The DtS sample had the greatest number of OTUs (2,416 bacterial OTUs and 1,146 fungal OTUs), whereas the number of OTUs in DtR samples was the lowest (2,280 bacterial OTUs and 1,065 fungal OTUs). A total of 1,457 bacterial OTUs and 286 fungal OTUs coexisted among all the samples, accounting for 20.74% and 8.67%, respectively. These results suggested that there were some differences in the endophytic composition among the leaves, stems, and roots of *D. tanguticum*. The Chao1 and Shannon indices presented in Figs. 1C and 1D show that there were no differences among the organs.

**Figure 2:**
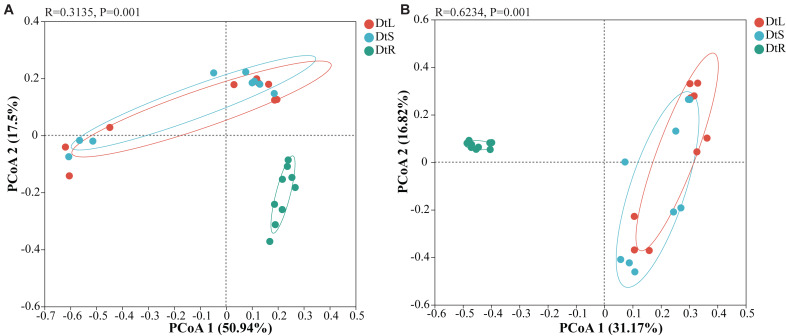
Principal co-ordinates analysis of endophytic bacterial community in leaf (DtL), stem (DtS), and root (DtR) organs of *D. tanguticum*. (A) Bacteria, (B) Fungi. Each point represents one sample, and the different colors represent different organs samples.

The differences in the endophytic microbial compositions of the leaves, stems, and roots of *D. tanguticum* were analyzed using principal coordinate analysis (PCoA) at the genus level (Fig. 2). In the bacterial PCoA, PCoA1 and PCoA2 explained 50.94% and 17.5% of the total variance, respectively (Fig. 2A). In the fungal PCoA, PCoA1 and PCoA2 explained 31.17% and 16.82% of the total variance, respectively (Fig. 2B). The results revealed obvious differences in the different organs. However, the endophytic bacterial and fungal communities in the DtL and DtS samples were similarly distributed, and the endophytic bacterial and fungal communities in the DtR sample were significantly separated from the microbial communities in the DtL and DtS samples (*P* < 0.01). The results indicated that the endophytic microbial community was more similar between the DtL and DtS samples and significantly different from that in the DtR sample.

### Taxonomic composition of endophytes in different organs of *D. tanguticum*

**Figure 3:**
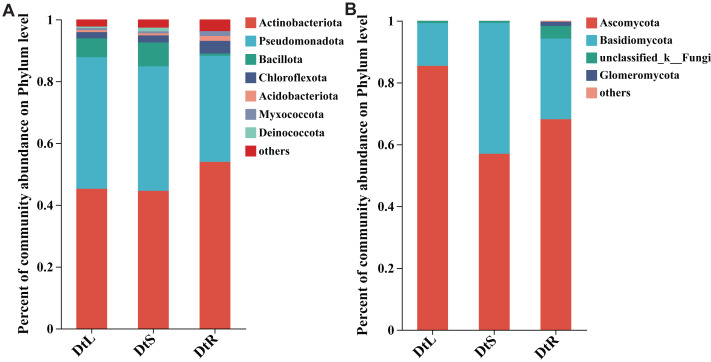
Endophytic bacterial (A) and fungal (B) community in leaf (DtL), stem (DtS), and root (DtR) organs of *D. tanguticum* on phylum level.

**Figure 4:**
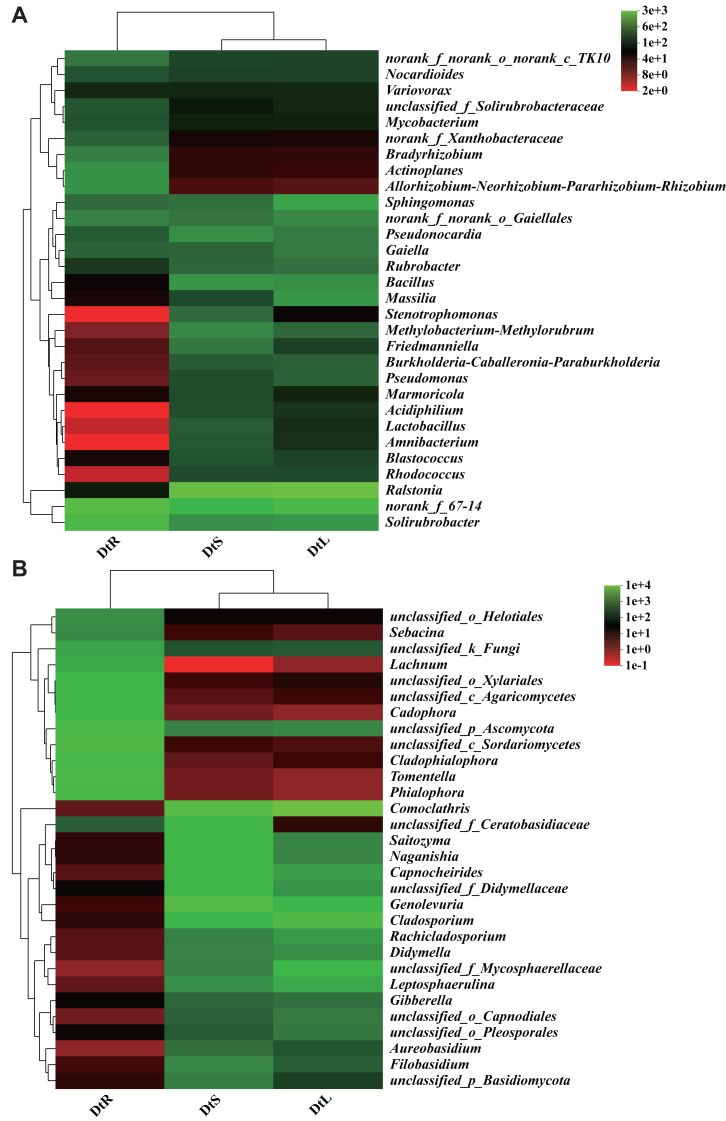
Heatmap of endophytic bacterial (A) and fungal (B) community in leaf (DtL), stem (DtS), and root (DtR) organs of *D. tanguticum* on genus level.

The taxonomic composition of endophytes at the phylum and genus levels was analyzed based on the OTU data. A total of 32 bacterial phyla and 10 fungal phyla were identified. Actinobacteriota (44.56%–53.94%), Pseudomonadota (34.34%–42.60%), and Chloroflexota (2.01%-−4.06%) were the most dominant bacterial phyla (Fig. 3A). Ascomycota (56.97%–85.42%) and Basidiomycota (13.84%–42.35%) were the most dominant fungal phyla (Fig. 3B). The results indicated that the abundance of the dominant phyla differed considerably across the different organs. At the genus level, 616, 629, and 516 bacterial genera and 345, 389, and 309 fungal genera were detected in the leaves, stems, and roots of *D. tanguticum*, respectively (Figs. 4A and 4B). Among them, the stems of *D. tanguticum* presented the greatest number of microbial genera, whereas the roots presented the lowest number. The composition and abundance of the dominant genera (relative abundance >1%) showed considerable differences. For example, DtL had 18 dominant bacterial genera and 15 dominant fungal genera, and the top three bacterial genera were *Ralstonia* (17.45%), *norank_f_67-14* (7.78%), and *Sphingomonas* (4.98%), and the top three fungal genera were *Comoclathris* (33.69%), *Cladosporium* (11.23%), and *Genolevuria* (6.18%). DtS had dominant 27 bacterial genera and 17 fungal genera; *Ralstonia* (15.22%), *norank_f_67-14* (6.19%) and *Bacillus* (3.99%); *Comoclathris* (14.80%), *Genolevuria* (12.46%), and *Saitozyma* (7.11%) were the top three bacterial genera and fungal genera, respectively. DtR had 20 dominant bacterial genera and 14 fungal genera; *norank_f_67-14* (11.62%), *Solirubrobacter* (8.52%), and *Allorhizobium-Neorhizobium-Pararhizobium-Rhizobium* (3.83%) were the three most dominant bacterial genera, and unclassified_c_Sordariomycetes (11.46%), *Tomentella* (10.10%), and *Phialophora* (9.17%) were the top three fungal genera.

### Analysis of the differences in endophytes in different organs of *D. tanguticum*

**Figure 5:**
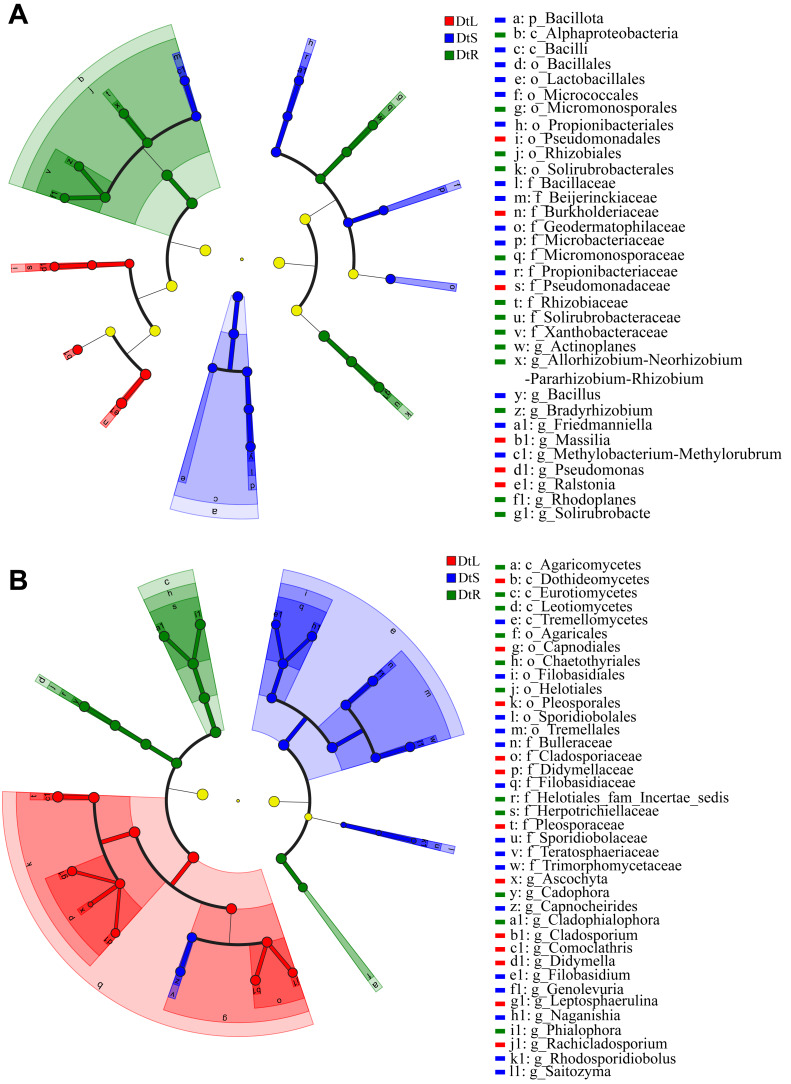
LEfSe analysis of endophytic bacteria (A) and fungi (B) in leaf (DtL), stem (DtS), and root (DtR) organs of *D. tanguticum*.

LEfSe analysis with a threshold of ≥4 was used to identify the differentially abundant endophytes at the genus level and analyze the characteristics of endophytes in the different organs of *D. tanguticum*. A total of 33 significant biomarkers were identified in the bacterial community, as shown in Fig. 5A. Among these genera, *Massilia*, *Pseudomonas*, and *Ralstonia* were present at the highest levels in DtL samples, and *Bacillus*, *Friedmanniella*, and *Methylobacterium-Methylorubrum* peaked in DtS samples, whereas the biomarker bacterial genera from DtR were *Actinoplanes*, *Allorhizobium-Neorhizobium-Pararhizobium-Rhizobium*, *Bradyrhizobium*, *Rhodoplanes*, and *Solirubrobacter*. As shown in Fig. 5B, a total of 38 significant biomarkers were obtained from the fungal community, among which the fungal biomarker taxa from DtL were *Ascochyta*, *Cladosporium*, *Comoclathris*, *Didymella*, *Leptosphaerulina*, and *Rachicladosporium*; the significantly different biomarker taxa from DtS were *Capnocheirides*, *Filobasidium*, *Genolevuria*, *Naganishia*, *Rhodosporidiobolus*, and *Saitozyma*; *Cadophora*, *Cladophialophora*, and *Phialophora* were significantly enriched in DtR samples.

### Co-occurrence network analysis of the endophytic community

**Figure 6:**
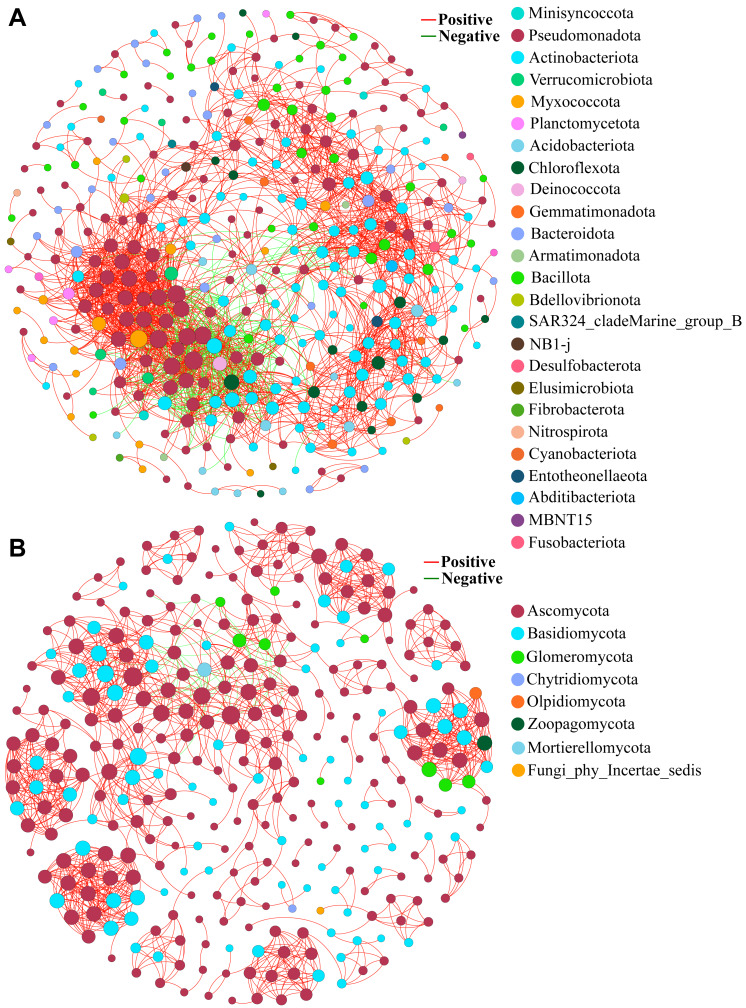
Co-occurrence network analysis of bacterial (A) and fungal (B) community in the organs of *D. tanguticum* at the genus level.

A correlation network analysis was conducted based on Spearman’s correction coefficient, and co-occurrence network indices were calculated to explore the complex interactions in the endophytic community. The results of the co-occurrence network analysis revealed that the positive proportions of the bacterial (89.44%) and fungal (96.63%) communities were mostly higher than the negative proportions. The bacterial network contained 381 nodes and 2,263 edges, and the closeness centrality, betweenness centrality, and average degree were 0.219, 0.007, and 11.879, respectively. The bacterial network had a typical modularity index (0.582), and the nodes were affiliated mainly with Pseudomonadota (34.38%), Actinobacteriota (25.46%), Bacillota (10.24%), and Bacteroidota (6.82%) (Fig. 6A, Table 1). The fungal network contained 326 nodes and 1,305 edges, and the closeness centrality, betweenness centrality, and average degree were 0.096, 0.009, and 8.006, respectively. The fungal network had a typical modularity index (0.828), and the nodes were affiliated mainly with Ascomycota (70.86%), Basidiomycota (24.85%), and Glomeromycota (2.76%) (Fig. 6B, Table 1). These results indicated that the endophytic community in *D. tanguticum* had greater network stability.

### Assembly processes of the endophytic community in different organs

**Figure 7:**
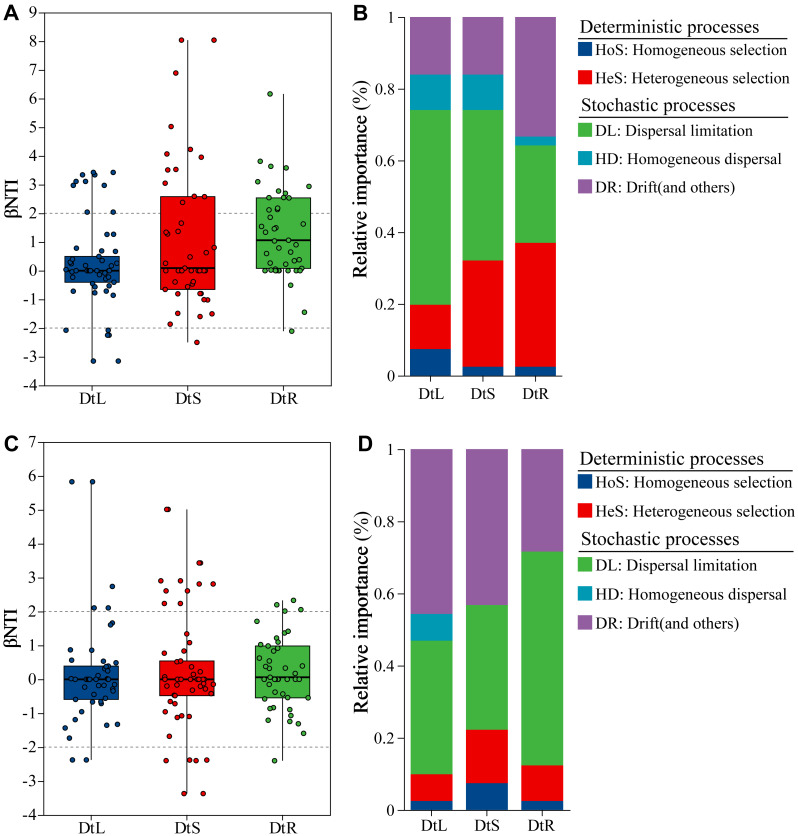
Microbial community assembly in the different organs of *D. tanguticum* using null model analysis. The contribution of stochastic processes (— *β*NTI— <2) and deterministic processes (— *β*NTI— ≥ 2) to assembly processes of endophytic bacterial community (A) and fungal community (C) in the various organs. The relative contribution ratio of different ecological processes to the bacterial (B) and fungal (D) community in the various organs of *D. tanguticum*.

The assembly processes were analyzed using null models to explore the assembly processes of the endophytic community in different organs of *D. tanguticum*. The results showed that stochastic processes (—*β*NTI—<2) dominated the assembly of bacterial and fungal communities in various organs (Figs. 7A and 7C). The analysis of the contributions of microbial communities in different organs revealed that the proportion of dispersal limitation in the bacterial community gradually decreased from leaf organs to root organs and accounted for a higher proportion in the leaf and stem organs. In contrast, the proportion of heterogeneous selection gradually increased from leaf organs to root organs and occupied a larger proportion in the root organs (Fig. 7B). In the fungal community, the proportion of homogeneous selection gradually decreased from leaf organs to root organs, and dispersal limitation of stochastic processes accounted for a greater proportion of the variation in root organs than in other organs (Fig. 7D).

**Table 1:** Co-occurrence network indices of endophytic community.

Endohhytes	Node number	Edge number	Modularity index	Closeness centrality	Betweenness centrality	Average degree	Positive edges	Negative edges
Bacteria	381	2,263	0.582	0.219	0.007	11.879	2,024	239
Fungi	326	1,305	0.828	0.096	0.009	8.006	1,261	44

### Characterization of polyphenolic compounds in different *D. tanguticum* organs

**Figure 8:**
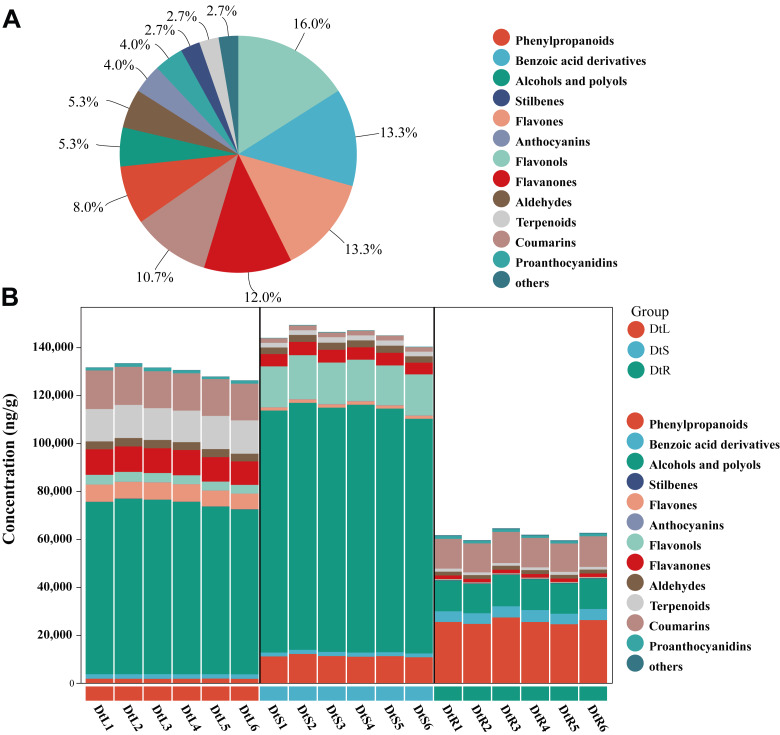
Composition analysis of polyphenolic compound in leaf, stem, and root of *D. tanguticum*.

**Figure 9:**
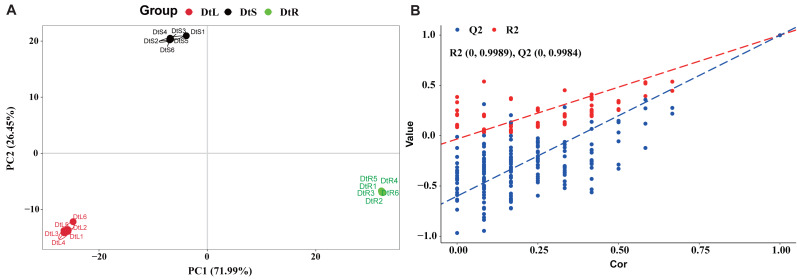
Multivariate statistical analysis of leaf, stem, and root of *D. tanguticum*. (A) PCA score plot of different organs. (B) PLS-DA score plot of different organs. Each point represents one sample, and the different colors represent different organs samples.

Chromatograms of total ion current for various quality control (QC) samples exhibited significant overlap in both retention time and peak intensity (Fig. S1), indicating a steady mass spectrometry (MS) signal over time and affirming the repeatability and reliability of the data. Metabolomic analysis showed that a total of 75 polyphenolic compounds were detected in the leaves (60), stems (55), and roots (57) of *D. tanguticum*, including 6 phenylpropanoids (8.0%), 10 benzoic acid derivatives (13.3%), 4 alcohols and polyols (5.3%), 2 stilbenes (2.7%), 3 anthocyanins (4.0%), 10 flavones (13.3%), 12 flavonols (16.0%), 9 flavanones (12.0%), 4 aldehydes (5.3%), 2 terpenoids (2.7%), 8 coumarins (10.7%), 3 proanthocyanidins (4.0%), and 2 other compounds (2.7%) (Table S3, Fig. 8A). As shown in Fig. 8B, the contents of total polyphenolic compounds differed among the samples; the content of total polyphenolic compounds in the DtS sample was the highest, but that in the DtR sample was the lowest. Moreover, the contents of each compound also differed; for example, the contents of alcohols and polyols were highest in the DtL and DtS samples; however, the content of phenylpropanoids was highest in the DtR sample. PCA further revealed that the polyphenolic compounds in DtL, DtS, and DtR had a high degree of dispersion, indicating that the metabolites in different organs were significantly different (Fig. 9A). The PLS-DA model also indicated that the different organs samples had significantly different metabolite compositions (*P* <  0.01) (Fig. 9B).

### Analysis of differentially abundant polyphenolic compounds in different organs of *D. tanguticum*

**Figure 10:**
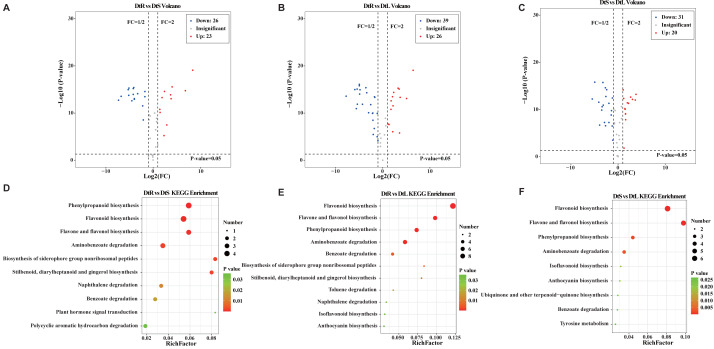
Volcano plots (A–C) and enrichment (D–F) analysis of different metabolites in leaf, stem, and root of *D. tanguticum*.

The differentially abundant polyphenolic compounds were analyzed using ANOVA with *P* < 0.05 after ANCOM-BC correction (FDR < 0.05), and a total of 75 differential polyphenolic compounds were screened in the different organs of *D. tanguticum* (Fig. S3). When we compared the DtR and DtS groups, we screened 49 differentially abundant polyphenolic compounds according to the criteria *P* < 0.05 and —Log2(FC)—> 1, including 23 significantly upregulated and 26 downregulated compounds (Fig. 10A), among which (+/-) catechin, procyanidin B3, acetovanillone, fraxetin, procyanidin B1, cucurbitacin I, epicatechin, 2,4-dihydroxybenzoic acid, quercetin 3-O-glucuronide, 3,3′,4′5-tetrahydroxystilbene and methyl gallate were identified only in the DtR group, whereas sinapaldehyde, narcissin, (-)-epigallocatechin, isorhamnetin, gallic acid, 4-methylumbelliferone, (-)-gallocatechin gallate, jaceosidin, sinapic acid, and proanthocyanidin A2 were identified only in the DtS group (Table S3). In the comparison of the DtR and DtL groups, a total of 65 differentially abundant metabolites were identified, including 26 upregulated and 39 downregulated metabolites (Fig. 10B). The contents of fraxin, caftaric acid, (+/-) catechin, procyanidin B3, scopoletin, caffeic acid, vanillic acid, and 3,4-dihydroxybenzaldehyde were significantly higher in the DtR group, whereas the contents of chlorogenic acid, perillyl alcohol, cryptochlorogenic acid, hesperidin, esculin, and diosmin were significantly higher in the DtL group (Table S3). A total of 51 differentially abundant metabolites were identified between the DtS and DtL groups, among which 20 were upregulated and 31 were downregulated (Fig. 10C). Six polyphenolic compounds were identified only in the DtS group, such as amentoflavone, gallic acid, caftaric acid, isorhamnetin-3-O-glucoside, narcissin, and sinapaldehyde, whereas 11 polyphenolic compounds were identified only in the DtL group, such as pelargonidin-3-glucoside, galangin, delphinidin 3-glucoside, apigenin, 4′,7-di-O-methylnaringenin, 2,4-dihydroxybenzoic acid, sakuranetin, isosakuranetin, naringenin, acetovanillone, and cucurbitacin I. Additionally, flavonol compounds such as quercetin 3-galactoside and astragalin were significantly enriched in the DtS group, and flavone compounds such as jaceosidin, cosmosiin, and luteolin were significantly enriched in the DtL group. KEGG pathway enrichment analysis of the differentially abundant metabolites was conducted to understand the metabolic pathways associated with the significantly differentially abundant metabolites. A total of 15 pathways were enriched among the differentially abundant metabolites (Figs. 10D–10F), such as phenylpropanoid biosynthesis; flavonoid biosynthesis; flavone and flavonol biosynthesis; aminobenzoate degradation; biosynthesis of siderophore group nonribosomal peptides; stilbenoid, diarylheptanoid and gingerol biosynthesis; naphthalene degradation; benzoate degradation; plant hormone signal transduction; polycyclic aromatic hydrocarbon degradation; toluene degradation; isoflavonoid biosynthesis; anthocyanin biosynthesis; ubiquinone and other terpenoid-quinone biosynthesis; and tyrosine metabolism. These pathways drive changes in the levels of polyphenolic compounds in different organs.

### Correlation analysis of microorganisms and metabolites in different organs of *D. tanguticum*

**Figure 11:**
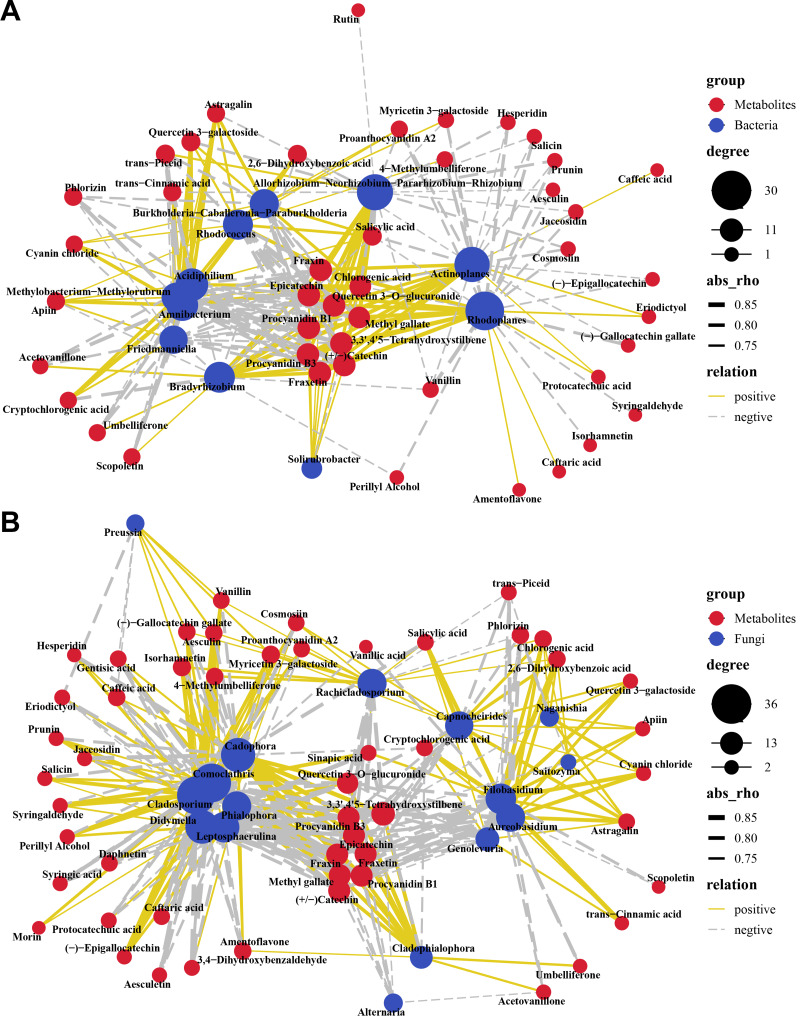
Correlation analysis of top 20 bacteria (A) and fungi (B) and metabolites. The red circle represents metabolites, blue circle represents microbial genus.

Endophytes play a direct or indirect role in the accumulation of metabolites in plants (Ling et al., 2022). The top 20 differentially abundant microorganisms were plotted against 75 polyphenolic compounds in a correlation network diagram using the thresholds absolute correlation coefficient —r—> 0.7 and *P* <  0.05 to understand the intricate relationships between microbes and polyphenolic compounds (Figs. 11A and 11B). The correlation network analysis indicated that 11 bacterial genera and 16 fungal genera were significantly correlated with 44 and 51 metabolites, respectively. In the correlation analysis of bacteria and polyphenolic compounds, the vast majority of bacteria and metabolites showed significant negative correlations (Fig. 11A); for example, *Rhodococcus* was positively and significantly correlated with 9 metabolites (chlorogenic acid, quercetin 3-galactoside, Astragalin, *etc.*) and negatively correlated with 11 metabolites (catechin, quercetin 3-O-glucuronide, epicatechin, and methyl gallate, *etc.*), whereas *Friedmanniella* was positively and significantly correlated with 4 metabolites (cryptochlorogenic acid, apiin, quercetin 3-galactoside and trans-cinnamic acid) and negatively correlated with 14 metabolites (scopoletin, procyanidin B3, fraxetin, *etc.*). However, *Solirubrobacter* was positively and significantly correlated with 8 metabolites (fraxin, catechin, fraxetin, *etc.*) and was negatively correlated with only chlorogenic acid. In the correlation analysis of fungi and polyphenolic compounds, *Cladosporium*, *Comoclathris*, *Didymella*, *Leptosphaerulina*, and *Rachicladosporium* (the biomarkers of DtL) were significantly positively correlated with 16, 16, 12, 6, and 12 metabolites and negatively correlated with 19, 20, 17, 19, and 11 metabolites, respectively. *Capnocheirides*, *Filobasidium*, *Genolevuria*, *Naganishia*, and *Saitozyma* (the biomarkers of DtS) were significantly positively correlated with 11, 10, 6, 6, and 3 metabolites and negatively correlated with 11, 15, 10, 3, and 2 metabolites, respectively. *Cadophora*, *Cladosporium*, and *Phialophora* (the biomarkers of DtR) were significantly positively correlated with 14, 16, and 17 metabolites and negatively correlated with 17, 19, and 6 metabolites, respectively.

## Discussion

Endophytes play a direct or indirect role in host growth and development, improve defenses against abiotic and biotic stress, and synthesize metabolites (Chen et al., 2019; Shan et al., 2024; Singh et al. , 2019). Endophytes have typical host preferences and organs specificities; for example, the diversity of endophytes in the leaves of *Arnebia euchroma* is greater than that in the roots (Chen et al., 2025), the endophytic diversity in the fibrils of *Panax quinquefolius* is greater than that in other organs (Li et al., 2023), the diversity of endophytic bacteria in the roots and fungi in the stems of *Gentiana officinalis* is relatively high (Hou et al., 2022). In this study, the endophytic bacterial richness in the roots and fungal richness in the leaves of *D. tanguticum* were relatively high, and the diversity of endophytes in the stems was relatively high; however, no significant difference was observed among the different organs. The results indicated that various factors influenced the community diversity of endophytes in the different organs of *D. tanguticum*. PCoA revealed obvious differences across different organs, and the endophytic communities in the leaves and stems were similar, while the endophytic communities in the roots were significantly separated from the microbial communities in the leaves and stems. This result is consistent with other studies in which the microbial communities were different between aboveground and belowground plant organs (Abdelfattah et al., 2021), and the endophytic communities in the leaves and stems of *Gentiana officinalis* were more similar than those in the roots (Hou et al., 2022).

Previous studies have shown that endophytic bacteria and fungi are concentrated in a few phyla, such as Actinobacteriota, Pseudomonadota, Ascomycota, and Basidiomycota (Li et al., 2023; Miguel et al., 2016). In this study, a total of 32 bacterial phyla and 10 fungal phyla were identified. Actinobacteriota and Pseudomonadota were the dominant bacterial phyla, and the dominant fungal phyla were Ascomycota and Basidiomycota. Similar results were reported for *Gentiana officinalis*, *Dendrobium*, *Rheum palmatum*, and *Panax ginseng* (Hou et al., 2022; Wang et al., 2022; Chen et al., 2023; Fan et al., 2022); however, the relative abundances of the dominant phyla differed significantly across the different organs. These results indicate that the endophytic community has a certain degree of organs specificity. Previous studies have shown that most species of Pseudomonadota and Actinobacteriota play crucial roles in the nitrogen and sulfur cycles, antibacterial activity, and host metabolism (Ansola, Arroyo & Sáenz, 2013; Bashir, Iqbal & Hasnain, 2020), whereas Ascomycota and Basidiomycota might participate in nutrient cycling (Chen et al., 2025). The relative abundance of Actinobacteriota was higher in the roots, potentially to inhibit some pathogenic bacteria and maintain the stability of the endophytic community. Hou et al. (2022) reported that the endophytic fungal and bacterial communities in the leaves and stems of *G. officinalis* were obviously clustered but were different from those in the roots, which is consistent with our results. Chen et al. (2023) reported that the bacterial composition of the leaf and root organs of *R. palmatum* was obviously clustered, which is different from our results. The results indicated that the endophytes have typical host preferences and organs specificities.

At the genus level, 616, 629, and 516 bacterial genera and 345, 389, and 309 fungal genera were detected in the leaves, stems, and roots of *D. tanguticum*, respectively. *Ralstonia* (17.45%), *Comoclathris* (33.69%), *Cladosporium* (11.23%), and *Genolevuria* (6.18%) were the dominant genera in the leaf organs; *Ralstonia* (15.22%), *Comoclathris* (14.80%), and *Genolevuria* (12.46%) were the dominant genera in the stem organs; and *Solirubrobacter* (8.52%), *Tomentella* (10.10%), and *Phialophora* (9.17%) were the dominant genera in the root organs. These results are consistent with those of previous studies in which the dominant genera were significantly different in various organs (Chen et al., 2023; Li et al., 2025). Previous studies have shown that some *Ralstonia* and *Comoclathris* species are plant pathogens, such as *Ralstonia solanacearum* and *Comoclathris incompta* (Caldwell, Kim & Iyer-Pascuzzi, 2017; Moral et al., 2017); however, some *Ralstonia* species, such as *Ralstonia pickettii*, can increase 1,1,1-trichloro-2,2-bis(4-chlorophenyl) ethane (DTT)biodegradation and potentially remediate environmental pollution (Adi Setyo, Diana & Ichiro , 2019; Ryan, Pembroke & Adley, 2010). *Ralstonia* and *Comoclathris* were the dominant genera in the leaf and stem organs, and thus *D. tanguticum* might be at risk of pathogenic bacterial infection. *Solirubrobacter* can promote the absorption of nutrients and plant growth (Jeewani et al., 2021), and *Tomentella*, a genus of ectomycorrhizal fungi, plays an important role in the absorption of nutrients (Suetsugu et al., 2021). *Solirubrobacter* and *Tomentella* were significantly enriched in the root tissue, which might help *D. tanguticum* absorb nutrients.

The co-occurrence network analysis revealed greater numbers of nodes and edges, as well as greater closeness centrality and average degree, in the endophytic bacterial community than in the fungal community, indicating that the bacterial community was more complex than the fungal community. These results are consistent with those of previous studies on the roots of *Stellera chamaejasme* (Zhang et al., 2024a; Zhang et al., 2024b). The endophytic community has a typical modular structure, reflecting different ecological niches and functions. Stochastic processes dominated the assembly of bacterial and fungal communities in various organs of *D. tanguticum*. Microbiome assembly is influenced by various factors, such as plant species, tissue niches, and seasons (He et al., 2023). Furthermore, a few studies have shown that active components in medicinal plants affect the endophytic community assembly process; in turn, endophytes also affect the contents of active components in medicinal plants (Tang et al., 2024). *D. tanguticum* is a traditional medicinal plant enriched with various chemical constituents, such as flavonoids, terpenoids, polysaccharides and volatile components (Zeng et al., 2010). In this study, a total of 75 polyphenolic compounds were detected in the leaves (60), stems (55), and roots (57) of *D. tanguticum*, and the differences in polyphenolic compounds among various organs might influence the endophytic community assembly process.

The whole plant of *D. tanguticum* is used for medicinal purposes, but the contents of polyphenolic compound were significantly different in various organs; the contents of polyphenolic compound in the stem were higher than those in other organs, and those in the root were the lowest. A total of 49 differentially abundant polyphenolic compounds (23 up- and 26 downregulated) were identified between the DtR and DtS groups, 65 differentially abundant metabolites (26 up- and 39 downregulated) were identified between the DtR and DtL groups, and 51 differentially abundant metabolites (20 up- and 31 downregulated) were identified between the DtS and DtL groups. Consistent with the findings of previous studies, the metabolite levels were significantly different in the root and leaf organs of *Arnebia euchroma* (Chen et al., 2025), the metabolite contents in the different organs of *Panax quinquefolius* also differed significantly (Li et al., 2023). Thus, the content of polyphenol compounds exhibited organs specificity in *D. tanguticum*. Endophytes are directly or indirectly involved in the biosynthesis of active ingredients in medicinal plants (Ling et al., 2022); for example, the content of flavonoids in *Paeonia lactiflora* is correlated with the abundance of *Ruminococcaceae bacterium GD7* (Yang et al., 2023). In *P. quinquefoliu* s, endophytes are significantly positively and negatively correlated with metabolites (Li et al., 2023). In *A. euchroma*, a significant correlation has been observed between endophytes and active components (Chen et al., 2025); thus, endophytes can be used as potential substitutes for bioactive metabolites. In this study, the intricate relationships between the top 20 microbes and polyphenol compounds were analyzed. The results indicated that 11 bacterial and 16 fungal genera were significantly correlated with 44 and 51 metabolites, respectively. For example, *Solirubrobacter* was positively and significantly correlated with 8 metabolites (fraxin, catechin, fraxetin, *etc.*) and was negatively correlated with only chlorogenic acid. *Cladosporium*, *Comoclathris*, *Didymella*, *Leptosphaerulina*, and *Rachicladosporium* were significantly positively correlated with 16, 16, 12, 6, and 12 metabolites and negatively correlated with 19, 20, 17, 19, and 11 metabolites, respectively, which is consistent with previous studies. These results indicate that endophytes may play critical roles in the accumulation of metabolites and that the endophyte abundance and metabolite content interact with each other. However, the specific correlations between endophytes and bioactive metabolites need further study.

## Conclusions

In summary, the compositions of the endophytic communities in the leaf and stem organs of *D. tanguticum* were similar, and the endophytic community diversity did not differ significantly across the various organs. Actinobacteriota, Pseudomonadota, Ascomycota, and Basidiomycota were the dominant endophytes in the different organs, while the relative abundances of the dominant endophytic phyla and the dominant endophytic genera were significantly different in the various organs of *D. tanguticum*. The interactions in the endophytic community were mainly positive. Stochastic processes predominantly dominated the endophytic community assembly in various organs of *D. tanguticum*. The contents of polyphenolic compound in the different organs of *D. tanguticum* were significantly different: those in the stem organs were higher than those in the other organs, and those in the root organs were lower. Varying degrees of positive and negative correlations were observed between endophytes and polyphenolic compounds, which provides a theoretical basis for identifying and applying functional microbiomes in *D. tanguticum*.

##  Supplemental Information

10.7717/peerj.21626/supp-1 Supplemental Information 1 Standard information

10.7717/peerj.21626/supp-2 Supplemental Information 2 Sequencing data processing statistics

10.7717/peerj.21626/supp-3 Supplemental Information 3 Composition analysis of polyphenolic compounds in different organs of *D. tanguticum*

10.7717/peerj.21626/supp-4 Supplemental Information 4 Total ion current

10.7717/peerj.21626/supp-5 Supplemental Information 5 Calibration curve

10.7717/peerj.21626/supp-6 Supplemental Information 6 Analysis of differential polyphenolic compounds in different organs of *D. tanguticum*

## Additional Information and Declarations

### DNA Deposition

The following information was supplied regarding the deposition of DNA sequences: The raw sequence data are available at NCBI BioProject: PRJNA1281808, PRJNA1281329.

### Data Availability

The following information was supplied regarding data availability: The Metabolomics data are available at CNCB OMIX: OMIX 016964 – https://ngdc.cncb.ac.cn/omix/release/OMIX016964
The raw sequence data are available at NCBI BioProject: PRJNA1281808, PRJNA1281329
