# Supplementary material for: Characterization of the microbiome and polyphenolic compounds in the medicinal plant Dracocephalum tanguticum
Source: PeerJ. 2026 Jul 29;14:e21626. doi: 10.7717/peerj.21626 (PMC13428542; doi:10.7717/peerj.21626)
Supplement: Supplemental Information 2 [file peerj-14-21626-s002.doc]

| Sample Name | Endophytic bacteria | | | | |  | Endophytic fungi | | | | |
| --- | --- | --- | --- | --- | --- | --- | --- | --- | --- | --- | --- |
|  | Valid sequence | OTU | Chao1 | Shannon | Good_coverage (%) |  | Valid sequence | OTU | Chao1 | Shannon | Good_coverage (%) |
| DtL1 | 15276 | 800 | 999.238 | 3.666 | 98.30 |  | 40184 | 406 | 431.000 | 3.610 | 99.90 |
| DtL2 | 15276 | 353 | 460.409 | 2.754 | 99.20 |  | 40184 | 435 | 476.600 | 3.987 | 99.80 |
| DtL3 | 15276 | 355 | 402.468 | 1.779 | 99.40 |  | 40184 | 378 | 395.818 | 3.343 | 99.90 |
| DtL4 | 15276 | 1156 | 1533.443 | 5.571 | 97.60 |  | 40184 | 253 | 316.088 | 2.135 | 99.80 |
| DtL5 | 15276 | 944 | 1360.966 | 4.617 | 97.70 |  | 40184 | 266 | 353.778 | 1.833 | 99.80 |
| DtL6 | 15276 | 1066 | 1391.380 | 5.245 | 97.80 |  | 40184 | 278 | 381.917 | 2.497 | 99.80 |
| DtL7 | 15276 | 994 | 1275.866 | 5.353 | 98.00 |  | 40184 | 242 | 281.079 | 2.548 | 99.90 |
| DtL8 | 15276 | 1038 | 1213.273 | 5.593 | 98.40 |  | 40184 | 209 | 244.000 | 2.047 | 99.90 |
| DtL9 | 15276 | 943 | 1187.101 | 5.303 | 98.30 |  | 40184 | 242 | 287.026 | 2.041 | 99.90 |
| DtS1 | 15276 | 654 | 774.744 | 2.936 | 98.80 |  | 40184 | 341 | 346.440 | 3.488 | 99.90 |
| DtS2 | 15276 | 539 | 699.575 | 3.248 | 98.70 |  | 40184 | 448 | 503.561 | 3.586 | 99.80 |
| DtS3 | 15276 | 414 | 509.485 | 2.586 | 99.10 |  | 40184 | 261 | 361.088 | 2.307 | 99.80 |
| DtS4 | 15276 | 1135 | 1370.877 | 5.456 | 97.90 |  | 40184 | 300 | 339.245 | 3.061 | 99.80 |
| DtS5 | 15276 | 819 | 1096.619 | 5.015 | 98.30 |  | 40184 | 278 | 339.737 | 1.762 | 99.80 |
| DtS6 | 15276 | 1179 | 1378.333 | 5.69 | 98.00 |  | 40184 | 293 | 336.977 | 3.153 | 99.80 |
| DtS7 | 15276 | 1130 | 1385.277 | 5.604 | 98.00 |  | 40184 | 274 | 375.528 | 2.782 | 99.80 |
| DtS8 | 15276 | 1154 | 1432.470 | 5.689 | 97.90 |  | 40184 | 322 | 382.769 | 2.661 | 99.80 |
| DtS9 | 15276 | 1048 | 1251.728 | 5.541 | 98.30 |  | 40184 | 271 | 342.154 | 2.522 | 99.80 |
| DtR1 | 15276 | 869 | 1141.297 | 5.265 | 98.30 |  | 40184 | 223 | 316.840 | 2.338 | 99.80 |
| DtR2 | 15276 | 1075 | 1351.124 | 5.615 | 98.00 |  | 40184 | 194 | 243.136 | 2.348 | 99.90 |
| DtR3 | 15276 | 1115 | 1456.017 | 5.423 | 97.70 |  | 40184 | 393 | 495.375 | 3.087 | 99.80 |
| DtR4 | 15276 | 1158 | 1492.693 | 5.8 | 97.60 |  | 40184 | 243 | 312.081 | 2.276 | 99.80 |
| DtR5 | 15276 | 1029 | 1297.190 | 5.452 | 98.00 |  | 40184 | 142 | 179.188 | 2.121 | 99.90 |
| DtR6 | 15276 | 1139 | 1490 | 5.694 | 97.70 |  | 40184 | 337 | 388.383 | 3.241 | 99.80 |
| DtR7 | 15276 | 809 | 1009.597 | 5.221 | 98.60 |  | 40184 | 295 | 370.477 | 2.504 | 99.80 |
| DtR8 | 15276 | 815 | 1100.058 | 5.219 | 98.40 |  | 40184 | 121 | 182.875 | 2.228 | 99.90 |
| DtR9 | 15276 | 764 | 1038.794 | 5.21 | 98.40 |  | 40184 | 140 | 189.136 | 1.735 | 99.90 |
