# Supplementary material for: Characterization of the microbiome and polyphenolic compounds in the medicinal plant Dracocephalum tanguticum
Source: PeerJ. 2026 Jul 29;14:e21626. doi: 10.7717/peerj.21626 (PMC13428542; doi:10.7717/peerj.21626)
Supplement: Supplemental Information 3 [file peerj-14-21626-s003.docx]

Table S3 Composition analysis of polyphenolic compounds in different organs of *D. tanguticum*

| Categories | Metabolites name | DtL (ng/g) | DtS (ng/g) | DtR (ng/g) |
| --- | --- | --- | --- | --- |
| Phenylpropanoids | Sinapic acid | 96.12±10.7 | 90.66±25.23 | 0 |
|  | trans-Cinnamic acid | 255.24±11.19 | 997.24±46.41 | 44.03±5.54 |
|  | 4-Hydroxycinnamic acid | 340.29±7.43 | 771.58±21.17 | 505.69±17.63 |
|  | Caffeic acid | 701.94±20.76 | 8725.48±450.42 | 23327.95±991.73 |
|  | Caftaric acid | 0 | 9.65±0.47 | 1103.87±32.7 |
|  | Ferulic acid | 394.02±23.86 | 648±26.65 | 625.97±31.08 |
| Benzoic acid derivatives | 2,4-Dihydroxybenzoic acid | 20.75±8.77 | 0 | 20.67±4.65 |
|  | Protocatechuic acid | 164.68±5.31 | 186.8±5.46 | 695.29±20.62 |
|  | 2,6-Dihydroxybenzoic acid | 66.05±9.00 | 185.05±5.52 | 19.1±1.6 |
|  | Salicylic acid | 181.77±4.63 | 182.43±4.63 | 54.48±2.21 |
|  | Syringic acid | 234.72±19.1 | 699.76±47.56 | 1028.71±181.92 |
|  | Vanillic acid | 183.53±45.82 | 182.53±16.21 | 2380.27±78.8 |
|  | 4-Hydroxybenzoic acid | 1025.75±26.67 | 191.9±9.41 | 321.06±11.73 |
|  | Gallic acid | 0 | 10.16±1.27 | 0 |
|  | Gentisic acid | 6.65±5.32 | 15.89±5.59 | 78.81±16.94 |
|  | Methyl gallate | 0 | 0 | 4.21±0.41 |
| Alcohols and polyols | Salicin | 2901.21±112.97 | 482.49±17.52 | 13.49±1.46 |
|  | Chlorogenic acid | 38358.38±1144.15 | 62843.56±1516.28 | 3676.59±78.52 |
|  | Cryptochlorogenic acid | 29411.97±809.8 | 36602.2±936.53 | 1049.02±32.32 |
|  | 3,4-Dihydroxybenzaldehyde | 711.45±17.85 | 1506.3±20.43 | 7993.49±200.42 |
| Stilbenes | 3,3',4'5-Tetrahydroxystilbene | 0 | 0 | 5.15±1.68 |
|  | trans-Piceid | 72.85±12.26 | 11.49±2.22 | 161.44±6.26 |
| Anthocyanins | Cyanin chloride | 25.08±4.78 | 115.79±3.61 | 6.24±1.55 |
|  | Delphinidin 3-glucoside | 14.46±2.87 | 0 | 0 |
|  | Pelargonidin-3-glucoside | 7.31±0.77 | 0 | 0 |
| Flavones | Vitexin | 47.62±5.53 | 20.07±1.24 | 34.33±1.55 |
|  | Amentoflavone | 0 | 22.87±0.14 | 28.61±0.49 |
|  | Apigenin | 16.75±0.44 | 0 | 0 |
|  | Apiin | 49.82±6.07 | 151.35±13.84 | 35.91±4.54 |
|  | Cosmosiin | 2799.73±158.2 | 1066.87±43.87 | 67.66±3.74 |
|  | Galangin | 13.81±7.12 | 0 | 0 |
|  | Isoorientin | 81.41±4.53 | 9.21±2.58 | 18.13±3.55 |
|  | Luteolin | 200.52±4.53 | 6.5±0.44 | 6.46±0.49 |
|  | Orientin | 72.68±13.11 | 4.81±2.27 | 36.07±4.54 |
|  | Jaceosidin | 3671.33±187.25 | 82.56±40.81 | 0 |
| Flavonols | Prunin | 985.55±26.5 | 424.84±10.59 | 22.98±2.3 |
|  | Quercetin 3-galactoside | 981.63±75.07 | 13622.16±532.03 | 124.1±8.23 |
|  | Quercetin 3-O-glucuronide | 0 | 0 | 18.23±1.38 |
|  | Rutin | 16.87±2.64 | 14.78±10.69 | 7.48±1.02 |
|  | Astragalin | 367.04±20.06 | 3031.06±143.51 | 18.84±2.7 |
|  | Isorhamnetin | 36.05±1.71 | 9.35±0.41 | 0 |
|  | Isorhamnetin-3-O-glucoside | 0 | 7.16±0.62 | 6.33±0.85 |
|  | Isosakuranetin | 24.54±1.24 | 0 | 0 |
|  | Morin | 2.03±0.14 | 1.67±0.14 | 1.55±0.06 |
|  | Myricetin 3-galactoside | 1410.8±99.5 | 135.32±6.16 | 29.25±2.57 |
|  | Narcissin | 0 | 6.48±1.5 | 0 |
|  | Aromadendrin | 6.36±0.35 | 3.7±0.34 | 4.18±0.43 |
| Flavanones | (-)-Epigallocatechin | 17.64±4.16 | 8.1±1.32 | 0 |
|  | (-)-Gallocatechin gallate | 510.33±42.11 | 46.07±7.55 | 0 |
|  | (+/-) Catechin | 0 | 0 | 1085.64±29.61 |
|  | Epicatechin | 0 | 0 | 24.44±3.43 |
|  | Sakuranetin | 24.05±1.16 | 0 | 0 |
|  | 4',7-Di-O-methylnaringenin | 18.59±1.04 | 0 | 0 |
|  | Eriodictyol | 6.07±0.29 | 7.35±0.48 | 20.5±1.04 |
|  | Hesperidin | 9650.69±329.23 | 5153.44±219.35 | 325.24±7.15 |
|  | Naringenin | 120.49±5.55 | 0 | 0 |
| Aldehydes | Syringaldehyde | 514.53±33.56 | 186.78±18.96 | 120.3±10.97 |
|  | Vanillin | 2775.1±134.42 | 2225.59±116.68 | 1249.06±35.64 |
|  | Sinapaldehyde | 0 | 3.69±0.11 | 0 |
|  | Coniferaldehyde | 70.16±2.96 | 442.32±17.87 | 242.01±7.89 |
| Terpenoids | Cucurbitacin I | 172.99±16.67 | 0 | 41.16±2.63 |
|  | Perillyl Alcohol | 13408.21±312.23 | 2059.36±158.49 | 1131.35±55.15 |
| Coumarins | Scopoletin | 35.6±2.2 | 9.25±3.95 | 3106.23±33.84 |
|  | Umbelliferone | 53.4±3.11 | 31.2±2.31 | 509.72±11.65 |
|  | 4-Methylumbelliferone | 241.34±14.1 | 14.21±1.62 | 0 |
|  | Aesculetin | 48.86±2.97 | 132.5±6.51 | 222.2±10.46 |
|  | Aesculin | 15178.95±321.43 | 1630.51±26.43 | 490.01±23.75 |
|  | Daphnetin | 50.65±3.05 | 150.25±10.76 | 255.95±5.94 |
|  | Fraxetin | 0 | 0 | 108.7±11 |
|  | Fraxin | 0 | 0 | 7704.48±363.93 |
| Proanthocyanidins | Procyanidin B1 | 0 | 0 | 55.98±6.26 |
|  | Procyanidin B3 | 0 | 0 | 1020.61±61.8 |
|  | Proanthocyanidin A2 | 1193.56±174.67 | 161.16±27.32 | 0 |
| Other | Acetovanillone | 122.8±8.36 | 0 | 311.91±28.86 |
|  | Phlorizin | 11.63±0.66 | 10.41±0.7 | 15.11±0.68 |
