# Supplementary figures and images for: Characterization of the microbiome and polyphenolic compounds in the medicinal plant Dracocephalum tanguticum
Source: PeerJ. 2026 Jul 29;14:e21626. doi: 10.7717/peerj.21626 (PMC13428542; doi:10.7717/peerj.21626)

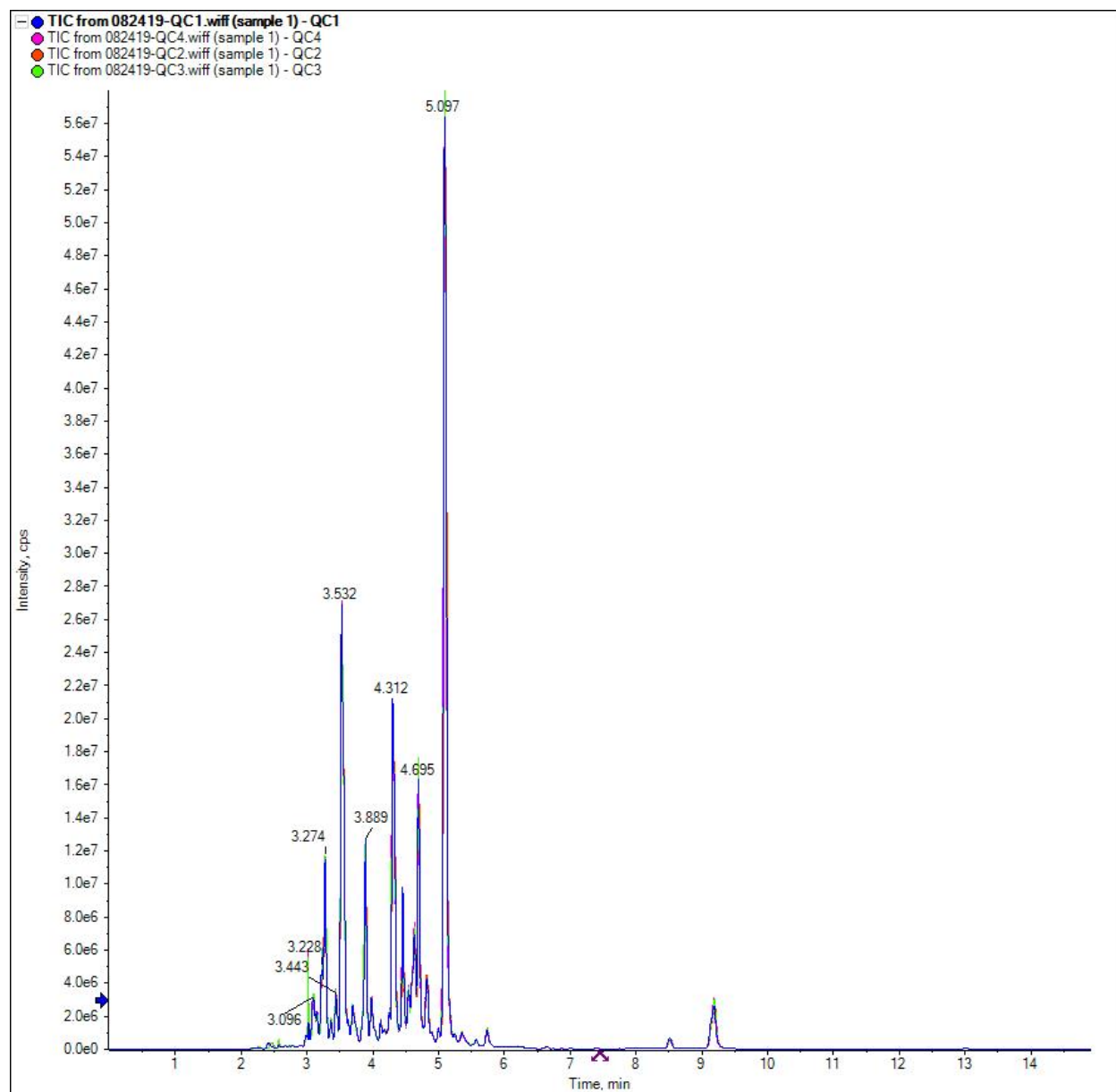

Figure S1 Overlapping total ion current of QC sample

Supplement: Supplemental Information 4 [file peerj-14-21626-s004.pdf]

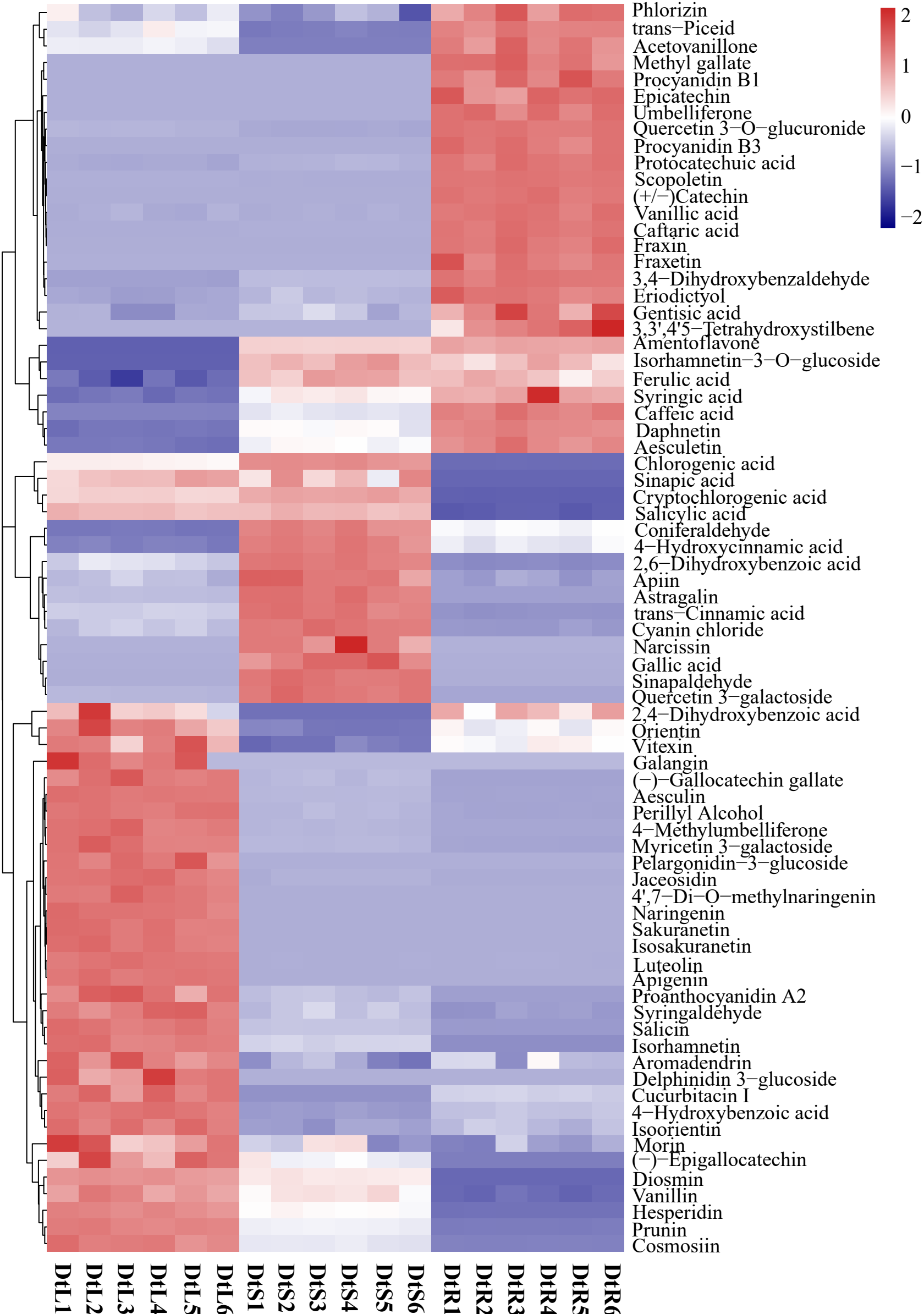

Supplement: Supplemental Information 6 [file peerj-14-21626-s006.pdf]
