# Supplementary material for: Characterization of the microbiome and polyphenolic compounds in the medicinal plant Dracocephalum tanguticum
Source: PeerJ. 2026 Jul 29;14:e21626. doi: 10.7717/peerj.21626 (PMC13428542; doi:10.7717/peerj.21626)
Supplement: Supplemental Information 5 [file peerj-14-21626-s005.pdf]

SCIEX

Created with SCIEX OS 1.6.1
Printed:9/18/2023 5:27:01 PM

Analyte Name: LM-flavones-01_1
Internal Standard: LM-flavones-IS02_1

**Data File**	flavones-STD-20230908.wiff	**Result Table**	DZLM2023082419-results-20230913-5500
**Acquisition Date**	9/8/2023 5:37:08 PM	**Algorithm Used**	MQ4
**Acquisition Method**	20230908-flavones-(mix130-T3)-15min.dam	**Instrument Name**	QTRAP 6500+ Low Mass
**Project**	N/A	**Processing Method**	20230412-flavones-(mix130-T3)-15min.qmethod

Regression Equation: y = 0.00248 x + -0.00234 (r = 0.99941, r^2 = 0.99881) (weighting: 1 / x)

Expected Concentration	Number of Values	Mean Calculated Concentration (No data for Analyte Unit)	% Accuracy	Std. Deviation	%CV
0.01	0 of 1	N/A	N/A	N/A	N/A
0.02	0 of 1	N/A	N/A	N/A	N/A
0.05	0 of 1	N/A	N/A	N/A	N/A
0.13	0 of 1	N/A	N/A	N/A	N/A
0.33	0 of 1	N/A	N/A	N/A	N/A
0.82	0 of 1	N/A	N/A	N/A	N/A
2.05	0 of 1	N/A	N/A	N/A	N/A
5.12	1 of 1	5.722e0	111.8	N/A	N/A
12.80	1 of 1	1.172e1	91.6	N/A	N/A
32.00	1 of 1	3.129e1	97.8	N/A	N/A
80.00	1 of 1	7.775e1	97.2	N/A	N/A
200.00	1 of 1	2.034e2	101.7	N/A	N/A

Analyte Name: LM-flavones-01_1

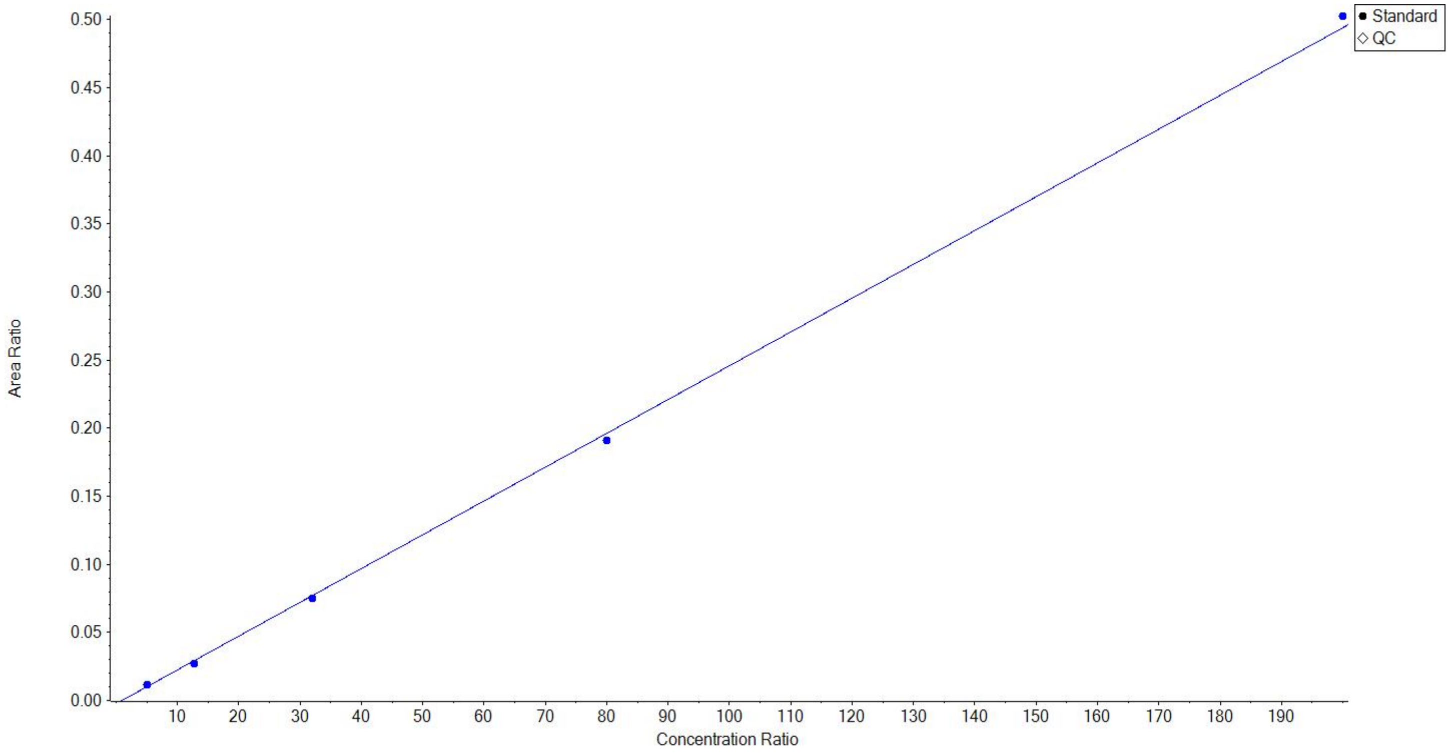
0.50
0.45
0.40
0.35
0.30
0.25
0.20
0.15
0.10
0.05
0.00
Area Ratio
10
20
30
40
50
60
70
80
90
100
110
120
130
140
150
160
170
180
190
Concentration Ratio
Standard
QC

Analyte Name: LM-flavones-02_1
Internal Standard: LM-flavones-IS02_1

**Data File**	flavones-STD-20230908.wiff	**Result Table**	DZLM2023082419-results-20230913-5500
**Acquisition Date**	9/8/2023 5:37:08 PM	**Algorithm Used**	MQ4
**Acquisition Method**	20230908-flavones-(mix130-T3)-15min.dam	**Instrument Name**	QTRAP 6500+ Low Mass
**Project**	N/A	**Processing Method**	20230412-flavones-(mix130-T3)-15min.qmethod

Regression Equation: y = 0.00357 x + -3.48463e-4 (r = 0.99978, r² = 0.99957) (weighting: 1 / x)

Expected Concentration	Number of Values	Mean Calculated Concentration (No data for Analyte Unit)	% Accuracy	Std. Deviation	%CV
0.01	0 of 1	N/A	N/A	N/A	N/A
0.02	0 of 1	N/A	N/A	N/A	N/A
0.05	0 of 1	N/A	N/A	N/A	N/A
0.13	0 of 1	N/A	N/A	N/A	N/A
0.33	0 of 1	N/A	N/A	N/A	N/A
0.82	0 of 1	N/A	N/A	N/A	N/A
2.05	1 of 1	2.137e0	104.2	N/A	N/A
5.12	1 of 1	5.145e0	100.5	N/A	N/A
12.80	1 of 1	1.274e1	99.6	N/A	N/A
32.00	1 of 1	3.089e1	96.6	N/A	N/A
80.00	1 of 1	7.819e1	97.7	N/A	N/A
200.00	1 of 1	2.029e2	101.4	N/A	N/A

Analyte Name: LM-flavones-02_1

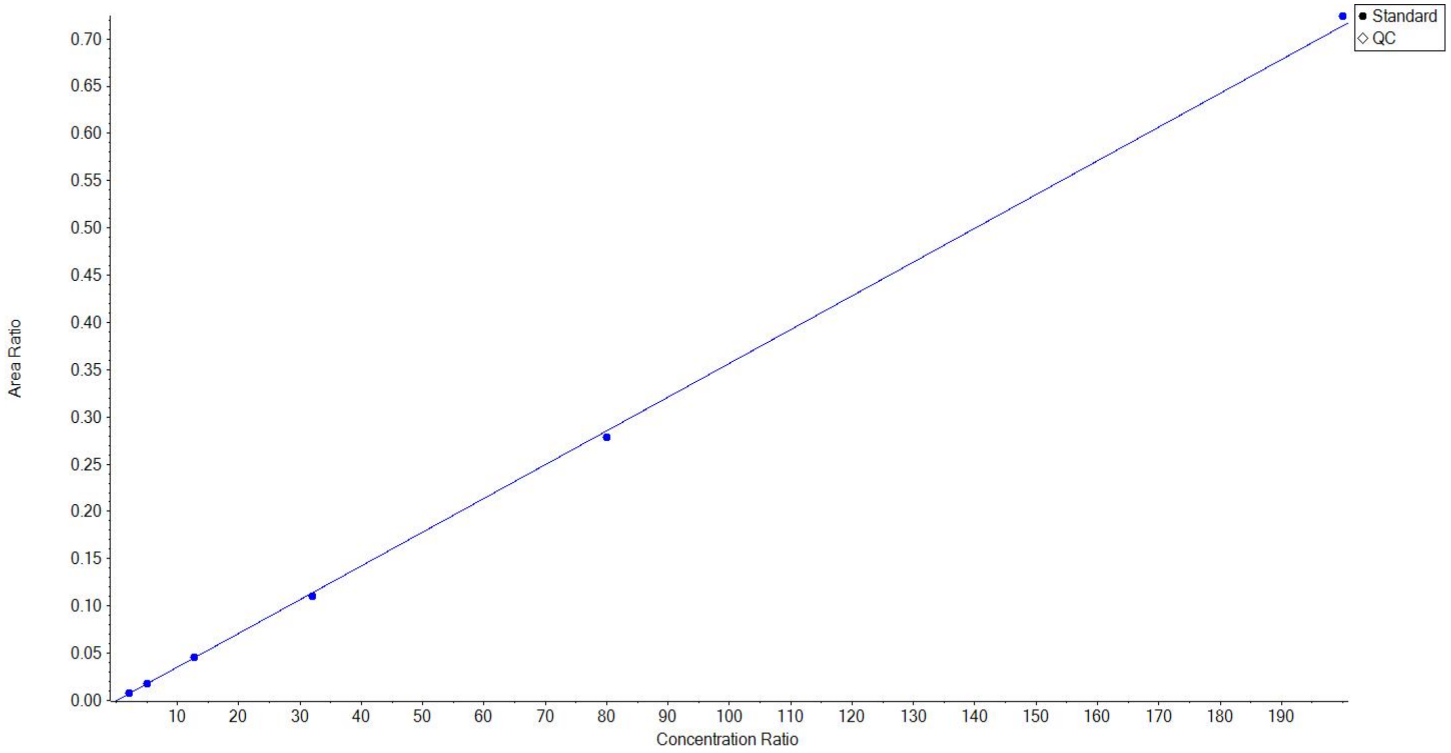
Standard
QC
Area Ratio
Concentration Ratio
0.00
0.05
0.10
0.15
0.20
0.25
0.30
0.35
0.40
0.45
0.50
0.55
0.60
0.65
0.70
10
20
30
40
50
60
70
80
90
100
110
120
130
140
150
160
170
180
190

Analyte Name: LM-flavones-03_1
Internal Standard: LM-flavones-IS02_1

**Data File**	flavones-STD-20230908.wiff	**Result Table**	DZLM2023082419-results-20230913-5500
**Acquisition Date**	9/8/2023 5:37:08 PM	**Algorithm Used**	MQ4
**Acquisition Method**	20230908-flavones-(mix130-T3)-15min.dam	**Instrument Name**	QTRAP 6500+ Low Mass
**Project**	N/A	**Processing Method**	20230412-flavones-(mix130-T3)-15min.qmethod

Regression Equation: y = 0.00170 x + -7.96587e-4 (r = 0.99936, r^2 = 0.99873) (weighting: 1 / x)

Expected Concentration	Number of Values	Mean Calculated Concentration (No data for Analyte Unit)	% Accuracy	Std. Deviation	%CV
0.01	0 of 1	N/A	N/A	N/A	N/A
0.02	0 of 1	N/A	N/A	N/A	N/A
0.05	0 of 1	N/A	N/A	N/A	N/A
0.13	0 of 1	N/A	N/A	N/A	N/A
0.33	0 of 1	N/A	N/A	N/A	N/A
0.82	0 of 1	N/A	N/A	N/A	N/A
2.05	1 of 1	2.403e0	117.2	N/A	N/A
5.12	1 of 1	4.972e0	97.1	N/A	N/A
12.80	1 of 1	1.161e1	90.7	N/A	N/A
32.00	1 of 1	3.019e1	94.3	N/A	N/A
80.00	1 of 1	7.896e1	98.7	N/A	N/A
200.00	1 of 1	2.038e2	101.9	N/A	N/A

Analyte Name: LM-flavones-03_1

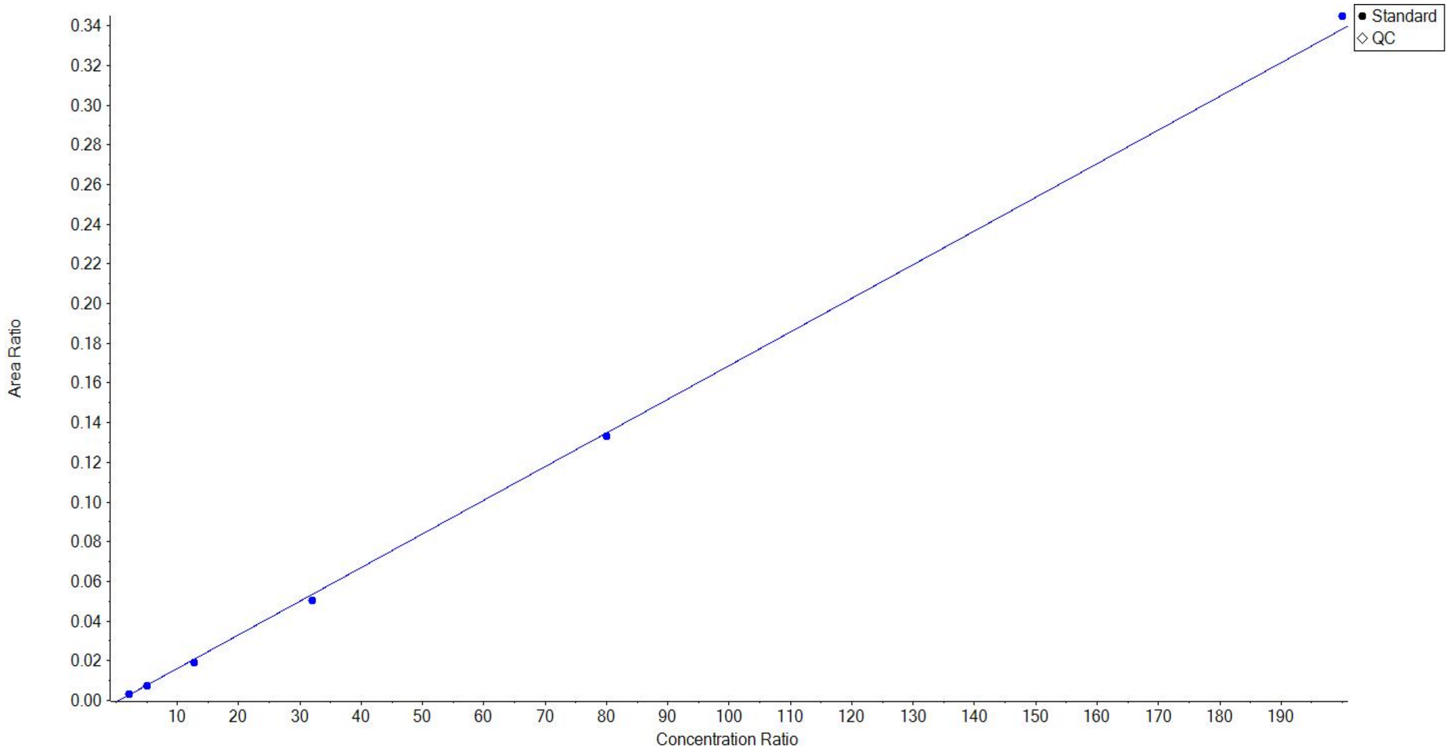
Standard
QC
Area Ratio
0.34
0.32
0.30
0.28
0.26
0.24
0.22
0.20
0.18
0.16
0.14
0.12
0.10
0.08
0.06
0.04
0.02
0.00
10
20
30
40
50
60
70
80
90
100
110
120
130
140
150
160
170
180
190
Concentration Ratio

Analyte Name: LM-flavones-04_1
Internal Standard: LM-flavones-IS02_1

**Data File**	flavones-STD-20230908.wiff	**Result Table**	DZLM2023082419-results-20230913-5500
**Acquisition Date**	9/8/2023 5:37:08 PM	**Algorithm Used**	MQ4
**Acquisition Method**	20230908-flavones-(mix130-T3)-15min.dam	**Instrument Name**	QTRAP 6500+ Low Mass
**Project**	N/A	**Processing Method**	20230412-flavones-(mix130-T3)-15min.qmethod

Regression Equation: y = 4.45508e-4 x + -8.37358e-4 (r = 0.99920, r^2 = 0.99840) (weighting: 1 / x)

Expected Concentration	Number of Values	Mean Calculated Concentration (No data for Analyte Unit)	% Accuracy	Std. Deviation	%CV
0.01	0 of 1	N/A	N/A	N/A	N/A
0.02	0 of 1	N/A	N/A	N/A	N/A
0.05	0 of 1	N/A	N/A	N/A	N/A
0.13	0 of 1	N/A	N/A	N/A	N/A
0.33	0 of 1	N/A	N/A	N/A	N/A
0.82	0 of 1	N/A	N/A	N/A	N/A
2.05	0 of 1	N/A	N/A	N/A	N/A
5.12	1 of 1	4.575e0	89.4	N/A	N/A
12.80	1 of 1	1.397e1	109.2	N/A	N/A
32.00	1 of 1	3.366e1	105.2	N/A	N/A
80.00	1 of 1	7.657e1	95.7	N/A	N/A
200.00	1 of 1	2.011e2	100.6	N/A	N/A

Analyte Name: LM-flavones-04_1

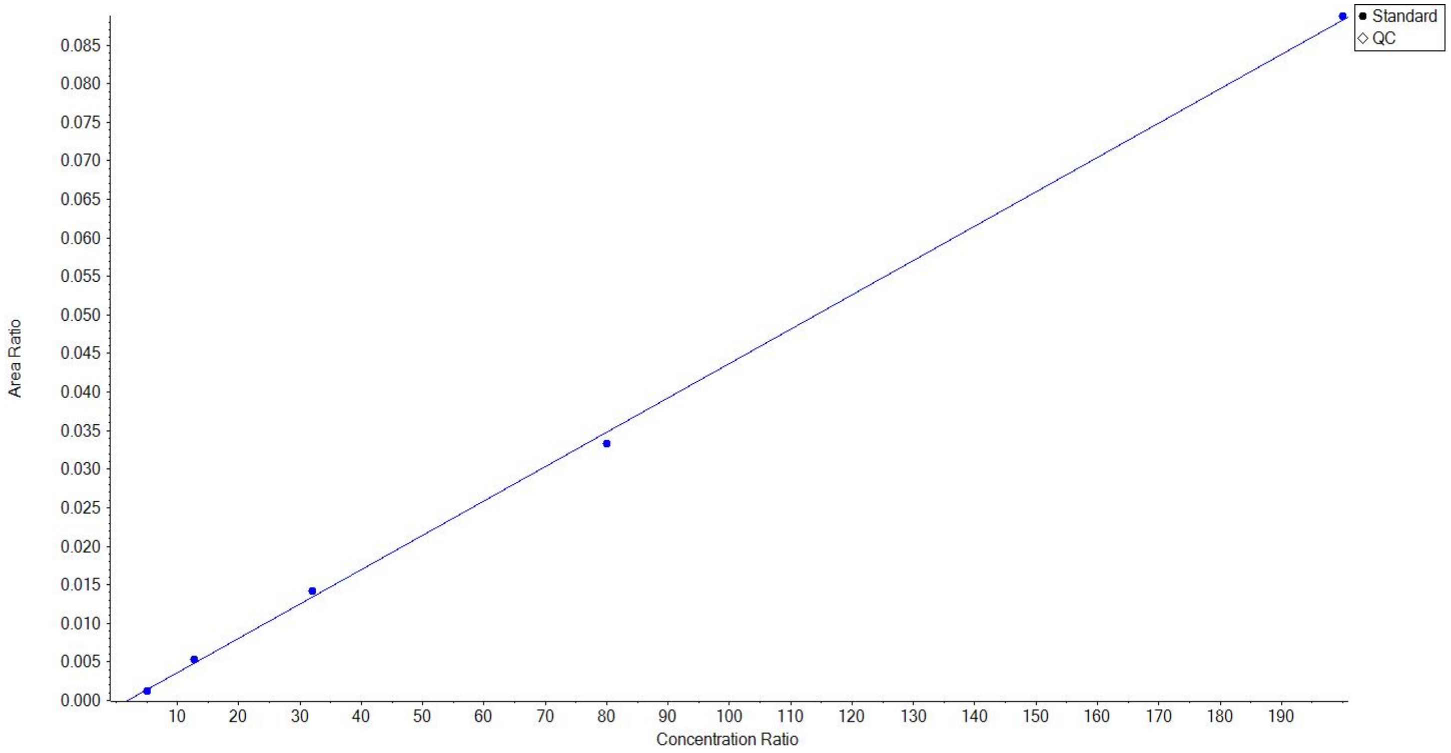
Standard
QC
Area Ratio
0.085
0.080
0.075
0.070
0.065
0.060
0.055
0.050
0.045
0.040
0.035
0.030
0.025
0.020
0.015
0.010
0.005
0.000
10 20 30 40 50 60 70 80 90 100 110 120 130 140 150 160 170 180 190
Concentration Ratio

Analyte Name: LM-flavones-07_1
Internal Standard: LM-flavones-IS02_1

**Data File**	flavones-STD-20230908.wiff	**Result Table**	DZLM2023082419-results-20230913-5500
**Acquisition Date**	9/8/2023 5:37:08 PM	**Algorithm Used**	MQ4
**Acquisition Method**	20230908-flavones-(mix130-T3)-15min.dam	**Instrument Name**	QTRAP 6500+ Low Mass
**Project**	N/A	**Processing Method**	20230412-flavones-(mix130-T3)-15min.qmethod

Regression Equation: y = 0.00671 x + -0.00104 (r = 0.99967, r² = 0.99934) (weighting: 1 / x)

Expected Concentration	Number of Values	Mean Calculated Concentration (No data for Analyte Unit)	% Accuracy	Std. Deviation	%CV
0.01	0 of 1	N/A	N/A	N/A	N/A
0.02	0 of 1	N/A	N/A	N/A	N/A
0.05	0 of 1	N/A	N/A	N/A	N/A
0.13	0 of 1	N/A	N/A	N/A	N/A
0.33	0 of 1	N/A	N/A	N/A	N/A
0.82	0 of 1	N/A	N/A	N/A	N/A
2.05	1 of 1	2.228e0	108.7	N/A	N/A
5.12	1 of 1	5.014e0	97.9	N/A	N/A
12.80	1 of 1	1.205e1	94.1	N/A	N/A
32.00	1 of 1	3.232e1	101.0	N/A	N/A
80.00	1 of 1	7.742e1	96.8	N/A	N/A
200.00	1 of 1	2.029e2	101.5	N/A	N/A

Analyte Name: LM-flavones-07_1

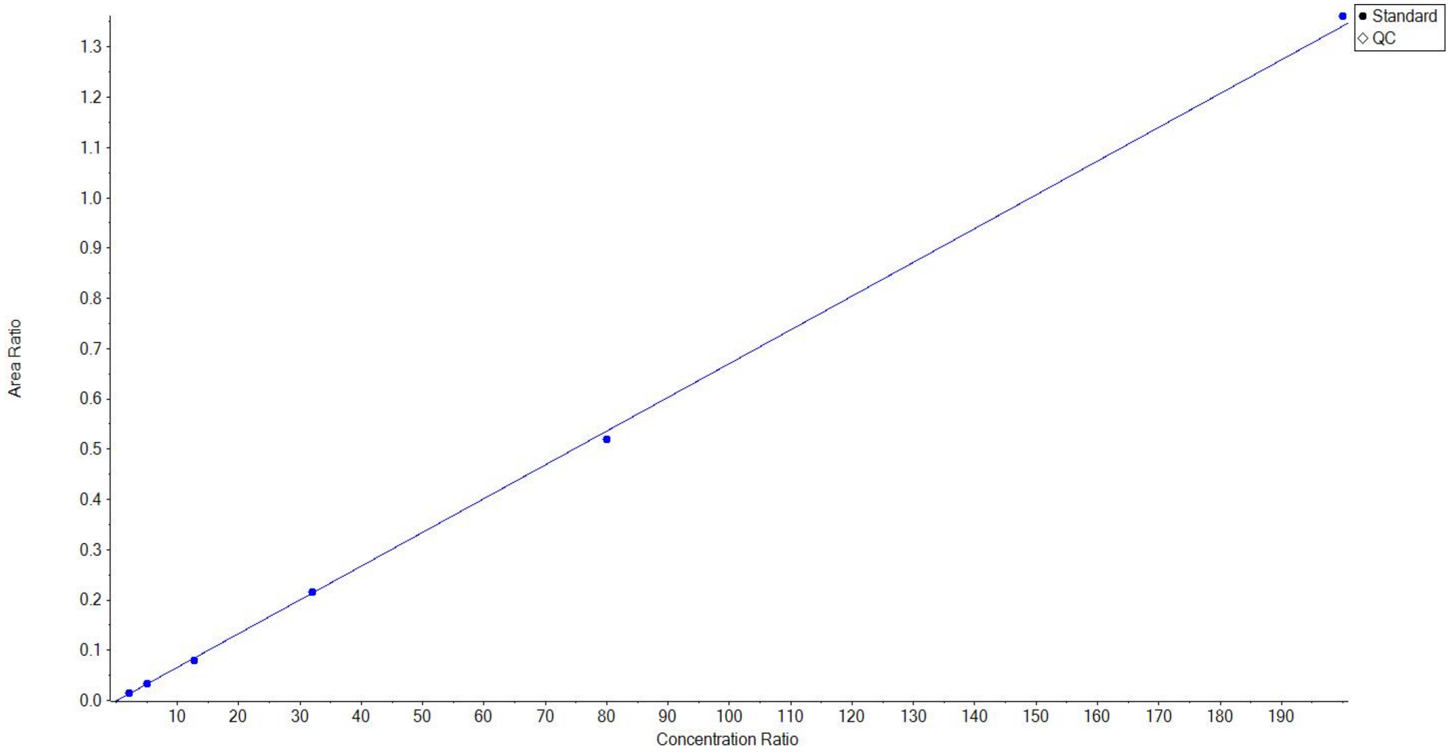
Standard
QC
Area Ratio
Concentration Ratio
0.0
0.1
0.2
0.3
0.4
0.5
0.6
0.7
0.8
0.9
1.0
1.1
1.2
1.3
10
20
30
40
50
60
70
80
90
100
110
120
130
140
150
160
170
180
190

Analyte Name: LM-flavones-08_1
Internal Standard: LM-flavones-IS02_1

**Data File**	flavones-STD-20230908.wiff	**Result Table**	DZLM2023082419-results-20230913-5500
**Acquisition Date**	9/8/2023 5:37:08 PM	**Algorithm Used**	MQ4
**Acquisition Method**	20230908-flavones-(mix130-T3)-15min.dam	**Instrument Name**	QTRAP 6500+ Low Mass
**Project**	N/A	**Processing Method**	20230412-flavones-(mix130-T3)-15min.qmethod

Regression Equation: y = 0.00176 x + -0.00256 (r = 0.99993, r^2 = 0.99986) (weighting: 1 / x)

Expected Concentration	Number of Values	Mean Calculated Concentration (No data for Analyte Unit)	% Accuracy	Std. Deviation	%CV
0.01	0 of 1	N/A	N/A	N/A	N/A
0.02	0 of 1	N/A	N/A	N/A	N/A
0.05	0 of 1	N/A	N/A	N/A	N/A
0.13	0 of 1	N/A	N/A	N/A	N/A
0.33	0 of 1	N/A	N/A	N/A	N/A
0.82	0 of 1	N/A	N/A	N/A	N/A
2.05	0 of 1	N/A	N/A	N/A	N/A
5.12	1 of 1	5.144e0	100.5	N/A	N/A
12.80	1 of 1	1.304e1	101.9	N/A	N/A
32.00	1 of 1	3.147e1	98.3	N/A	N/A
80.00	1 of 1	7.895e1	98.7	N/A	N/A
200.00	1 of 1	2.013e2	100.7	N/A	N/A

Analyte Name: LM-flavones-08_1

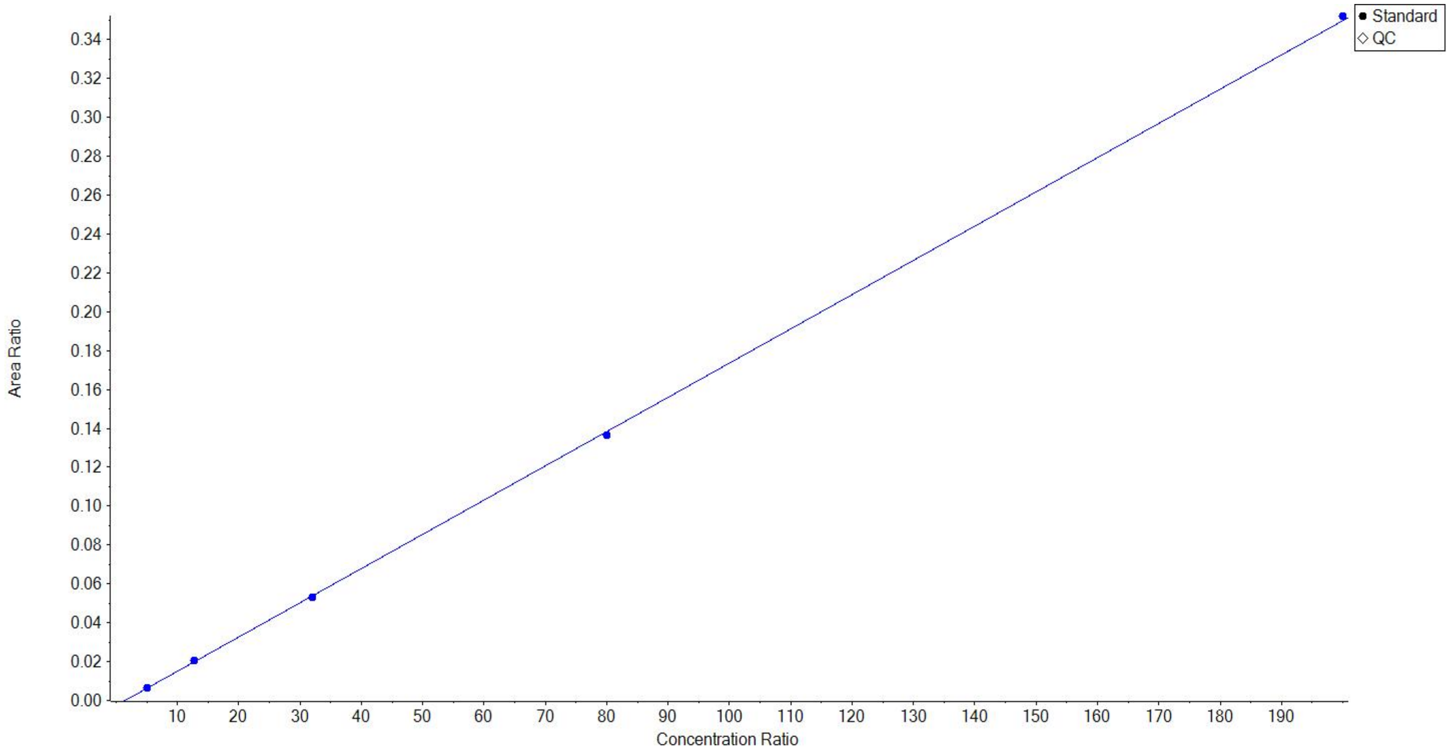
Standard
QC
Area Ratio
0.34
0.32
0.30
0.28
0.26
0.24
0.22
0.20
0.18
0.16
0.14
0.12
0.10
0.08
0.06
0.04
0.02
0.00
10
20
30
40
50
60
70
80
90
100
110
120
130
140
150
160
170
180
190
Concentration Ratio

Analyte Name: LM-flavones-09_1
Internal Standard: LM-flavones-IS02_1

**Data File**	flavones-STD-20230908.wiff	**Result Table**	DZLM2023082419-results-20230913-5500
**Acquisition Date**	9/8/2023 5:37:08 PM	**Algorithm Used**	MQ4
**Acquisition Method**	20230908-flavones-(mix130-T3)-15min.dam	**Instrument Name**	QTRAP 6500+ Low Mass
**Project**	N/A	**Processing Method**	20230412-flavones-(mix130-T3)-15min.qmethod

Regression Equation: y = 0.00239 x + -0.00104 (r = 0.99972, r² = 0.99944) (weighting: 1 / x)

Expected Concentration	Number of Values	Mean Calculated Concentration (No data for Analyte Unit)	% Accuracy	Std. Deviation	%CV
0.01	0 of 1	N/A	N/A	N/A	N/A
0.02	0 of 1	N/A	N/A	N/A	N/A
0.05	0 of 1	N/A	N/A	N/A	N/A
0.13	0 of 1	N/A	N/A	N/A	N/A
0.33	0 of 1	N/A	N/A	N/A	N/A
0.82	0 of 1	N/A	N/A	N/A	N/A
2.05	1 of 1	2.282e0	111.3	N/A	N/A
5.12	1 of 1	4.791e0	93.6	N/A	N/A
12.80	1 of 1	1.270e1	99.2	N/A	N/A
32.00	1 of 1	3.023e1	94.5	N/A	N/A
80.00	1 of 1	8.055e1	100.7	N/A	N/A
200.00	1 of 1	2.014e2	100.7	N/A	N/A

Analyte Name: LM-flavones-09_1

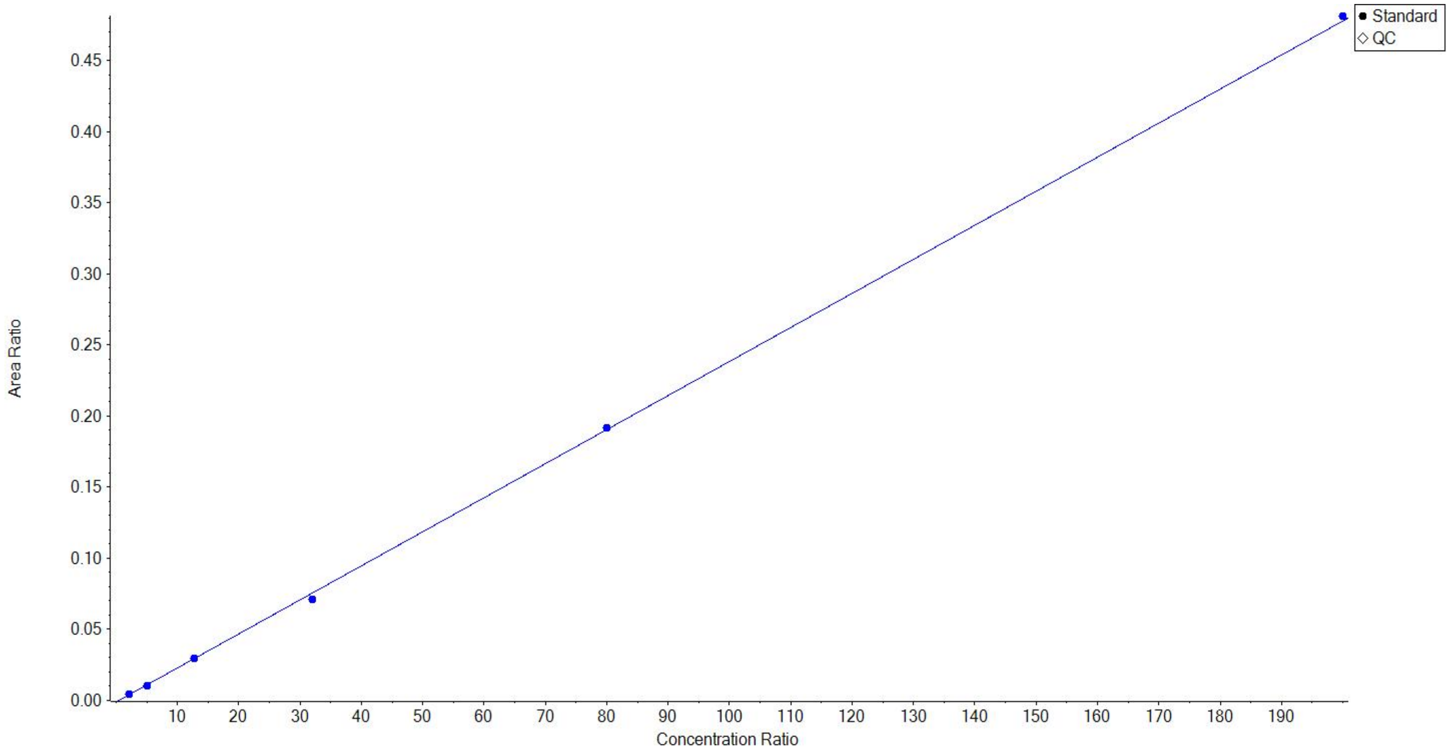
Standard
QC
Area Ratio
0.00
0.05
0.10
0.15
0.20
0.25
0.30
0.35
0.40
0.45
10
20
30
40
50
60
70
80
90
100
110
120
130
140
150
160
170
180
190
Concentration Ratio

Analyte Name: LM-flavones-10
Internal Standard: LM-flavones-IS02_1

**Data File**	flavones-STD-20230908.wiff	**Result Table**	DZLM2023082419-results-20230913-5500
**Acquisition Date**	9/8/2023 5:37:08 PM	**Algorithm Used**	MQ4
**Acquisition Method**	20230908-flavones-(mix130-T3)-15min.dam	**Instrument Name**	QTRAP 6500+ Low Mass
**Project**	N/A	**Processing Method**	20230412-flavones-(mix130-T3)-15min.qmethod

Regression Equation: y = 0.00174 x + 2.13694e-4 (r = 0.99943, r² = 0.99887) (weighting: 1 / x)

Expected Concentration	Number of Values	Mean Calculated Concentration (No data for Analyte Unit)	% Accuracy	Std. Deviation	%CV
0.01	0 of 1	N/A	N/A	N/A	N/A
0.02	0 of 1	N/A	N/A	N/A	N/A
0.05	0 of 1	N/A	N/A	N/A	N/A
0.13	0 of 1	N/A	N/A	N/A	N/A
0.33	0 of 1	N/A	N/A	N/A	N/A
0.82	0 of 1	N/A	N/A	N/A	N/A
2.05	1 of 1	2.126e0	103.7	N/A	N/A
5.12	1 of 1	5.402e0	105.5	N/A	N/A
12.80	1 of 1	1.250e1	97.7	N/A	N/A
32.00	1 of 1	3.017e1	94.3	N/A	N/A
80.00	1 of 1	7.726e1	96.6	N/A	N/A
200.00	1 of 1	2.045e2	102.3	N/A	N/A

Analyte Name: LM-flavones-10

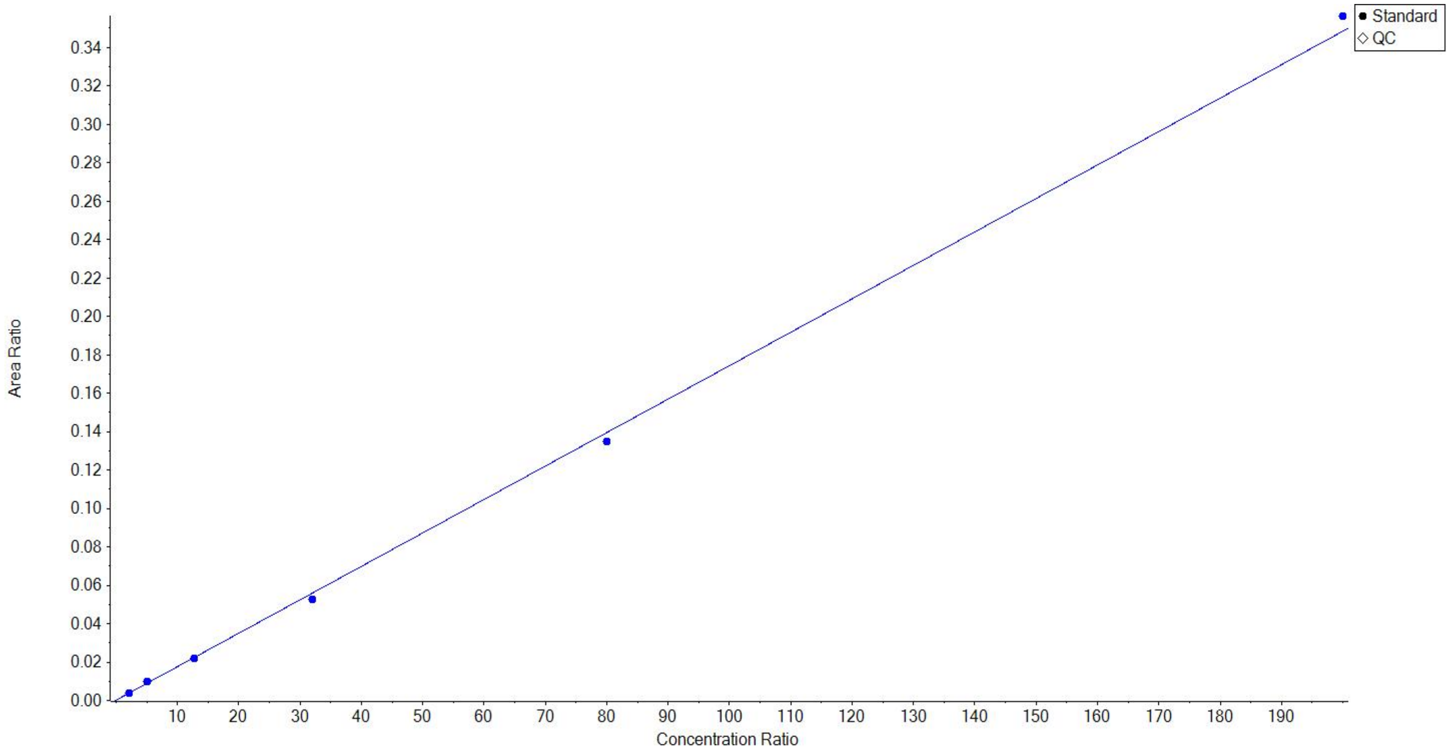
Standard
QC
Area Ratio
Concentration Ratio
0.00
0.02
0.04
0.06
0.08
0.10
0.12
0.14
0.16
0.18
0.20
0.22
0.24
0.26
0.28
0.30
0.32
0.34
10
20
30
40
50
60
70
80
90
100
110
120
130
140
150
160
170
180
190

Analyte Name: LM-flavones-11_1
Internal Standard: LM-flavones-IS02_1

**Data File**	flavones-STD-20230908.wiff	**Result Table**	DZLM2023082419-results-20230913-5500
**Acquisition Date**	9/8/2023 5:37:08 PM	**Algorithm Used**	MQ4
**Acquisition Method**	20230908-flavones-(mix130-T3)-15min.dam	**Instrument Name**	QTRAP 6500+ Low Mass
**Project**	N/A	**Processing Method**	20230412-flavones-(mix130-T3)-15min.qmethod

Regression Equation: y = 0.00799 x + 0.00132 (r = 0.99914, r² = 0.99827) (weighting: 1 / x)

Expected Concentration	Number of Values	Mean Calculated Concentration (No data for Analyte Unit)	% Accuracy	Std. Deviation	%CV
0.01	0 of 1	N/A	N/A	N/A	N/A
0.02	0 of 1	N/A	N/A	N/A	N/A
0.05	0 of 1	N/A	N/A	N/A	N/A
0.13	0 of 1	N/A	N/A	N/A	N/A
0.33	0 of 1	N/A	N/A	N/A	N/A
0.82	0 of 1	N/A	N/A	N/A	N/A
2.05	1 of 1	1.967e0	96.0	N/A	N/A
5.12	1 of 1	4.959e0	96.9	N/A	N/A
12.80	1 of 1	1.244e1	97.2	N/A	N/A
32.00	1 of 1	3.492e1	109.1	N/A	N/A
80.00	1 of 1	8.265e1	103.3	N/A	N/A
200.00	1 of 1	1.950e2	97.5	N/A	N/A

Analyte Name: LM-flavones-11_1

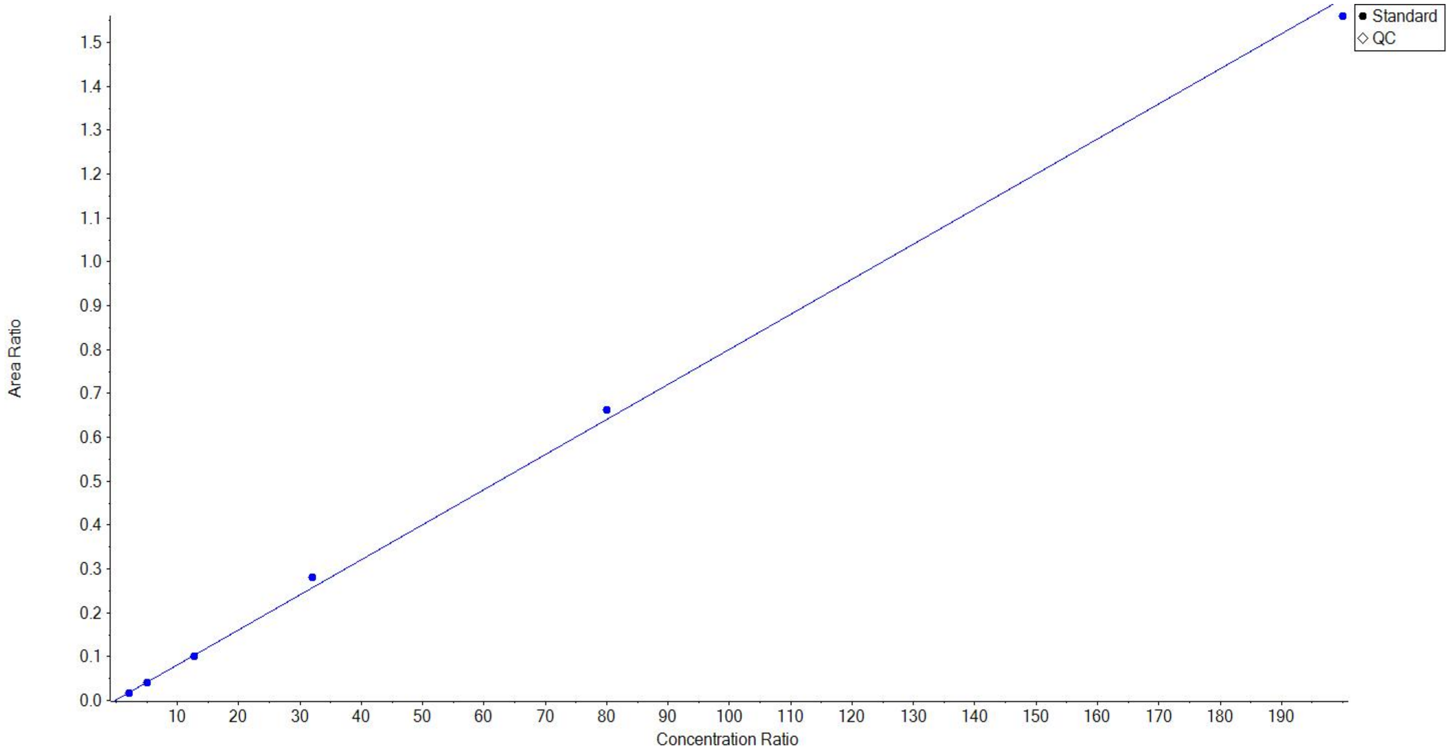
Area Ratio
Concentration Ratio
Standard
QC
0.0
0.1
0.2
0.3
0.4
0.5
0.6
0.7
0.8
0.9
1.0
1.1
1.2
1.3
1.4
1.5
10
20
30
40
50
60
70
80
90
100
110
120
130
140
150
160
170
180
190

Analyte Name: LM-flavones-12_1
Internal Standard: LM-flavones-IS02_1

**Data File**	flavones-STD-20230908.wiff	**Result Table**	DZLM2023082419-results-20230913-5500
**Acquisition Date**	9/8/2023 5:37:08 PM	**Algorithm Used**	MQ4
**Acquisition Method**	20230908-flavones-(mix130-T3)-15min.dam	**Instrument Name**	QTRAP 6500+ Low Mass
**Project**	N/A	**Processing Method**	20230412-flavones-(mix130-T3)-15min.qmethod

Regression Equation: y = 0.00142 x + -2.27780e-4 (r = 0.99975, r^2 = 0.99950) (weighting: 1 / x)

Expected Concentration	Number of Values	Mean Calculated Concentration (No data for Analyte Unit)	% Accuracy	Std. Deviation	%CV
0.01	0 of 1	N/A	N/A	N/A	N/A
0.02	0 of 1	N/A	N/A	N/A	N/A
0.05	0 of 1	N/A	N/A	N/A	N/A
0.13	0 of 1	N/A	N/A	N/A	N/A
0.33	0 of 1	N/A	N/A	N/A	N/A
0.82	0 of 1	N/A	N/A	N/A	N/A
2.05	1 of 1	2.144e0	104.6	N/A	N/A
5.12	1 of 1	4.729e0	92.4	N/A	N/A
12.80	1 of 1	1.271e1	99.3	N/A	N/A
32.00	1 of 1	3.273e1	102.3	N/A	N/A
80.00	1 of 1	8.219e1	102.7	N/A	N/A
200.00	1 of 1	1.975e2	98.7	N/A	N/A

Analyte Name: LM-flavones-12_1

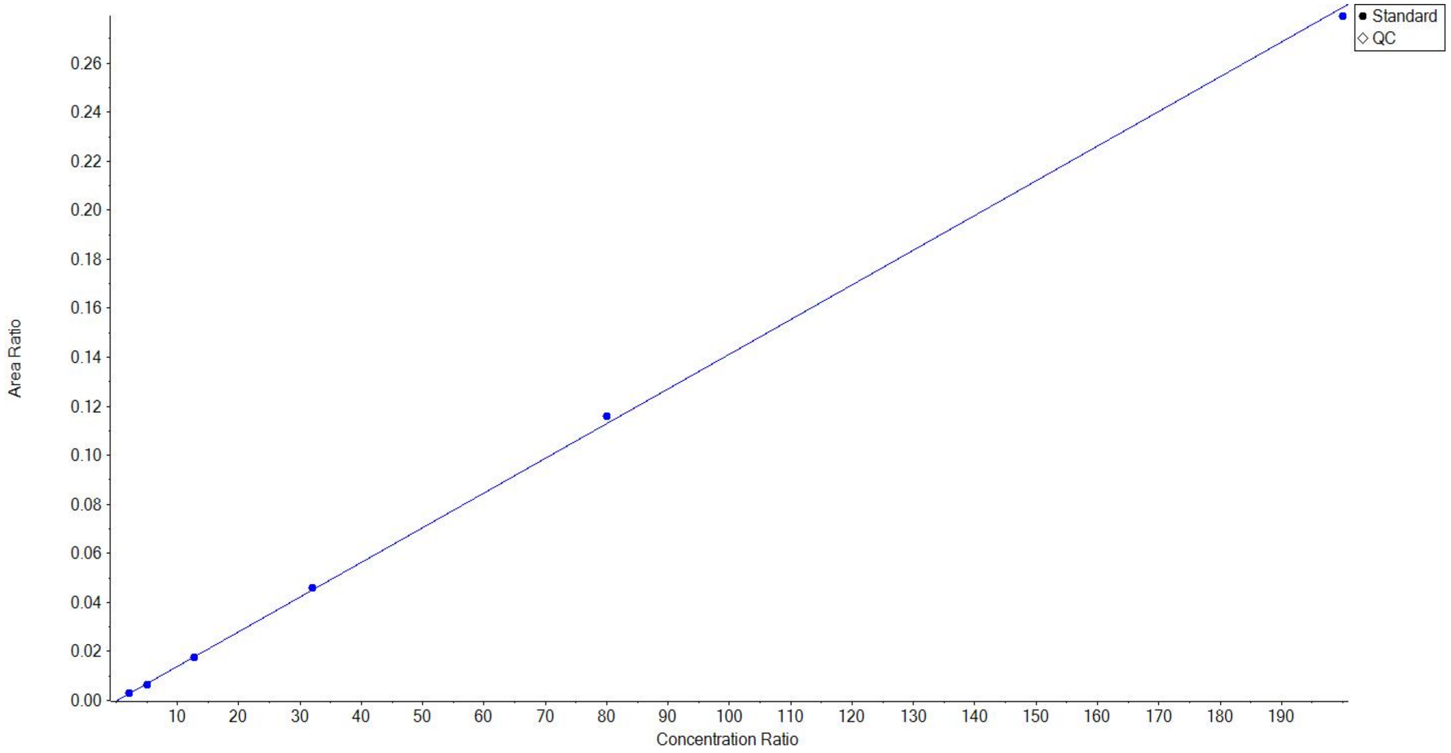
Standard
QC
Area Ratio
0.00
0.02
0.04
0.06
0.08
0.10
0.12
0.14
0.16
0.18
0.20
0.22
0.24
0.26
10
20
30
40
50
60
70
80
90
100
110
120
130
140
150
160
170
180
190
Concentration Ratio

Analyte Name: LM-flavones-13_1
Internal Standard: LM-flavones-IS02_1

**Data File**	flavones-STD-20230908.wiff	**Result Table**	DZLM2023082419-results-20230913-5500
**Acquisition Date**	9/8/2023 5:37:08 PM	**Algorithm Used**	MQ4
**Acquisition Method**	20230908-flavones-(mix130-T3)-15min.dam	**Instrument Name**	QTRAP 6500+ Low Mass
**Project**	N/A	**Processing Method**	20230412-flavones-(mix130-T3)-15min.qmethod

Regression Equation: y = 8.49148e-4 x + -3.96430e-4 (r = 0.99940, r^2 = 0.99881) (weighting: 1 / x)

Expected Concentration	Number of Values	Mean Calculated Concentration (No data for Analyte Unit)	% Accuracy	Std. Deviation	%CV
0.01	0 of 1	N/A	N/A	N/A	N/A
0.02	0 of 1	N/A	N/A	N/A	N/A
0.05	0 of 1	N/A	N/A	N/A	N/A
0.13	0 of 1	N/A	N/A	N/A	N/A
0.33	0 of 1	N/A	N/A	N/A	N/A
0.82	0 of 1	N/A	N/A	N/A	N/A
2.05	1 of 1	2.155e0	105.1	N/A	N/A
5.12	1 of 1	5.516e0	107.7	N/A	N/A
12.80	1 of 1	1.197e1	93.5	N/A	N/A
32.00	1 of 1	3.005e1	93.9	N/A	N/A
80.00	1 of 1	7.807e1	97.6	N/A	N/A
200.00	1 of 1	2.042e2	102.1	N/A	N/A

Analyte Name: LM-flavones-13_1

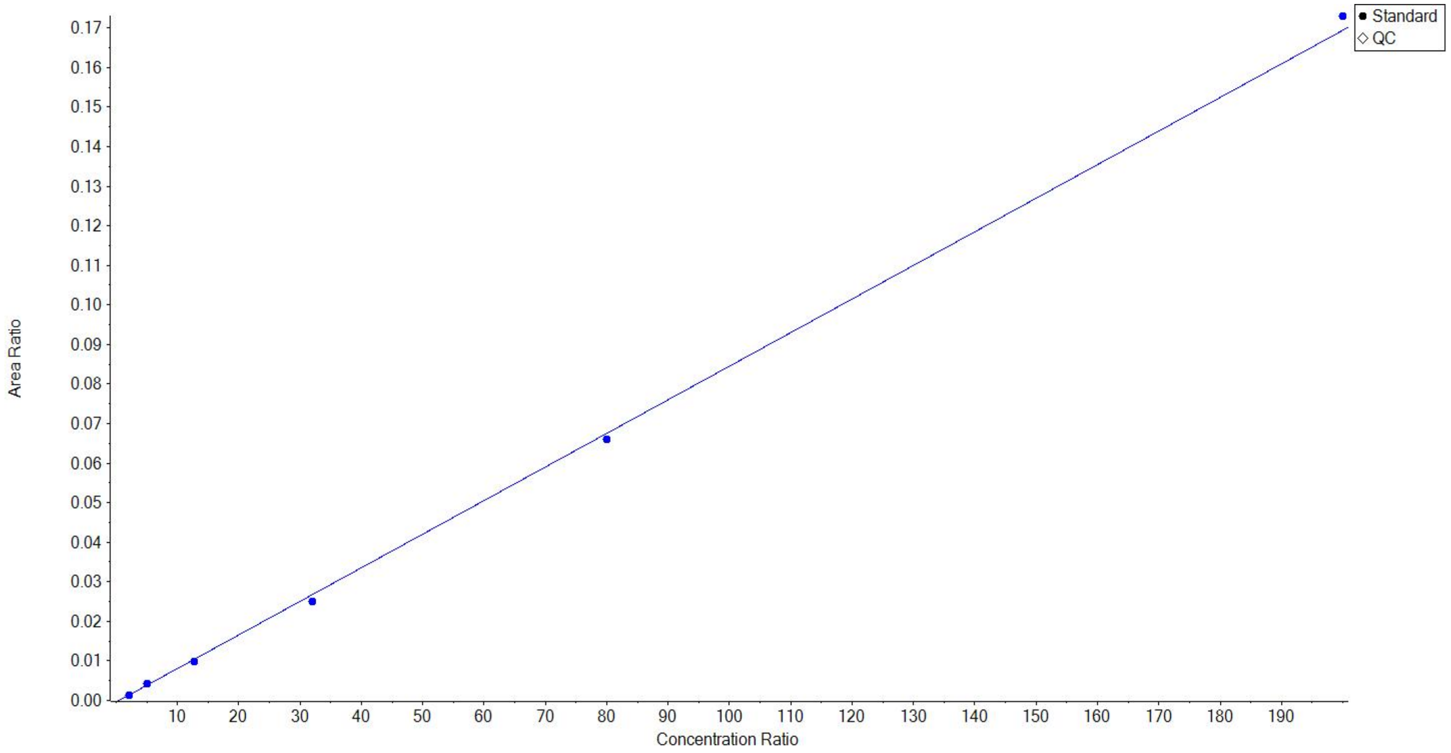
Standard
QC
Area Ratio
Concentration Ratio
0.17
0.16
0.15
0.14
0.13
0.12
0.11
0.10
0.09
0.08
0.07
0.06
0.05
0.04
0.03
0.02
0.01
0.00
10
20
30
40
50
60
70
80
90
100
110
120
130
140
150
160
170
180
190

Analyte Name: LM-flavones-14_1
Internal Standard: LM-flavones-IS02_1

**Data File**	flavones-STD-20230908.wiff	**Result Table**	DZLM2023082419-results-20230913-5500
**Acquisition Date**	9/8/2023 5:37:08 PM	**Algorithm Used**	MQ4
**Acquisition Method**	20230908-flavones-(mix130-T3)-15min.dam	**Instrument Name**	QTRAP 6500+ Low Mass
**Project**	N/A	**Processing Method**	20230412-flavones-(mix130-T3)-15min.qmethod

Regression Equation: y = 0.00355 x + 4.69449e-4 (r = 0.99966, r² = 0.99931) (weighting: 1 / x)

Expected Concentration	Number of Values	Mean Calculated Concentration (No data for Analyte Unit)	% Accuracy	Std. Deviation	%CV
0.01	0 of 1	N/A	N/A	N/A	N/A
0.02	0 of 1	N/A	N/A	N/A	N/A
0.05	0 of 1	N/A	N/A	N/A	N/A
0.13	0 of 1	N/A	N/A	N/A	N/A
0.33	0 of 1	N/A	N/A	N/A	N/A
0.82	0 of 1	N/A	N/A	N/A	N/A
2.05	1 of 1	1.872e0	91.3	N/A	N/A
5.12	1 of 1	5.508e0	107.6	N/A	N/A
12.80	1 of 1	1.298e1	101.4	N/A	N/A
32.00	1 of 1	3.283e1	102.6	N/A	N/A
80.00	1 of 1	7.700e1	96.3	N/A	N/A
200.00	1 of 1	2.018e2	100.9	N/A	N/A

Analyte Name: LM-flavones-14_1

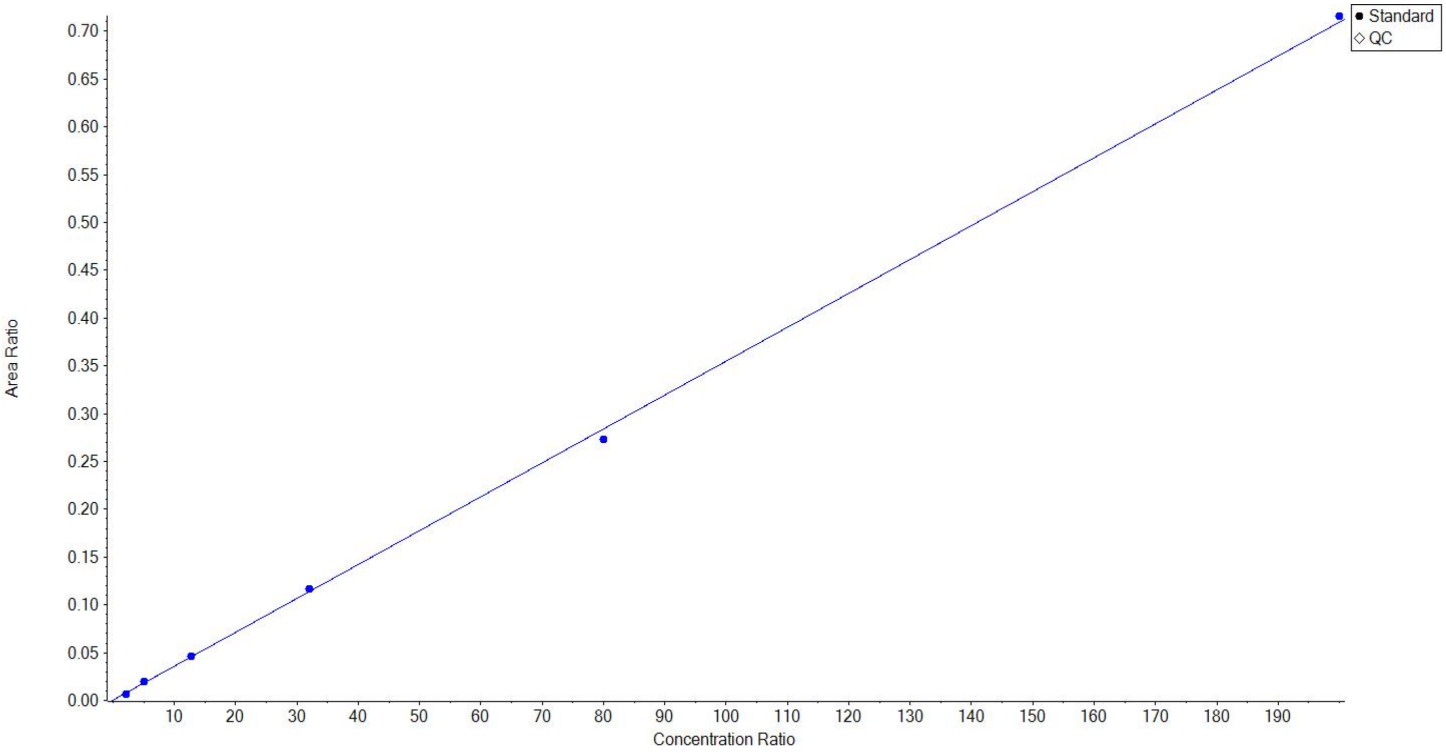
Standard
QC
Area Ratio
Concentration Ratio
0.70
0.65
0.60
0.55
0.50
0.45
0.40
0.35
0.30
0.25
0.20
0.15
0.10
0.05
0.00
10
20
30
40
50
60
70
80
90
100
110
120
130
140
150
160
170
180
190

Analyte Name: LM-flavones-15_1
Internal Standard: LM-flavones-IS02_1

**Data File**	flavones-STD-20230908.wiff	**Result Table**	DZLM2023082419-results-20230913-5500
**Acquisition Date**	9/8/2023 5:37:08 PM	**Algorithm Used**	MQ4
**Acquisition Method**	20230908-flavones-(mix130-T3)-15min.dam	**Instrument Name**	QTRAP 6500+ Low Mass
**Project**	N/A	**Processing Method**	20230412-flavones-(mix130-T3)-15min.qmethod

Regression Equation: y = 0.00215 x + -7.31626e-4 (r = 0.99975, r^2 = 0.99949) (weighting: 1 / x)

Expected Concentration	Number of Values	Mean Calculated Concentration (No data for Analyte Unit)	% Accuracy	Std. Deviation	%CV
0.01	0 of 1	N/A	N/A	N/A	N/A
0.02	0 of 1	N/A	N/A	N/A	N/A
0.05	0 of 1	N/A	N/A	N/A	N/A
0.13	0 of 1	N/A	N/A	N/A	N/A
0.33	0 of 1	N/A	N/A	N/A	N/A
0.82	0 of 1	N/A	N/A	N/A	N/A
2.05	1 of 1	2.167e0	105.7	N/A	N/A
5.12	1 of 1	4.983e0	97.3	N/A	N/A
12.80	1 of 1	1.306e1	102.0	N/A	N/A
32.00	1 of 1	3.053e1	95.4	N/A	N/A
80.00	1 of 1	7.855e1	98.2	N/A	N/A
200.00	1 of 1	2.027e2	101.3	N/A	N/A

Analyte Name: LM-flavones-15_1

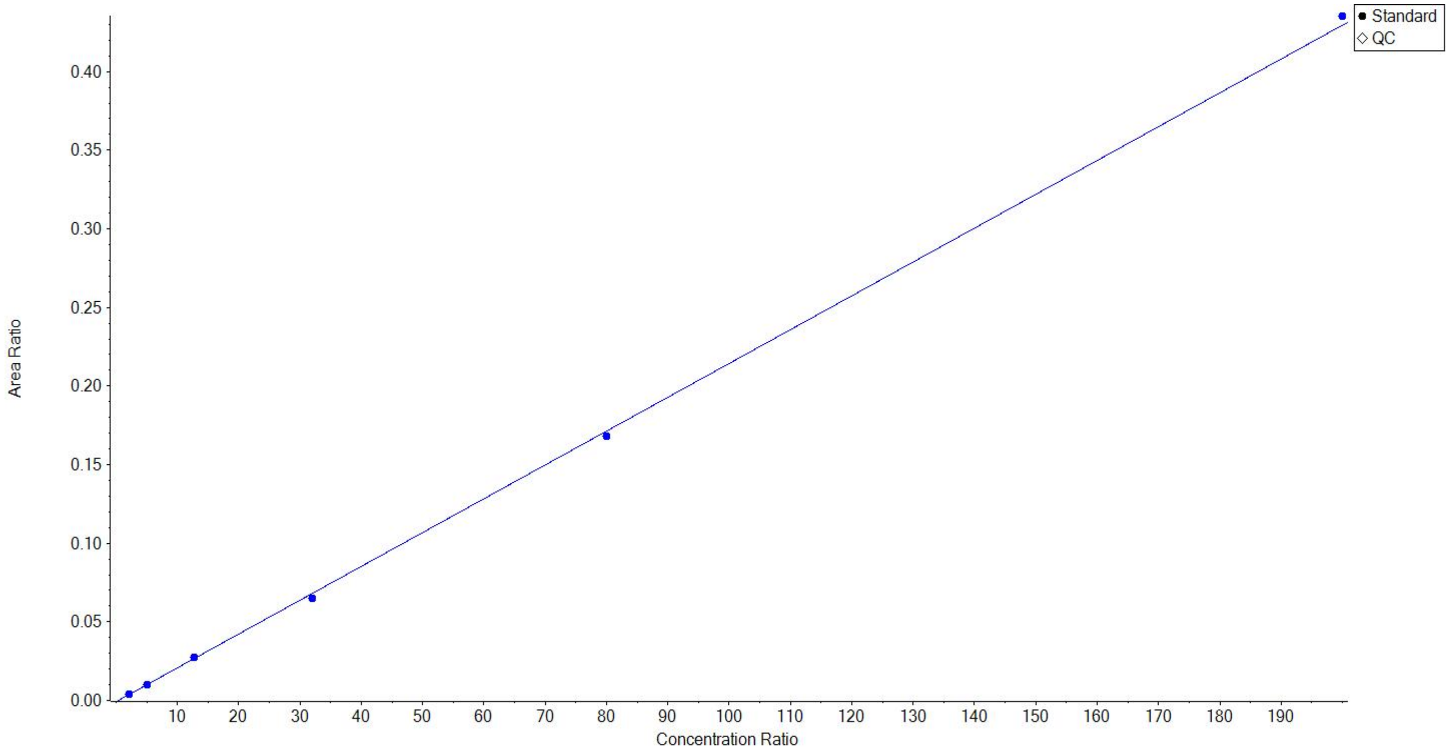
Standard
QC
Area Ratio
0.00
0.05
0.10
0.15
0.20
0.25
0.30
0.35
0.40
10
20
30
40
50
60
70
80
90
100
110
120
130
140
150
160
170
180
190
Concentration Ratio

Created with SCIEX OS 1.6.1
Printed:9/18/2023 5:27:01 PM

Analyte Name: LM-flavones-18_1
Internal Standard: LM-flavones-IS02_1

**Data File**	flavones-STD-20230908.wiff	**Result Table**	DZLM2023082419-results-20230913-5500
**Acquisition Date**	9/8/2023 5:37:08 PM	**Algorithm Used**	MQ4
**Acquisition Method**	20230908-flavones-(mix130-T3)-15min.dam	**Instrument Name**	QTRAP 6500+ Low Mass
**Project**	N/A	**Processing Method**	20230412-flavones-(mix130-T3)-15min.qmethod

Regression Equation: $y = 0.02825\ x + 0.00281\ (r = 0.99990,\ r^2 = 0.99980)$ (weighting: 1 / x)

Expected Concentration	Number of Values	Mean Calculated Concentration (No data for Analyte Unit)	% Accuracy	Std. Deviation	%CV
0.01	0 of 1	N/A	N/A	N/A	N/A
0.02	0 of 1	N/A	N/A	N/A	N/A
0.05	0 of 1	N/A	N/A	N/A	N/A
0.13	0 of 1	N/A	N/A	N/A	N/A
0.33	0 of 1	N/A	N/A	N/A	N/A
0.82	0 of 1	N/A	N/A	N/A	N/A
2.05	1 of 1	2.048e0	99.9	N/A	N/A
5.12	1 of 1	4.996e0	97.6	N/A	N/A
12.80	1 of 1	1.295e1	101.2	N/A	N/A
32.00	1 of 1	3.293e1	102.9	N/A	N/A
80.00	1 of 1	7.857e1	98.2	N/A	N/A
200.00	1 of 1	2.005e2	100.2	N/A	N/A

Analyte Name: LM-flavones-18_1

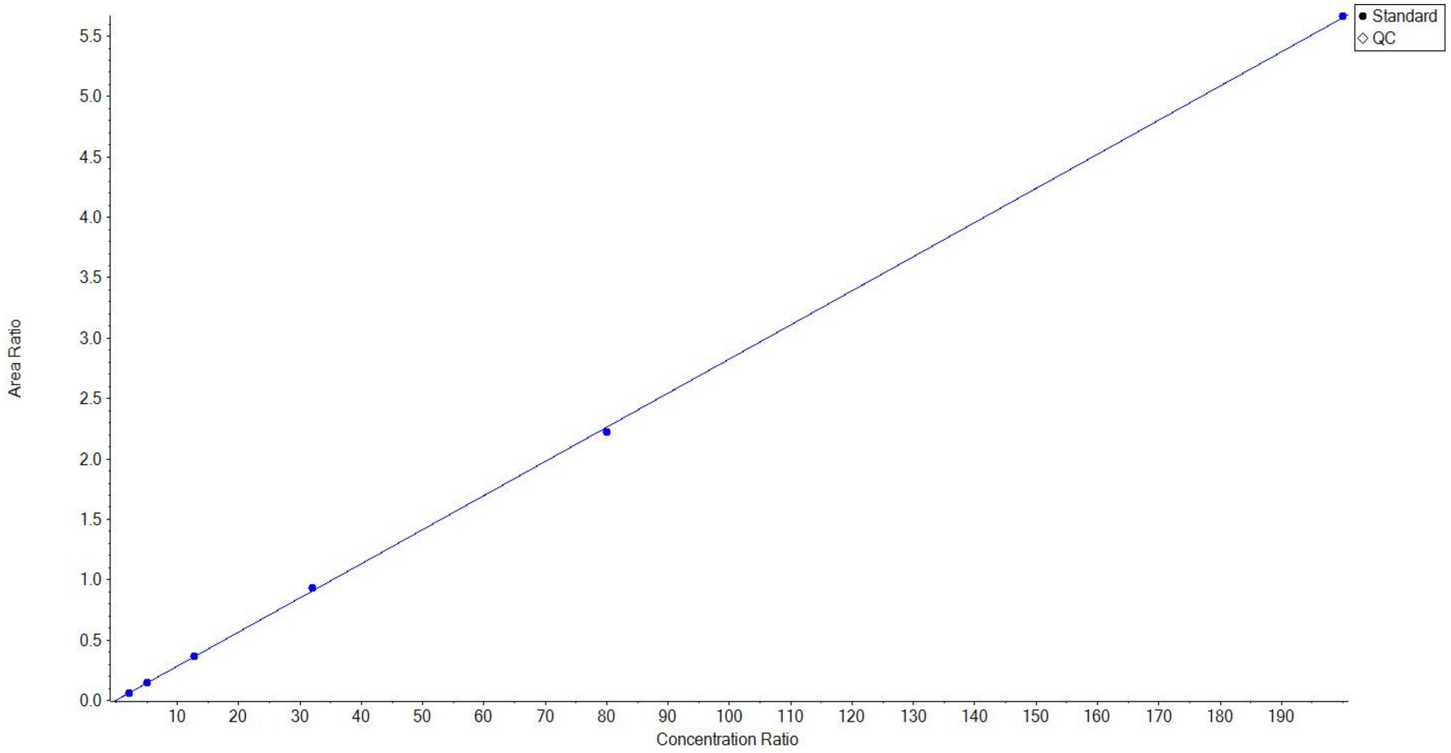
Standard
QC
Area Ratio
Concentration Ratio
0.0
0.5
1.0
1.5
2.0
2.5
3.0
3.5
4.0
4.5
5.0
5.5
10
20
30
40
50
60
70
80
90
100
110
120
130
140
150
160
170
180
190

Analyte Name: LM-flavones-19
Internal Standard: LM-flavones-IS02_1

**Data File**	flavones-STD-20230908.wiff	**Result Table**	DZLM2023082419-results-20230913-5500
**Acquisition Date**	9/8/2023 5:37:08 PM	**Algorithm Used**	MQ4
**Acquisition Method**	20230908-flavones-(mix130-T3)-15min.dam	**Instrument Name**	QTRAP 6500+ Low Mass
**Project**	N/A	**Processing Method**	20230412-flavones-(mix130-T3)-15min.qmethod

Regression Equation: y = 0.01016 x + -5.74373e-4 (r = 0.99949, r^2 = 0.99897) (weighting: 1 / x)

Expected Concentration	Number of Values	Mean Calculated Concentration (No data for Analyte Unit)	% Accuracy	Std. Deviation	%CV
0.01	0 of 1	N/A	N/A	N/A	N/A
0.02	0 of 1	N/A	N/A	N/A	N/A
0.05	0 of 1	N/A	N/A	N/A	N/A
0.13	0 of 1	N/A	N/A	N/A	N/A
0.33	0 of 1	N/A	N/A	N/A	N/A
0.82	0 of 1	N/A	N/A	N/A	N/A
2.05	1 of 1	2.238e0	109.2	N/A	N/A
5.12	1 of 1	5.050e0	98.6	N/A	N/A
12.80	1 of 1	1.199e1	93.7	N/A	N/A
32.00	1 of 1	3.230e1	100.9	N/A	N/A
80.00	1 of 1	7.654e1	95.7	N/A	N/A
200.00	1 of 1	2.039e2	101.9	N/A	N/A

Analyte Name: LM-flavones-19

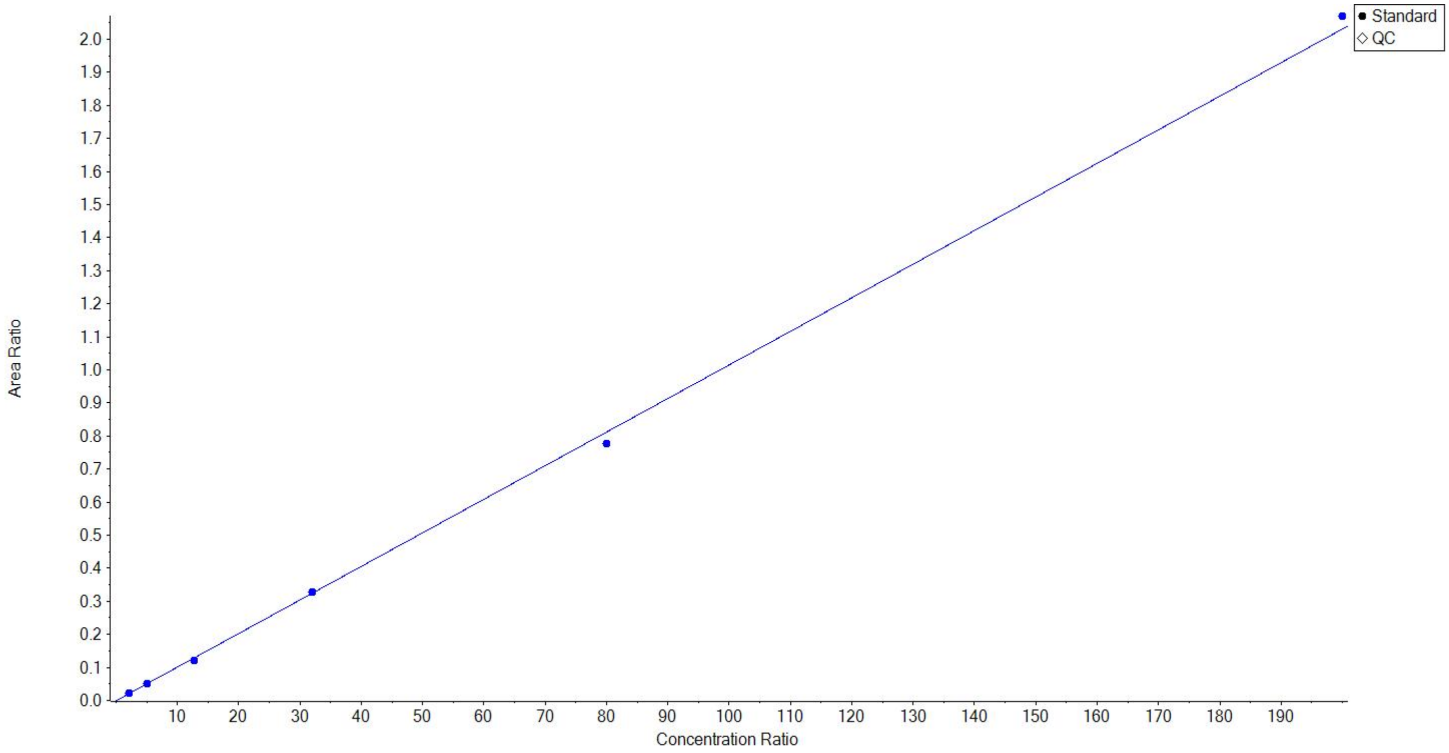
Standard
QC
Area Ratio
Concentration Ratio
2.0
1.9
1.8
1.7
1.6
1.5
1.4
1.3
1.2
1.1
1.0
0.9
0.8
0.7
0.6
0.5
0.4
0.3
0.2
0.1
0.0
10 20 30 40 50 60 70 80 90 100 110 120 130 140 150 160 170 180 190

Analyte Name: LM-flavones-20_1
Internal Standard: LM-flavones-IS02_1

**Data File**	flavones-STD-20230908.wiff	**Result Table**	DZLM2023082419-results-20230913-5500
**Acquisition Date**	9/8/2023 5:37:08 PM	**Algorithm Used**	MQ4
**Acquisition Method**	20230908-flavones-(mix130-T3)-15min.dam	**Instrument Name**	QTRAP 6500+ Low Mass
**Project**	N/A	**Processing Method**	20230412-flavones-(mix130-T3)-15min.qmethod

Regression Equation: y = 0.01422 x + -6.76439e-4 (r = 0.99997, r^2 = 0.99994) (weighting: 1 / x)

Expected Concentration	Number of Values	Mean Calculated Concentration (No data for Analyte Unit)	% Accuracy	Std. Deviation	%CV
0.01	0 of 1	N/A	N/A	N/A	N/A
0.02	0 of 1	N/A	N/A	N/A	N/A
0.05	0 of 1	N/A	N/A	N/A	N/A
0.13	0 of 1	N/A	N/A	N/A	N/A
0.33	0 of 1	N/A	N/A	N/A	N/A
0.82	0 of 1	N/A	N/A	N/A	N/A
2.05	1 of 1	2.029e0	99.0	N/A	N/A
5.12	1 of 1	5.042e0	98.5	N/A	N/A
12.80	1 of 1	1.307e1	102.1	N/A	N/A
32.00	1 of 1	3.239e1	101.2	N/A	N/A
80.00	1 of 1	7.932e1	99.2	N/A	N/A
200.00	1 of 1	2.001e2	100.1	N/A	N/A

Analyte Name: LM-flavones-20_1

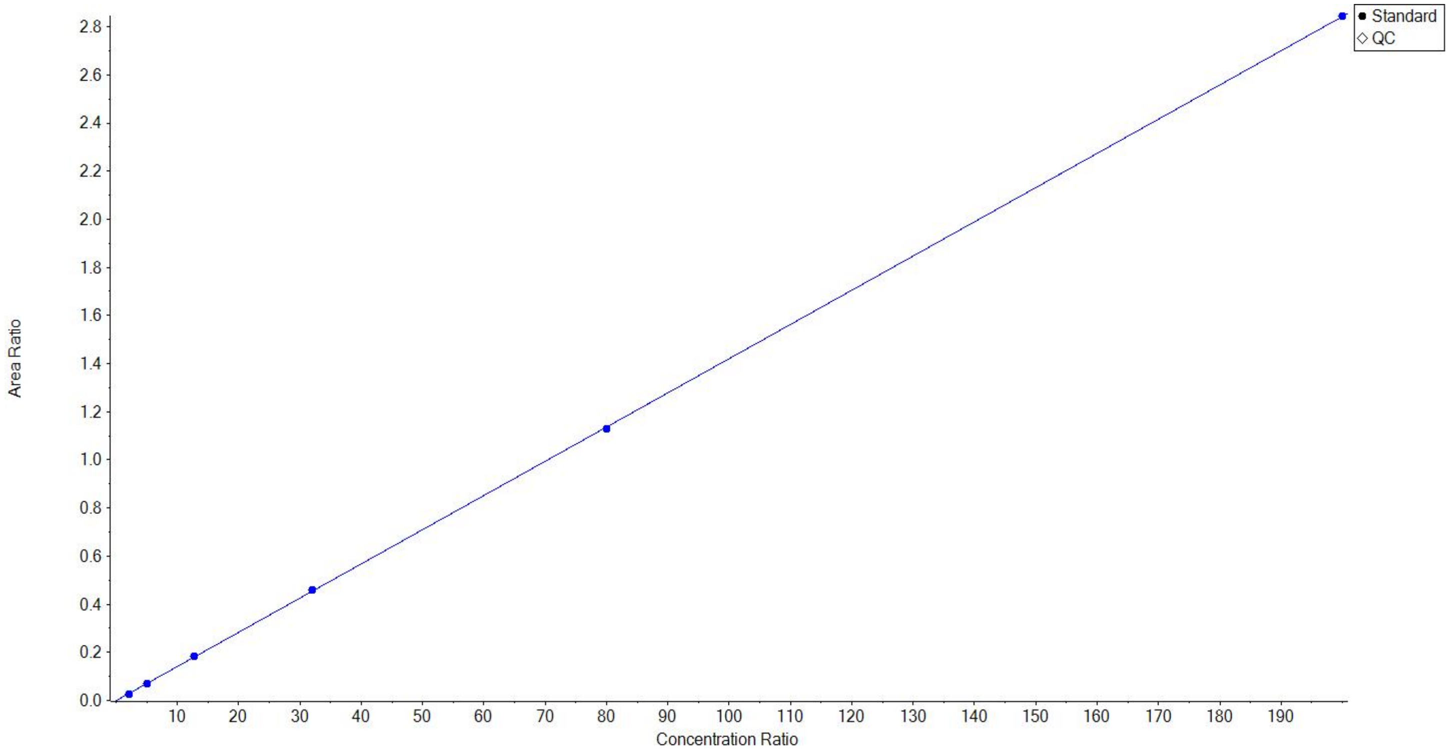
Standard
QC
Area Ratio
Concentration Ratio
0.0
0.2
0.4
0.6
0.8
1.0
1.2
1.4
1.6
1.8
2.0
2.2
2.4
2.6
2.8
10
20
30
40
50
60
70
80
90
100
110
120
130
140
150
160
170
180
190

Analyte Name: LM-flavones-22_1
Internal Standard: LM-flavones-IS02_1

**Data File**	flavones-STD-20230908.wiff	**Result Table**	DZLM2023082419-results-20230913-5500
**Acquisition Date**	9/8/2023 5:37:08 PM	**Algorithm Used**	MQ4
**Acquisition Method**	20230908-flavones-(mix130-T3)-15min.dam	**Instrument Name**	QTRAP 6500+ Low Mass
**Project**	N/A	**Processing Method**	20230412-flavones-(mix130-T3)-15min.qmethod

Regression Equation: $y = 0.00134\ x + 1.41460e-4$ ($r = 0.99921$, $r^2 = 0.99841$) (weighting: 1 / x)

Expected Concentration	Number of Values	Mean Calculated Concentration (No data for Analyte Unit)	% Accuracy	Std. Deviation	%CV
0.01	0 of 1	N/A	N/A	N/A	N/A
0.02	0 of 1	N/A	N/A	N/A	N/A
0.05	0 of 1	N/A	N/A	N/A	N/A
0.13	0 of 1	N/A	N/A	N/A	N/A
0.33	0 of 1	N/A	N/A	N/A	N/A
0.82	0 of 1	N/A	N/A	N/A	N/A
2.05	1 of 1	1.923e0	93.8	N/A	N/A
5.12	1 of 1	5.187e0	101.3	N/A	N/A
12.80	1 of 1	1.218e1	95.2	N/A	N/A
32.00	1 of 1	3.510e1	109.7	N/A	N/A
80.00	1 of 1	8.166e1	102.1	N/A	N/A
200.00	1 of 1	1.959e2	98.0	N/A	N/A

Analyte Name: LM-flavones-22_1

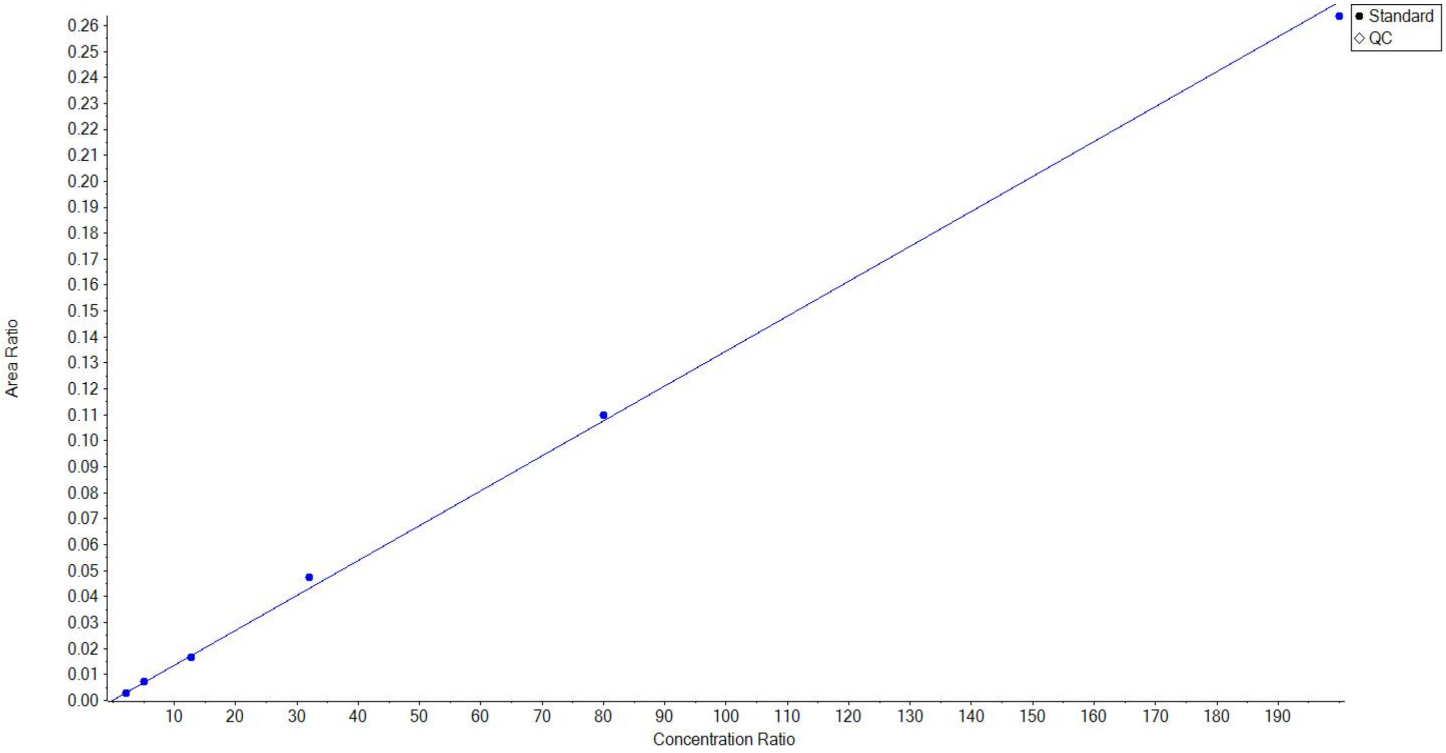
Standard
QC
Area Ratio
Concentration Ratio
0.00
0.01
0.02
0.03
0.04
0.05
0.06
0.07
0.08
0.09
0.10
0.11
0.12
0.13
0.14
0.15
0.16
0.17
0.18
0.19
0.20
0.21
0.22
0.23
0.24
0.25
0.26
10
20
30
40
50
60
70
80
90
100
110
120
130
140
150
160
170
180
190

Analyte Name: LM-flavones-26_1
Internal Standard: LM-flavones-IS02_1

**Data File**	flavones-STD-20230908.wiff	**Result Table**	DZLM2023082419-results-20230913-5500
**Acquisition Date**	9/8/2023 5:37:08 PM	**Algorithm Used**	MQ4
**Acquisition Method**	20230908-flavones-(mix130-T3)-15min.dam	**Instrument Name**	QTRAP 6500+ Low Mass
**Project**	N/A	**Processing Method**	20230412-flavones-(mix130-T3)-15min.qmethod

Regression Equation: y = 0.00871 x + 0.00433 (r = 0.99863, r^2 = 0.99727) (weighting: 1 / x)

Expected Concentration	Number of Values	Mean Calculated Concentration (No data for Analyte Unit)	% Accuracy	Std. Deviation	%CV
0.01	0 of 1	N/A	N/A	N/A	N/A
0.02	0 of 1	N/A	N/A	N/A	N/A
0.05	0 of 1	N/A	N/A	N/A	N/A
0.13	0 of 1	N/A	N/A	N/A	N/A
0.33	0 of 1	N/A	N/A	N/A	N/A
0.82	0 of 1	N/A	N/A	N/A	N/A
2.05	1 of 1	1.783e0	87.0	N/A	N/A
5.12	1 of 1	5.251e0	102.6	N/A	N/A
12.80	1 of 1	1.398e1	109.2	N/A	N/A
32.00	1 of 1	3.335e1	104.2	N/A	N/A
80.00	1 of 1	7.760e1	97.0	N/A	N/A
200.00	0 of 1	N/A	N/A	N/A	N/A

Analyte Name: LM-flavones-26_1

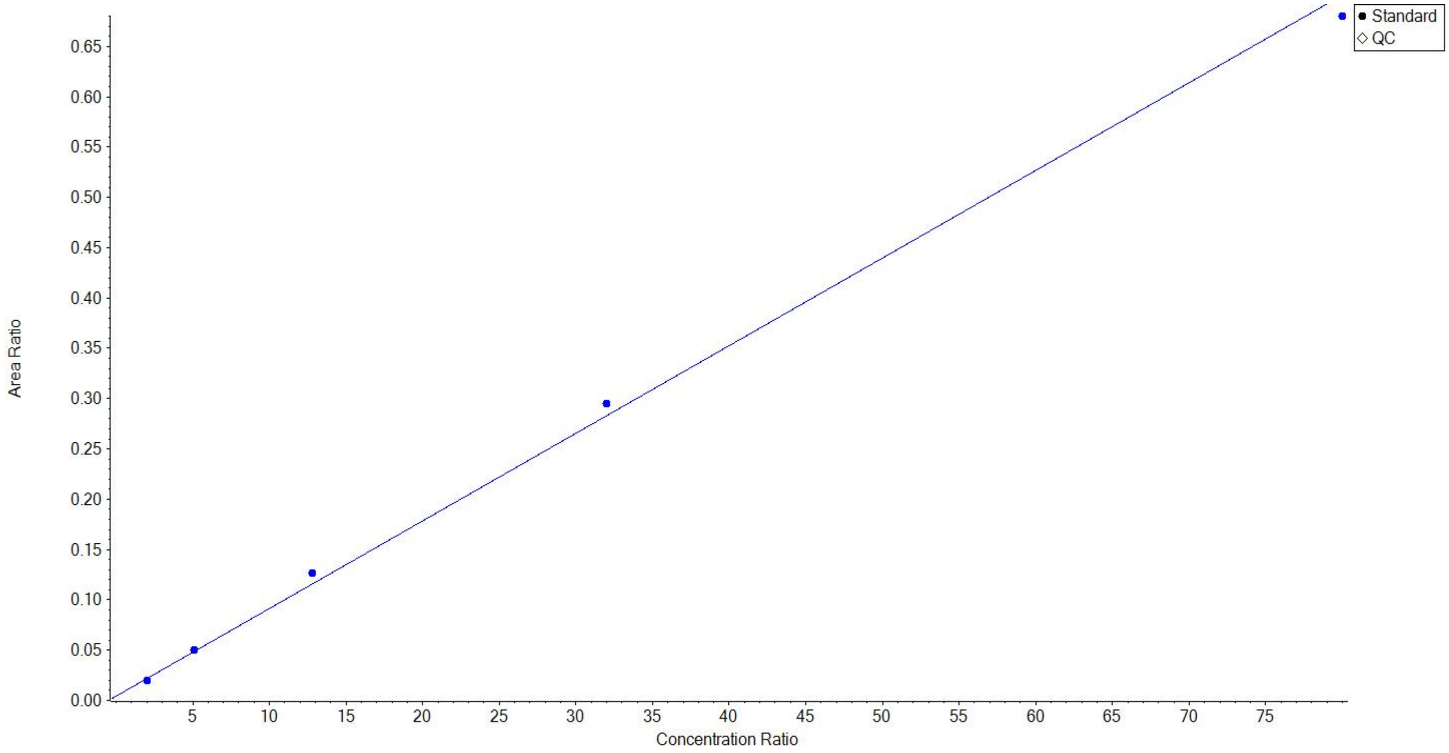
Standard
QC
Area Ratio
0.65
0.60
0.55
0.50
0.45
0.40
0.35
0.30
0.25
0.20
0.15
0.10
0.05
0.00
5
10
15
20
25
30
35
40
45
50
55
60
65
70
75
Concentration Ratio

Analyte Name: LM-flavones-28_1
Internal Standard: LM-flavones-IS02_1

**Data File**	flavones-STD-20230908.wiff	**Result Table**	DZLM2023082419-results-20230913-5500
**Acquisition Date**	9/8/2023 5:37:08 PM	**Algorithm Used**	MQ4
**Acquisition Method**	20230908-flavones-(mix130-T3)-15min.dam	**Instrument Name**	QTRAP 6500+ Low Mass
**Project**	N/A	**Processing Method**	20230412-flavones-(mix130-T3)-15min.qmethod

Regression Equation: y = 0.00375 x + -2.95802e-4 (r = 0.99990, r^2 = 0.99981) (weighting: 1 / x)

Expected Concentration	Number of Values	Mean Calculated Concentration (No data for Analyte Unit)	% Accuracy	Std. Deviation	%CV
0.01	0 of 1	N/A	N/A	N/A	N/A
0.02	0 of 1	N/A	N/A	N/A	N/A
0.05	0 of 1	N/A	N/A	N/A	N/A
0.13	0 of 1	N/A	N/A	N/A	N/A
0.33	0 of 1	N/A	N/A	N/A	N/A
0.82	0 of 1	N/A	N/A	N/A	N/A
2.05	1 of 1	2.115e0	103.2	N/A	N/A
5.12	1 of 1	4.914e0	96.0	N/A	N/A
12.80	1 of 1	1.264e1	98.8	N/A	N/A
32.00	1 of 1	3.302e1	103.2	N/A	N/A
80.00	1 of 1	7.906e1	98.8	N/A	N/A
200.00	1 of 1	2.002e2	100.1	N/A	N/A

Analyte Name: LM-flavones-28_1

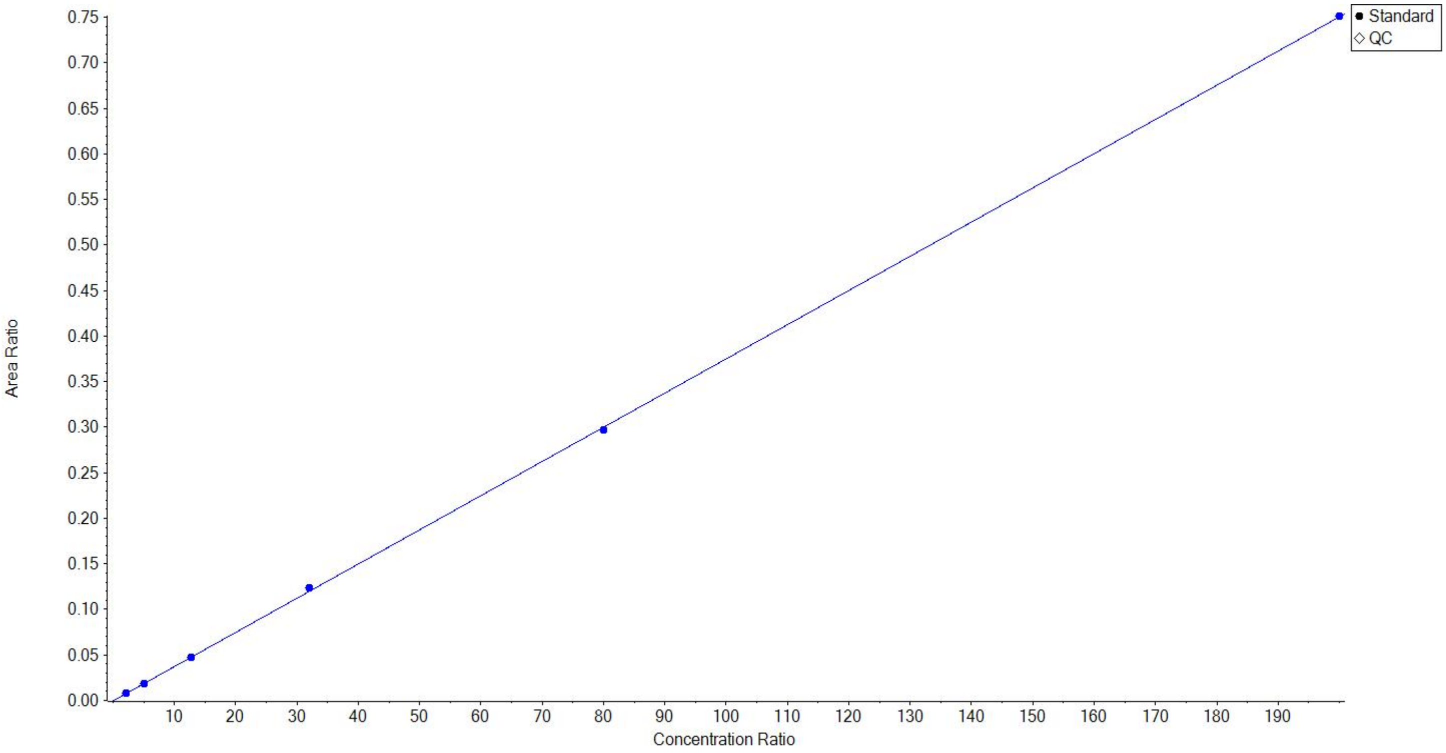
Standard
QC
Area Ratio
Concentration Ratio
0.00
0.05
0.10
0.15
0.20
0.25
0.30
0.35
0.40
0.45
0.50
0.55
0.60
0.65
0.70
0.75
10
20
30
40
50
60
70
80
90
100
110
120
130
140
150
160
170
180
190

Analyte Name: LM-flavones-29_1
Internal Standard: LM-flavones-IS02_1

**Data File**	flavones-STD-20230908.wiff	**Result Table**	DZLM2023082419-results-20230913-5500
**Acquisition Date**	9/8/2023 5:37:08 PM	**Algorithm Used**	MQ4
**Acquisition Method**	20230908-flavones-(mix130-T3)-15min.dam	**Instrument Name**	QTRAP 6500+ Low Mass
**Project**	N/A	**Processing Method**	20230412-flavones-(mix130-T3)-15min.qmethod

Regression Equation: y = 0.00719 x + -0.01647 (r = 0.99938, r^2 = 0.99877) (weighting: 1 / x)

Expected Concentration	Number of Values	Mean Calculated Concentration (No data for Analyte Unit)	% Accuracy	Std. Deviation	%CV
0.01	0 of 1	N/A	N/A	N/A	N/A
0.02	0 of 1	N/A	N/A	N/A	N/A
0.05	0 of 1	N/A	N/A	N/A	N/A
0.13	0 of 1	N/A	N/A	N/A	N/A
0.33	0 of 1	N/A	N/A	N/A	N/A
0.82	0 of 1	N/A	N/A	N/A	N/A
2.05	0 of 1	N/A	N/A	N/A	N/A
5.12	1 of 1	5.732e0	112.0	N/A	N/A
12.80	1 of 1	1.193e1	93.2	N/A	N/A
32.00	1 of 1	3.054e1	95.5	N/A	N/A
80.00	1 of 1	7.808e1	97.6	N/A	N/A
200.00	1 of 1	2.036e2	101.8	N/A	N/A

Analyte Name: LM-flavones-29_1

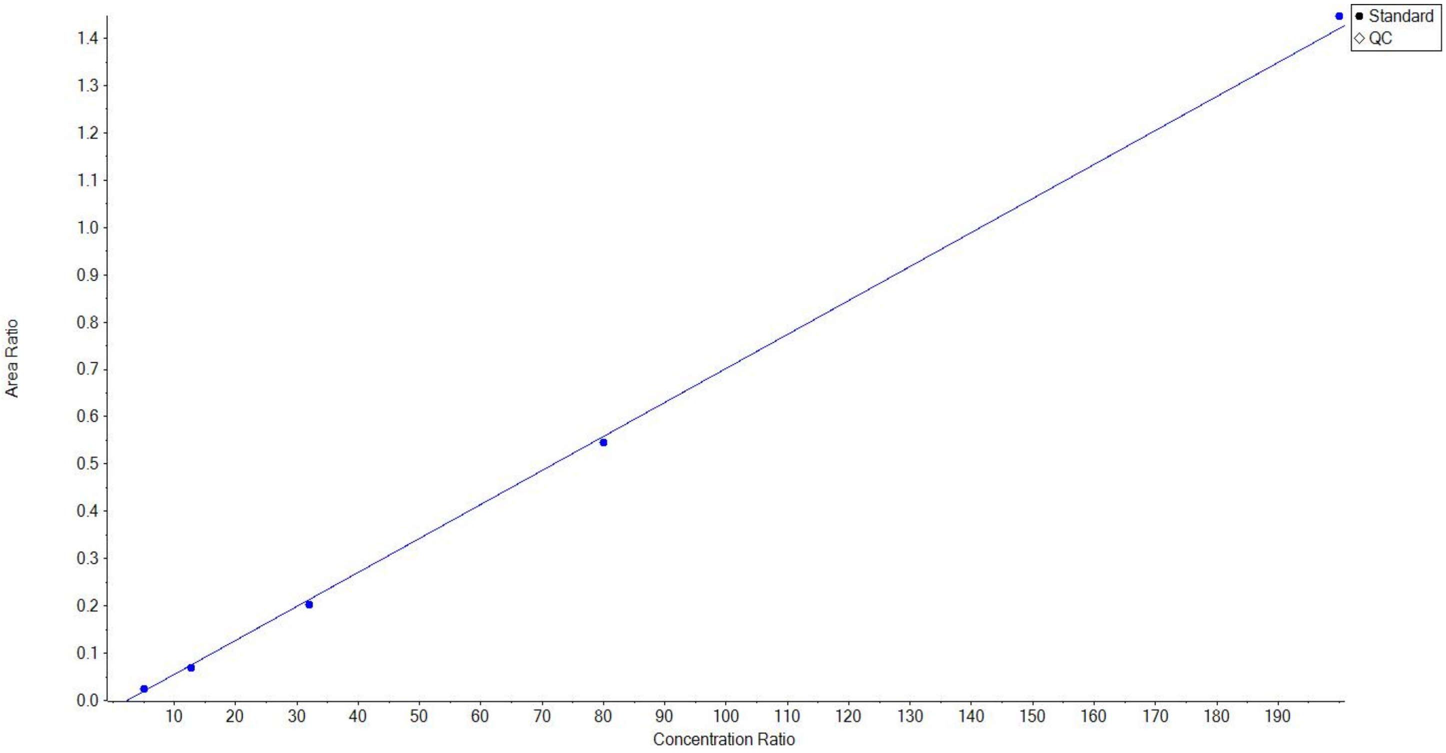
Standard
QC
Area Ratio
1.4
1.3
1.2
1.1
1.0
0.9
0.8
0.7
0.6
0.5
0.4
0.3
0.2
0.1
0.0
10 20 30 40 50 60 70 80 90 100 110 120 130 140 150 160 170 180 190
Concentration Ratio

Analyte Name: LM-flavones-31
Internal Standard: LM-flavones-IS02_1

**Data File**	flavones-STD-20230908.wiff	**Result Table**	DZLM2023082419-results-20230913-5500
**Acquisition Date**	9/8/2023 5:37:08 PM	**Algorithm Used**	MQ4
**Acquisition Method**	20230908-flavones-(mix130-T3)-15min.dam	**Instrument Name**	QTRAP 6500+ Low Mass
**Project**	N/A	**Processing Method**	20230412-flavones-(mix130-T3)-15min.qmethod

Regression Equation: $y = 0.01138 x + -0.00330$ ($r = 0.99973$, $r^2 = 0.99946$) (weighting: 1 / x)

Expected Concentration	Number of Values	Mean Calculated Concentration (No data for Analyte Unit)	% Accuracy	Std. Deviation	%CV
0.01	0 of 1	N/A	N/A	N/A	N/A
0.02	0 of 1	N/A	N/A	N/A	N/A
0.05	0 of 1	N/A	N/A	N/A	N/A
0.13	0 of 1	N/A	N/A	N/A	N/A
0.33	0 of 1	N/A	N/A	N/A	N/A
0.82	0 of 1	N/A	N/A	N/A	N/A
2.05	1 of 1	2.191e0	106.9	N/A	N/A
5.12	1 of 1	5.101e0	99.6	N/A	N/A
12.80	1 of 1	1.228e1	95.9	N/A	N/A
32.00	1 of 1	3.170e1	99.1	N/A	N/A
80.00	1 of 1	7.757e1	97.0	N/A	N/A
200.00	1 of 1	2.031e2	101.6	N/A	N/A

Analyte Name: LM-flavones-31

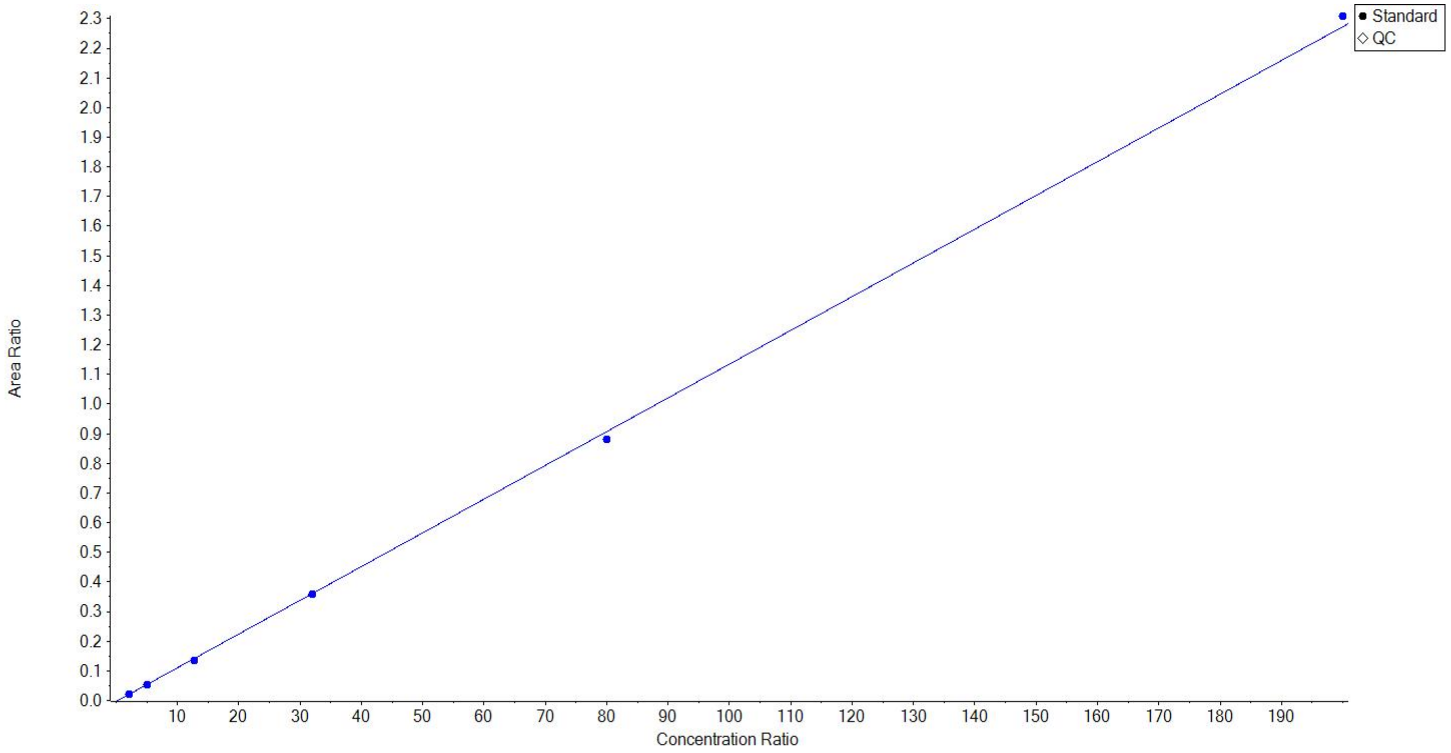
2.3
2.2
2.1
2.0
1.9
1.8
1.7
1.6
1.5
1.4
1.3
1.2
1.1
1.0
0.9
0.8
0.7
0.6
0.5
0.4
0.3
0.2
0.1
0.0
Area Ratio
10 20 30 40 50 60 70 80 90 100 110 120 130 140 150 160 170 180 190
Concentration Ratio
Standard
QC

Analyte Name: LM-flavones-30_1
Internal Standard: LM-flavones-IS02_1

**Data File**	flavones-STD-20230908.wiff	**Result Table**	DZLM2023082419-results-20230913-5500
**Acquisition Date**	9/8/2023 5:37:08 PM	**Algorithm Used**	MQ4
**Acquisition Method**	20230908-flavones-(mix130-T3)-15min.dam	**Instrument Name**	QTRAP 6500+ Low Mass
**Project**	N/A	**Processing Method**	20230412-flavones-(mix130-T3)-15min.qmethod

Regression Equation: y = 0.00291 x + -0.00129 (r = 0.99980, r^2 = 0.99960) (weighting: 1 / x)

Expected Concentration	Number of Values	Mean Calculated Concentration (No data for Analyte Unit)	% Accuracy	Std. Deviation	%CV
0.01	0 of 1	N/A	N/A	N/A	N/A
0.02	0 of 1	N/A	N/A	N/A	N/A
0.05	0 of 1	N/A	N/A	N/A	N/A
0.13	0 of 1	N/A	N/A	N/A	N/A
0.33	0 of 1	N/A	N/A	N/A	N/A
0.82	0 of 1	N/A	N/A	N/A	N/A
2.05	1 of 1	2.188e0	106.7	N/A	N/A
5.12	1 of 1	5.071e0	99.1	N/A	N/A
12.80	1 of 1	1.229e1	96.0	N/A	N/A
32.00	1 of 1	3.182e1	99.4	N/A	N/A
80.00	1 of 1	7.802e1	97.5	N/A	N/A
200.00	1 of 1	2.026e2	101.3	N/A	N/A

Analyte Name: LM-flavones-30_1

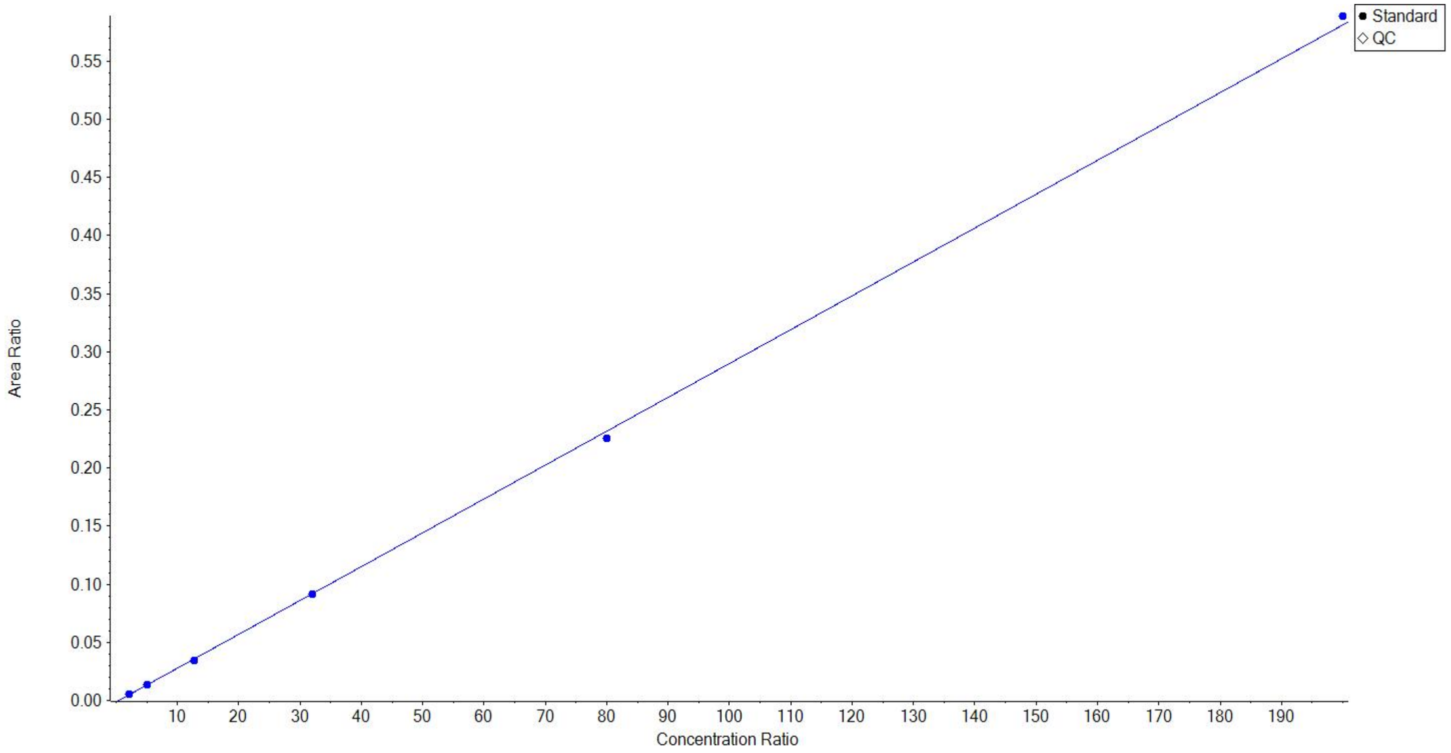
Standard
QC
Area Ratio
0.55
0.50
0.45
0.40
0.35
0.30
0.25
0.20
0.15
0.10
0.05
0.00
10
20
30
40
50
60
70
80
90
100
110
120
130
140
150
160
170
180
190
Concentration Ratio

Analyte Name: LM-flavones-32_1
Internal Standard: LM-flavones-IS02_1

**Data File**	flavones-STD-20230908.wiff	**Result Table**	DZLM2023082419-results-20230913-5500
**Acquisition Date**	9/8/2023 5:37:08 PM	**Algorithm Used**	MQ4
**Acquisition Method**	20230908-flavones-(mix130-T3)-15min.dam	**Instrument Name**	QTRAP 6500+ Low Mass
**Project**	N/A	**Processing Method**	20230412-flavones-(mix130-T3)-15min.qmethod

Regression Equation: y = 0.00168 x + -3.67582e-4 (r = 0.99970, r² = 0.99940) (weighting: 1 / x)

Expected Concentration	Number of Values	Mean Calculated Concentration (No data for Analyte Unit)	% Accuracy	Std. Deviation	%CV
0.01	0 of 1	N/A	N/A	N/A	N/A
0.02	0 of 1	N/A	N/A	N/A	N/A
0.05	0 of 1	N/A	N/A	N/A	N/A
0.13	0 of 1	N/A	N/A	N/A	N/A
0.33	0 of 1	N/A	N/A	N/A	N/A
0.82	0 of 1	N/A	N/A	N/A	N/A
2.05	1 of 1	2.344e0	114.3	N/A	N/A
5.12	1 of 1	4.558e0	89.0	N/A	N/A
12.80	1 of 1	1.231e1	96.2	N/A	N/A
32.00	1 of 1	3.245e1	101.4	N/A	N/A
80.00	1 of 1	7.852e1	98.2	N/A	N/A
200.00	1 of 1	2.018e2	100.9	N/A	N/A

Analyte Name: LM-flavones-32_1

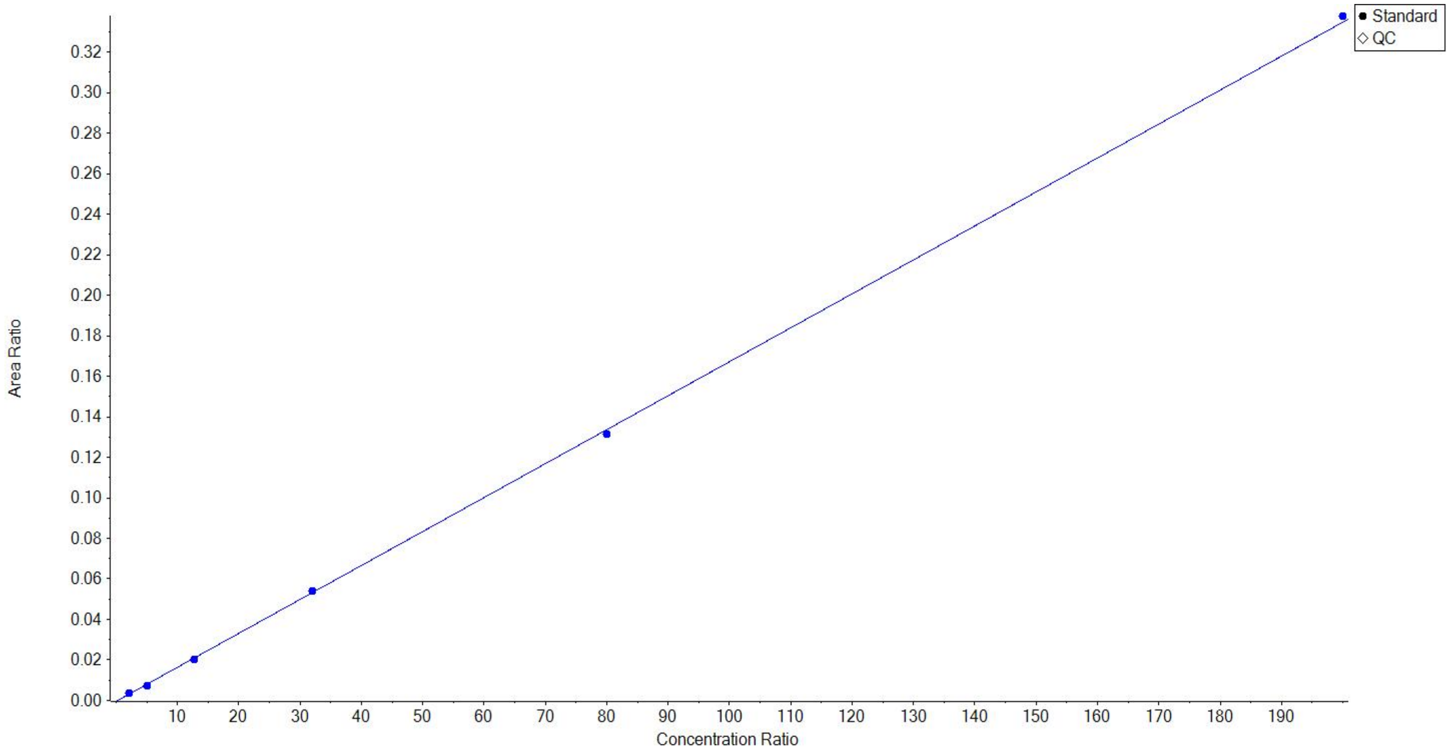
Standard
QC
Area Ratio
0.00
0.02
0.04
0.06
0.08
0.10
0.12
0.14
0.16
0.18
0.20
0.22
0.24
0.26
0.28
0.30
0.32
10
20
30
40
50
60
70
80
90
100
110
120
130
140
150
160
170
180
190
Concentration Ratio

Analyte Name: LM-flavones-33_1
Internal Standard: LM-flavones-IS02_1

**Data File**	flavones-STD-20230908.wiff	**Result Table**	DZLM2023082419-results-20230913-5500
**Acquisition Date**	9/8/2023 5:37:08 PM	**Algorithm Used**	MQ4
**Acquisition Method**	20230908-flavones-(mix130-T3)-15min.dam	**Instrument Name**	QTRAP 6500+ Low Mass
**Project**	N/A	**Processing Method**	20230412-flavones-(mix130-T3)-15min.qmethod

Regression Equation: y = 0.00373 x + -4.47678e-4 (r = 0.99984, r² = 0.99968) (weighting: 1 / x)

Expected Concentration	Number of Values	Mean Calculated Concentration (No data for Analyte Unit)	% Accuracy	Std. Deviation	%CV
0.01	0 of 1	N/A	N/A	N/A	N/A
0.02	0 of 1	N/A	N/A	N/A	N/A
0.05	0 of 1	N/A	N/A	N/A	N/A
0.13	0 of 1	N/A	N/A	N/A	N/A
0.33	0 of 1	N/A	N/A	N/A	N/A
0.82	0 of 1	N/A	N/A	N/A	N/A
2.05	1 of 1	2.108e0	102.8	N/A	N/A
5.12	1 of 1	5.104e0	99.7	N/A	N/A
12.80	1 of 1	1.226e1	95.8	N/A	N/A
32.00	1 of 1	3.194e1	99.8	N/A	N/A
80.00	1 of 1	8.210e1	102.6	N/A	N/A
200.00	1 of 1	1.985e2	99.2	N/A	N/A

Analyte Name: LM-flavones-33_1

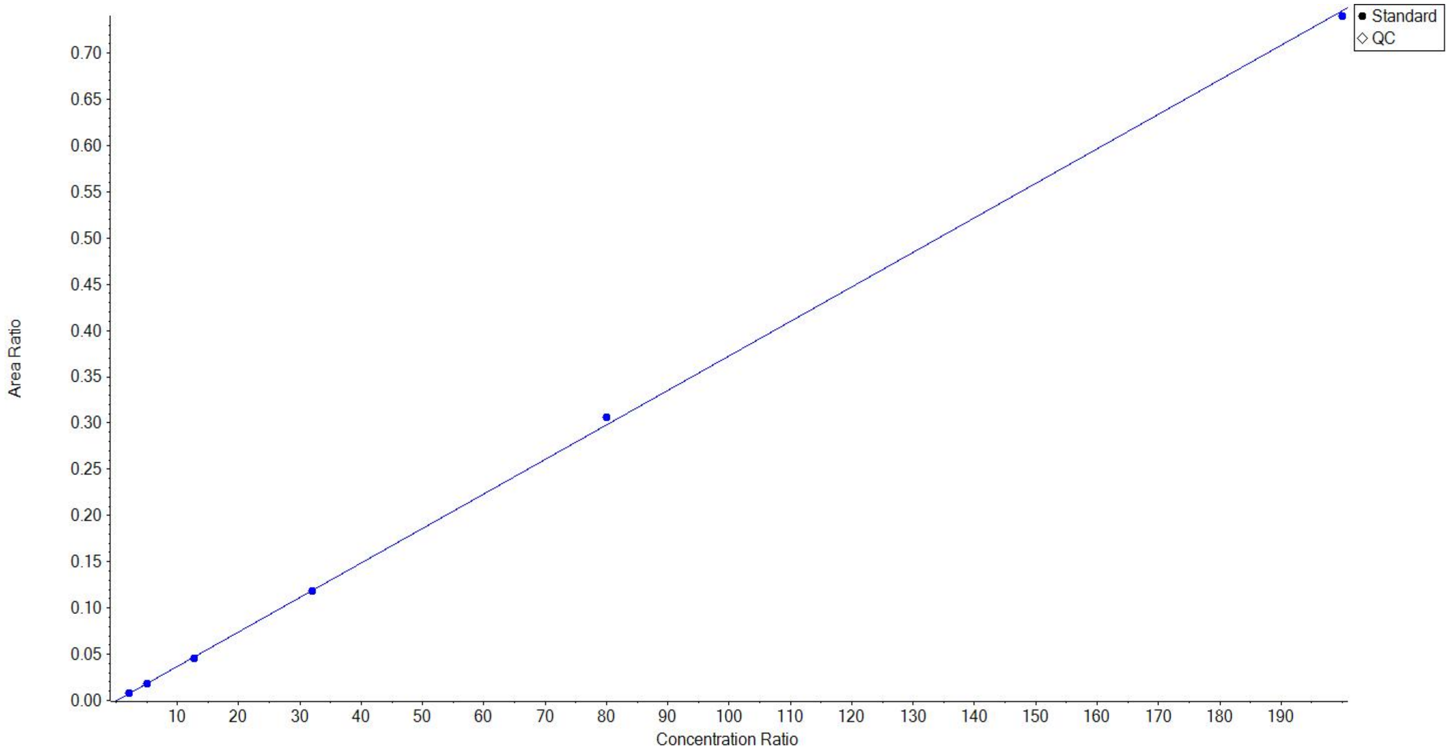
Standard
QC
Area Ratio
0.70
0.65
0.60
0.55
0.50
0.45
0.40
0.35
0.30
0.25
0.20
0.15
0.10
0.05
0.00
10
20
30
40
50
60
70
80
90
100
110
120
130
140
150
160
170
180
190
Concentration Ratio

Analyte Name: LM-flavones-34_1
Internal Standard: LM-flavones-IS02_1

**Data File**	flavones-STD-20230908.wiff	**Result Table**	DZLM2023082419-results-20230913-5500
**Acquisition Date**	9/8/2023 5:37:08 PM	**Algorithm Used**	MQ4
**Acquisition Method**	20230908-flavones-(mix130-T3)-15min.dam	**Instrument Name**	QTRAP 6500+ Low Mass
**Project**	N/A	**Processing Method**	20230412-flavones-(mix130-T3)-15min.qmethod

Regression Equation: y = 0.00186 x + -1.23536e-4 (r = 0.99942, r^2 = 0.99883) (weighting: 1 / x)

Expected Concentration	Number of Values	Mean Calculated Concentration (No data for Analyte Unit)	% Accuracy	Std. Deviation	%CV
0.01	0 of 1	N/A	N/A	N/A	N/A
0.02	0 of 1	N/A	N/A	N/A	N/A
0.05	0 of 1	N/A	N/A	N/A	N/A
0.13	0 of 1	N/A	N/A	N/A	N/A
0.33	0 of 1	N/A	N/A	N/A	N/A
0.82	0 of 1	N/A	N/A	N/A	N/A
2.05	1 of 1	2.177e0	106.2	N/A	N/A
5.12	1 of 1	5.242e0	102.4	N/A	N/A
12.80	1 of 1	1.164e1	90.9	N/A	N/A
32.00	1 of 1	3.095e1	96.7	N/A	N/A
80.00	1 of 1	8.369e1	104.6	N/A	N/A
200.00	1 of 1	1.983e2	99.1	N/A	N/A

Analyte Name: LM-flavones-34_1

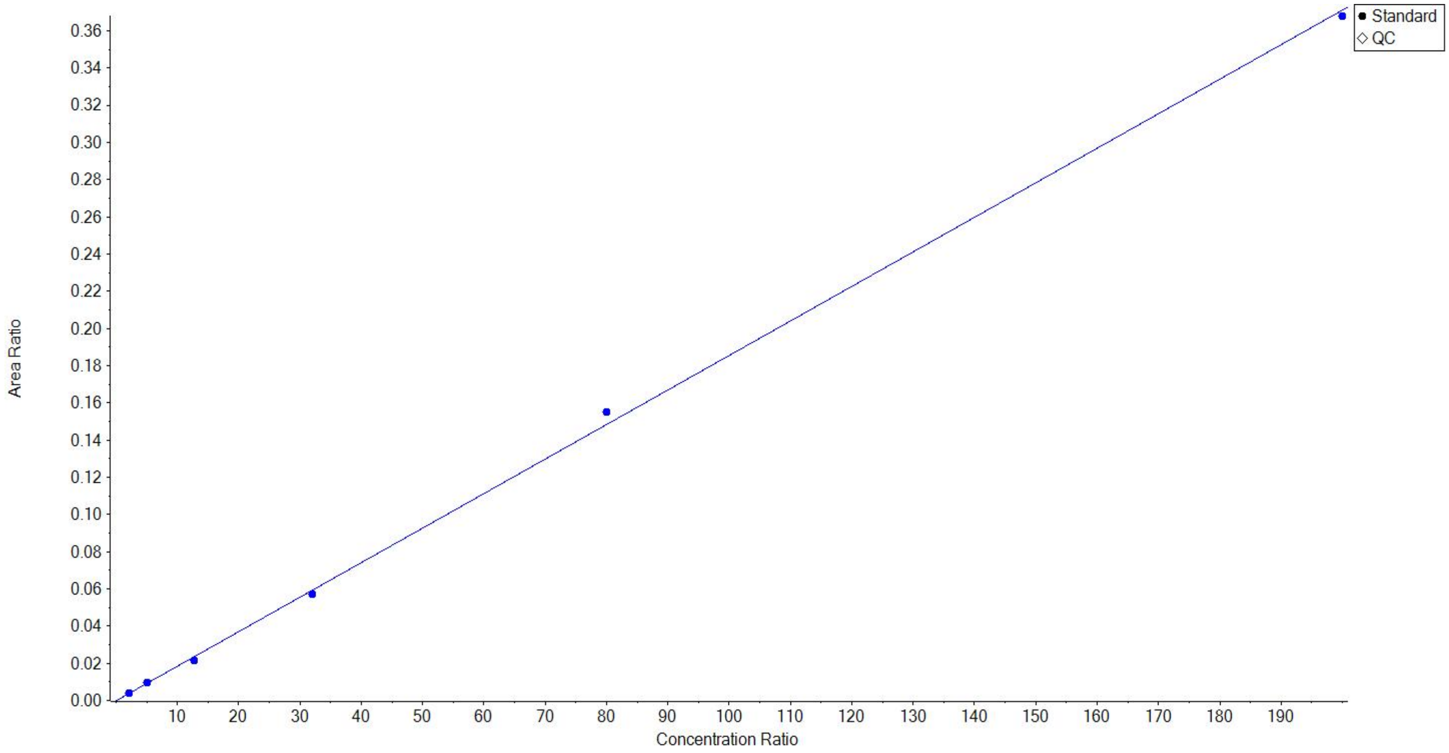
Standard
QC
Area Ratio
0.36
0.34
0.32
0.30
0.28
0.26
0.24
0.22
0.20
0.18
0.16
0.14
0.12
0.10
0.08
0.06
0.04
0.02
0.00
10
20
30
40
50
60
70
80
90
100
110
120
130
140
150
160
170
180
190
Concentration Ratio

Analyte Name: LM-flavones-35_1
Internal Standard: LM-flavones-IS02_1

**Data File**	flavones-STD-20230908.wiff	**Result Table**	DZLM2023082419-results-20230913-5500
**Acquisition Date**	9/8/2023 5:37:08 PM	**Algorithm Used**	MQ4
**Acquisition Method**	20230908-flavones-(mix130-T3)-15min.dam	**Instrument Name**	QTRAP 6500+ Low Mass
**Project**	N/A	**Processing Method**	20230412-flavones-(mix130-T3)-15min.qmethod

Regression Equation: y = 0.00344 x + -1.55492e-4 (r = 0.99983, r^2 = 0.99967) (weighting: 1 / x)

Expected Concentration	Number of Values	Mean Calculated Concentration (No data for Analyte Unit)	% Accuracy	Std. Deviation	%CV
0.01	0 of 1	N/A	N/A	N/A	N/A
0.02	0 of 1	N/A	N/A	N/A	N/A
0.05	0 of 1	N/A	N/A	N/A	N/A
0.13	0 of 1	N/A	N/A	N/A	N/A
0.33	0 of 1	N/A	N/A	N/A	N/A
0.82	0 of 1	N/A	N/A	N/A	N/A
2.05	1 of 1	2.263e0	110.4	N/A	N/A
5.12	1 of 1	4.889e0	95.5	N/A	N/A
12.80	1 of 1	1.193e1	93.2	N/A	N/A
32.00	1 of 1	3.222e1	100.7	N/A	N/A
80.00	1 of 1	7.985e1	99.8	N/A	N/A
200.00	1 of 1	2.008e2	100.4	N/A	N/A

Analyte Name: LM-flavones-35_1

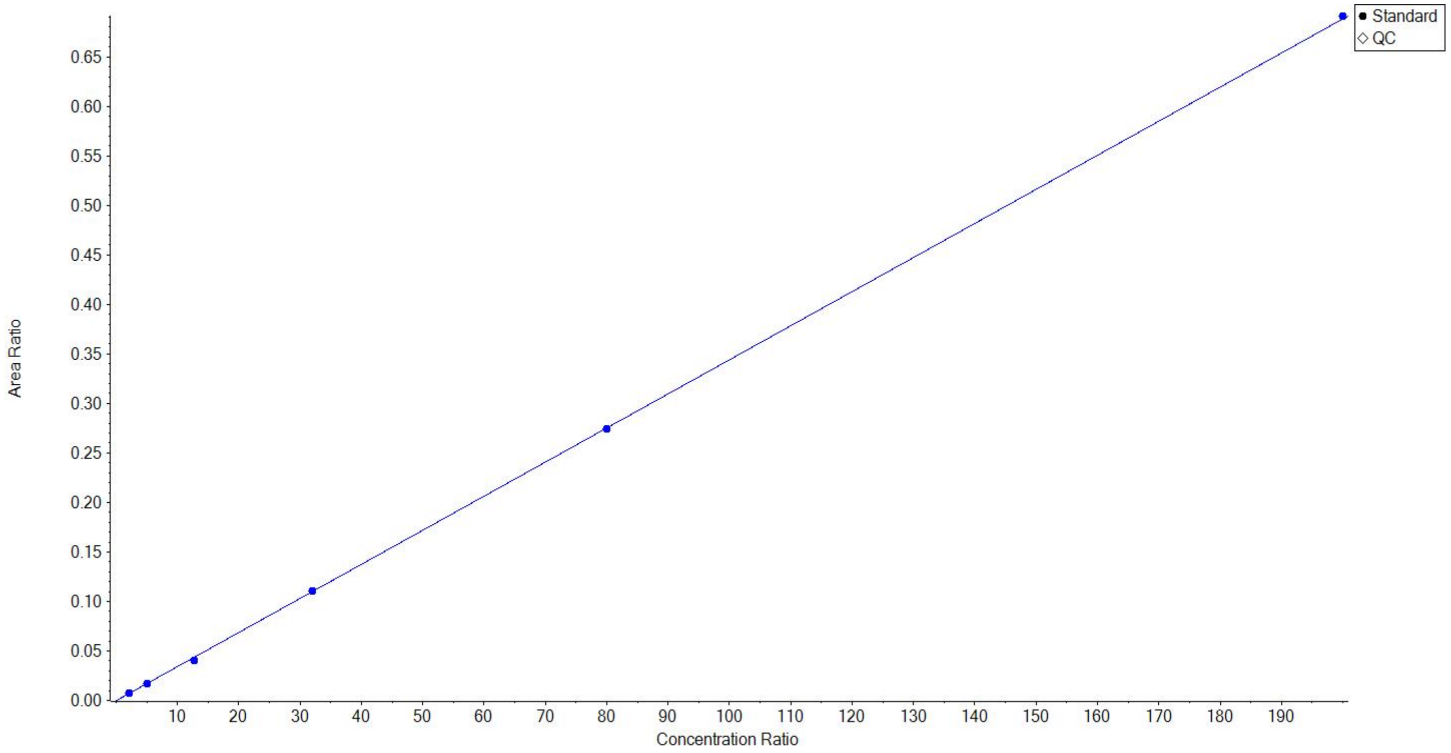
Standard
QC
Area Ratio
Concentration Ratio
0.00
0.05
0.10
0.15
0.20
0.25
0.30
0.35
0.40
0.45
0.50
0.55
0.60
0.65
10
20
30
40
50
60
70
80
90
100
110
120
130
140
150
160
170
180
190

Analyte Name: LM-flavones-36_1
Internal Standard: LM-flavones-IS02_1

**Data File**	flavones-STD-20230908.wiff	**Result Table**	DZLM2023082419-results-20230913-5500
**Acquisition Date**	9/8/2023 5:37:08 PM	**Algorithm Used**	MQ4
**Acquisition Method**	20230908-flavones-(mix130-T3)-15min.dam	**Instrument Name**	QTRAP 6500+ Low Mass
**Project**	N/A	**Processing Method**	20230412-flavones-(mix130-T3)-15min.qmethod

Regression Equation: y = 4.53255e-5 x + -2.15677e-5 (r = 0.99865, r² = 0.99730) (weighting: 1 / x)

Expected Concentration	Number of Values	Mean Calculated Concentration (No data for Analyte Unit)	% Accuracy	Std. Deviation	%CV
0.01	0 of 1	N/A	N/A	N/A	N/A
0.02	0 of 1	N/A	N/A	N/A	N/A
0.05	0 of 1	N/A	N/A	N/A	N/A
0.13	0 of 1	N/A	N/A	N/A	N/A
0.33	0 of 1	N/A	N/A	N/A	N/A
0.82	0 of 1	N/A	N/A	N/A	N/A
2.05	0 of 1	N/A	N/A	N/A	N/A
5.12	1 of 1	5.912e0	115.5	N/A	N/A
12.80	1 of 1	1.135e1	88.6	N/A	N/A
32.00	1 of 1	3.156e1	98.6	N/A	N/A
80.00	1 of 1	7.562e1	94.5	N/A	N/A
200.00	1 of 1	2.055e2	102.7	N/A	N/A

Analyte Name: LM-flavones-36_1

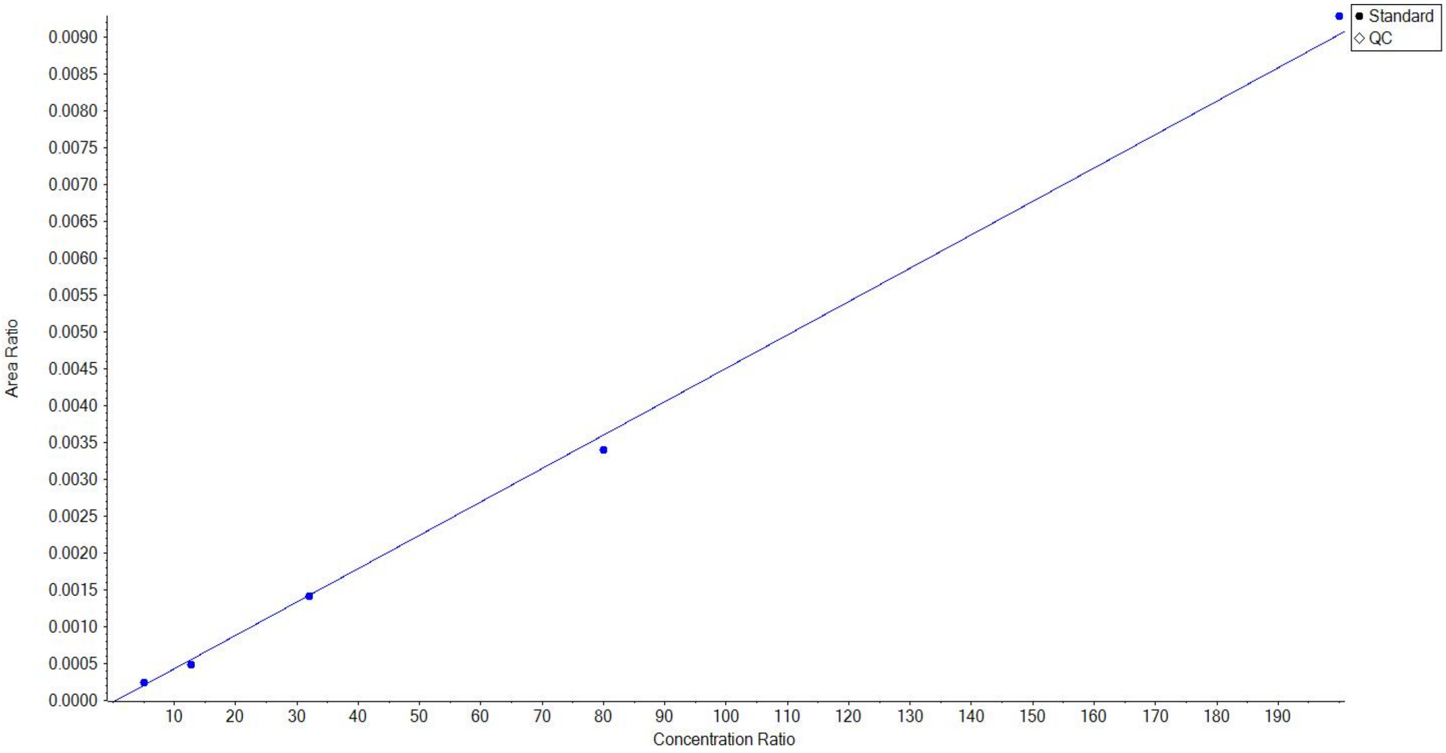
Standard
QC
Area Ratio
0.0090
0.0085
0.0080
0.0075
0.0070
0.0065
0.0060
0.0055
0.0050
0.0045
0.0040
0.0035
0.0030
0.0025
0.0020
0.0015
0.0010
0.0005
0.0000
10 20 30 40 50 60 70 80 90 100 110 120 130 140 150 160 170 180 190
Concentration Ratio

Analyte Name: LM-flavones-37_1
Internal Standard: LM-flavones-IS02_1

**Data File**	flavones-STD-20230908.wiff	**Result Table**	DZLM2023082419-results-20230913-5500
**Acquisition Date**	9/8/2023 5:37:08 PM	**Algorithm Used**	MQ4
**Acquisition Method**	20230908-flavones-(mix130-T3)-15min.dam	**Instrument Name**	QTRAP 6500+ Low Mass
**Project**	N/A	**Processing Method**	20230412-flavones-(mix130-T3)-15min.qmethod

Regression Equation: y = 0.00577 x + -0.00183 (r = 0.99987, r² = 0.99974) (weighting: 1 / x)

Expected Concentration	Number of Values	Mean Calculated Concentration (No data for Analyte Unit)	% Accuracy	Std. Deviation	%CV
0.01	0 of 1	N/A	N/A	N/A	N/A
0.02	0 of 1	N/A	N/A	N/A	N/A
0.05	0 of 1	N/A	N/A	N/A	N/A
0.13	0 of 1	N/A	N/A	N/A	N/A
0.33	0 of 1	N/A	N/A	N/A	N/A
0.82	0 of 1	N/A	N/A	N/A	N/A
2.05	1 of 1	2.160e0	105.5	N/A	N/A
5.12	1 of 1	4.916e0	96.0	N/A	N/A
12.80	1 of 1	1.221e1	95.4	N/A	N/A
32.00	1 of 1	3.300e1	103.1	N/A	N/A
80.00	1 of 1	8.021e1	100.3	N/A	N/A
200.00	1 of 1	1.995e2	99.7	N/A	N/A

Analyte Name: LM-flavones-37_1

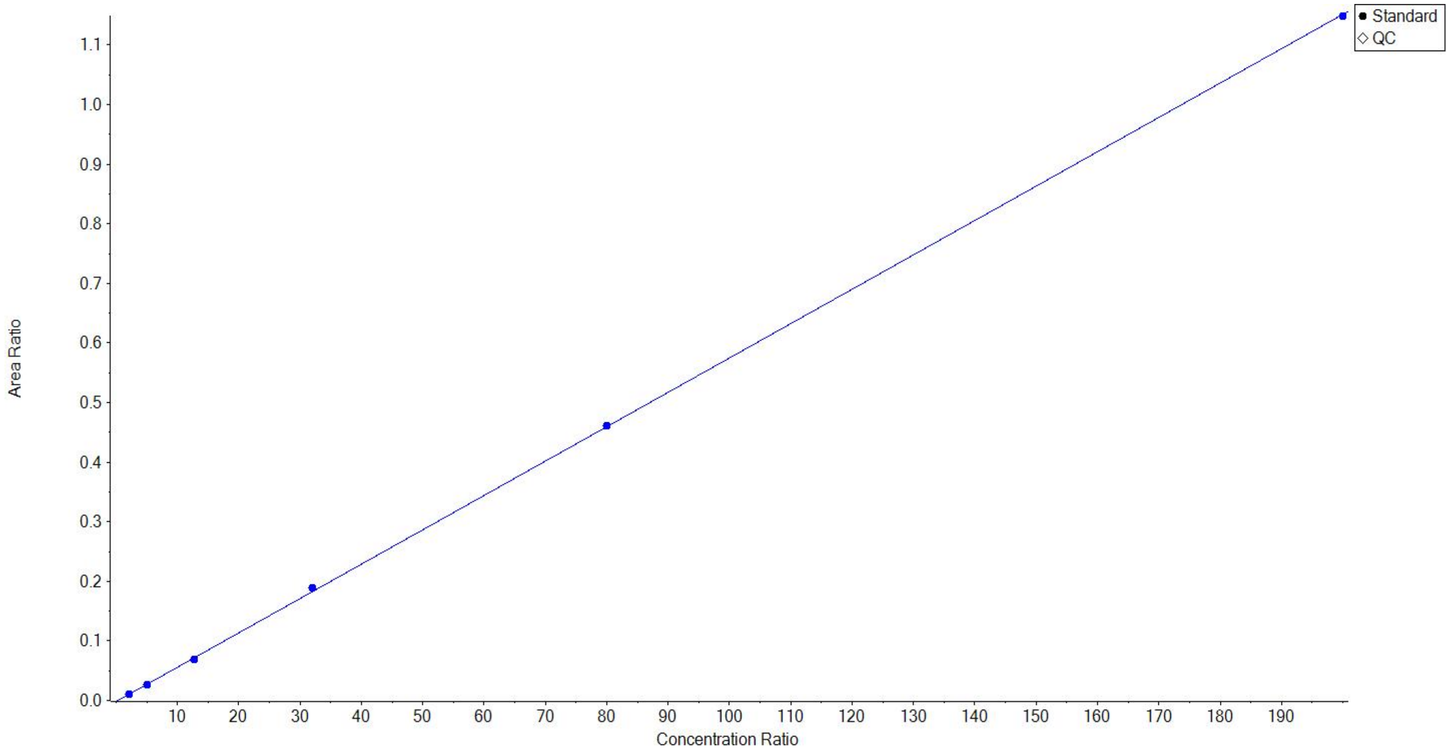
Standard
QC
Area Ratio
0.0
0.1
0.2
0.3
0.4
0.5
0.6
0.7
0.8
0.9
1.0
1.1
10
20
30
40
50
60
70
80
90
100
110
120
130
140
150
160
170
180
190
Concentration Ratio

Analyte Name: LM-flavones-38_1
Internal Standard: LM-flavones-IS02_1

**Data File**	flavones-STD-20230908.wiff	**Result Table**	DZLM2023082419-results-20230913-5500
**Acquisition Date**	9/8/2023 5:37:08 PM	**Algorithm Used**	MQ4
**Acquisition Method**	20230908-flavones-(mix130-T3)-15min.dam	**Instrument Name**	QTRAP 6500+ Low Mass
**Project**	N/A	**Processing Method**	20230412-flavones-(mix130-T3)-15min.qmethod

Regression Equation: y = 0.02327 x + -0.00341 (r = 0.99981, r² = 0.99962) (weighting: 1 / x)

Expected Concentration	Number of Values	Mean Calculated Concentration (No data for Analyte Unit)	% Accuracy	Std. Deviation	%CV
0.01	0 of 1	N/A	N/A	N/A	N/A
0.02	0 of 1	N/A	N/A	N/A	N/A
0.05	0 of 1	N/A	N/A	N/A	N/A
0.13	0 of 1	N/A	N/A	N/A	N/A
0.33	0 of 1	N/A	N/A	N/A	N/A
0.82	0 of 1	N/A	N/A	N/A	N/A
2.05	1 of 1	2.064e0	100.8	N/A	N/A
5.12	1 of 1	4.882e0	95.3	N/A	N/A
12.80	1 of 1	1.277e1	99.8	N/A	N/A
32.00	1 of 1	3.369e1	105.3	N/A	N/A
80.00	1 of 1	7.942e1	99.3	N/A	N/A
200.00	1 of 1	1.991e2	99.6	N/A	N/A

Analyte Name: LM-flavones-38_1

Created with SCIEX OS 1.6.1
Printed:9/18/2023 5:27:01 PM

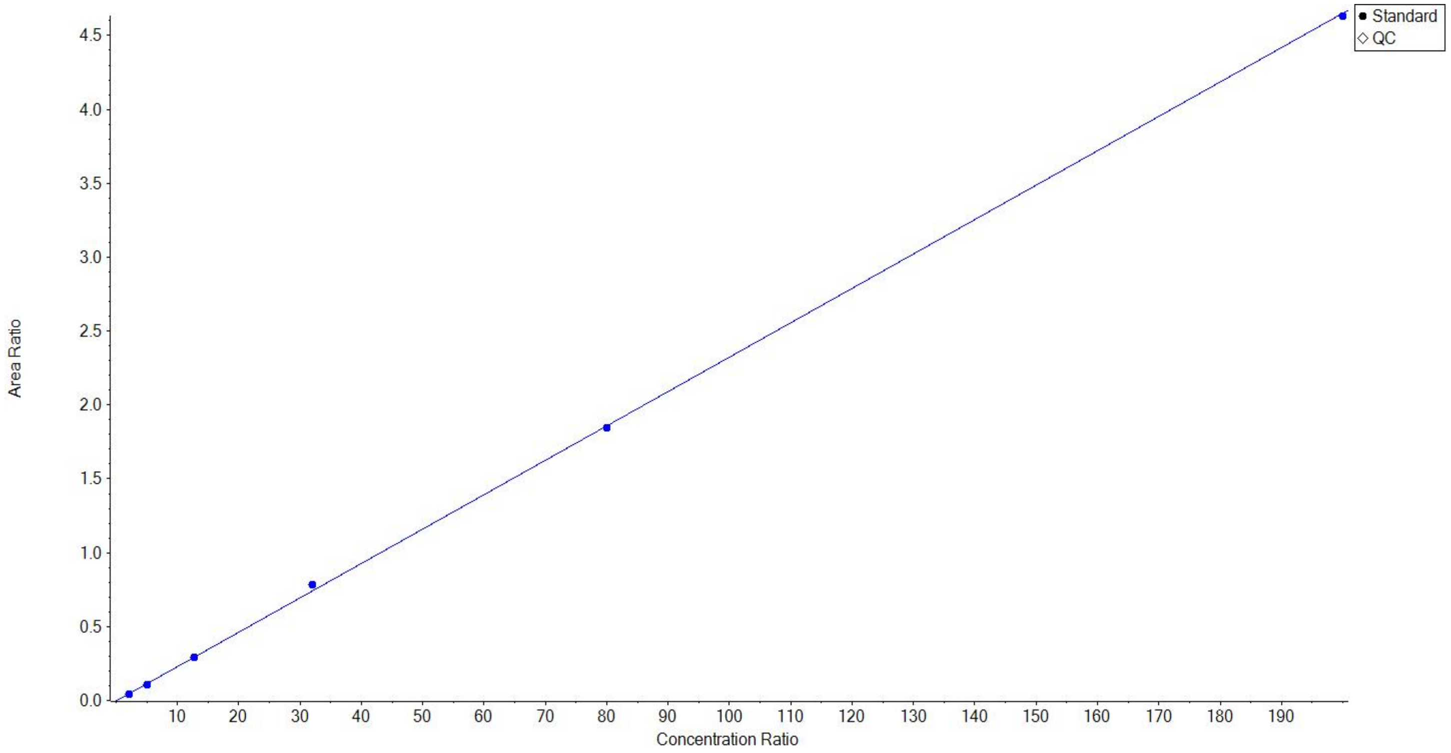

Analyte Name: LM-flavones-38_2
Internal Standard: N/A

**Data File**	flavones-STD-20230908.wiff	**Result Table**	DZLM2023082419-results-20230913-5500
**Acquisition Date**	9/8/2023 5:37:08 PM	**Algorithm Used**	MQ4
**Acquisition Method**	20230908-flavones-(mix130-T3)-15min.dam	**Instrument Name**	QTRAP 6500+ Low Mass
**Project**	N/A	**Processing Method**	20230412-flavones-(mix130-T3)-15min.qmethod

Regression Equation: Degenerate regression equation.

Expected Concentration	Number of Values	Mean Calculated Concentration (No data for Analyte Unit)	% Accuracy	Std. Deviation	%CV
0.00	0 of 12	N/A	N/A	N/A	N/A

Analyte Name: LM-flavones-38_2

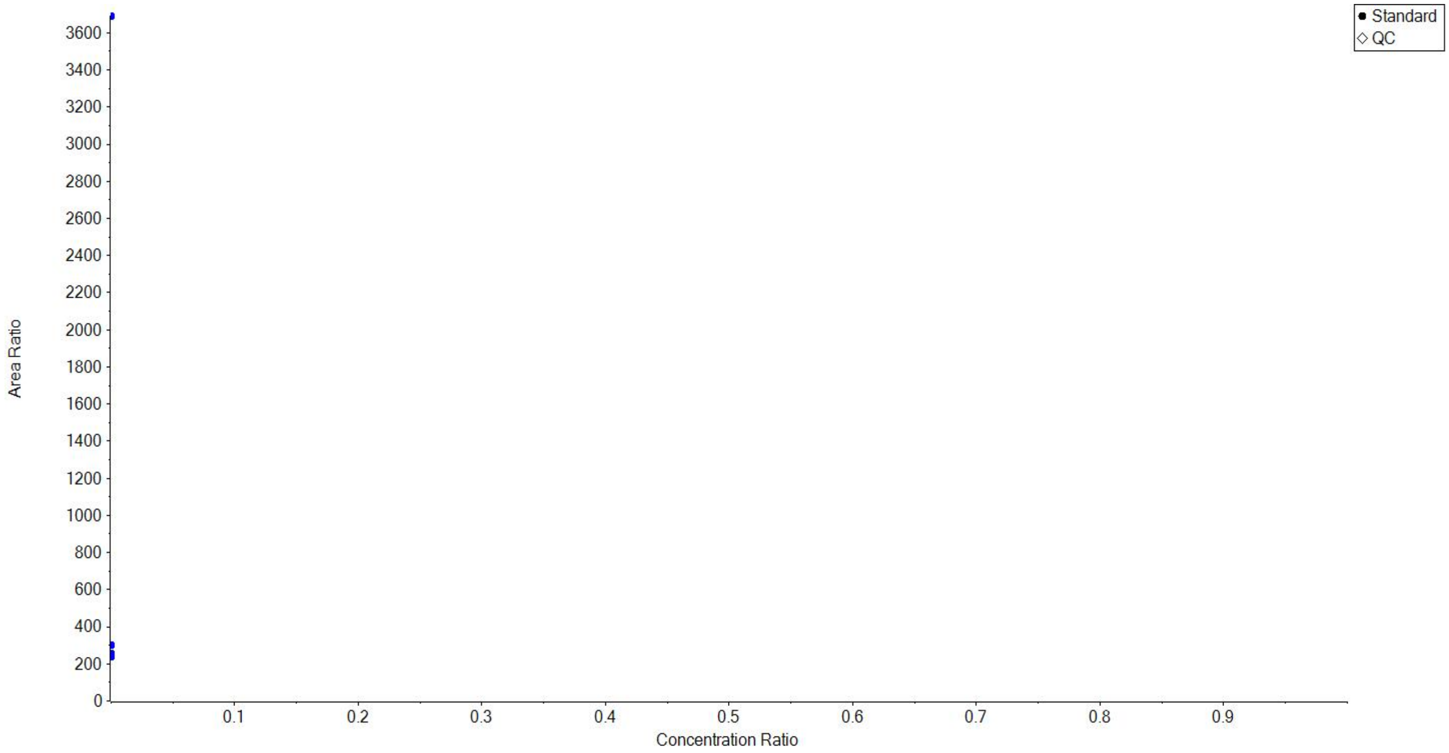

Analyte Name: LM-flavones-39
Internal Standard: LM-flavones-IS02_1

**Data File**	flavones-STD-20230908.wiff	**Result Table**	DZLM2023082419-results-20230913-5500
**Acquisition Date**	9/8/2023 5:37:08 PM	**Algorithm Used**	MQ4
**Acquisition Method**	20230908-flavones-(mix130-T3)-15min.dam	**Instrument Name**	QTRAP 6500+ Low Mass
**Project**	N/A	**Processing Method**	20230412-flavones-(mix130-T3)-15min.qmethod

Regression Equation: y = 0.00781 x + -0.00279 (r = 0.99960, r² = 0.99920) (weighting: 1 / x)

Expected Concentration	Number of Values	Mean Calculated Concentration (No data for Analyte Unit)	% Accuracy	Std. Deviation	%CV
0.01	0 of 1	N/A	N/A	N/A	N/A
0.02	0 of 1	N/A	N/A	N/A	N/A
0.05	0 of 1	N/A	N/A	N/A	N/A
0.13	0 of 1	N/A	N/A	N/A	N/A
0.33	0 of 1	N/A	N/A	N/A	N/A
0.82	0 of 1	N/A	N/A	N/A	N/A
2.05	1 of 1	2.242e0	109.5	N/A	N/A
5.12	1 of 1	4.876e0	95.2	N/A	N/A
12.80	1 of 1	1.178e1	92.0	N/A	N/A
32.00	1 of 1	3.353e1	104.8	N/A	N/A
80.00	1 of 1	7.828e1	97.9	N/A	N/A
200.00	1 of 1	2.013e2	100.6	N/A	N/A

Analyte Name: LM-flavones-39

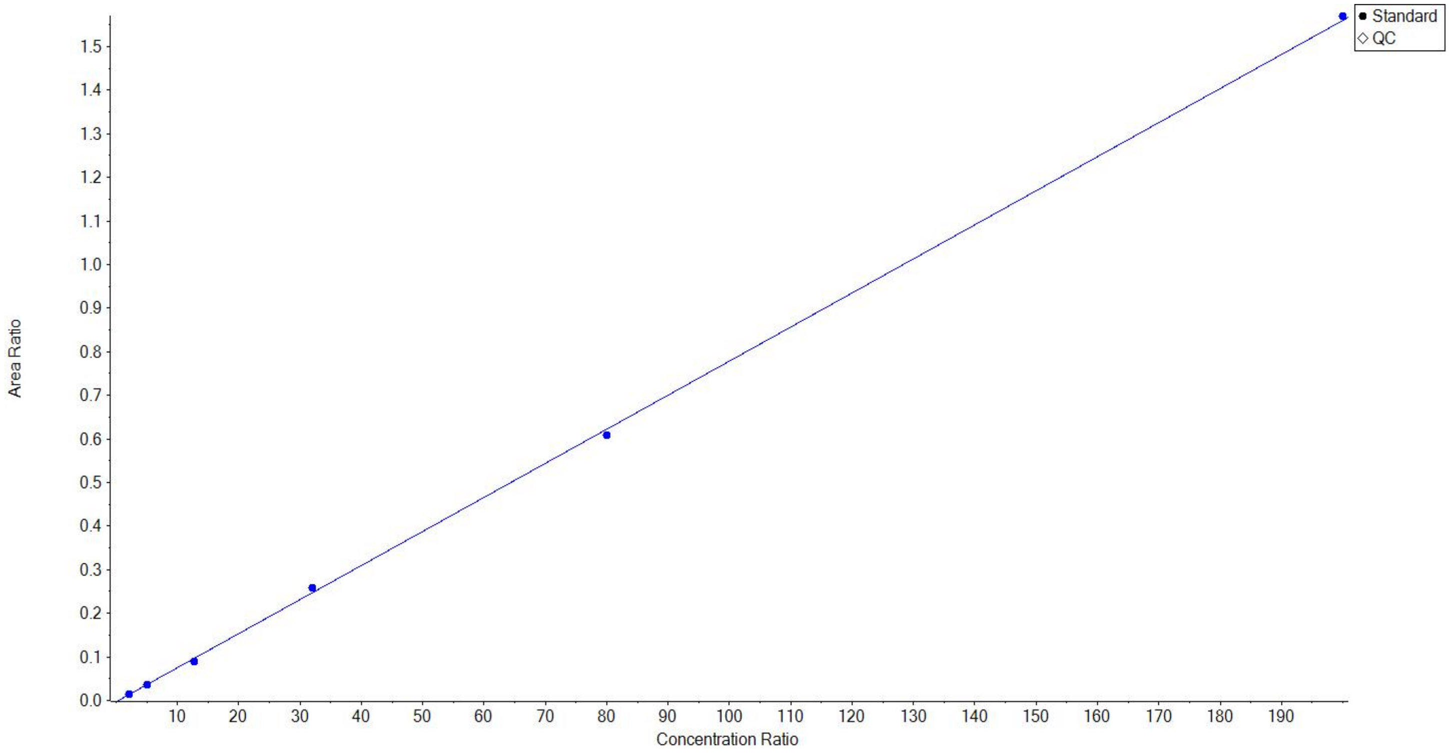
Standard
QC
Area Ratio
Concentration Ratio
0.0
0.1
0.2
0.3
0.4
0.5
0.6
0.7
0.8
0.9
1.0
1.1
1.2
1.3
1.4
1.5
10
20
30
40
50
60
70
80
90
100
110
120
130
140
150
160
170
180
190

Analyte Name: LM-flavones-40_1
Internal Standard: LM-flavones-IS02_1

**Data File**	flavones-STD-20230908.wiff	**Result Table**	DZLM2023082419-results-20230913-5500
**Acquisition Date**	9/8/2023 5:37:08 PM	**Algorithm Used**	MQ4
**Acquisition Method**	20230908-flavones-(mix130-T3)-15min.dam	**Instrument Name**	QTRAP 6500+ Low Mass
**Project**	N/A	**Processing Method**	20230412-flavones-(mix130-T3)-15min.qmethod

Regression Equation: y = 0.00137 x + -4.20966e-4 (r = 0.99953, r² = 0.99906) (weighting: 1 / x)

Expected Concentration	Number of Values	Mean Calculated Concentration (No data for Analyte Unit)	% Accuracy	Std. Deviation	%CV
0.01	0 of 1	N/A	N/A	N/A	N/A
0.02	0 of 1	N/A	N/A	N/A	N/A
0.05	0 of 1	N/A	N/A	N/A	N/A
0.13	0 of 1	N/A	N/A	N/A	N/A
0.33	0 of 1	N/A	N/A	N/A	N/A
0.82	0 of 1	N/A	N/A	N/A	N/A
2.05	1 of 1	2.349e0	114.6	N/A	N/A
5.12	1 of 1	5.028e0	98.2	N/A	N/A
12.80	1 of 1	1.144e1	89.4	N/A	N/A
32.00	1 of 1	3.130e1	97.8	N/A	N/A
80.00	1 of 1	7.879e1	98.5	N/A	N/A
200.00	1 of 1	2.031e2	101.5	N/A	N/A

Analyte Name: LM-flavones-40_1

Created with SCIEX OS 1.6.1
Printed:9/18/2023 5:27:01 PM

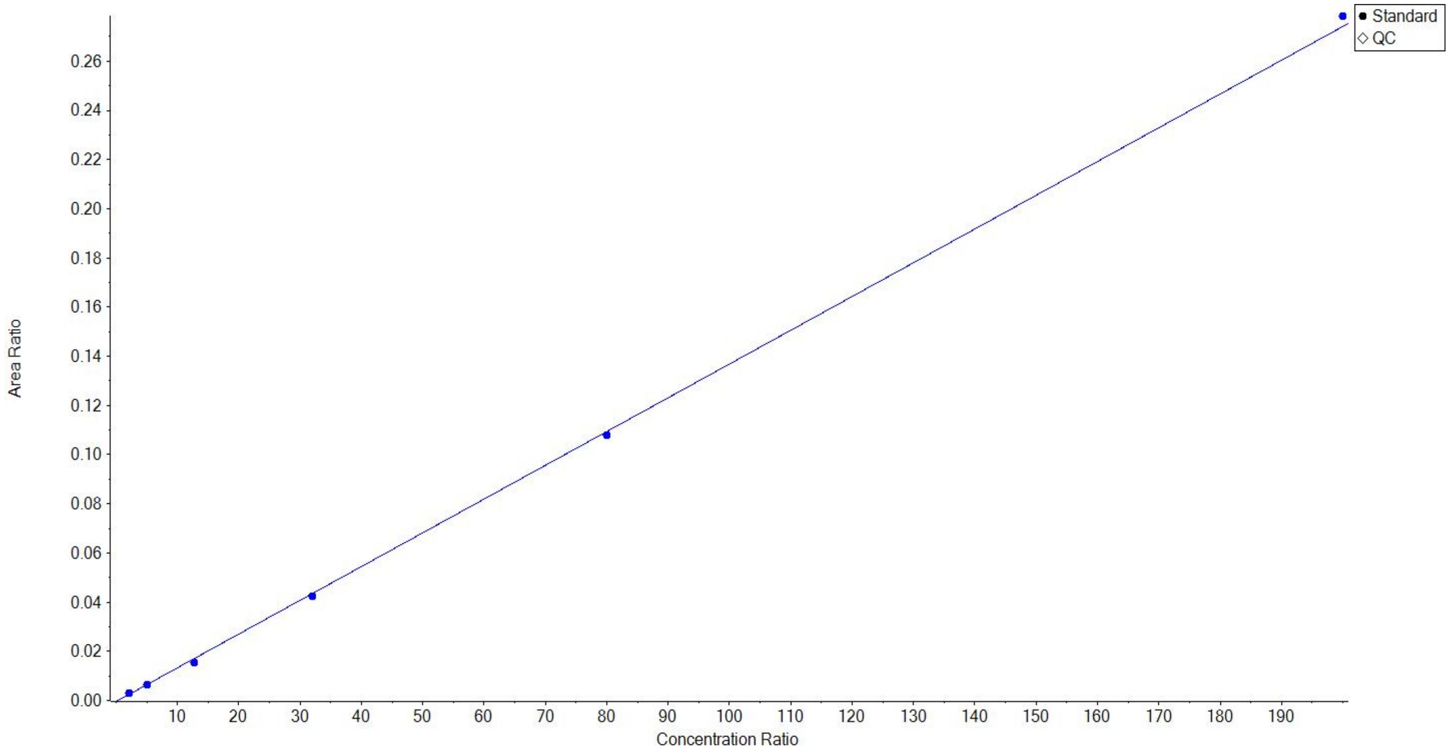

Analyte Name: LM-flavones-05_1
Internal Standard: LM-flavones-IS02_1

**Data File**	flavones-STD-20230908.wiff	**Result Table**	DZLM2023082419-results-20230913-5500
**Acquisition Date**	9/8/2023 5:37:08 PM	**Algorithm Used**	MQ4
**Acquisition Method**	20230908-flavones-(mix130-T3)-15min.dam	**Instrument Name**	QTRAP 6500+ Low Mass
**Project**	N/A	**Processing Method**	20230412-flavones-(mix130-T3)-15min.qmethod

Regression Equation: y = 4.50323e-4 x + 3.37893e-4 (r = 0.99913, r^2 = 0.99826) (weighting: 1 / x)

Expected Concentration	Number of Values	Mean Calculated Concentration (No data for Analyte Unit)	% Accuracy	Std. Deviation	%CV
0.01	0 of 1	N/A	N/A	N/A	N/A
0.02	0 of 1	N/A	N/A	N/A	N/A
0.05	0 of 1	N/A	N/A	N/A	N/A
0.13	0 of 1	N/A	N/A	N/A	N/A
0.33	0 of 1	N/A	N/A	N/A	N/A
0.82	0 of 1	N/A	N/A	N/A	N/A
2.05	1 of 1	1.716e0	83.7	N/A	N/A
5.12	1 of 1	5.339e0	104.3	N/A	N/A
12.80	1 of 1	1.370e1	107.0	N/A	N/A
32.00	1 of 1	3.489e1	109.0	N/A	N/A
80.00	1 of 1	7.705e1	96.3	N/A	N/A
200.00	1 of 1	1.993e2	99.6	N/A	N/A

Analyte Name: LM-flavones-05_1

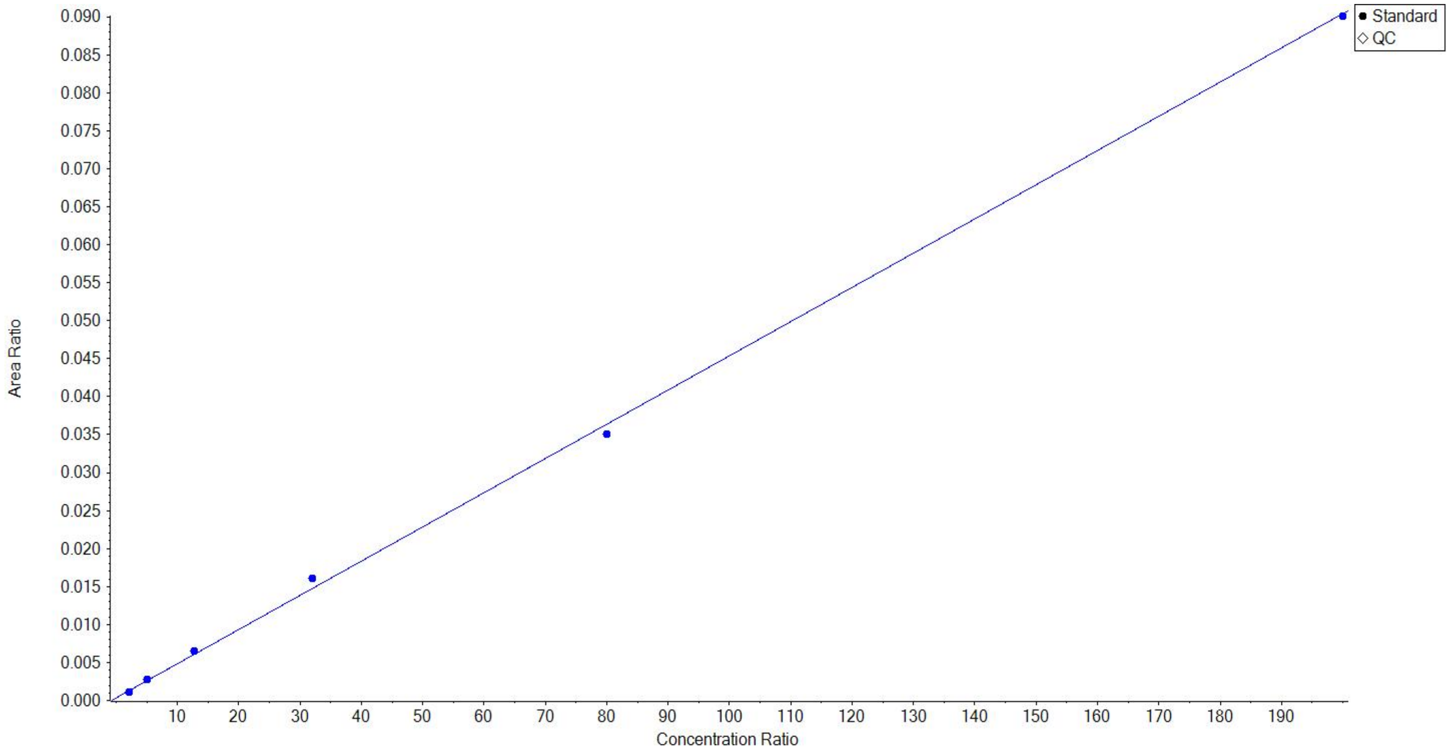
Standard
QC
Area Ratio
Concentration Ratio
0.090
0.085
0.080
0.075
0.070
0.065
0.060
0.055
0.050
0.045
0.040
0.035
0.030
0.025
0.020
0.015
0.010
0.005
0.000
10 20 30 40 50 60 70 80 90 100 110 120 130 140 150 160 170 180 190

Analyte Name: LM-flavones-42
Internal Standard: LM-flavones-IS02_1

**Data File**	flavones-STD-20230908.wiff	**Result Table**	DZLM2023082419-results-20230913-5500
**Acquisition Date**	9/8/2023 5:37:08 PM	**Algorithm Used**	MQ4
**Acquisition Method**	20230908-flavones-(mix130-T3)-15min.dam	**Instrument Name**	QTRAP 6500+ Low Mass
**Project**	N/A	**Processing Method**	20230412-flavones-(mix130-T3)-15min.qmethod

Regression Equation: y = 0.00451 x + 0.00128 (r = 0.99956, r² = 0.99912) (weighting: 1 / x)

Expected Concentration	Number of Values	Mean Calculated Concentration (No data for Analyte Unit)	% Accuracy	Std. Deviation	%CV
0.01	0 of 1	N/A	N/A	N/A	N/A
0.02	0 of 1	N/A	N/A	N/A	N/A
0.05	0 of 1	N/A	N/A	N/A	N/A
0.13	0 of 1	N/A	N/A	N/A	N/A
0.33	0 of 1	N/A	N/A	N/A	N/A
0.82	0 of 1	N/A	N/A	N/A	N/A
2.05	1 of 1	1.991e0	97.2	N/A	N/A
5.12	1 of 1	4.891e0	95.5	N/A	N/A
12.80	1 of 1	1.281e1	100.1	N/A	N/A
32.00	1 of 1	3.434e1	107.3	N/A	N/A
80.00	1 of 1	8.120e1	101.5	N/A	N/A
200.00	1 of 1	1.967e2	98.4	N/A	N/A

Analyte Name: LM-flavones-42

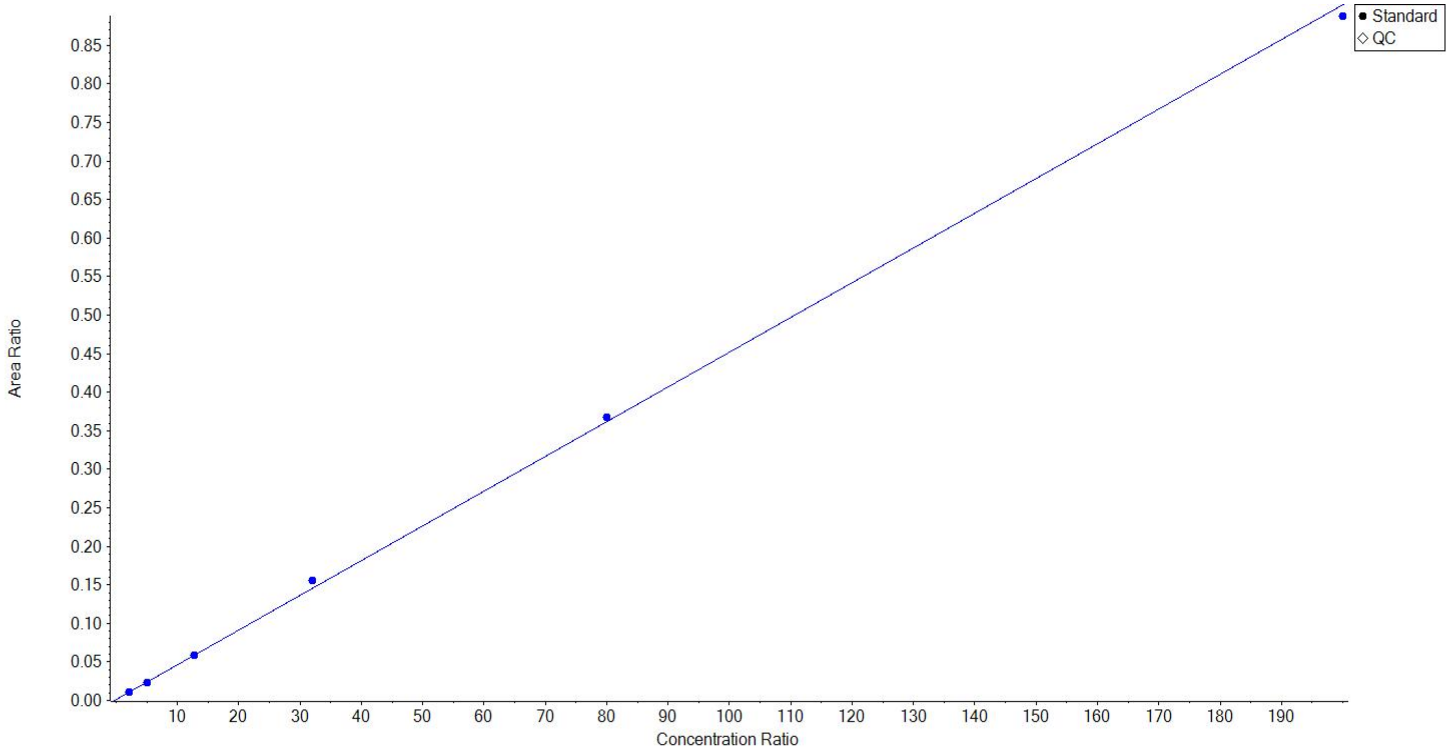
Standard
QC
Area Ratio
Concentration Ratio
0.00
0.05
0.10
0.15
0.20
0.25
0.30
0.35
0.40
0.45
0.50
0.55
0.60
0.65
0.70
0.75
0.80
0.85
10
20
30
40
50
60
70
80
90
100
110
120
130
140
150
160
170
180
190

Analyte Name: LM-flavones-43_1
Internal Standard: LM-flavones-IS02_1

**Data File**	flavones-STD-20230908.wiff	**Result Table**	DZLM2023082419-results-20230913-5500
**Acquisition Date**	9/8/2023 5:37:08 PM	**Algorithm Used**	MQ4
**Acquisition Method**	20230908-flavones-(mix130-T3)-15min.dam	**Instrument Name**	QTRAP 6500+ Low Mass
**Project**	N/A	**Processing Method**	20230412-flavones-(mix130-T3)-15min.qmethod

Regression Equation: y = 0.00167 x + -9.90110e-4 (r = 0.99878, r^2 = 0.99757) (weighting: 1 / x)

Expected Concentration	Number of Values	Mean Calculated Concentration (No data for Analyte Unit)	% Accuracy	Std. Deviation	%CV
0.01	0 of 1	N/A	N/A	N/A	N/A
0.02	0 of 1	N/A	N/A	N/A	N/A
0.05	0 of 1	N/A	N/A	N/A	N/A
0.13	0 of 1	N/A	N/A	N/A	N/A
0.33	0 of 1	N/A	N/A	N/A	N/A
0.82	0 of 1	N/A	N/A	N/A	N/A
2.05	1 of 1	2.424e0	118.3	N/A	N/A
5.12	1 of 1	4.956e0	96.8	N/A	N/A
12.80	1 of 1	1.171e1	91.5	N/A	N/A
32.00	1 of 1	3.063e1	95.7	N/A	N/A
80.00	1 of 1	7.553e1	94.4	N/A	N/A
200.00	1 of 1	2.067e2	103.4	N/A	N/A

Analyte Name: LM-flavones-43_1

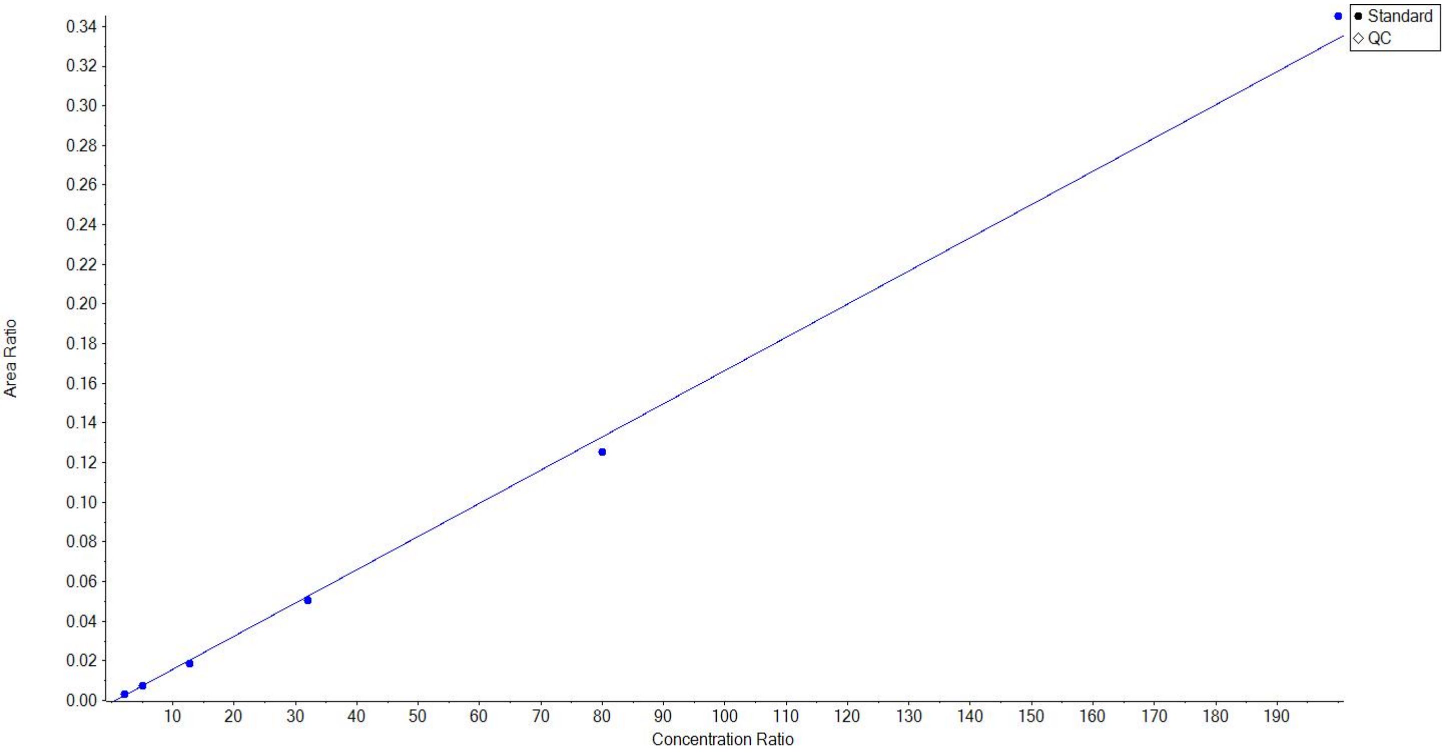
Standard
QC
Area Ratio
0.34
0.32
0.30
0.28
0.26
0.24
0.22
0.20
0.18
0.16
0.14
0.12
0.10
0.08
0.06
0.04
0.02
0.00
10
20
30
40
50
60
70
80
90
100
110
120
130
140
150
160
170
180
190
Concentration Ratio

Analyte Name: LM-flavones-45_1
Internal Standard: LM-flavones-IS02_1

**Data File**	flavones-STD-20230908.wiff	**Result Table**	DZLM2023082419-results-20230913-5500
**Acquisition Date**	9/8/2023 5:37:08 PM	**Algorithm Used**	MQ4
**Acquisition Method**	20230908-flavones-(mix130-T3)-15min.dam	**Instrument Name**	QTRAP 6500+ Low Mass
**Project**	N/A	**Processing Method**	20230412-flavones-(mix130-T3)-15min.qmethod

Regression Equation: y = 0.00242 x + -9.06996e-5 (r = 0.99967, r² = 0.99933) (weighting: 1 / x)

Expected Concentration	Number of Values	Mean Calculated Concentration (No data for Analyte Unit)	% Accuracy	Std. Deviation	%CV
0.01	0 of 1	N/A	N/A	N/A	N/A
0.02	0 of 1	N/A	N/A	N/A	N/A
0.05	0 of 1	N/A	N/A	N/A	N/A
0.13	0 of 1	N/A	N/A	N/A	N/A
0.33	0 of 1	N/A	N/A	N/A	N/A
0.82	0 of 1	N/A	N/A	N/A	N/A
2.05	1 of 1	2.192e0	106.9	N/A	N/A
5.12	1 of 1	4.764e0	93.1	N/A	N/A
12.80	1 of 1	1.222e1	95.5	N/A	N/A
32.00	1 of 1	3.391e1	106.0	N/A	N/A
80.00	1 of 1	7.881e1	98.5	N/A	N/A
200.00	1 of 1	2.001e2	100.0	N/A	N/A

Analyte Name: LM-flavones-45_1

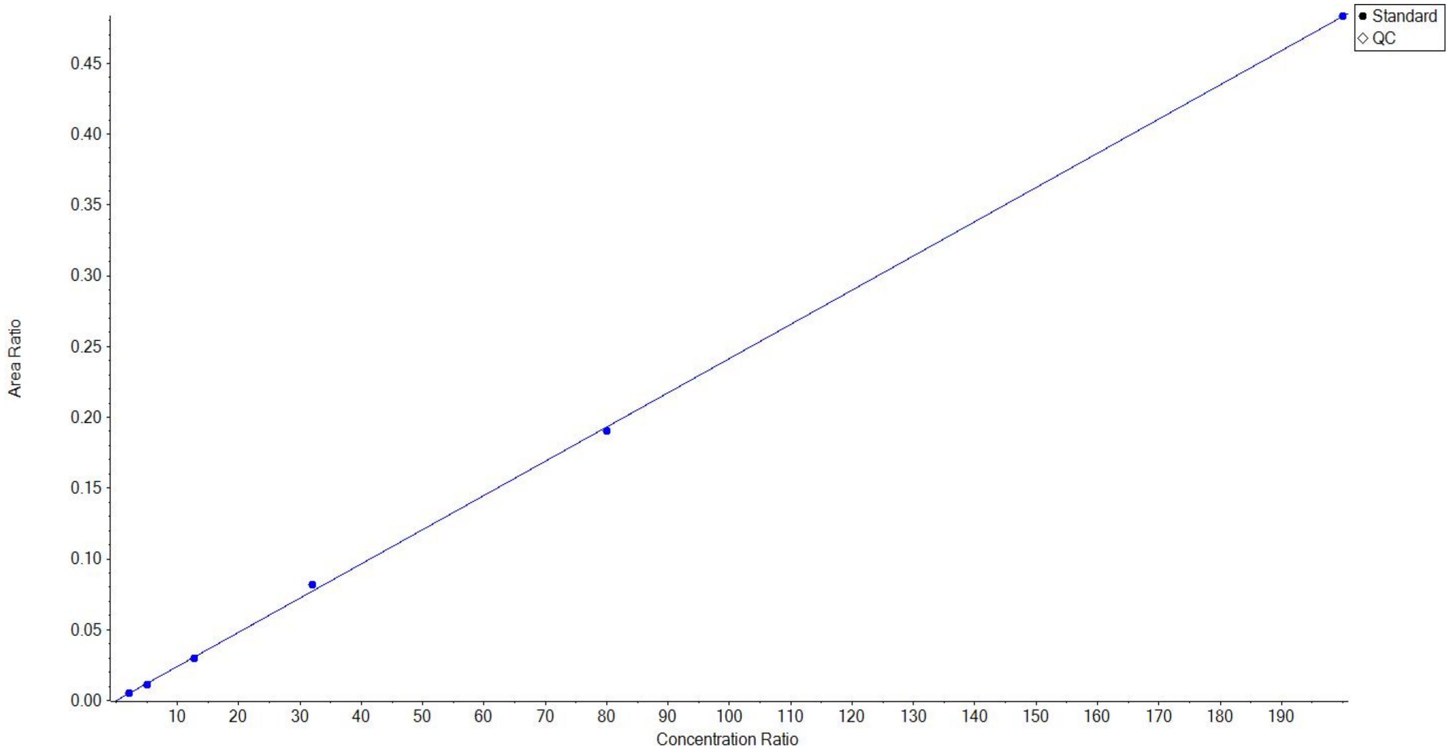
Standard
QC
Area Ratio
0.00
0.05
0.10
0.15
0.20
0.25
0.30
0.35
0.40
0.45
10
20
30
40
50
60
70
80
90
100
110
120
130
140
150
160
170
180
190
Concentration Ratio

Analyte Name: LM-flavones-46_1
Internal Standard: LM-flavones-IS02_1

**Data File**	flavones-STD-20230908.wiff	**Result Table**	DZLM2023082419-results-20230913-5500
**Acquisition Date**	9/8/2023 5:37:08 PM	**Algorithm Used**	MQ4
**Acquisition Method**	20230908-flavones-(mix130-T3)-15min.dam	**Instrument Name**	QTRAP 6500+ Low Mass
**Project**	N/A	**Processing Method**	20230412-flavones-(mix130-T3)-15min.qmethod

Regression Equation: y = 0.00156 x + 4.44771e-4 (r = 0.99970, r^2 = 0.99941) (weighting: 1 / x)

Expected Concentration	Number of Values	Mean Calculated Concentration (No data for Analyte Unit)	% Accuracy	Std. Deviation	%CV
0.01	0 of 1	N/A	N/A	N/A	N/A
0.02	0 of 1	N/A	N/A	N/A	N/A
0.05	0 of 1	N/A	N/A	N/A	N/A
0.13	0 of 1	N/A	N/A	N/A	N/A
0.33	0 of 1	N/A	N/A	N/A	N/A
0.82	0 of 1	N/A	N/A	N/A	N/A
2.05	1 of 1	2.021e0	98.6	N/A	N/A
5.12	1 of 1	5.319e0	103.9	N/A	N/A
12.80	1 of 1	1.190e1	93.0	N/A	N/A
32.00	1 of 1	3.375e1	105.5	N/A	N/A
80.00	1 of 1	7.948e1	99.4	N/A	N/A
200.00	1 of 1	1.995e2	99.8	N/A	N/A

Analyte Name: LM-flavones-46_1

Created with SCIEX OS 1.6.1
Printed:9/18/2023 5:27:01 PM

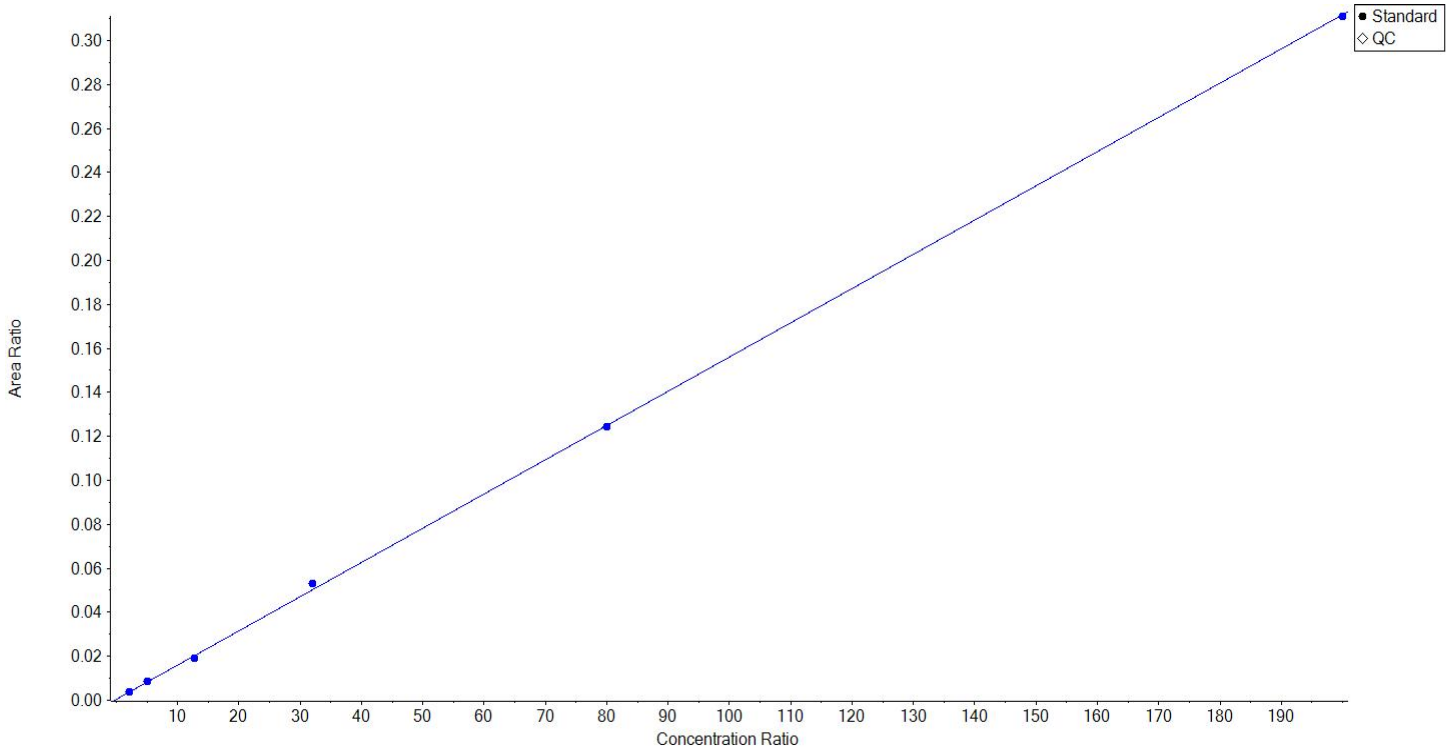

Analyte Name: LM-flavones-52_1
Internal Standard: LM-flavones-IS02_1

**Data File**	flavones-STD-20230908.wiff	**Result Table**	DZLM2023082419-results-20230913-5500
**Acquisition Date**	9/8/2023 5:37:08 PM	**Algorithm Used**	MQ4
**Acquisition Method**	20230908-flavones-(mix130-T3)-15min.dam	**Instrument Name**	QTRAP 6500+ Low Mass
**Project**	N/A	**Processing Method**	20230412-flavones-(mix130-T3)-15min.qmethod

Regression Equation: y = 8.71317e-4 x + 4.61786e-4 (r = 0.99869, r² = 0.99738) (weighting: 1 / x)

Expected Concentration	Number of Values	Mean Calculated Concentration (No data for Analyte Unit)	% Accuracy	Std. Deviation	%CV
0.01	0 of 1	N/A	N/A	N/A	N/A
0.02	0 of 1	N/A	N/A	N/A	N/A
0.05	0 of 1	N/A	N/A	N/A	N/A
0.13	0 of 1	N/A	N/A	N/A	N/A
0.33	0 of 1	N/A	N/A	N/A	N/A
0.82	0 of 1	N/A	N/A	N/A	N/A
2.05	1 of 1	1.489e0	72.6	N/A	N/A
5.12	1 of 1	5.688e0	111.1	N/A	N/A
12.80	1 of 1	1.379e1	107.7	N/A	N/A
32.00	1 of 1	3.510e1	109.7	N/A	N/A
80.00	1 of 1	8.124e1	101.6	N/A	N/A
200.00	1 of 1	1.947e2	97.3	N/A	N/A

Analyte Name: LM-flavones-52_1

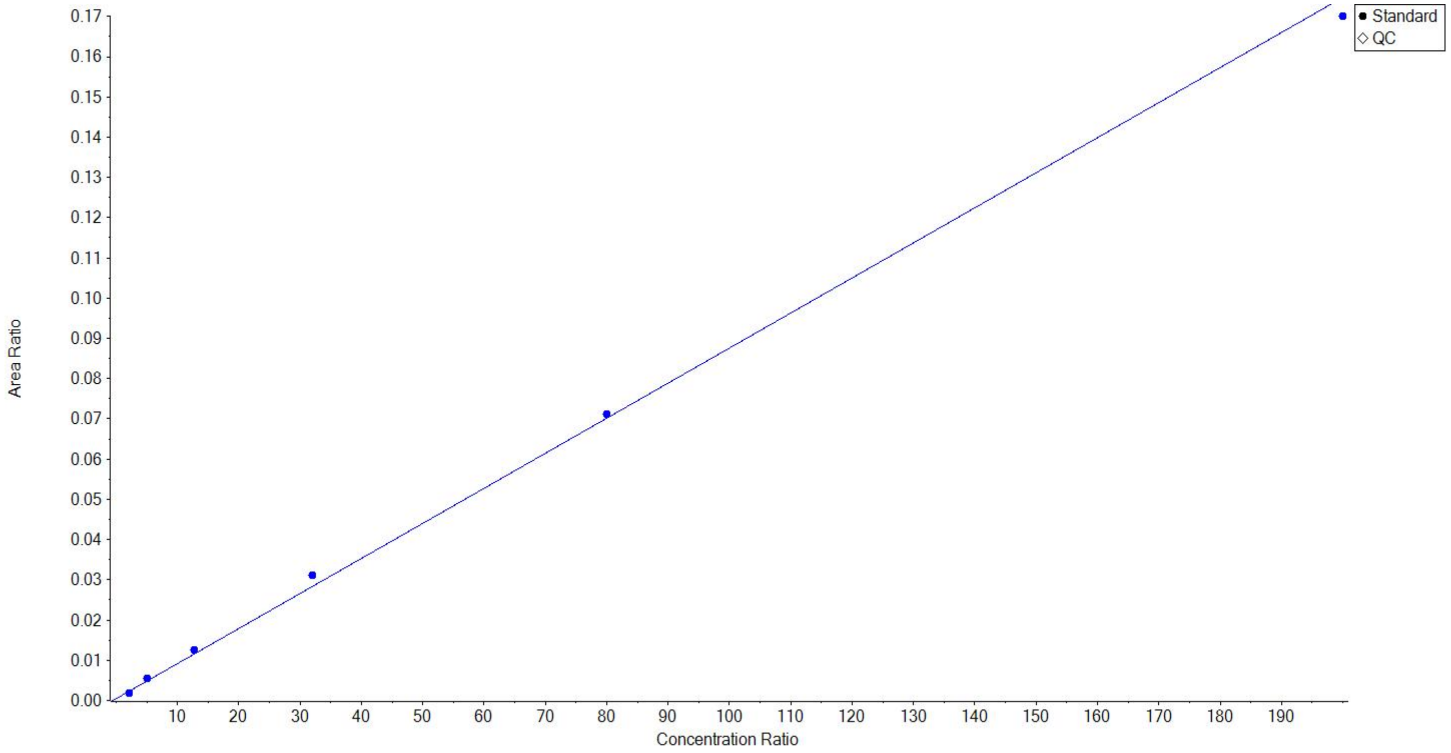
0.17
0.16
0.15
0.14
0.13
0.12
0.11
0.10
0.09
0.08
0.07
0.06
0.05
0.04
0.03
0.02
0.01
0.00
Area Ratio
10
20
30
40
50
60
70
80
90
100
110
120
130
140
150
160
170
180
190
Concentration Ratio
Standard
QC

Analyte Name: LM-flavones-54_1
Internal Standard: LM-flavones-IS02_1

**Data File**	flavones-STD-20230908.wiff	**Result Table**	DZLM2023082419-results-20230913-5500
**Acquisition Date**	9/8/2023 5:37:08 PM	**Algorithm Used**	MQ4
**Acquisition Method**	20230908-flavones-(mix130-T3)-15min.dam	**Instrument Name**	QTRAP 6500+ Low Mass
**Project**	N/A	**Processing Method**	20230412-flavones-(mix130-T3)-15min.qmethod

Regression Equation: y = 0.00311 x + 4.44576e-4 (r = 0.99986, r² = 0.99972) (weighting: 1 / x)

Expected Concentration	Number of Values	Mean Calculated Concentration (No data for Analyte Unit)	% Accuracy	Std. Deviation	%CV
0.01	0 of 1	N/A	N/A	N/A	N/A
0.02	0 of 1	N/A	N/A	N/A	N/A
0.05	0 of 1	N/A	N/A	N/A	N/A
0.13	0 of 1	N/A	N/A	N/A	N/A
0.33	0 of 1	N/A	N/A	N/A	N/A
0.82	0 of 1	N/A	N/A	N/A	N/A
2.05	1 of 1	1.957e0	95.5	N/A	N/A
5.12	1 of 1	5.077e0	99.2	N/A	N/A
12.80	1 of 1	1.302e1	101.7	N/A	N/A
32.00	1 of 1	3.337e1	104.3	N/A	N/A
80.00	1 of 1	8.014e1	100.2	N/A	N/A
200.00	1 of 1	1.984e2	99.2	N/A	N/A

Analyte Name: LM-flavones-54_1

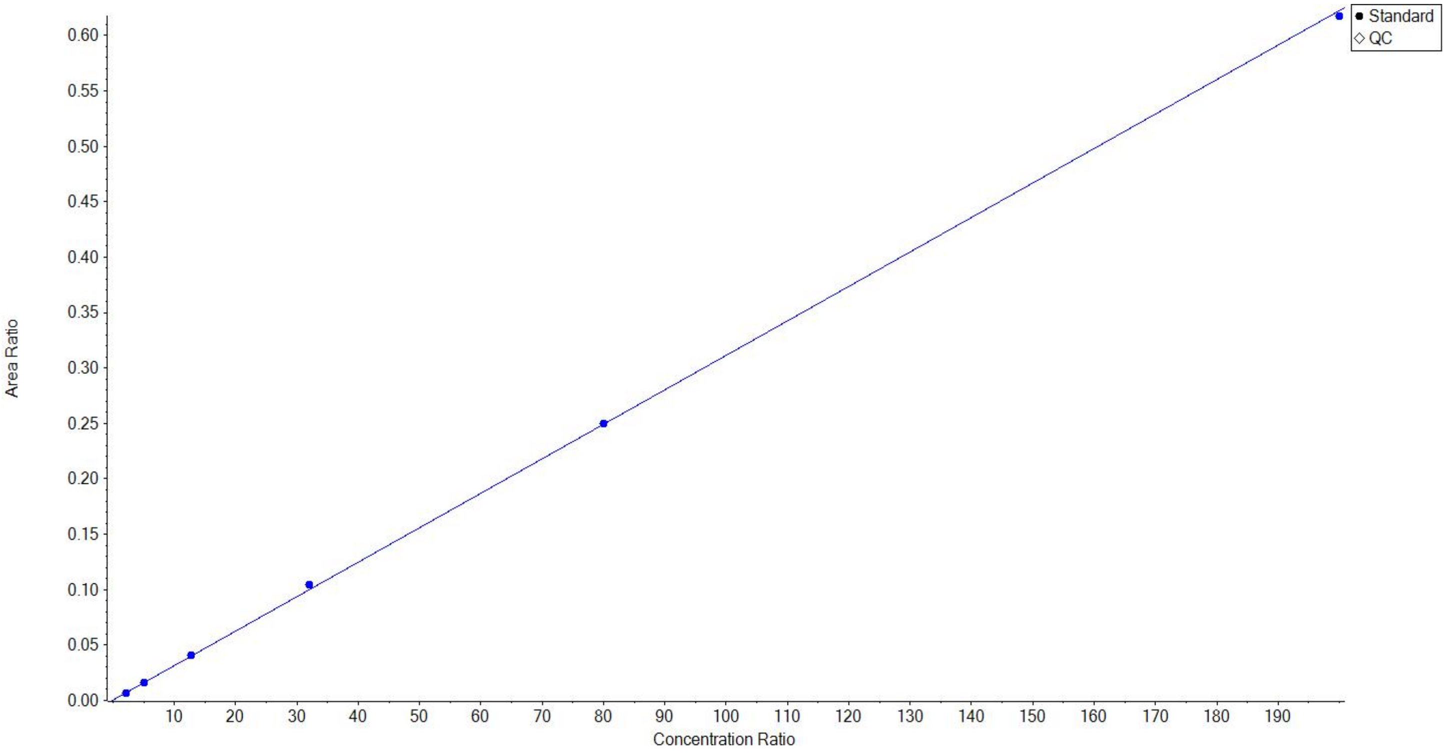

Standard
QC
Area Ratio
Concentration Ratio
0.00
0.05
0.10
0.15
0.20
0.25
0.30
0.35
0.40
0.45
0.50
0.55
0.60
10
20
30
40
50
60
70
80
90
100
110
120
130
140
150
160
170
180
190

Analyte Name: LM-flavones-55_2
Internal Standard: LM-flavones-IS02_1

**Data File**	flavones-STD-20230908.wiff	**Result Table**	DZLM2023082419-results-20230913-5500
**Acquisition Date**	9/8/2023 5:37:08 PM	**Algorithm Used**	MQ4
**Acquisition Method**	20230908-flavones-(mix130-T3)-15min.dam	**Instrument Name**	QTRAP 6500+ Low Mass
**Project**	N/A	**Processing Method**	20230412-flavones-(mix130-T3)-15min.qmethod

Regression Equation: y = 1.53882e-4 x + -4.77133e-5 (r = 0.99506, r² = 0.99015) (weighting: 1 / x)

Expected Concentration	Number of Values	Mean Calculated Concentration (No data for Analyte Unit)	% Accuracy	Std. Deviation	%CV
0.01	0 of 1	N/A	N/A	N/A	N/A
0.02	0 of 1	N/A	N/A	N/A	N/A
0.05	0 of 1	N/A	N/A	N/A	N/A
0.13	0 of 1	N/A	N/A	N/A	N/A
0.33	0 of 1	N/A	N/A	N/A	N/A
0.82	0 of 1	N/A	N/A	N/A	N/A
2.05	1 of 1	2.414e0	117.8	N/A	N/A
5.12	1 of 1	5.538e0	108.2	N/A	N/A
12.80	1 of 1	1.177e1	92.0	N/A	N/A
32.00	1 of 1	2.790e1	87.2	N/A	N/A
80.00	1 of 1	7.032e1	87.9	N/A	N/A
200.00	1 of 1	2.140e2	107.0	N/A	N/A

Analyte Name: LM-flavones-55_2

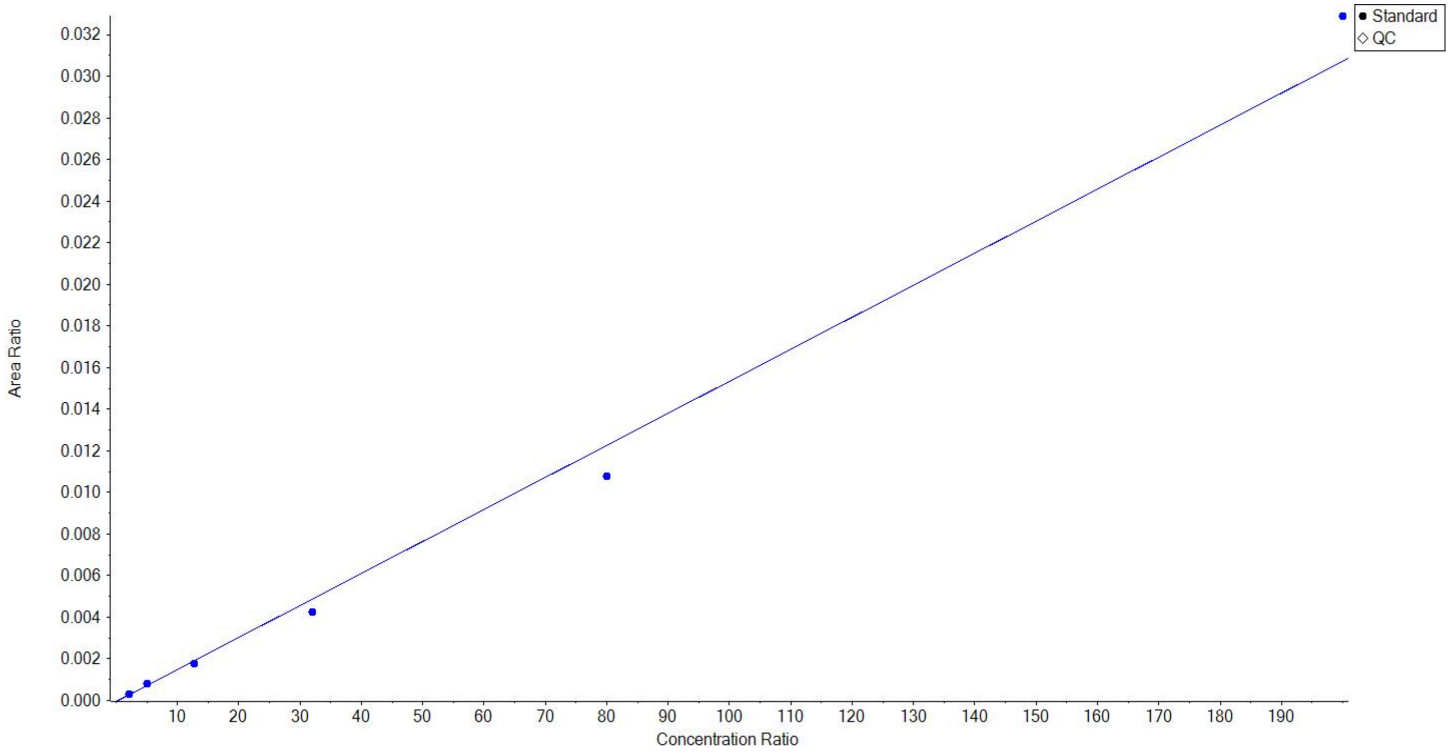
0.032
0.030
0.028
0.026
0.024
0.022
0.020
0.018
0.016
0.014
0.012
0.010
0.008
0.006
0.004
0.002
0.000
Area Ratio
10
20
30
40
50
60
70
80
90
100
110
120
130
140
150
160
170
180
190
Concentration Ratio
Standard
QC

Analyte Name: LM-flavones-56_1
Internal Standard: LM-flavones-IS02_1

**Data File**	flavones-STD-20230908.wiff	**Result Table**	DZLM2023082419-results-20230913-5500
**Acquisition Date**	9/8/2023 5:37:08 PM	**Algorithm Used**	MQ4
**Acquisition Method**	20230908-flavones-(mix130-T3)-15min.dam	**Instrument Name**	QTRAP 6500+ Low Mass
**Project**	N/A	**Processing Method**	20230412-flavones-(mix130-T3)-15min.qmethod

Regression Equation: y = 3.35150e-4 x + 2.43788e-4 (r = 0.99891, r^2 = 0.99783) (weighting: 1 / x)

Expected Concentration	Number of Values	Mean Calculated Concentration (No data for Analyte Unit)	% Accuracy	Std. Deviation	%CV
0.01	0 of 1	N/A	N/A	N/A	N/A
0.02	0 of 1	N/A	N/A	N/A	N/A
0.05	0 of 1	N/A	N/A	N/A	N/A
0.13	0 of 1	N/A	N/A	N/A	N/A
0.33	0 of 1	N/A	N/A	N/A	N/A
0.82	0 of 1	N/A	N/A	N/A	N/A
2.05	1 of 1	1.897e0	92.5	N/A	N/A
5.12	1 of 1	4.731e0	92.4	N/A	N/A
12.80	1 of 1	1.340e1	104.7	N/A	N/A
32.00	1 of 1	3.542e1	110.7	N/A	N/A
80.00	1 of 1	8.192e1	102.4	N/A	N/A
200.00	1 of 1	1.946e2	97.3	N/A	N/A

Analyte Name: LM-flavones-56_1

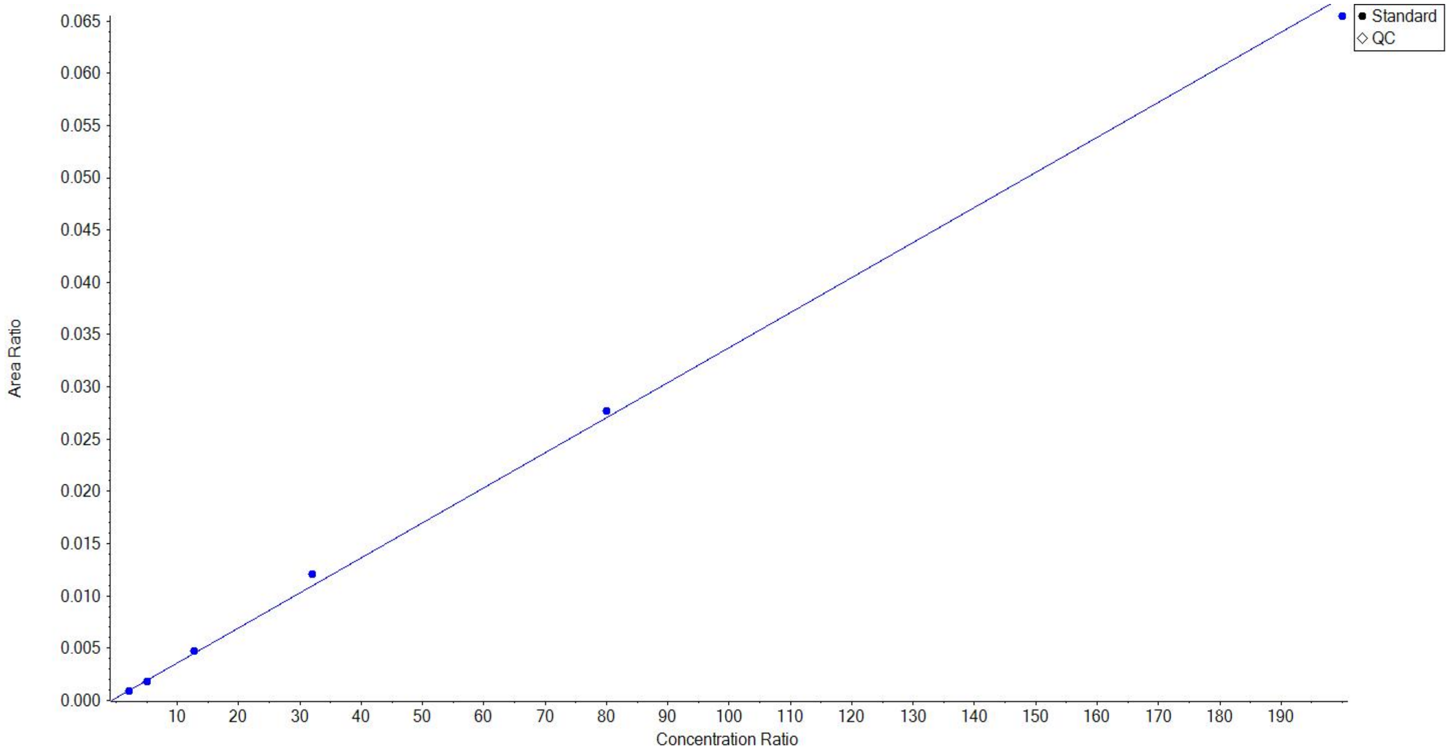
0.065
0.060
0.055
0.050
0.045
0.040
0.035
0.030
0.025
0.020
0.015
0.010
0.005
0.000
Area Ratio
10 20 30 40 50 60 70 80 90 100 110 120 130 140 150 160 170 180 190
Concentration Ratio
Standard
QC

Analyte Name: LM-flavones-57_1
Internal Standard: LM-flavones-IS02_1

**Data File**	flavones-STD-20230908.wiff	**Result Table**	DZLM2023082419-results-20230913-5500
**Acquisition Date**	9/8/2023 5:37:08 PM	**Algorithm Used**	MQ4
**Acquisition Method**	20230908-flavones-(mix130-T3)-15min.dam	**Instrument Name**	QTRAP 6500+ Low Mass
**Project**	N/A	**Processing Method**	20230412-flavones-(mix130-T3)-15min.qmethod

Regression Equation: y = 4.90655e-4 x + -6.81859e-5 (r = 0.99871, r² = 0.99741) (weighting: 1 / x)

Expected Concentration	Number of Values	Mean Calculated Concentration (No data for Analyte Unit)	% Accuracy	Std. Deviation	%CV
0.01	0 of 1	N/A	N/A	N/A	N/A
0.02	0 of 1	N/A	N/A	N/A	N/A
0.05	0 of 1	N/A	N/A	N/A	N/A
0.13	0 of 1	N/A	N/A	N/A	N/A
0.33	0 of 1	N/A	N/A	N/A	N/A
0.82	0 of 1	N/A	N/A	N/A	N/A
2.05	1 of 1	1.915e0	93.4	N/A	N/A
5.12	1 of 1	5.163e0	100.9	N/A	N/A
12.80	1 of 1	1.245e1	97.3	N/A	N/A
32.00	1 of 1	3.349e1	104.7	N/A	N/A
80.00	1 of 1	8.581e1	107.3	N/A	N/A
200.00	1 of 1	1.931e2	96.6	N/A	N/A

Analyte Name: LM-flavones-57_1

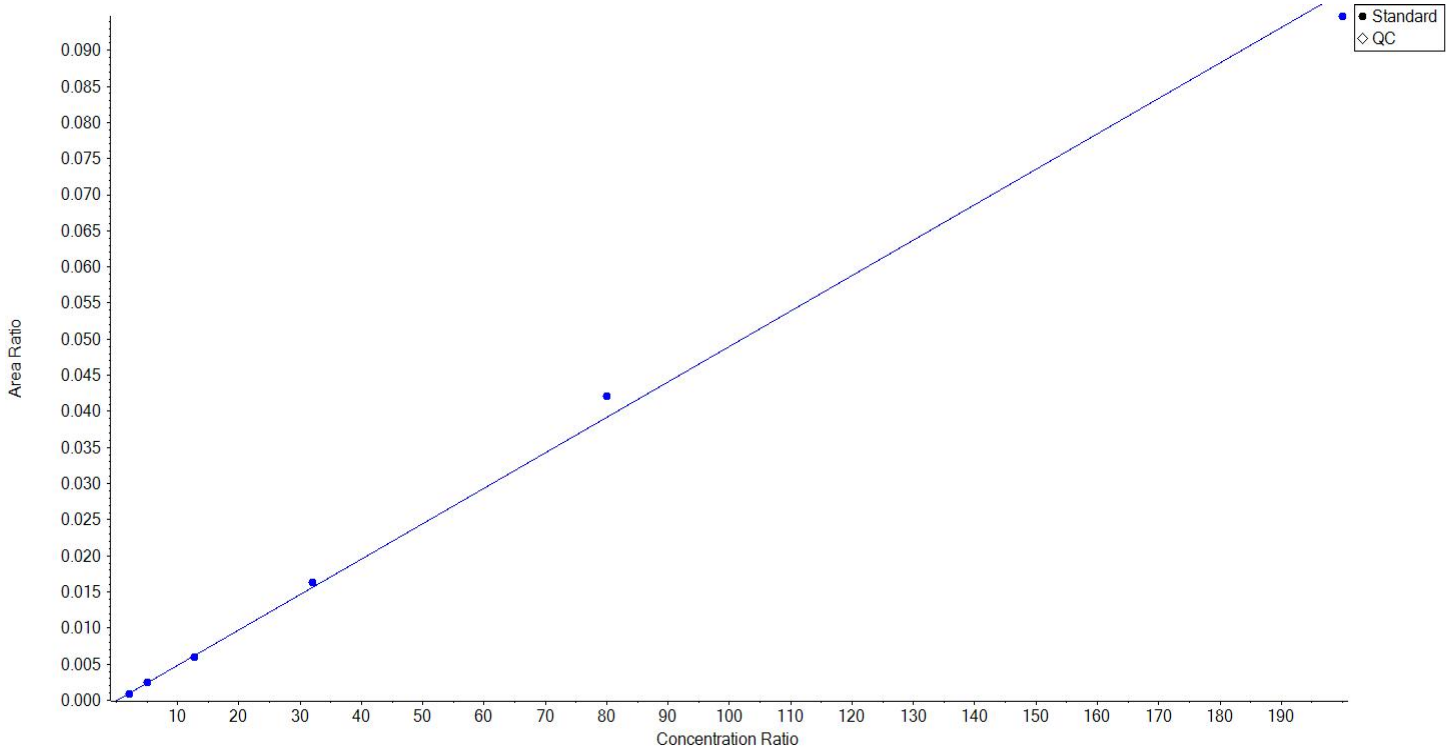
Standard
QC
Area Ratio
0.090
0.085
0.080
0.075
0.070
0.065
0.060
0.055
0.050
0.045
0.040
0.035
0.030
0.025
0.020
0.015
0.010
0.005
0.000
10 20 30 40 50 60 70 80 90 100 110 120 130 140 150 160 170 180 190
Concentration Ratio

Analyte Name: LM-flavones-58_1
Internal Standard: LM-flavones-IS02_1

**Data File**	flavones-STD-20230908.wiff	**Result Table**	DZLM2023082419-results-20230913-5500
**Acquisition Date**	9/8/2023 5:37:08 PM	**Algorithm Used**	MQ4
**Acquisition Method**	20230908-flavones-(mix130-T3)-15min.dam	**Instrument Name**	QTRAP 6500+ Low Mass
**Project**	N/A	**Processing Method**	20230412-flavones-(mix130-T3)-15min.qmethod

Regression Equation: y = 0.00613 x + -0.00148 (r = 0.99980, r² = 0.99960) (weighting: 1 / x)

Expected Concentration	Number of Values	Mean Calculated Concentration (No data for Analyte Unit)	% Accuracy	Std. Deviation	%CV
0.01	0 of 1	N/A	N/A	N/A	N/A
0.02	0 of 1	N/A	N/A	N/A	N/A
0.05	0 of 1	N/A	N/A	N/A	N/A
0.13	0 of 1	N/A	N/A	N/A	N/A
0.33	0 of 1	N/A	N/A	N/A	N/A
0.82	0 of 1	N/A	N/A	N/A	N/A
2.05	1 of 1	2.182e0	106.4	N/A	N/A
5.12	1 of 1	5.157e0	100.7	N/A	N/A
12.80	1 of 1	1.214e1	94.8	N/A	N/A
32.00	1 of 1	3.165e1	98.9	N/A	N/A
80.00	1 of 1	7.829e1	97.9	N/A	N/A
200.00	1 of 1	2.026e2	101.3	N/A	N/A

Analyte Name: LM-flavones-58_1

Created with SCIEX OS 1.6.1
Printed:9/18/2023 5:27:01 PM

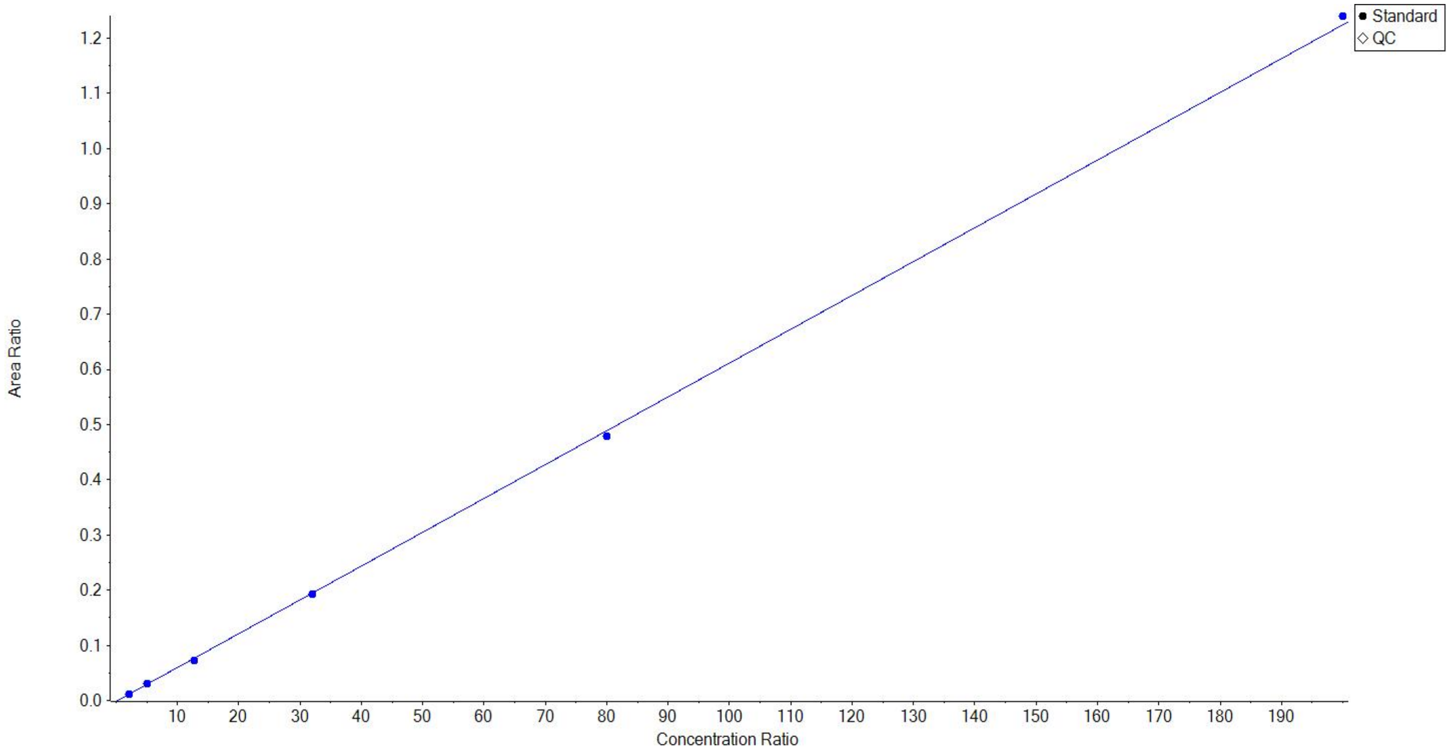

Analyte Name: LM-flavones-59_1
Internal Standard: LM-flavones-IS02_1

**Data File**	flavones-STD-20230908.wiff	**Result Table**	DZLM2023082419-results-20230913-5500
**Acquisition Date**	9/8/2023 5:37:08 PM	**Algorithm Used**	MQ4
**Acquisition Method**	20230908-flavones-(mix130-T3)-15min.dam	**Instrument Name**	QTRAP 6500+ Low Mass
**Project**	N/A	**Processing Method**	20230412-flavones-(mix130-T3)-15min.qmethod

Regression Equation: y = 0.00178 x + 1.36721e-4 (r = 0.99891, r² = 0.99783) (weighting: 1 / x)

Expected Concentration	Number of Values	Mean Calculated Concentration (No data for Analyte Unit)	% Accuracy	Std. Deviation	%CV
0.01	0 of 1	N/A	N/A	N/A	N/A
0.02	0 of 1	N/A	N/A	N/A	N/A
0.05	0 of 1	N/A	N/A	N/A	N/A
0.13	0 of 1	N/A	N/A	N/A	N/A
0.33	0 of 1	N/A	N/A	N/A	N/A
0.82	0 of 1	N/A	N/A	N/A	N/A
2.05	1 of 1	2.219e0	108.3	N/A	N/A
5.12	1 of 1	5.187e0	101.3	N/A	N/A
12.80	1 of 1	1.180e1	92.2	N/A	N/A
32.00	1 of 1	3.055e1	95.5	N/A	N/A
80.00	1 of 1	8.222e1	102.8	N/A	N/A
200.00	0 of 1	N/A	N/A	N/A	N/A

Analyte Name: LM-flavones-59_1

Created with SCIEX OS 1.6.1
Printed:9/18/2023 5:27:01 PM

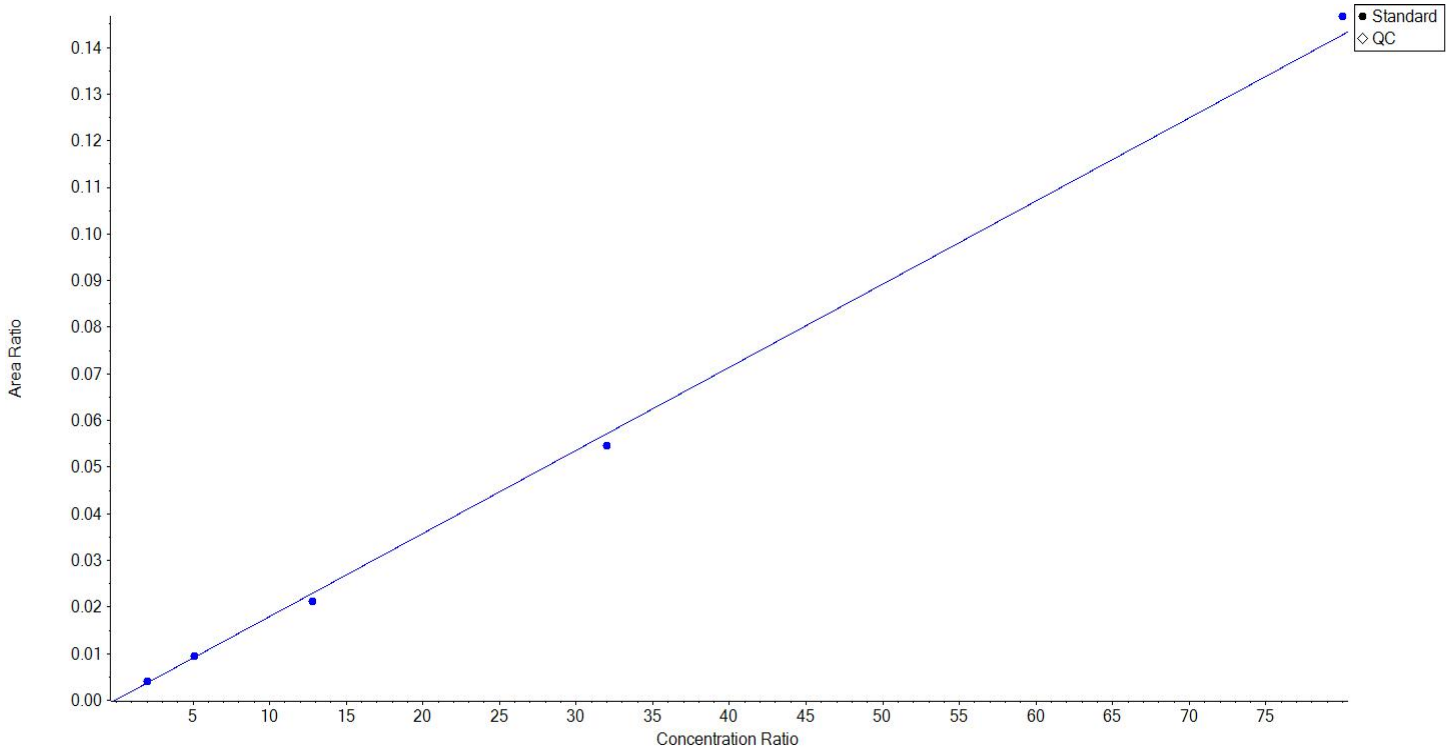

Analyte Name: LM-flavones-60_1
Internal Standard: LM-flavones-IS02_1

**Data File**	flavones-STD-20230908.wiff	**Result Table**	DZLM2023082419-results-20230913-5500
**Acquisition Date**	9/8/2023 5:37:08 PM	**Algorithm Used**	MQ4
**Acquisition Method**	20230908-flavones-(mix130-T3)-15min.dam	**Instrument Name**	QTRAP 6500+ Low Mass
**Project**	N/A	**Processing Method**	20230412-flavones-(mix130-T3)-15min.qmethod

Regression Equation: y = 0.01939 x + -0.00665 (r = 0.99967, r² = 0.99935) (weighting: 1 / x)

Expected Concentration	Number of Values	Mean Calculated Concentration (No data for Analyte Unit)	% Accuracy	Std. Deviation	%CV
0.01	0 of 1	N/A	N/A	N/A	N/A
0.02	0 of 1	N/A	N/A	N/A	N/A
0.05	0 of 1	N/A	N/A	N/A	N/A
0.13	0 of 1	N/A	N/A	N/A	N/A
0.33	0 of 1	N/A	N/A	N/A	N/A
0.82	0 of 1	N/A	N/A	N/A	N/A
2.05	1 of 1	2.226e0	108.6	N/A	N/A
5.12	1 of 1	4.955e0	96.8	N/A	N/A
12.80	1 of 1	1.231e1	96.1	N/A	N/A
32.00	1 of 1	3.214e1	100.4	N/A	N/A
80.00	1 of 1	7.720e1	96.5	N/A	N/A
200.00	1 of 1	2.031e2	101.6	N/A	N/A

Analyte Name: LM-flavones-60_1

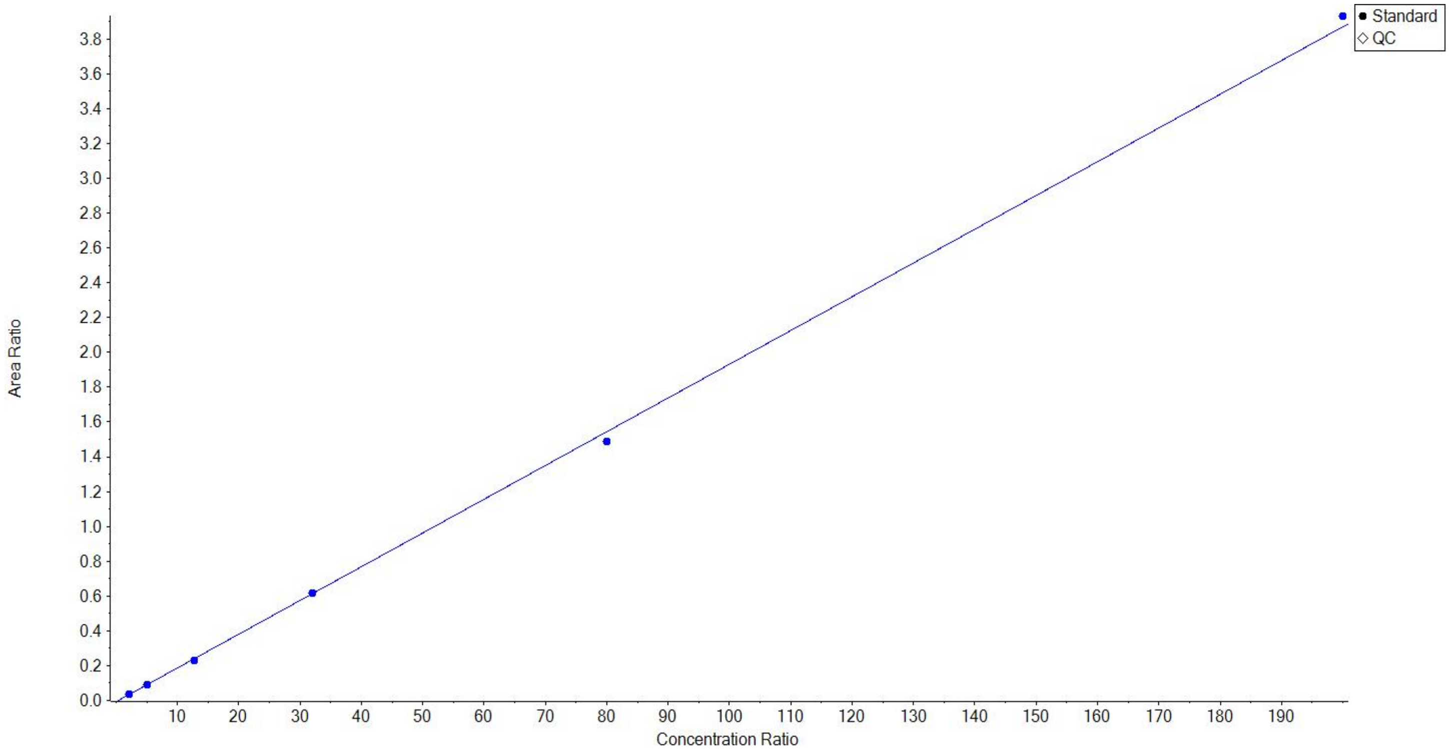
Standard
QC
Area Ratio
Concentration Ratio
0.0
0.2
0.4
0.6
0.8
1.0
1.2
1.4
1.6
1.8
2.0
2.2
2.4
2.6
2.8
3.0
3.2
3.4
3.6
3.8
10
20
30
40
50
60
70
80
90
100
110
120
130
140
150
160
170
180
190

Created with SCIEX OS 1.6.1
Printed:9/18/2023 5:27:01 PM

Analyte Name: LM-flavones-62_1
Internal Standard: LM-flavones-IS02_1

**Data File**	flavones-STD-20230908.wiff	**Result Table**	DZLM2023082419-results-20230913-5500
**Acquisition Date**	9/8/2023 5:37:08 PM	**Algorithm Used**	MQ4
**Acquisition Method**	20230908-flavones-(mix130-T3)-15min.dam	**Instrument Name**	QTRAP 6500+ Low Mass
**Project**	N/A	**Processing Method**	20230412-flavones-(mix130-T3)-15min.qmethod

Regression Equation: y = 0.00122 x + -3.06568e-4 (r = 0.99890, r² = 0.99780) (weighting: 1 / x)

Expected Concentration	Number of Values	Mean Calculated Concentration (No data for Analyte Unit)	% Accuracy	Std. Deviation	%CV
0.01	0 of 1	N/A	N/A	N/A	N/A
0.02	0 of 1	N/A	N/A	N/A	N/A
0.05	0 of 1	N/A	N/A	N/A	N/A
0.13	0 of 1	N/A	N/A	N/A	N/A
0.33	0 of 1	N/A	N/A	N/A	N/A
0.82	0 of 1	N/A	N/A	N/A	N/A
2.05	1 of 1	2.229e0	108.9	N/A	N/A
5.12	1 of 1	5.104e0	99.7	N/A	N/A
12.80	1 of 1	1.176e1	91.9	N/A	N/A
32.00	1 of 1	3.321e1	103.8	N/A	N/A
80.00	1 of 1	7.462e1	93.3	N/A	N/A
200.00	1 of 1	2.050e2	102.5	N/A	N/A

Analyte Name: LM-flavones-62_1

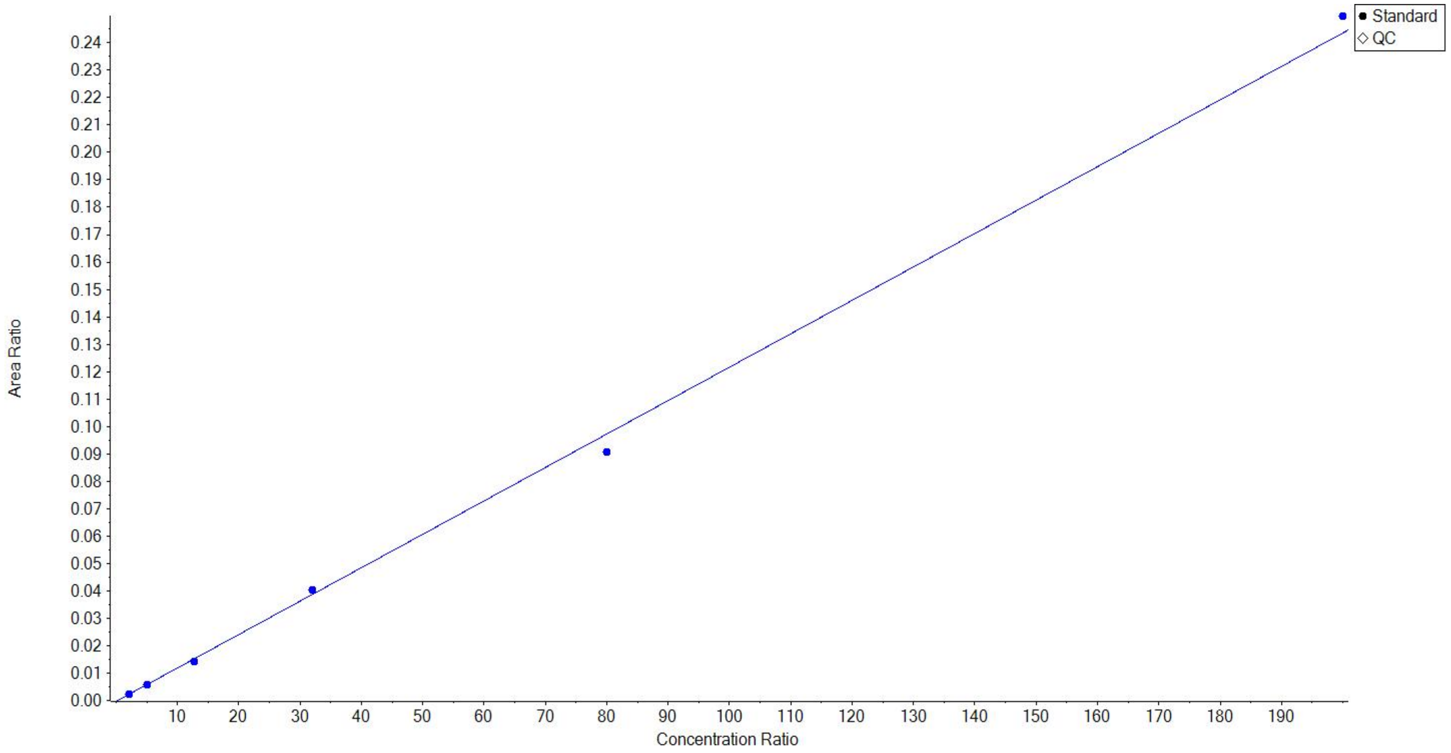
Standard
QC
Area Ratio
0.24
0.23
0.22
0.21
0.20
0.19
0.18
0.17
0.16
0.15
0.14
0.13
0.12
0.11
0.10
0.09
0.08
0.07
0.06
0.05
0.04
0.03
0.02
0.01
0.00
10
20
30
40
50
60
70
80
90
100
110
120
130
140
150
160
170
180
190
Concentration Ratio

Analyte Name: LM-flavones-63_1
Internal Standard: LM-flavones-IS02_1

**Data File**	flavones-STD-20230908.wiff	**Result Table**	DZLM2023082419-results-20230913-5500
**Acquisition Date**	9/8/2023 5:37:08 PM	**Algorithm Used**	MQ4
**Acquisition Method**	20230908-flavones-(mix130-T3)-15min.dam	**Instrument Name**	QTRAP 6500+ Low Mass
**Project**	N/A	**Processing Method**	20230412-flavones-(mix130-T3)-15min.qmethod

Regression Equation: y = 2.34064e-4 x + 7.49575e-5 (r = 0.99926, r² = 0.99852) (weighting: 1 / x)

Expected Concentration	Number of Values	Mean Calculated Concentration (No data for Analyte Unit)	% Accuracy	Std. Deviation	%CV
0.01	0 of 1	N/A	N/A	N/A	N/A
0.02	0 of 1	N/A	N/A	N/A	N/A
0.05	0 of 1	N/A	N/A	N/A	N/A
0.13	0 of 1	N/A	N/A	N/A	N/A
0.33	0 of 1	N/A	N/A	N/A	N/A
0.82	1 of 1	8.537e-1	104.1	N/A	N/A
2.05	1 of 1	2.071e0	101.0	N/A	N/A
5.12	1 of 1	5.043e0	98.5	N/A	N/A
12.80	1 of 1	1.199e1	93.6	N/A	N/A
32.00	1 of 1	3.287e1	102.7	N/A	N/A
80.00	0 of 1	N/A	N/A	N/A	N/A
200.00	0 of 1	N/A	N/A	N/A	N/A

Analyte Name: LM-flavones-63_1

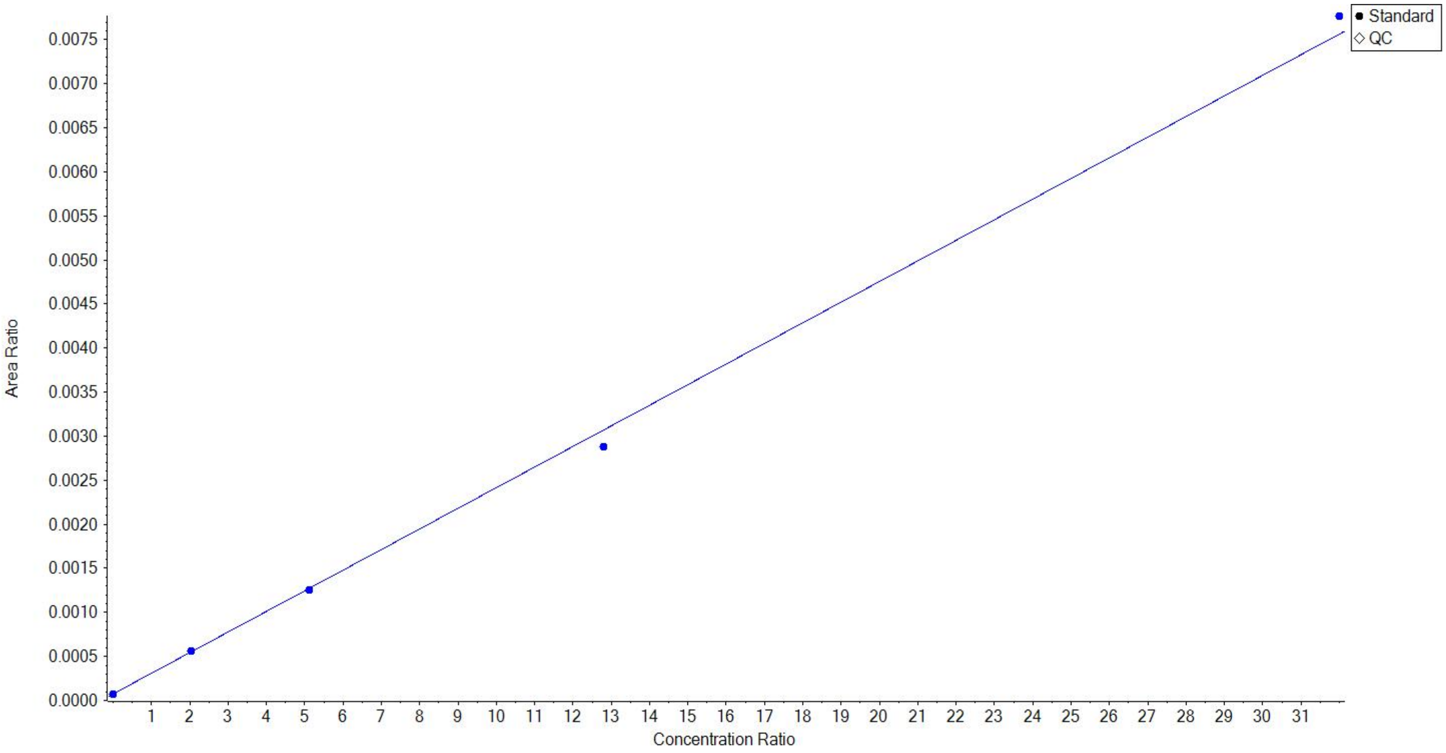
Standard
QC
Area Ratio
0.0000
0.0005
0.0010
0.0015
0.0020
0.0025
0.0030
0.0035
0.0040
0.0045
0.0050
0.0055
0.0060
0.0065
0.0070
0.0075
Concentration Ratio
1
2
3
4
5
6
7
8
9
10
11
12
13
14
15
16
17
18
19
20
21
22
23
24
25
26
27
28
29
30
31

Analyte Name: LM-flavones-64_1
Internal Standard: LM-flavones-IS02_1

**Data File**	flavones-STD-20230908.wiff	**Result Table**	DZLM2023082419-results-20230913-5500
**Acquisition Date**	9/8/2023 5:37:08 PM	**Algorithm Used**	MQ4
**Acquisition Method**	20230908-flavones-(mix130-T3)-15min.dam	**Instrument Name**	QTRAP 6500+ Low Mass
**Project**	N/A	**Processing Method**	20230412-flavones-(mix130-T3)-15min.qmethod

Regression Equation: y = 0.00490 x + -0.00112 (r = 0.99951, r^2 = 0.99903) (weighting: 1 / x)

Expected Concentration	Number of Values	Mean Calculated Concentration (No data for Analyte Unit)	% Accuracy	Std. Deviation	%CV
0.01	0 of 1	N/A	N/A	N/A	N/A
0.02	0 of 1	N/A	N/A	N/A	N/A
0.05	0 of 1	N/A	N/A	N/A	N/A
0.13	0 of 1	N/A	N/A	N/A	N/A
0.33	0 of 1	N/A	N/A	N/A	N/A
0.82	0 of 1	N/A	N/A	N/A	N/A
2.05	1 of 1	2.257e0	110.1	N/A	N/A
5.12	1 of 1	4.929e0	96.3	N/A	N/A
12.80	1 of 1	1.200e1	93.7	N/A	N/A
32.00	1 of 1	3.274e1	102.3	N/A	N/A
80.00	1 of 1	7.677e1	96.0	N/A	N/A
200.00	1 of 1	2.033e2	101.6	N/A	N/A

Analyte Name: LM-flavones-64_1

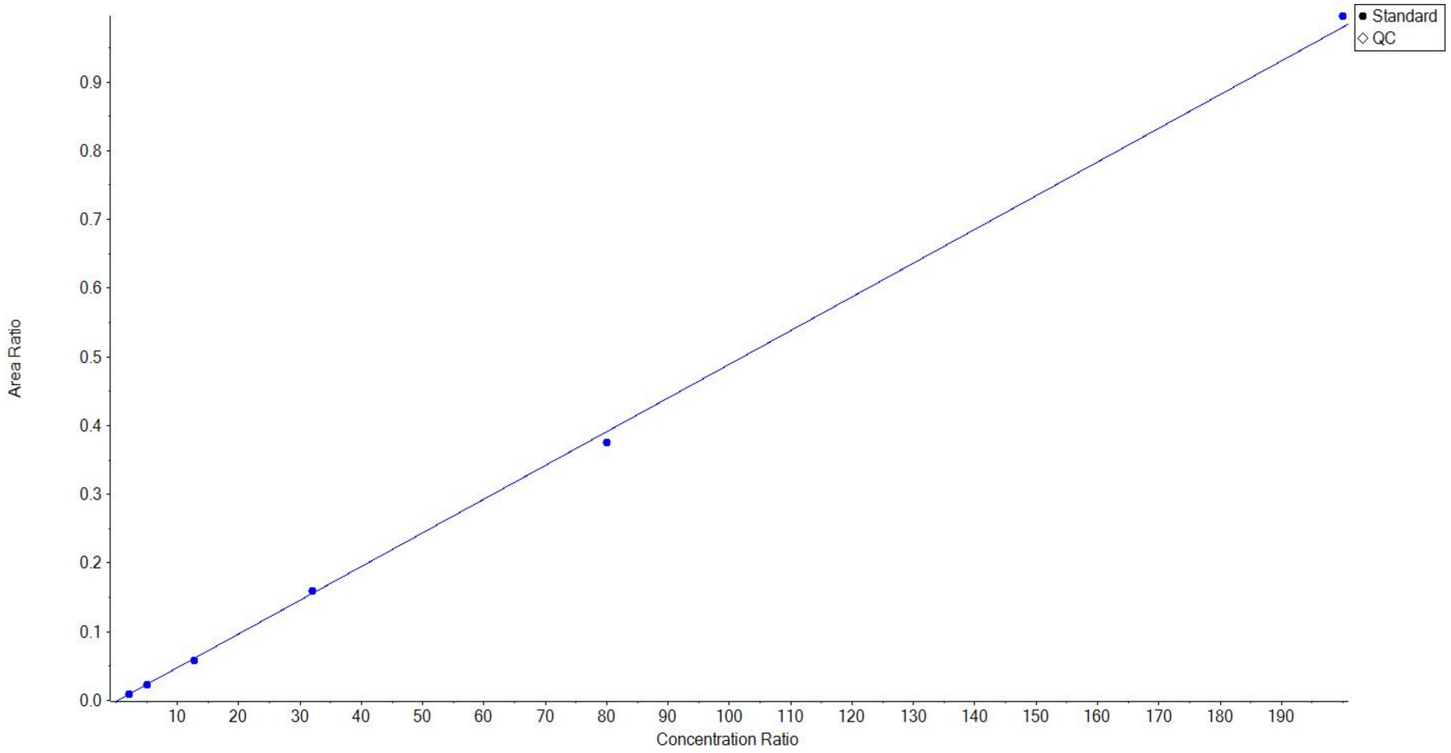
Standard
QC
Area Ratio
0.0
0.1
0.2
0.3
0.4
0.5
0.6
0.7
0.8
0.9
10
20
30
40
50
60
70
80
90
100
110
120
130
140
150
160
170
180
190
Concentration Ratio

Created with SCIEX OS 1.6.1
Printed:9/18/2023 5:27:01 PM

Analyte Name: LM-flavones-66_1
Internal Standard: LM-flavones-IS02_1

**Data File**	flavones-STD-20230908.wiff	**Result Table**	DZLM2023082419-results-20230913-5500
**Acquisition Date**	9/8/2023 5:37:08 PM	**Algorithm Used**	MQ4
**Acquisition Method**	20230908-flavones-(mix130-T3)-15min.dam	**Instrument Name**	QTRAP 6500+ Low Mass
**Project**	N/A	**Processing Method**	20230412-flavones-(mix130-T3)-15min.qmethod

Regression Equation: y = 0.00204 x + 3.69886e-5 (r = 0.99972, r^2 = 0.99945) (weighting: 1 / x)

Expected Concentration	Number of Values	Mean Calculated Concentration (No data for Analyte Unit)	% Accuracy	Std. Deviation	%CV
0.01	0 of 1	N/A	N/A	N/A	N/A
0.02	0 of 1	N/A	N/A	N/A	N/A
0.05	0 of 1	N/A	N/A	N/A	N/A
0.13	0 of 1	N/A	N/A	N/A	N/A
0.33	0 of 1	N/A	N/A	N/A	N/A
0.82	0 of 1	N/A	N/A	N/A	N/A
2.05	1 of 1	2.360e0	115.2	N/A	N/A
5.12	1 of 1	4.572e0	89.3	N/A	N/A
12.80	1 of 1	1.211e1	94.6	N/A	N/A
32.00	1 of 1	3.233e1	101.0	N/A	N/A
80.00	1 of 1	7.938e1	99.2	N/A	N/A
200.00	1 of 1	2.012e2	100.6	N/A	N/A

Analyte Name: LM-flavones-66_1

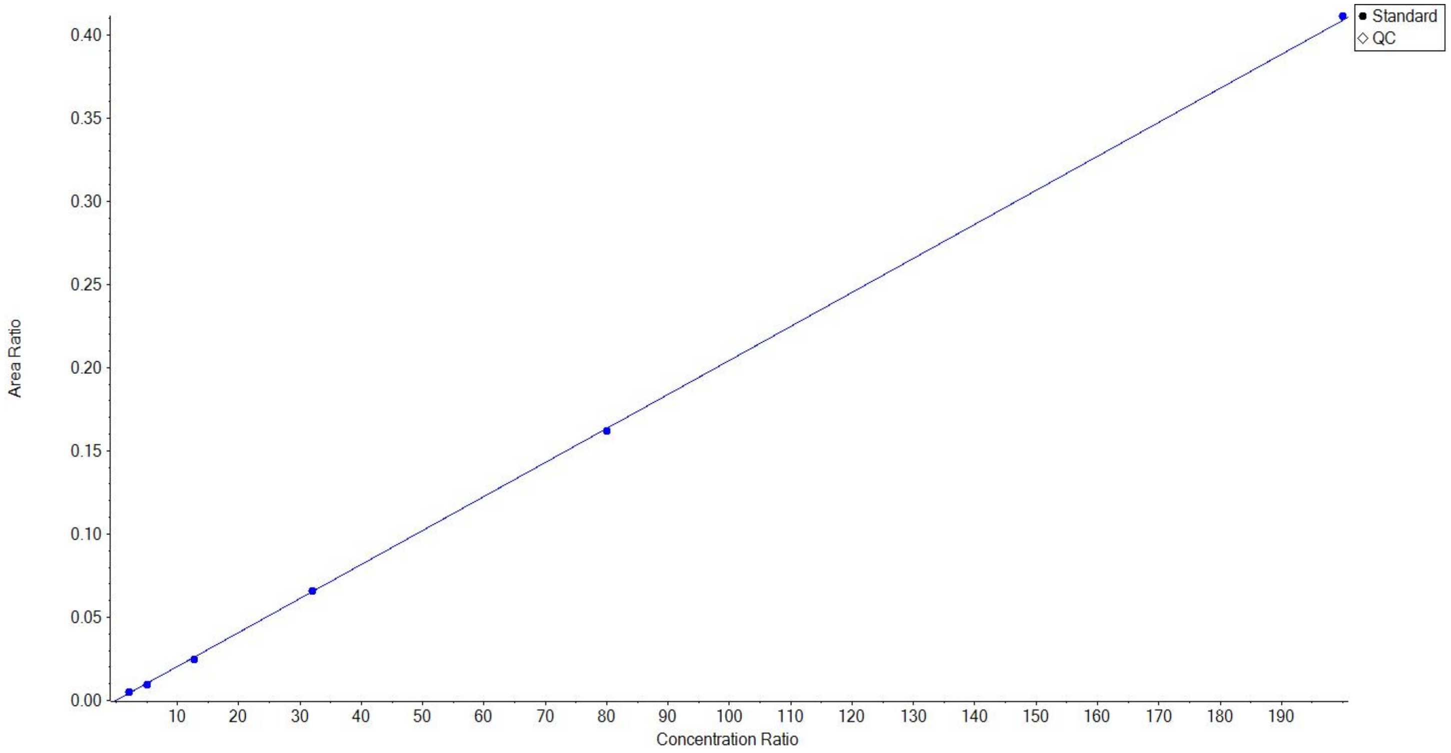
Standard
QC
Area Ratio
0.40
0.35
0.30
0.25
0.20
0.15
0.10
0.05
0.00
10
20
30
40
50
60
70
80
90
100
110
120
130
140
150
160
170
180
190
Concentration Ratio

Analyte Name: LM-flavones-67_1
Internal Standard: LM-flavones-IS02_1

**Data File**	flavones-STD-20230908.wiff	**Result Table**	DZLM2023082419-results-20230913-5500
**Acquisition Date**	9/8/2023 5:37:08 PM	**Algorithm Used**	MQ4
**Acquisition Method**	20230908-flavones-(mix130-T3)-15min.dam	**Instrument Name**	QTRAP 6500+ Low Mass
**Project**	N/A	**Processing Method**	20230412-flavones-(mix130-T3)-15min.qmethod

Regression Equation: y = 0.00486 x + -0.00725 (r = 0.99920, r² = 0.99840) (weighting: 1 / x)

Expected Concentration	Number of Values	Mean Calculated Concentration (No data for Analyte Unit)	% Accuracy	Std. Deviation	%CV
0.01	0 of 1	N/A	N/A	N/A	N/A
0.02	0 of 1	N/A	N/A	N/A	N/A
0.05	0 of 1	N/A	N/A	N/A	N/A
0.13	0 of 1	N/A	N/A	N/A	N/A
0.33	0 of 1	N/A	N/A	N/A	N/A
0.82	0 of 1	N/A	N/A	N/A	N/A
2.05	0 of 1	N/A	N/A	N/A	N/A
5.12	1 of 1	5.718e0	111.7	N/A	N/A
12.80	1 of 1	1.234e1	96.4	N/A	N/A
32.00	1 of 1	2.915e1	91.1	N/A	N/A
80.00	1 of 1	7.933e1	99.2	N/A	N/A
200.00	1 of 1	2.034e2	101.7	N/A	N/A

Analyte Name: LM-flavones-67_1

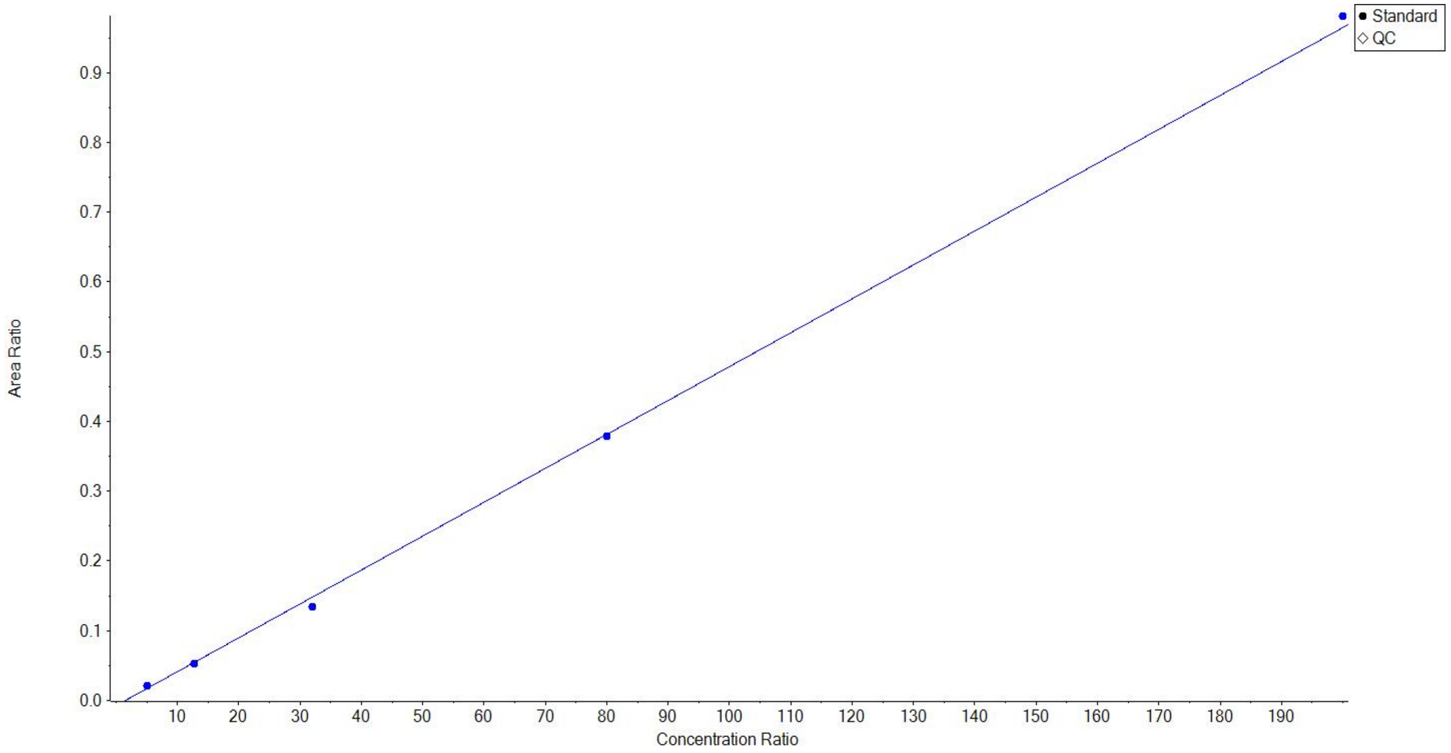
Standard
QC
Area Ratio
0.0
0.1
0.2
0.3
0.4
0.5
0.6
0.7
0.8
0.9
10
20
30
40
50
60
70
80
90
100
110
120
130
140
150
160
170
180
190
Concentration Ratio

Analyte Name: LM-flavones-69_1
Internal Standard: LM-flavones-IS02_1

**Data File**	flavones-STD-20230908.wiff	**Result Table**	DZLM2023082419-results-20230913-5500
**Acquisition Date**	9/8/2023 5:37:08 PM	**Algorithm Used**	MQ4
**Acquisition Method**	20230908-flavones-(mix130-T3)-15min.dam	**Instrument Name**	QTRAP 6500+ Low Mass
**Project**	N/A	**Processing Method**	20230412-flavones-(mix130-T3)-15min.qmethod

Regression Equation: y = 0.00148 x + -1.43968e-4 (r = 0.99909, r^2 = 0.99818) (weighting: 1 / x)

Expected Concentration	Number of Values	Mean Calculated Concentration (No data for Analyte Unit)	% Accuracy	Std. Deviation	%CV
0.01	0 of 1	N/A	N/A	N/A	N/A
0.02	0 of 1	N/A	N/A	N/A	N/A
0.05	0 of 1	N/A	N/A	N/A	N/A
0.13	0 of 1	N/A	N/A	N/A	N/A
0.33	0 of 1	N/A	N/A	N/A	N/A
0.82	0 of 1	N/A	N/A	N/A	N/A
2.05	1 of 1	2.376e0	116.0	N/A	N/A
5.12	1 of 1	4.571e0	89.3	N/A	N/A
12.80	1 of 1	1.117e1	87.2	N/A	N/A
32.00	1 of 1	3.454e1	108.0	N/A	N/A
80.00	1 of 1	7.984e1	99.8	N/A	N/A
200.00	1 of 1	1.995e2	99.7	N/A	N/A

Analyte Name: LM-flavones-69_1

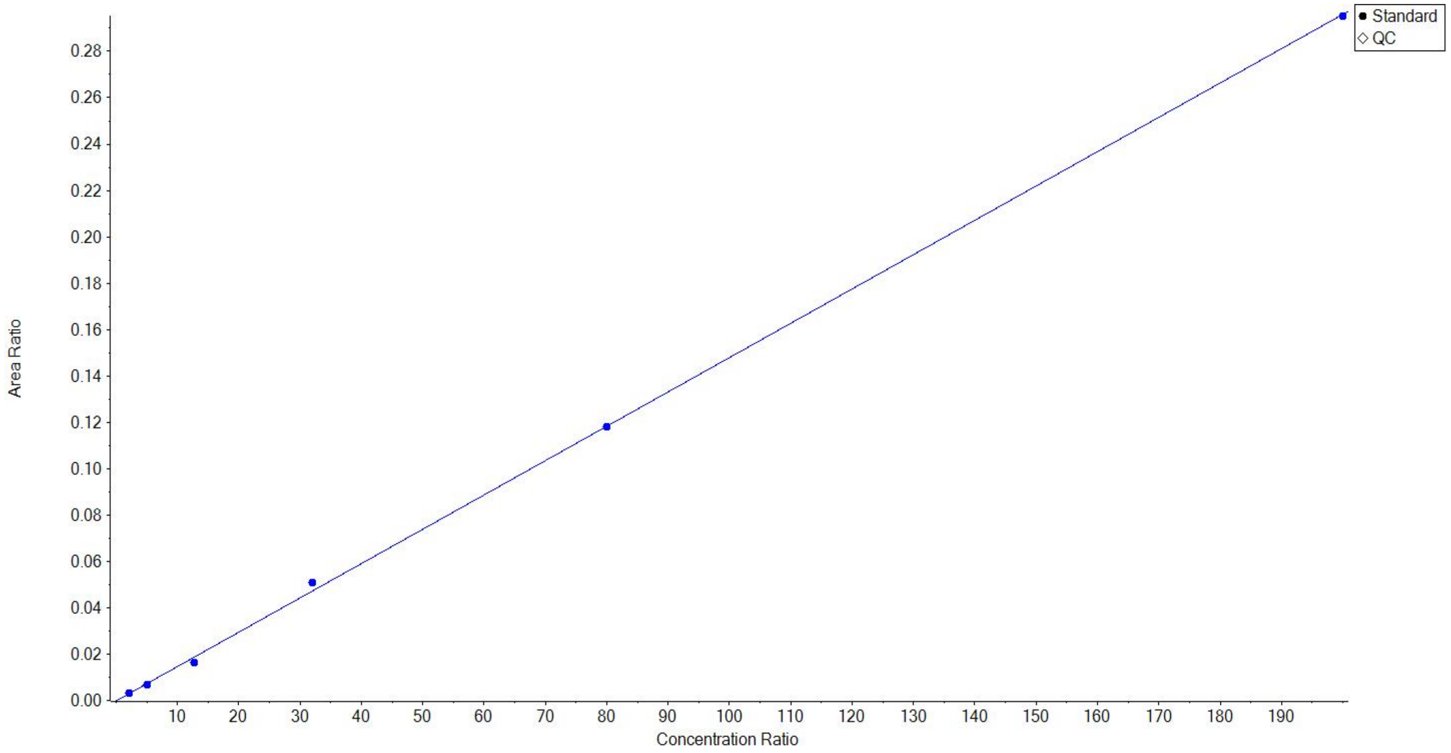
Standard
QC
Area Ratio
0.28
0.26
0.24
0.22
0.20
0.18
0.16
0.14
0.12
0.10
0.08
0.06
0.04
0.02
0.00
10
20
30
40
50
60
70
80
90
100
110
120
130
140
150
160
170
180
190
Concentration Ratio

Analyte Name: LM-flavones-70_1
Internal Standard: LM-flavones-IS02_1

**Data File**	flavones-STD-20230908.wiff	**Result Table**	DZLM2023082419-results-20230913-5500
**Acquisition Date**	9/8/2023 5:37:08 PM	**Algorithm Used**	MQ4
**Acquisition Method**	20230908-flavones-(mix130-T3)-15min.dam	**Instrument Name**	QTRAP 6500+ Low Mass
**Project**	N/A	**Processing Method**	20230412-flavones-(mix130-T3)-15min.qmethod

Regression Equation: y = 0.01051 x + -0.00534 (r = 0.99949, r² = 0.99898) (weighting: 1 / x)

Expected Concentration	Number of Values	Mean Calculated Concentration (No data for Analyte Unit)	% Accuracy	Std. Deviation	%CV
0.01	0 of 1	N/A	N/A	N/A	N/A
0.02	0 of 1	N/A	N/A	N/A	N/A
0.05	0 of 1	N/A	N/A	N/A	N/A
0.13	0 of 1	N/A	N/A	N/A	N/A
0.33	0 of 1	N/A	N/A	N/A	N/A
0.82	0 of 1	N/A	N/A	N/A	N/A
2.05	1 of 1	2.285e0	111.6	N/A	N/A
5.12	1 of 1	4.932e0	96.3	N/A	N/A
12.80	1 of 1	1.210e1	94.5	N/A	N/A
32.00	1 of 1	3.192e1	99.7	N/A	N/A
80.00	1 of 1	7.667e1	95.8	N/A	N/A
200.00	1 of 1	2.041e2	102.0	N/A	N/A

Analyte Name: LM-flavones-70_1

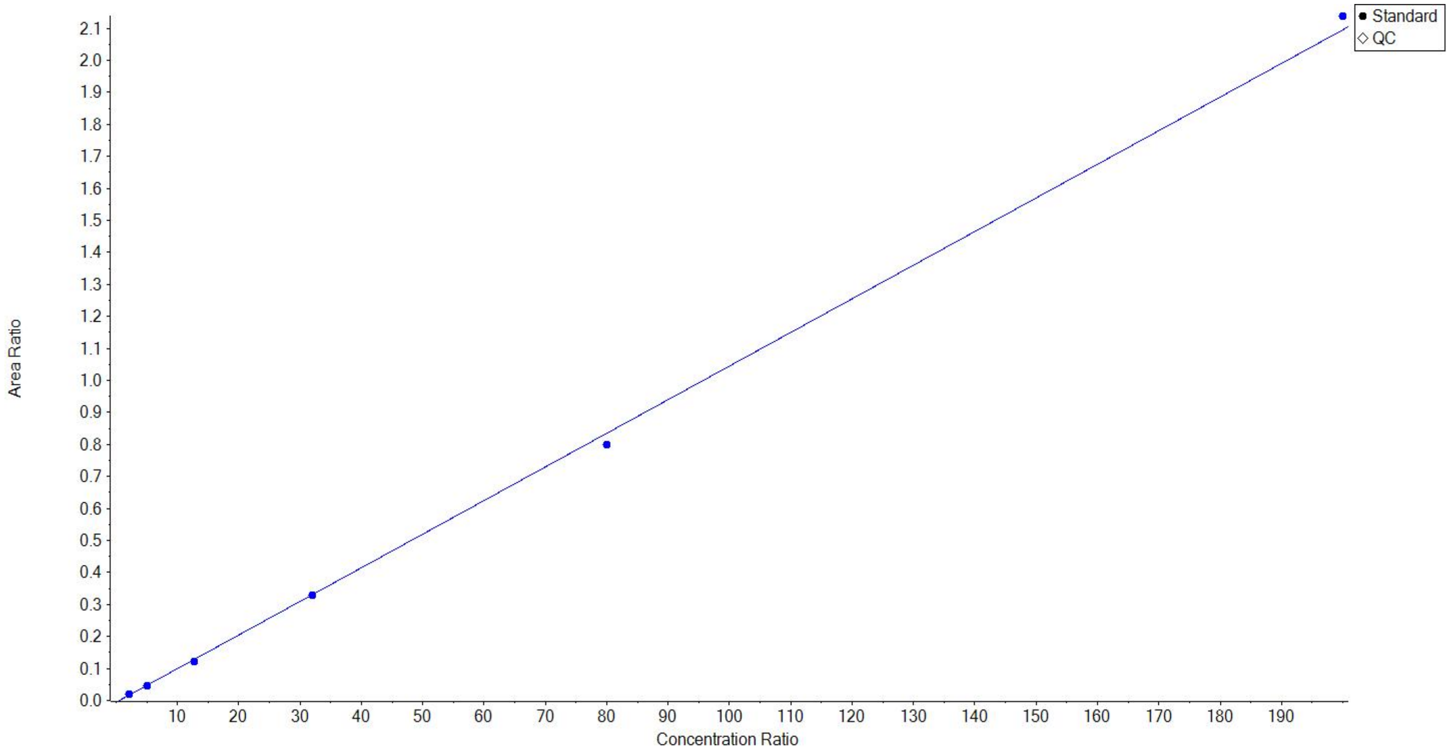
Area Ratio
Concentration Ratio
Standard
QC
0.0
0.1
0.2
0.3
0.4
0.5
0.6
0.7
0.8
0.9
1.0
1.1
1.2
1.3
1.4
1.5
1.6
1.7
1.8
1.9
2.0
2.1
10
20
30
40
50
60
70
80
90
100
110
120
130
140
150
160
170
180
190

Analyte Name: LM-flavones-71_1
Internal Standard: LM-flavones-IS02_1

**Data File**	flavones-STD-20230908.wiff	**Result Table**	DZLM2023082419-results-20230913-5500
**Acquisition Date**	9/8/2023 5:37:08 PM	**Algorithm Used**	MQ4
**Acquisition Method**	20230908-flavones-(mix130-T3)-15min.dam	**Instrument Name**	QTRAP 6500+ Low Mass
**Project**	N/A	**Processing Method**	20230412-flavones-(mix130-T3)-15min.qmethod

Regression Equation: y = 0.00185 x + 9.00730e-4 (r = 0.99783, r² = 0.99567) (weighting: 1 / x)

Expected Concentration	Number of Values	Mean Calculated Concentration (No data for Analyte Unit)	% Accuracy	Std. Deviation	%CV
0.01	0 of 1	N/A	N/A	N/A	N/A
0.02	0 of 1	N/A	N/A	N/A	N/A
0.05	0 of 1	N/A	N/A	N/A	N/A
0.13	0 of 1	N/A	N/A	N/A	N/A
0.33	0 of 1	N/A	N/A	N/A	N/A
0.82	0 of 1	N/A	N/A	N/A	N/A
2.05	1 of 1	1.888e0	92.2	N/A	N/A
5.12	1 of 1	4.380e0	85.6	N/A	N/A
12.80	1 of 1	1.466e1	114.6	N/A	N/A
32.00	1 of 1	3.340e1	104.4	N/A	N/A
80.00	1 of 1	8.600e1	107.5	N/A	N/A
200.00	1 of 1	1.916e2	95.8	N/A	N/A

Analyte Name: LM-flavones-71_1

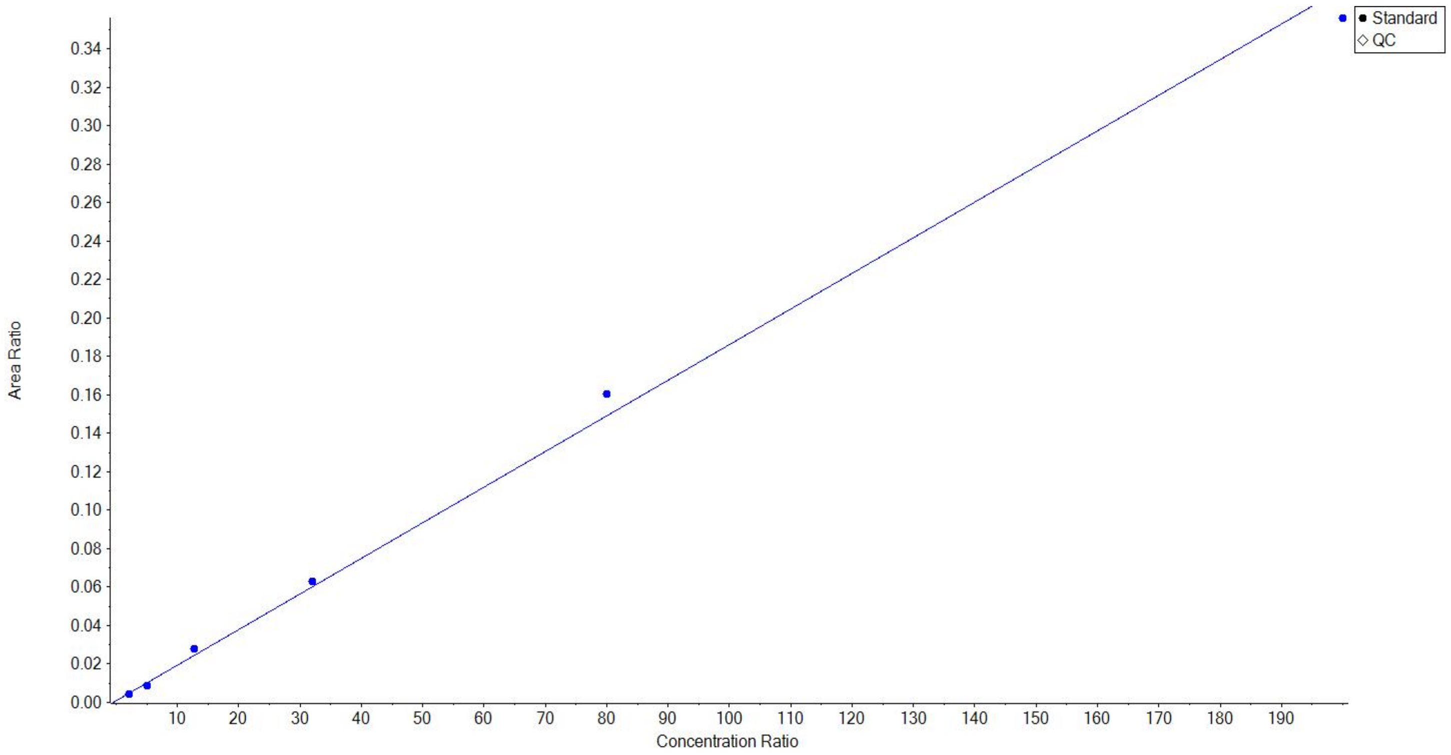
Standard
QC
Area Ratio
0.34
0.32
0.30
0.28
0.26
0.24
0.22
0.20
0.18
0.16
0.14
0.12
0.10
0.08
0.06
0.04
0.02
0.00
10
20
30
40
50
60
70
80
90
100
110
120
130
140
150
160
170
180
190
Concentration Ratio

Analyte Name: LM-flavones-72_1
Internal Standard: LM-flavones-IS02_1

**Data File**	flavones-STD-20230908.wiff	**Result Table**	DZLM2023082419-results-20230913-5500
**Acquisition Date**	9/8/2023 5:37:08 PM	**Algorithm Used**	MQ4
**Acquisition Method**	20230908-flavones-(mix130-T3)-15min.dam	**Instrument Name**	QTRAP 6500+ Low Mass
**Project**	N/A	**Processing Method**	20230412-flavones-(mix130-T3)-15min.qmethod

Regression Equation: y = 0.00828 x + -0.00541 (r = 0.99933, r² = 0.99866) (weighting: 1 / x)

Expected Concentration	Number of Values	Mean Calculated Concentration (No data for Analyte Unit)	% Accuracy	Std. Deviation	%CV
0.01	0 of 1	N/A	N/A	N/A	N/A
0.02	0 of 1	N/A	N/A	N/A	N/A
0.05	0 of 1	N/A	N/A	N/A	N/A
0.13	0 of 1	N/A	N/A	N/A	N/A
0.33	0 of 1	N/A	N/A	N/A	N/A
0.82	0 of 1	N/A	N/A	N/A	N/A
2.05	1 of 1	2.352e0	114.8	N/A	N/A
5.12	1 of 1	4.804e0	93.8	N/A	N/A
12.80	1 of 1	1.183e1	92.5	N/A	N/A
32.00	1 of 1	3.240e1	101.3	N/A	N/A
80.00	1 of 1	7.644e1	95.6	N/A	N/A
200.00	1 of 1	2.041e2	102.1	N/A	N/A

Analyte Name: LM-flavones-72_1

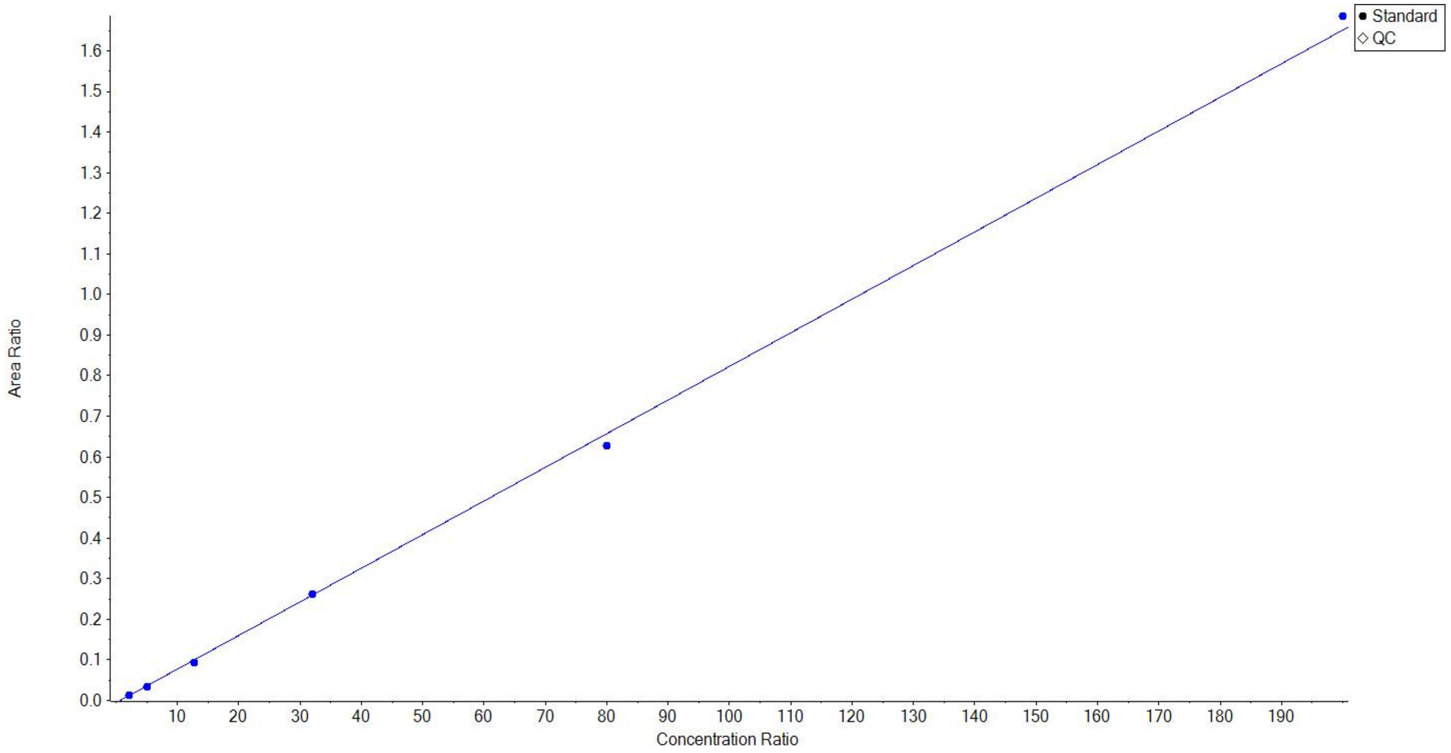
Standard
QC
Area Ratio
1.6
1.5
1.4
1.3
1.2
1.1
1.0
0.9
0.8
0.7
0.6
0.5
0.4
0.3
0.2
0.1
0.0
10
20
30
40
50
60
70
80
90
100
110
120
130
140
150
160
170
180
190
Concentration Ratio

Analyte Name: LM-flavones-73_1
Internal Standard: LM-flavones-IS02_1

**Data File**	flavones-STD-20230908.wiff	**Result Table**	DZLM2023082419-results-20230913-5500
**Acquisition Date**	9/8/2023 5:37:08 PM	**Algorithm Used**	MQ4
**Acquisition Method**	20230908-flavones-(mix130-T3)-15min.dam	**Instrument Name**	QTRAP 6500+ Low Mass
**Project**	N/A	**Processing Method**	20230412-flavones-(mix130-T3)-15min.qmethod

Regression Equation: y = 0.00382 x + 6.65987e-5 (r = 0.99972, r² = 0.99945) (weighting: 1 / x)

Expected Concentration	Number of Values	Mean Calculated Concentration (No data for Analyte Unit)	% Accuracy	Std. Deviation	%CV
0.01	0 of 1	N/A	N/A	N/A	N/A
0.02	0 of 1	N/A	N/A	N/A	N/A
0.05	0 of 1	N/A	N/A	N/A	N/A
0.13	0 of 1	N/A	N/A	N/A	N/A
0.33	0 of 1	N/A	N/A	N/A	N/A
0.82	0 of 1	N/A	N/A	N/A	N/A
2.05	1 of 1	1.915e0	93.4	N/A	N/A
5.12	1 of 1	5.138e0	100.3	N/A	N/A
12.80	1 of 1	1.307e1	102.1	N/A	N/A
32.00	1 of 1	3.299e1	103.1	N/A	N/A
80.00	1 of 1	8.218e1	102.7	N/A	N/A
200.00	1 of 1	1.967e2	98.3	N/A	N/A

Analyte Name: LM-flavones-73_1

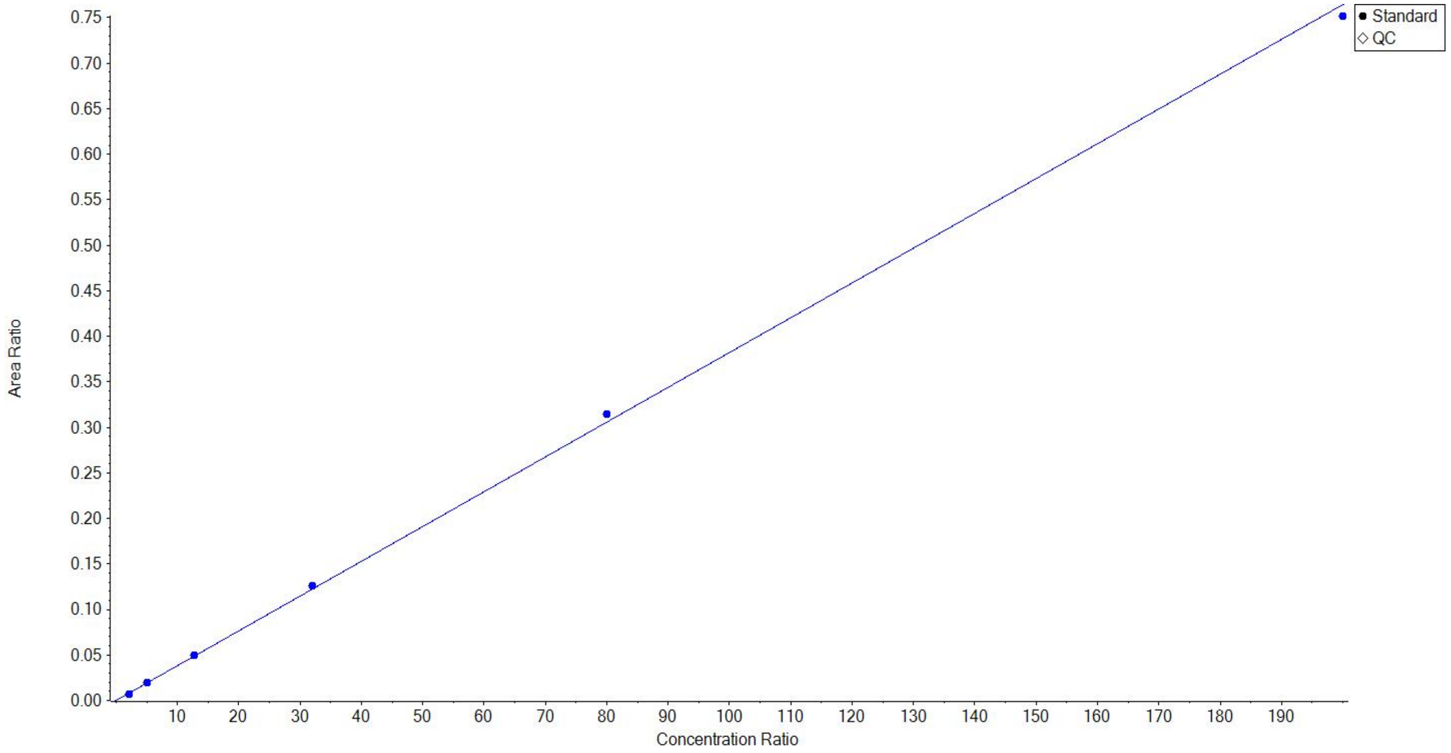
Standard
QC
Area Ratio
Concentration Ratio
0.00
0.05
0.10
0.15
0.20
0.25
0.30
0.35
0.40
0.45
0.50
0.55
0.60
0.65
0.70
0.75
10
20
30
40
50
60
70
80
90
100
110
120
130
140
150
160
170
180
190

Analyte Name: LM-flavones-74_1
Internal Standard: LM-flavones-IS02_1

**Data File**	flavones-STD-20230908.wiff	**Result Table**	DZLM2023082419-results-20230913-5500
**Acquisition Date**	9/8/2023 5:37:08 PM	**Algorithm Used**	MQ4
**Acquisition Method**	20230908-flavones-(mix130-T3)-15min.dam	**Instrument Name**	QTRAP 6500+ Low Mass
**Project**	N/A	**Processing Method**	20230412-flavones-(mix130-T3)-15min.qmethod

Regression Equation: y = 0.01279 x + -0.00261 (r = 0.99985, r² = 0.99971) (weighting: 1 / x)

Expected Concentration	Number of Values	Mean Calculated Concentration (No data for Analyte Unit)	% Accuracy	Std. Deviation	%CV
0.01	0 of 1	N/A	N/A	N/A	N/A
0.02	0 of 1	N/A	N/A	N/A	N/A
0.05	0 of 1	N/A	N/A	N/A	N/A
0.13	0 of 1	N/A	N/A	N/A	N/A
0.33	0 of 1	N/A	N/A	N/A	N/A
0.82	0 of 1	N/A	N/A	N/A	N/A
2.05	1 of 1	2.204e0	107.5	N/A	N/A
5.12	1 of 1	4.888e0	95.5	N/A	N/A
12.80	1 of 1	1.234e1	96.4	N/A	N/A
32.00	1 of 1	3.254e1	101.7	N/A	N/A
80.00	1 of 1	7.855e1	98.2	N/A	N/A
200.00	1 of 1	2.014e2	100.7	N/A	N/A

Analyte Name: LM-flavones-74_1

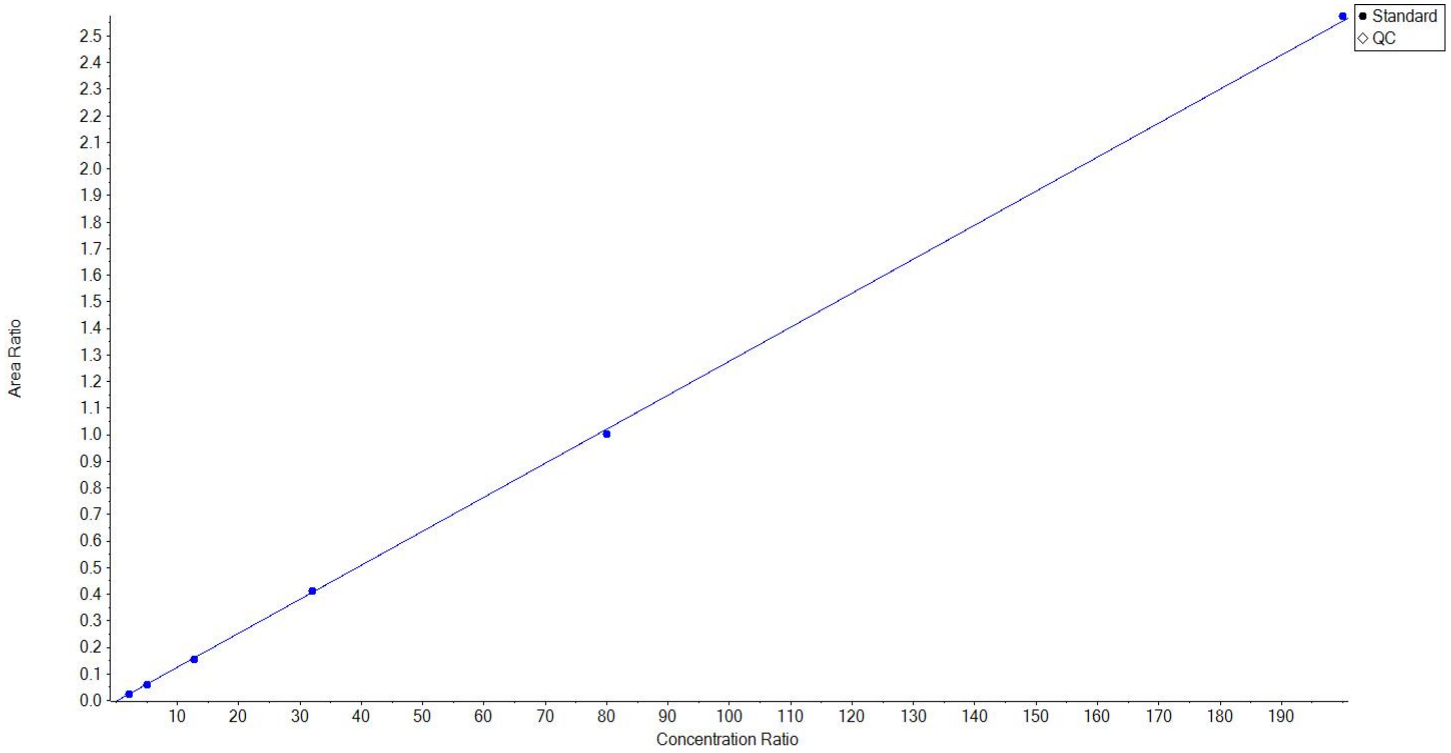
Standard
QC
Area Ratio
Concentration Ratio
0.0
0.1
0.2
0.3
0.4
0.5
0.6
0.7
0.8
0.9
1.0
1.1
1.2
1.3
1.4
1.5
1.6
1.7
1.8
1.9
2.0
2.1
2.2
2.3
2.4
2.5
10
20
30
40
50
60
70
80
90
100
110
120
130
140
150
160
170
180
190

Analyte Name: LM-flavones-75_1
Internal Standard: LM-flavones-IS02_1

**Data File**	flavones-STD-20230908.wiff	**Result Table**	DZLM2023082419-results-20230913-5500
**Acquisition Date**	9/8/2023 5:37:08 PM	**Algorithm Used**	MQ4
**Acquisition Method**	20230908-flavones-(mix130-T3)-15min.dam	**Instrument Name**	QTRAP 6500+ Low Mass
**Project**	N/A	**Processing Method**	20230412-flavones-(mix130-T3)-15min.qmethod

Regression Equation: y = 0.01397 x + -0.00461 (r = 0.99984, r^2 = 0.99969) (weighting: 1 / x)

Expected Concentration	Number of Values	Mean Calculated Concentration (No data for Analyte Unit)	% Accuracy	Std. Deviation	%CV
0.01	0 of 1	N/A	N/A	N/A	N/A
0.02	0 of 1	N/A	N/A	N/A	N/A
0.05	0 of 1	N/A	N/A	N/A	N/A
0.13	0 of 1	N/A	N/A	N/A	N/A
0.33	0 of 1	N/A	N/A	N/A	N/A
0.82	0 of 1	N/A	N/A	N/A	N/A
2.05	1 of 1	2.182e0	106.4	N/A	N/A
5.12	1 of 1	4.990e0	97.5	N/A	N/A
12.80	1 of 1	1.237e1	96.6	N/A	N/A
32.00	1 of 1	3.224e1	100.8	N/A	N/A
80.00	1 of 1	7.818e1	97.7	N/A	N/A
200.00	1 of 1	2.020e2	101.0	N/A	N/A

Analyte Name: LM-flavones-75_1

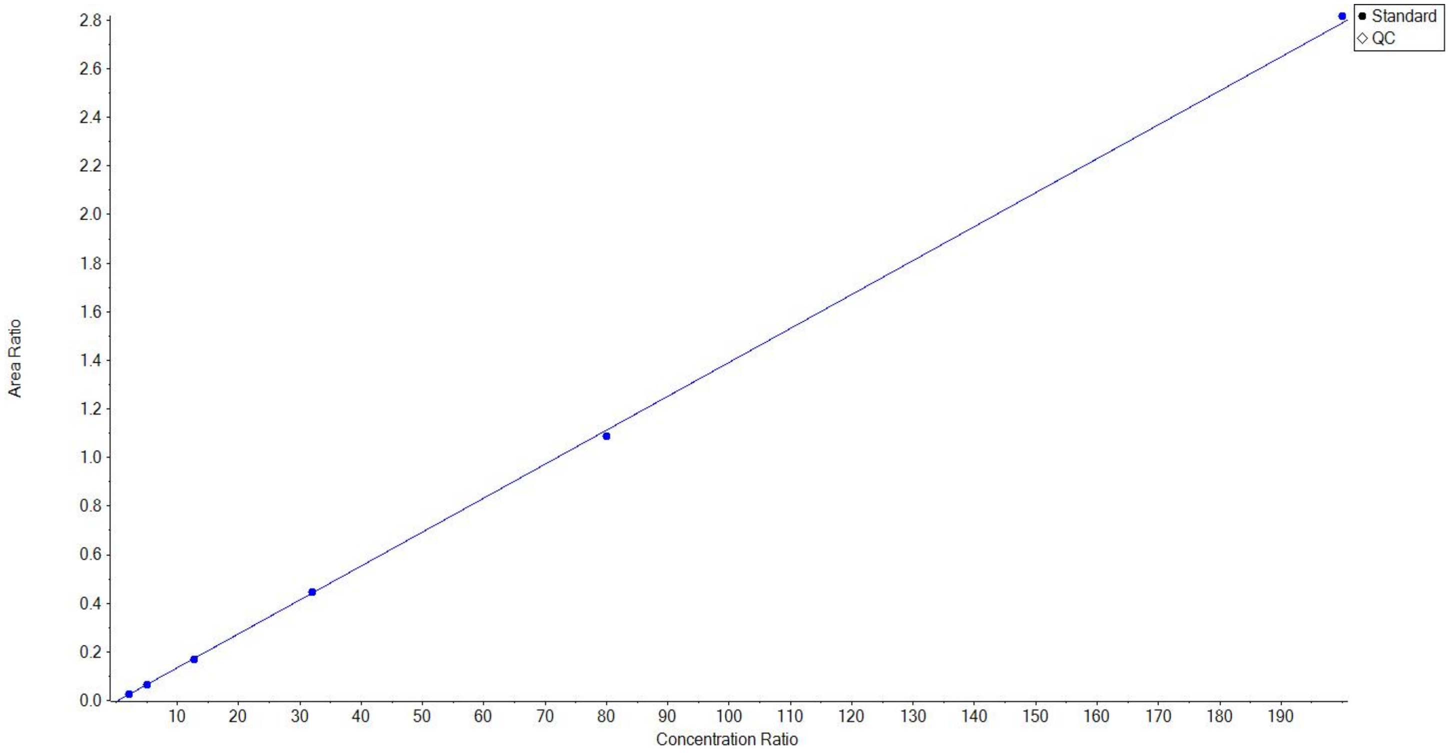
Standard
QC
Area Ratio
Concentration Ratio
0.0
0.2
0.4
0.6
0.8
1.0
1.2
1.4
1.6
1.8
2.0
2.2
2.4
2.6
2.8
10
20
30
40
50
60
70
80
90
100
110
120
130
140
150
160
170
180
190

Analyte Name: LM-flavones-76_1
Internal Standard: LM-flavones-IS02_1

**Data File**	flavones-STD-20230908.wiff	**Result Table**	DZLM2023082419-results-20230913-5500
**Acquisition Date**	9/8/2023 5:37:08 PM	**Algorithm Used**	MQ4
**Acquisition Method**	20230908-flavones-(mix130-T3)-15min.dam	**Instrument Name**	QTRAP 6500+ Low Mass
**Project**	N/A	**Processing Method**	20230412-flavones-(mix130-T3)-15min.qmethod

Regression Equation: y = 3.49922e-4 x + -4.62681e-4 (r = 0.99947, r² = 0.99894) (weighting: 1 / x)

Expected Concentration	Number of Values	Mean Calculated Concentration (No data for Analyte Unit)	% Accuracy	Std. Deviation	%CV
0.01	0 of 1	N/A	N/A	N/A	N/A
0.02	0 of 1	N/A	N/A	N/A	N/A
0.05	0 of 1	N/A	N/A	N/A	N/A
0.13	0 of 1	N/A	N/A	N/A	N/A
0.33	0 of 1	N/A	N/A	N/A	N/A
0.82	0 of 1	N/A	N/A	N/A	N/A
2.05	0 of 1	N/A	N/A	N/A	N/A
5.12	1 of 1	5.037e0	98.4	N/A	N/A
12.80	1 of 1	1.364e1	106.6	N/A	N/A
32.00	1 of 1	3.119e1	97.5	N/A	N/A
80.00	1 of 1	7.671e1	95.9	N/A	N/A
200.00	1 of 1	2.033e2	101.7	N/A	N/A

Analyte Name: LM-flavones-76_1

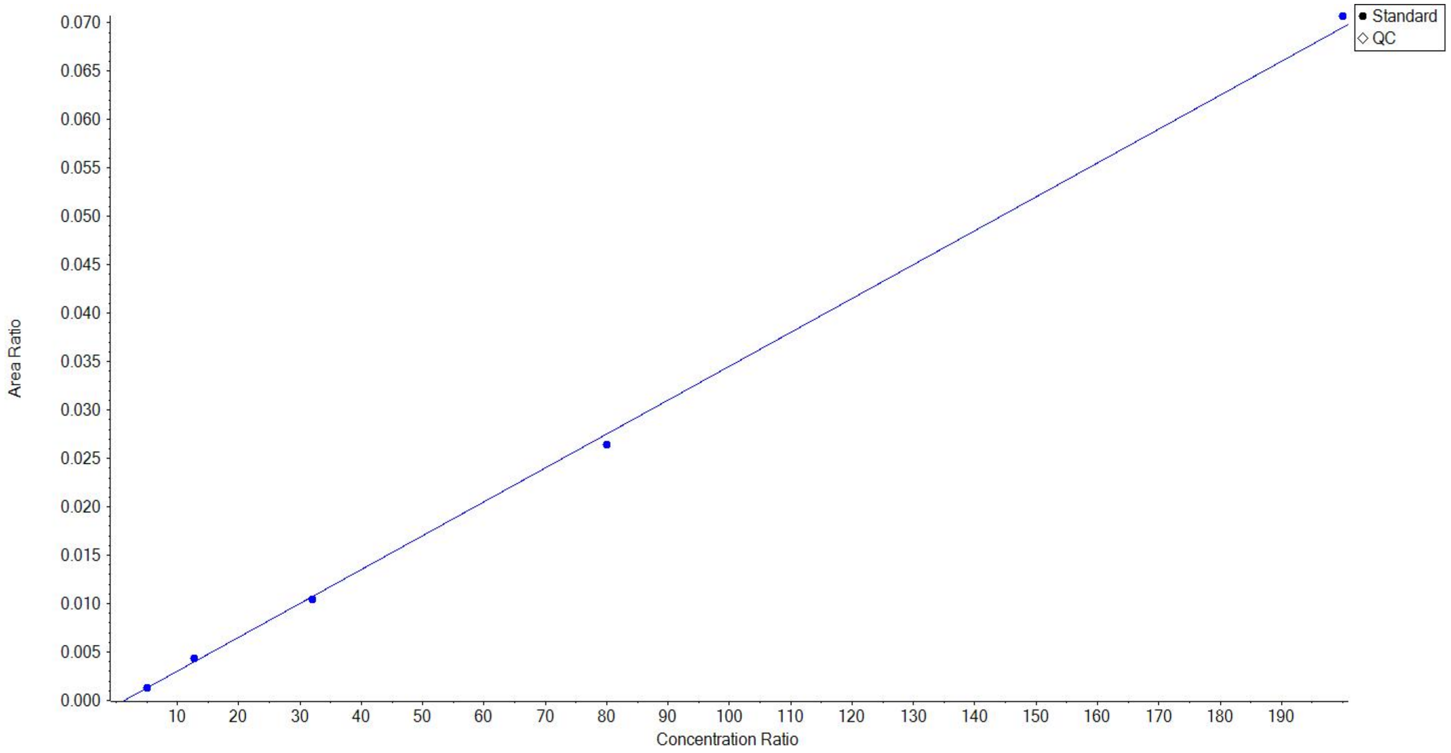
Standard
QC
Area Ratio
0.070
0.065
0.060
0.055
0.050
0.045
0.040
0.035
0.030
0.025
0.020
0.015
0.010
0.005
0.000
10
20
30
40
50
60
70
80
90
100
110
120
130
140
150
160
170
180
190
Concentration Ratio

Analyte Name: LM-flavones-77_1
Internal Standard: LM-flavones-IS02_1

**Data File**	flavones-STD-20230908.wiff	**Result Table**	DZLM2023082419-results-20230913-5500
**Acquisition Date**	9/8/2023 5:37:08 PM	**Algorithm Used**	MQ4
**Acquisition Method**	20230908-flavones-(mix130-T3)-15min.dam	**Instrument Name**	QTRAP 6500+ Low Mass
**Project**	N/A	**Processing Method**	20230412-flavones-(mix130-T3)-15min.qmethod

Regression Equation: y = 4.42495e-4 x + -1.07814e-4 (r = 0.99962, r² = 0.99923) (weighting: 1 / x)

Expected Concentration	Number of Values	Mean Calculated Concentration (No data for Analyte Unit)	% Accuracy	Std. Deviation	%CV
0.01	0 of 1	N/A	N/A	N/A	N/A
0.02	0 of 1	N/A	N/A	N/A	N/A
0.05	0 of 1	N/A	N/A	N/A	N/A
0.13	0 of 1	N/A	N/A	N/A	N/A
0.33	0 of 1	N/A	N/A	N/A	N/A
0.82	0 of 1	N/A	N/A	N/A	N/A
2.05	1 of 1	2.411e0	117.6	N/A	N/A
5.12	1 of 1	4.544e0	88.8	N/A	N/A
12.80	1 of 1	1.201e1	93.9	N/A	N/A
32.00	1 of 1	3.210e1	100.3	N/A	N/A
80.00	1 of 1	7.871e1	98.4	N/A	N/A
200.00	1 of 1	2.022e2	101.1	N/A	N/A

Analyte Name: LM-flavones-77_1

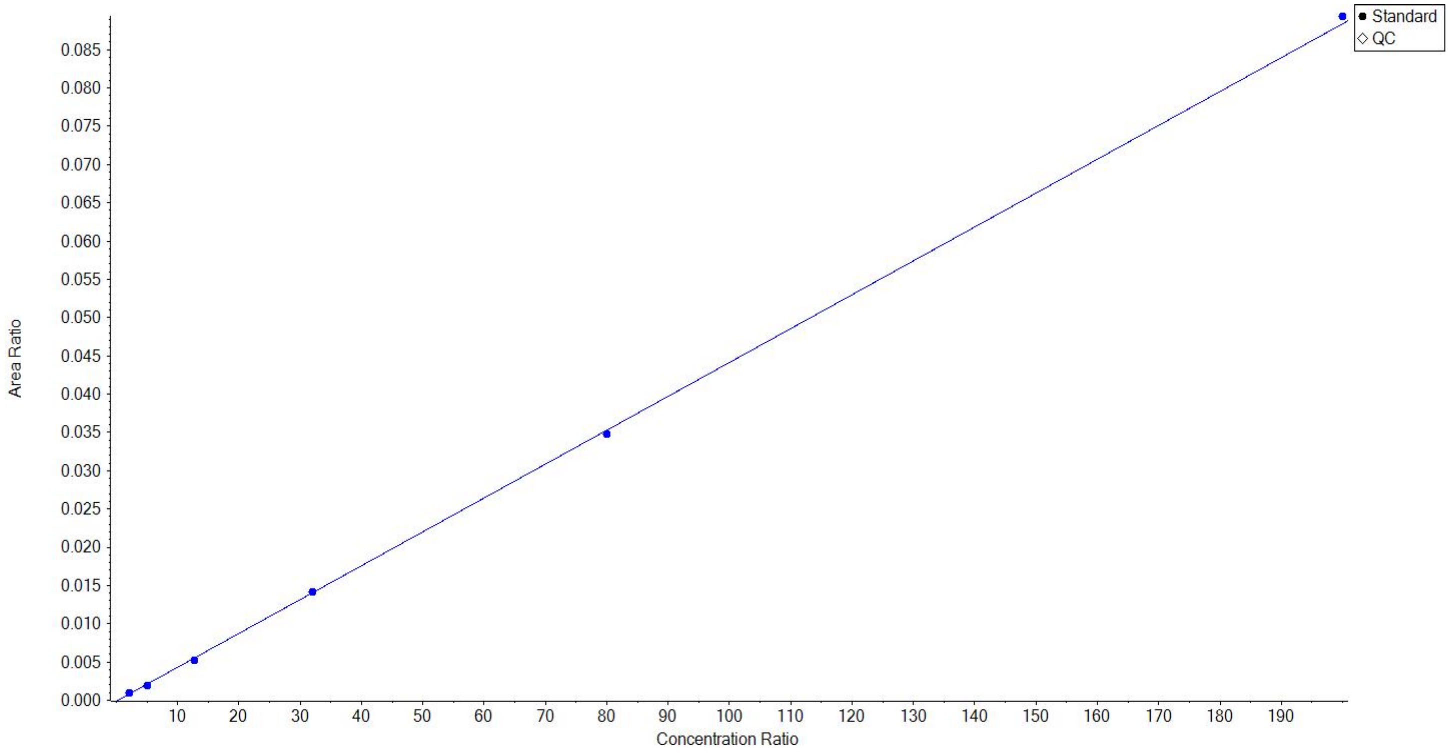
Standard
QC
Area Ratio
Concentration Ratio
0.000
0.005
0.010
0.015
0.020
0.025
0.030
0.035
0.040
0.045
0.050
0.055
0.060
0.065
0.070
0.075
0.080
0.085
10 20 30 40 50 60 70 80 90 100 110 120 130 140 150 160 170 180 190

Analyte Name: LM-flavones-78_1
Internal Standard: LM-flavones-IS02_1

**Data File**	flavones-STD-20230908.wiff	**Result Table**	DZLM2023082419-results-20230913-5500
**Acquisition Date**	9/8/2023 5:37:08 PM	**Algorithm Used**	MQ4
**Acquisition Method**	20230908-flavones-(mix130-T3)-15min.dam	**Instrument Name**	QTRAP 6500+ Low Mass
**Project**	N/A	**Processing Method**	20230412-flavones-(mix130-T3)-15min.qmethod

Regression Equation: y = 2.21610e-4 x + -3.30073e-5 (r = 0.99993, r² = 0.99985) (weighting: 1 / x)

Expected Concentration	Number of Values	Mean Calculated Concentration (No data for Analyte Unit)	% Accuracy	Std. Deviation	%CV
0.01	0 of 1	N/A	N/A	N/A	N/A
0.02	0 of 1	N/A	N/A	N/A	N/A
0.05	0 of 1	N/A	N/A	N/A	N/A
0.13	0 of 1	N/A	N/A	N/A	N/A
0.33	0 of 1	N/A	N/A	N/A	N/A
0.82	0 of 1	N/A	N/A	N/A	N/A
2.05	1 of 1	2.162e0	105.5	N/A	N/A
5.12	1 of 1	4.735e0	92.5	N/A	N/A
12.80	1 of 1	1.295e1	101.2	N/A	N/A
32.00	1 of 1	3.240e1	101.3	N/A	N/A
80.00	1 of 1	7.968e1	99.6	N/A	N/A
200.00	1 of 1	2.000e2	100.0	N/A	N/A

Analyte Name: LM-flavones-78_1

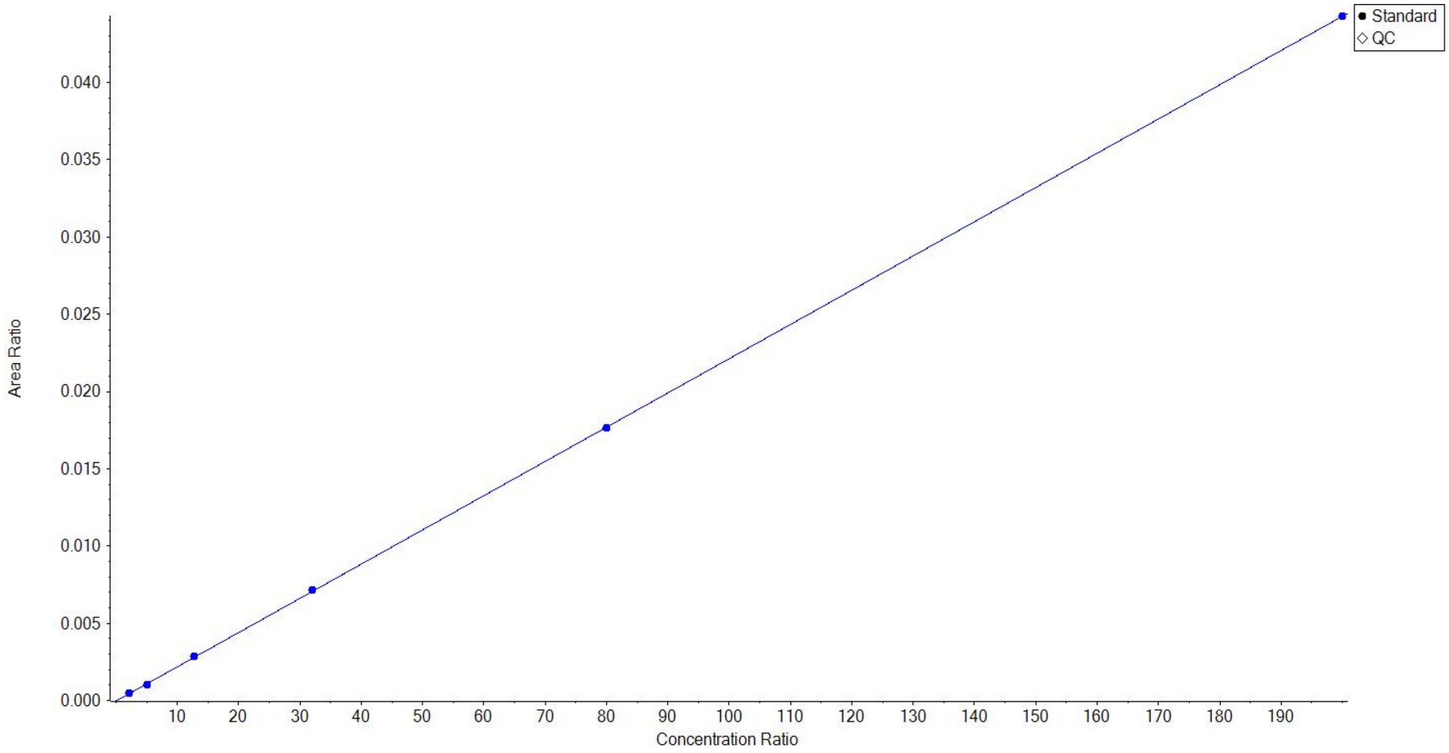
Standard
QC
0.040
0.035
0.030
0.025
0.020
0.015
0.010
0.005
0.000
Area Ratio
10
20
30
40
50
60
70
80
90
100
110
120
130
140
150
160
170
180
190
Concentration Ratio

Analyte Name: LM-flavones-79_1
Internal Standard: LM-flavones-IS02_1

**Data File**	flavones-STD-20230908.wiff	**Result Table**	DZLM2023082419-results-20230913-5500
**Acquisition Date**	9/8/2023 5:37:08 PM	**Algorithm Used**	MQ4
**Acquisition Method**	20230908-flavones-(mix130-T3)-15min.dam	**Instrument Name**	QTRAP 6500+ Low Mass
**Project**	N/A	**Processing Method**	20230412-flavones-(mix130-T3)-15min.qmethod

Regression Equation: y = 0.00819 x + -7.70716e-4 (r = 0.99973, r^2 = 0.99946) (weighting: 1 / x)

Expected Concentration	Number of Values	Mean Calculated Concentration (No data for Analyte Unit)	% Accuracy	Std. Deviation	%CV
0.01	0 of 1	N/A	N/A	N/A	N/A
0.02	0 of 1	N/A	N/A	N/A	N/A
0.05	0 of 1	N/A	N/A	N/A	N/A
0.13	0 of 1	N/A	N/A	N/A	N/A
0.33	0 of 1	N/A	N/A	N/A	N/A
0.82	0 of 1	N/A	N/A	N/A	N/A
2.05	1 of 1	2.008e0	98.0	N/A	N/A
5.12	1 of 1	5.060e0	98.8	N/A	N/A
12.80	1 of 1	1.262e1	98.6	N/A	N/A
32.00	1 of 1	3.404e1	106.4	N/A	N/A
80.00	1 of 1	7.870e1	98.4	N/A	N/A
200.00	1 of 1	1.995e2	99.8	N/A	N/A

Analyte Name: LM-flavones-79_1

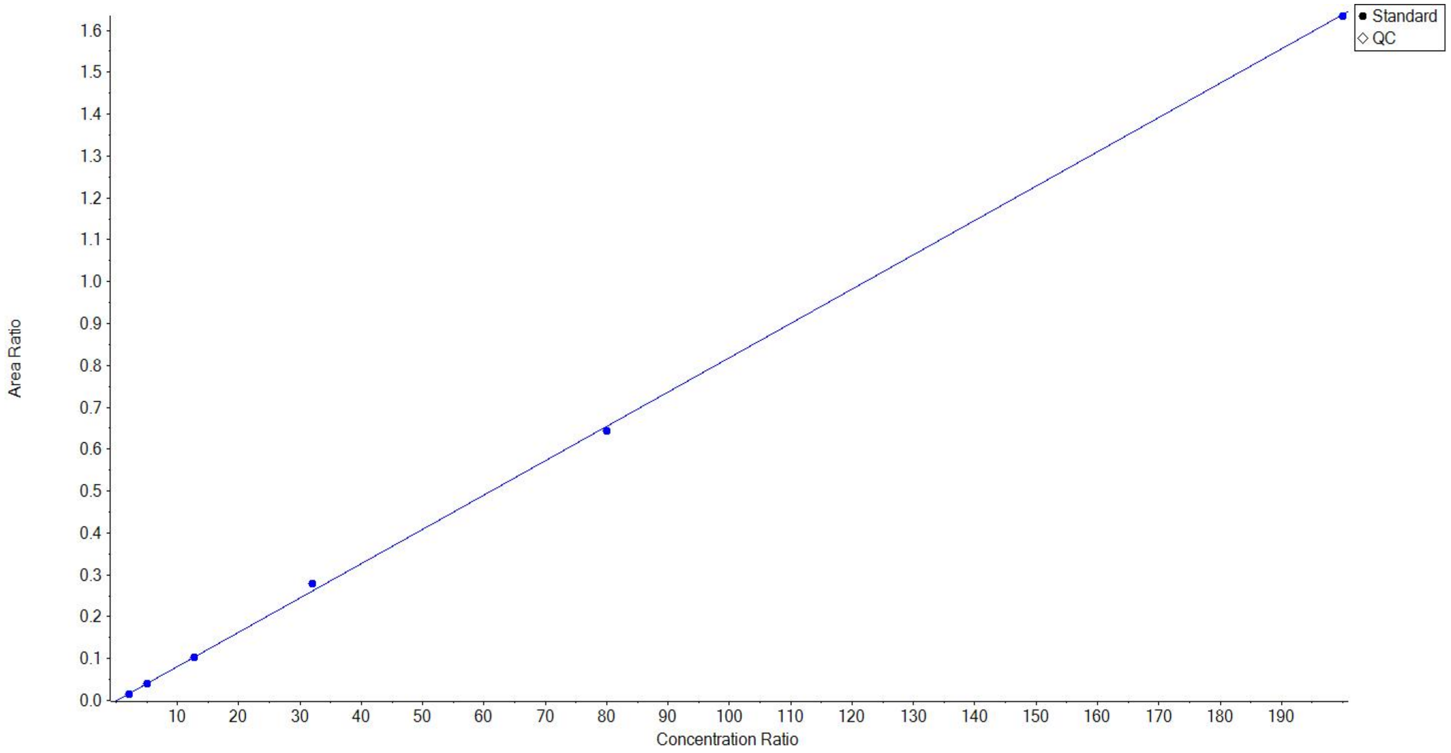
Standard
QC
Area Ratio
Concentration Ratio
1.6
1.5
1.4
1.3
1.2
1.1
1.0
0.9
0.8
0.7
0.6
0.5
0.4
0.3
0.2
0.1
0.0
10
20
30
40
50
60
70
80
90
100
110
120
130
140
150
160
170
180
190

Analyte Name: LM-flavones-81_1
Internal Standard: LM-flavones-IS02_1

**Data File**	flavones-STD-20230908.wiff	**Result Table**	DZLM2023082419-results-20230913-5500
**Acquisition Date**	9/8/2023 5:37:08 PM	**Algorithm Used**	MQ4
**Acquisition Method**	20230908-flavones-(mix130-T3)-15min.dam	**Instrument Name**	QTRAP 6500+ Low Mass
**Project**	N/A	**Processing Method**	20230412-flavones-(mix130-T3)-15min.qmethod

Regression Equation: y = 0.00241 x + -7.60653e-4 (r = 0.99972, r^2 = 0.99944) (weighting: 1 / x)

Expected Concentration	Number of Values	Mean Calculated Concentration (No data for Analyte Unit)	% Accuracy	Std. Deviation	%CV
0.01	0 of 1	N/A	N/A	N/A	N/A
0.02	0 of 1	N/A	N/A	N/A	N/A
0.05	0 of 1	N/A	N/A	N/A	N/A
0.13	0 of 1	N/A	N/A	N/A	N/A
0.33	0 of 1	N/A	N/A	N/A	N/A
0.82	0 of 1	N/A	N/A	N/A	N/A
2.05	1 of 1	2.131e0	104.1	N/A	N/A
5.12	1 of 1	5.375e0	105.0	N/A	N/A
12.80	1 of 1	1.204e1	94.1	N/A	N/A
32.00	1 of 1	3.122e1	97.6	N/A	N/A
80.00	1 of 1	7.827e1	97.8	N/A	N/A
200.00	1 of 1	2.029e2	101.5	N/A	N/A

Analyte Name: LM-flavones-81_1

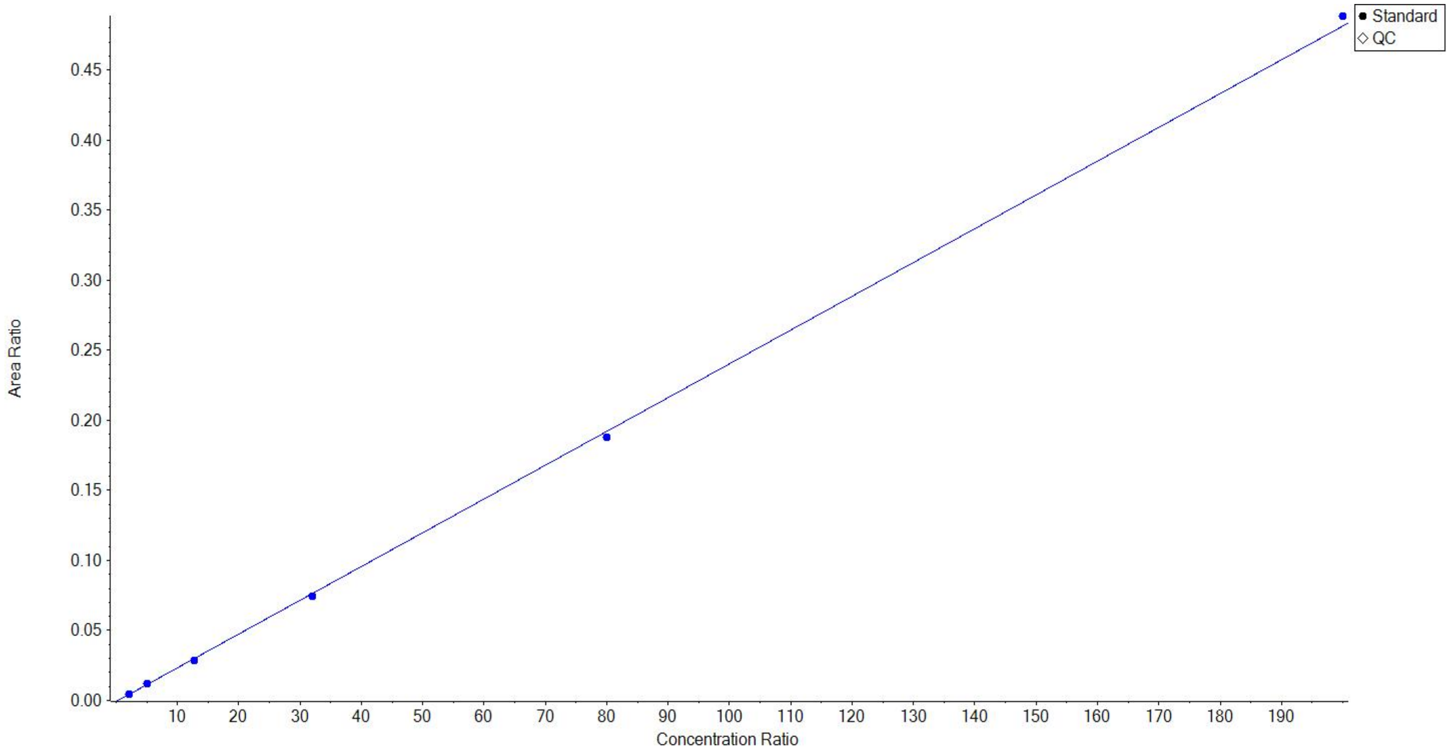
Standard
QC
Area Ratio
0.00
0.05
0.10
0.15
0.20
0.25
0.30
0.35
0.40
0.45
10
20
30
40
50
60
70
80
90
100
110
120
130
140
150
160
170
180
190
Concentration Ratio

Analyte Name: LM-flavones-82_1
Internal Standard: LM-flavones-IS02_1

**Data File**	flavones-STD-20230908.wiff	**Result Table**	DZLM2023082419-results-20230913-5500
**Acquisition Date**	9/8/2023 5:37:08 PM	**Algorithm Used**	MQ4
**Acquisition Method**	20230908-flavones-(mix130-T3)-15min.dam	**Instrument Name**	QTRAP 6500+ Low Mass
**Project**	N/A	**Processing Method**	20230412-flavones-(mix130-T3)-15min.qmethod

Regression Equation: y = 0.00580 x + -0.00127 (r = 0.99994, r² = 0.99988) (weighting: 1 / x)

Expected Concentration	Number of Values	Mean Calculated Concentration (No data for Analyte Unit)	% Accuracy	Std. Deviation	%CV
0.01	0 of 1	N/A	N/A	N/A	N/A
0.02	0 of 1	N/A	N/A	N/A	N/A
0.05	0 of 1	N/A	N/A	N/A	N/A
0.13	0 of 1	N/A	N/A	N/A	N/A
0.33	0 of 1	N/A	N/A	N/A	N/A
0.82	0 of 1	N/A	N/A	N/A	N/A
2.05	1 of 1	2.163e0	105.6	N/A	N/A
5.12	1 of 1	5.046e0	98.6	N/A	N/A
12.80	1 of 1	1.245e1	97.2	N/A	N/A
32.00	1 of 1	3.132e1	97.9	N/A	N/A
80.00	1 of 1	8.028e1	100.3	N/A	N/A
200.00	1 of 1	2.007e2	100.4	N/A	N/A

Analyte Name: LM-flavones-82_1

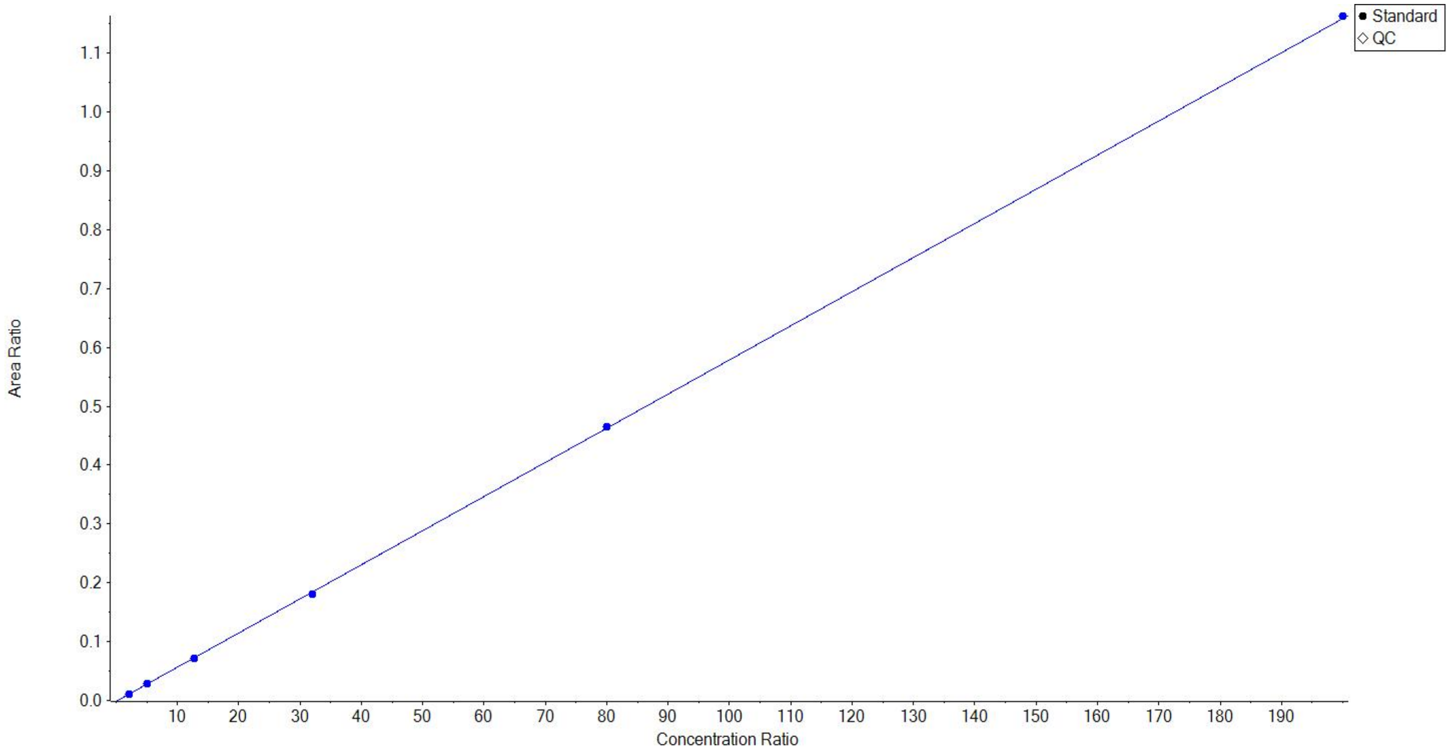
Standard
QC
Area Ratio
1.1
1.0
0.9
0.8
0.7
0.6
0.5
0.4
0.3
0.2
0.1
0.0
10
20
30
40
50
60
70
80
90
100
110
120
130
140
150
160
170
180
190
Concentration Ratio

Analyte Name: LM-flavones-83_1
Internal Standard: LM-flavones-IS02_1

**Data File**	flavones-STD-20230908.wiff	**Result Table**	DZLM2023082419-results-20230913-5500
**Acquisition Date**	9/8/2023 5:37:08 PM	**Algorithm Used**	MQ4
**Acquisition Method**	20230908-flavones-(mix130-T3)-15min.dam	**Instrument Name**	QTRAP 6500+ Low Mass
**Project**	N/A	**Processing Method**	20230412-flavones-(mix130-T3)-15min.qmethod

Regression Equation: y = 0.01016 x + -0.00146 (r = 0.99988, r² = 0.99976) (weighting: 1 / x)

Expected Concentration	Number of Values	Mean Calculated Concentration (No data for Analyte Unit)	% Accuracy	Std. Deviation	%CV
0.01	0 of 1	N/A	N/A	N/A	N/A
0.02	0 of 1	N/A	N/A	N/A	N/A
0.05	0 of 1	N/A	N/A	N/A	N/A
0.13	0 of 1	N/A	N/A	N/A	N/A
0.33	0 of 1	N/A	N/A	N/A	N/A
0.82	0 of 1	N/A	N/A	N/A	N/A
2.05	1 of 1	2.099e0	102.4	N/A	N/A
5.12	1 of 1	4.914e0	96.0	N/A	N/A
12.80	1 of 1	1.268e1	99.1	N/A	N/A
32.00	1 of 1	3.320e1	103.8	N/A	N/A
80.00	1 of 1	7.898e1	98.7	N/A	N/A
200.00	1 of 1	2.001e2	100.0	N/A	N/A

Analyte Name: LM-flavones-83_1

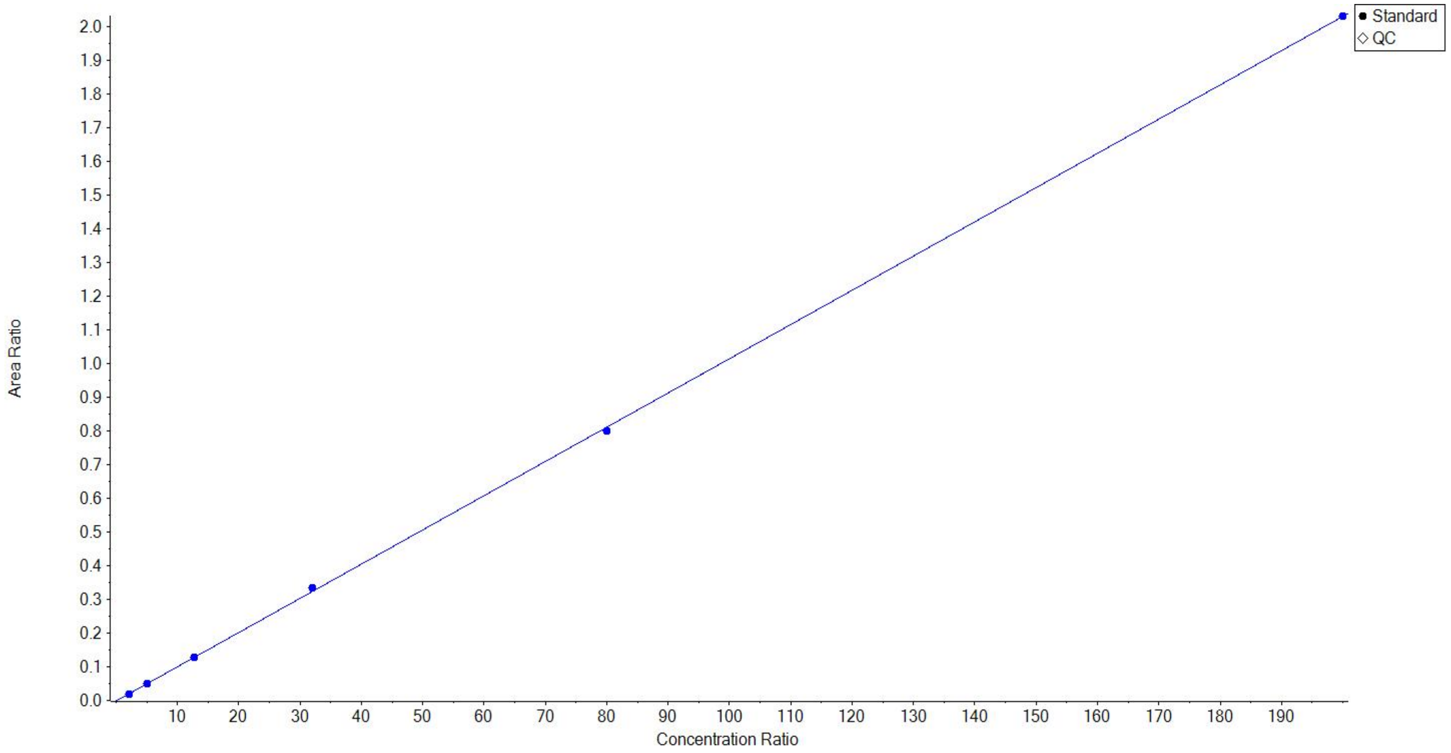
Standard
QC
Area Ratio
Concentration Ratio
2.0
1.9
1.8
1.7
1.6
1.5
1.4
1.3
1.2
1.1
1.0
0.9
0.8
0.7
0.6
0.5
0.4
0.3
0.2
0.1
0.0
10
20
30
40
50
60
70
80
90
100
110
120
130
140
150
160
170
180
190

Analyte Name: LM-flavones-84_1
Internal Standard: LM-flavones-IS02_1

**Data File**	flavones-STD-20230908.wiff	**Result Table**	DZLM2023082419-results-20230913-5500
**Acquisition Date**	9/8/2023 5:37:08 PM	**Algorithm Used**	MQ4
**Acquisition Method**	20230908-flavones-(mix130-T3)-15min.dam	**Instrument Name**	QTRAP 6500+ Low Mass
**Project**	N/A	**Processing Method**	20230412-flavones-(mix130-T3)-15min.qmethod

Regression Equation: y = 0.00225 x + -0.00117 (r = 0.99989, r² = 0.99979) (weighting: 1 / x)

Expected Concentration	Number of Values	Mean Calculated Concentration (No data for Analyte Unit)	% Accuracy	Std. Deviation	%CV
0.01	0 of 1	N/A	N/A	N/A	N/A
0.02	0 of 1	N/A	N/A	N/A	N/A
0.05	0 of 1	N/A	N/A	N/A	N/A
0.13	0 of 1	N/A	N/A	N/A	N/A
0.33	0 of 1	N/A	N/A	N/A	N/A
0.82	0 of 1	N/A	N/A	N/A	N/A
2.05	1 of 1	2.142e0	104.5	N/A	N/A
5.12	1 of 1	4.946e0	96.6	N/A	N/A
12.80	1 of 1	1.230e1	96.1	N/A	N/A
32.00	1 of 1	3.301e1	103.2	N/A	N/A
80.00	1 of 1	7.982e1	99.8	N/A	N/A
200.00	1 of 1	1.998e2	99.9	N/A	N/A

Analyte Name: LM-flavones-84_1

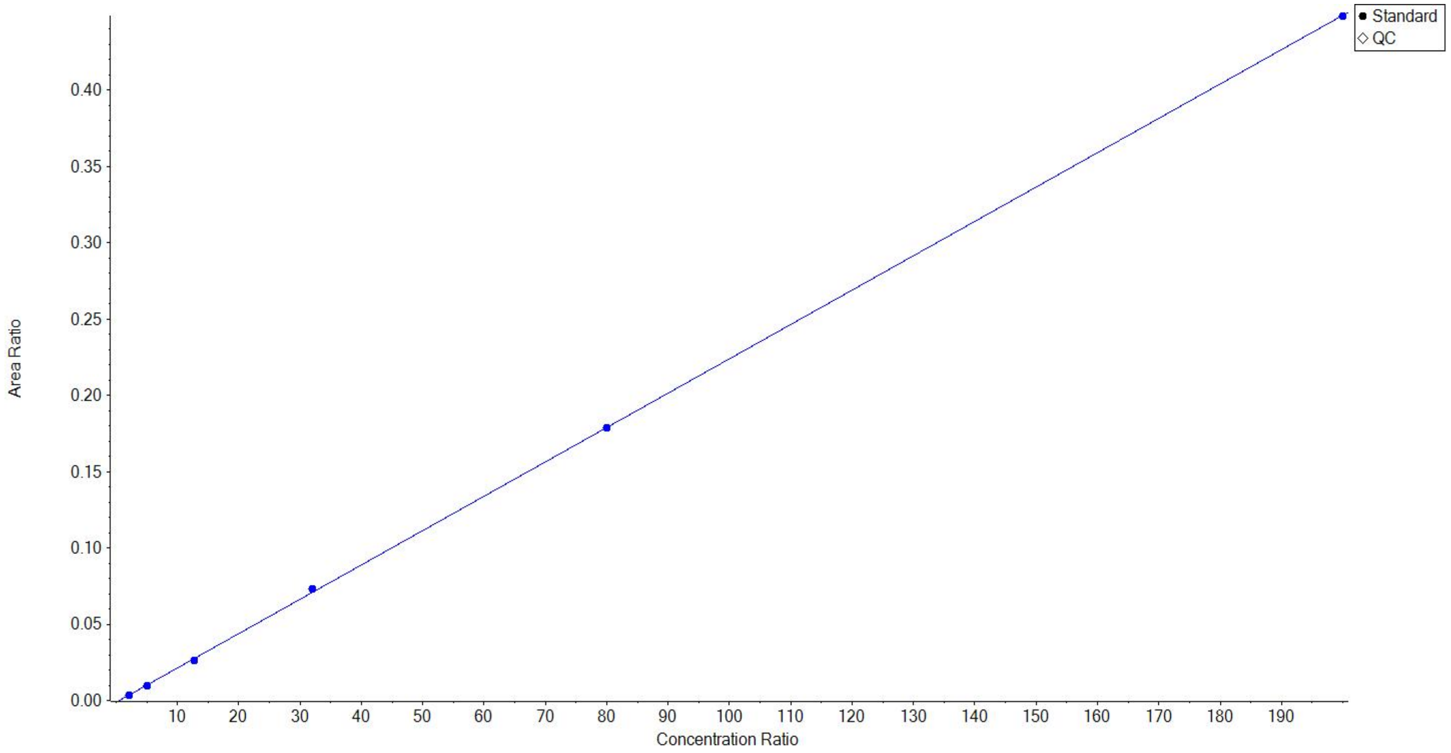
Standard
QC
Area Ratio
0.00
0.05
0.10
0.15
0.20
0.25
0.30
0.35
0.40
10
20
30
40
50
60
70
80
90
100
110
120
130
140
150
160
170
180
190
Concentration Ratio

Analyte Name: LM-flavones-86_1
Internal Standard: LM-flavones-IS02_1

**Data File**	flavones-STD-20230908.wiff	**Result Table**	DZLM2023082419-results-20230913-5500
**Acquisition Date**	9/8/2023 5:37:08 PM	**Algorithm Used**	MQ4
**Acquisition Method**	20230908-flavones-(mix130-T3)-15min.dam	**Instrument Name**	QTRAP 6500+ Low Mass
**Project**	N/A	**Processing Method**	20230412-flavones-(mix130-T3)-15min.qmethod

Regression Equation: $y = 0.00404\ x + 2.36430e\text{-}5$ ($r = 0.99962$, $r^2 = 0.99923$) (weighting: 1 / x)

Expected Concentration	Number of Values	Mean Calculated Concentration (No data for Analyte Unit)	% Accuracy	Std. Deviation	%CV
0.01	0 of 1	N/A	N/A	N/A	N/A
0.02	0 of 1	N/A	N/A	N/A	N/A
0.05	0 of 1	N/A	N/A	N/A	N/A
0.13	0 of 1	N/A	N/A	N/A	N/A
0.33	0 of 1	N/A	N/A	N/A	N/A
0.82	0 of 1	N/A	N/A	N/A	N/A
2.05	1 of 1	1.983e0	96.7	N/A	N/A
5.12	1 of 1	5.287e0	103.3	N/A	N/A
12.80	1 of 1	1.203e1	94.0	N/A	N/A
32.00	1 of 1	3.424e1	107.0	N/A	N/A
80.00	1 of 1	7.974e1	99.7	N/A	N/A
200.00	1 of 1	1.987e2	99.4	N/A	N/A

Analyte Name: LM-flavones-86_1

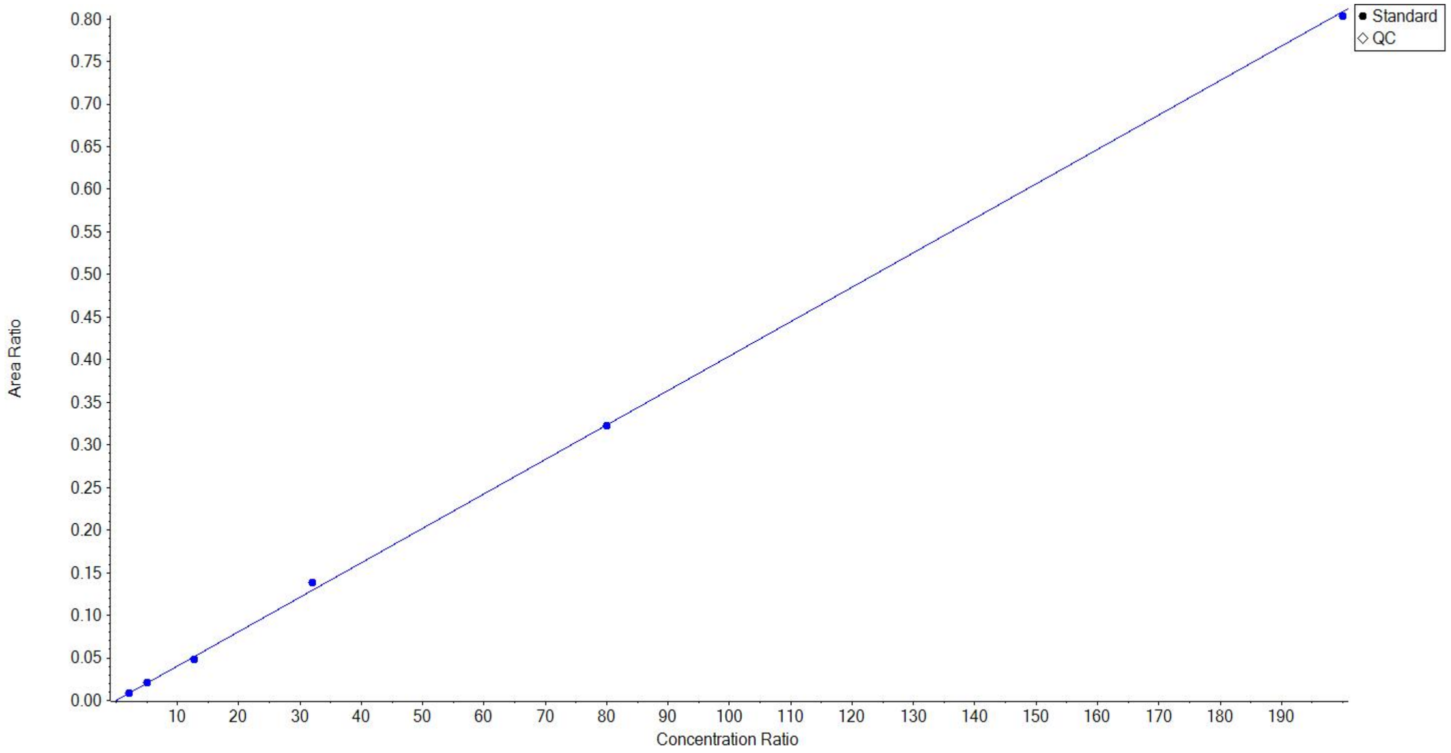
Area Ratio
0.80
0.75
0.70
0.65
0.60
0.55
0.50
0.45
0.40
0.35
0.30
0.25
0.20
0.15
0.10
0.05
0.00
10 20 30 40 50 60 70 80 90 100 110 120 130 140 150 160 170 180 190
Concentration Ratio
Standard
QC

Analyte Name: LM-flavones-87_1
Internal Standard: LM-flavones-IS02_1

**Data File**	flavones-STD-20230908.wiff	**Result Table**	DZLM2023082419-results-20230913-5500
**Acquisition Date**	9/8/2023 5:37:08 PM	**Algorithm Used**	MQ4
**Acquisition Method**	20230908-flavones-(mix130-T3)-15min.dam	**Instrument Name**	QTRAP 6500+ Low Mass
**Project**	N/A	**Processing Method**	20230412-flavones-(mix130-T3)-15min.qmethod

Regression Equation: y = 0.00175 x + 2.57062e-4 (r = 0.99875, r^2 = 0.99750) (weighting: 1 / x)

Expected Concentration	Number of Values	Mean Calculated Concentration (No data for Analyte Unit)	% Accuracy	Std. Deviation	%CV
0.01	0 of 1	N/A	N/A	N/A	N/A
0.02	0 of 1	N/A	N/A	N/A	N/A
0.05	0 of 1	N/A	N/A	N/A	N/A
0.13	0 of 1	N/A	N/A	N/A	N/A
0.33	0 of 1	N/A	N/A	N/A	N/A
0.82	0 of 1	N/A	N/A	N/A	N/A
2.05	1 of 1	2.120e0	103.4	N/A	N/A
5.12	1 of 1	4.357e0	85.1	N/A	N/A
12.80	1 of 1	1.283e1	100.3	N/A	N/A
32.00	1 of 1	3.617e1	113.0	N/A	N/A
80.00	1 of 1	7.994e1	99.9	N/A	N/A
200.00	1 of 1	1.965e2	98.3	N/A	N/A

Analyte Name: LM-flavones-87_1

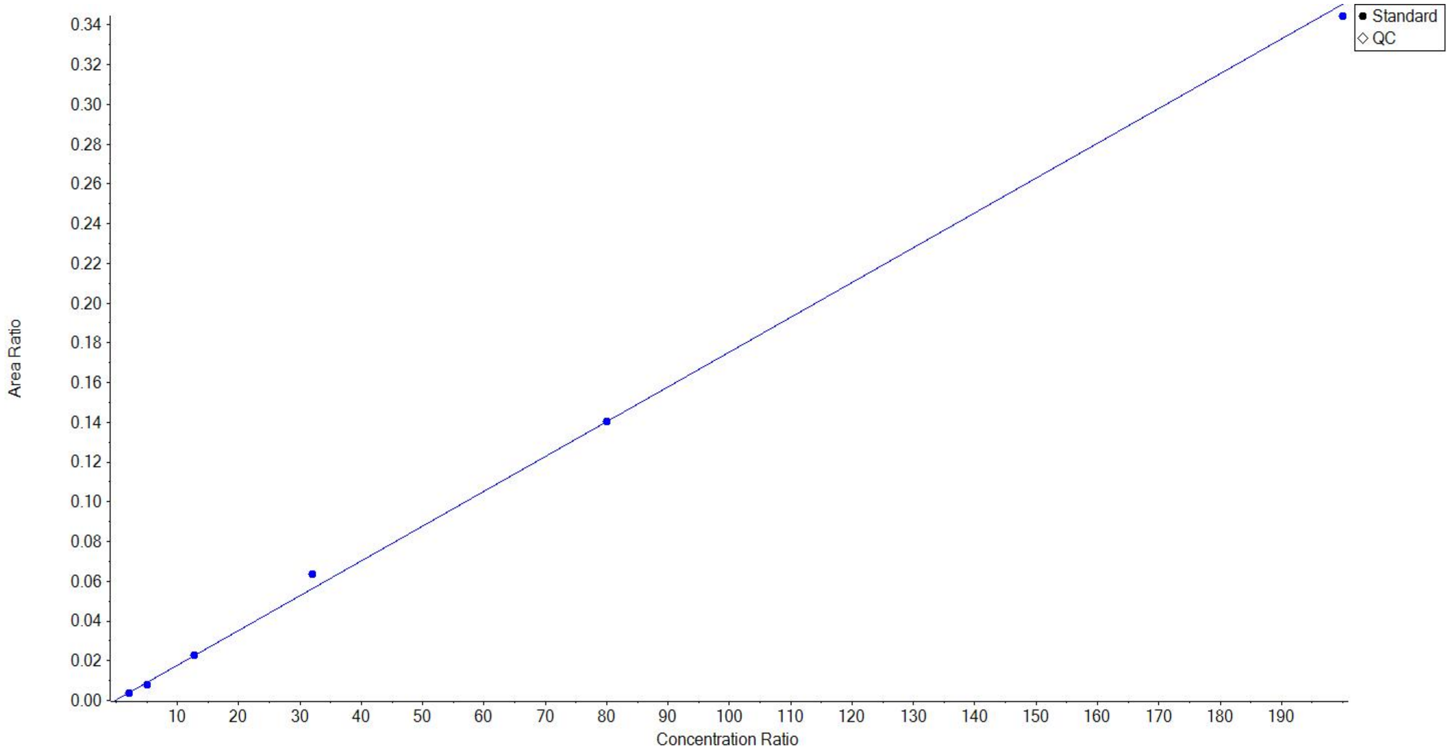
Standard
QC
Area Ratio
Concentration Ratio
0.34
0.32
0.30
0.28
0.26
0.24
0.22
0.20
0.18
0.16
0.14
0.12
0.10
0.08
0.06
0.04
0.02
0.00
10
20
30
40
50
60
70
80
90
100
110
120
130
140
150
160
170
180
190

Analyte Name: LM-flavones-88_1
Internal Standard: LM-flavones-IS02_1

**Data File**	flavones-STD-20230908.wiff	**Result Table**	DZLM2023082419-results-20230913-5500
**Acquisition Date**	9/8/2023 5:37:08 PM	**Algorithm Used**	MQ4
**Acquisition Method**	20230908-flavones-(mix130-T3)-15min.dam	**Instrument Name**	QTRAP 6500+ Low Mass
**Project**	N/A	**Processing Method**	20230412-flavones-(mix130-T3)-15min.qmethod

Regression Equation: y = 0.00116 x + -9.64359e-5 (r = 0.99970, r² = 0.99940) (weighting: 1 / x)

Expected Concentration	Number of Values	Mean Calculated Concentration (No data for Analyte Unit)	% Accuracy	Std. Deviation	%CV
0.01	0 of 1	N/A	N/A	N/A	N/A
0.02	0 of 1	N/A	N/A	N/A	N/A
0.05	0 of 1	N/A	N/A	N/A	N/A
0.13	0 of 1	N/A	N/A	N/A	N/A
0.33	0 of 1	N/A	N/A	N/A	N/A
0.82	0 of 1	N/A	N/A	N/A	N/A
2.05	1 of 1	2.214e0	108.0	N/A	N/A
5.12	1 of 1	5.059e0	98.8	N/A	N/A
12.80	1 of 1	1.200e1	93.8	N/A	N/A
32.00	1 of 1	3.233e1	101.0	N/A	N/A
80.00	1 of 1	7.764e1	97.1	N/A	N/A
200.00	1 of 1	2.027e2	101.4	N/A	N/A

Analyte Name: LM-flavones-88_1

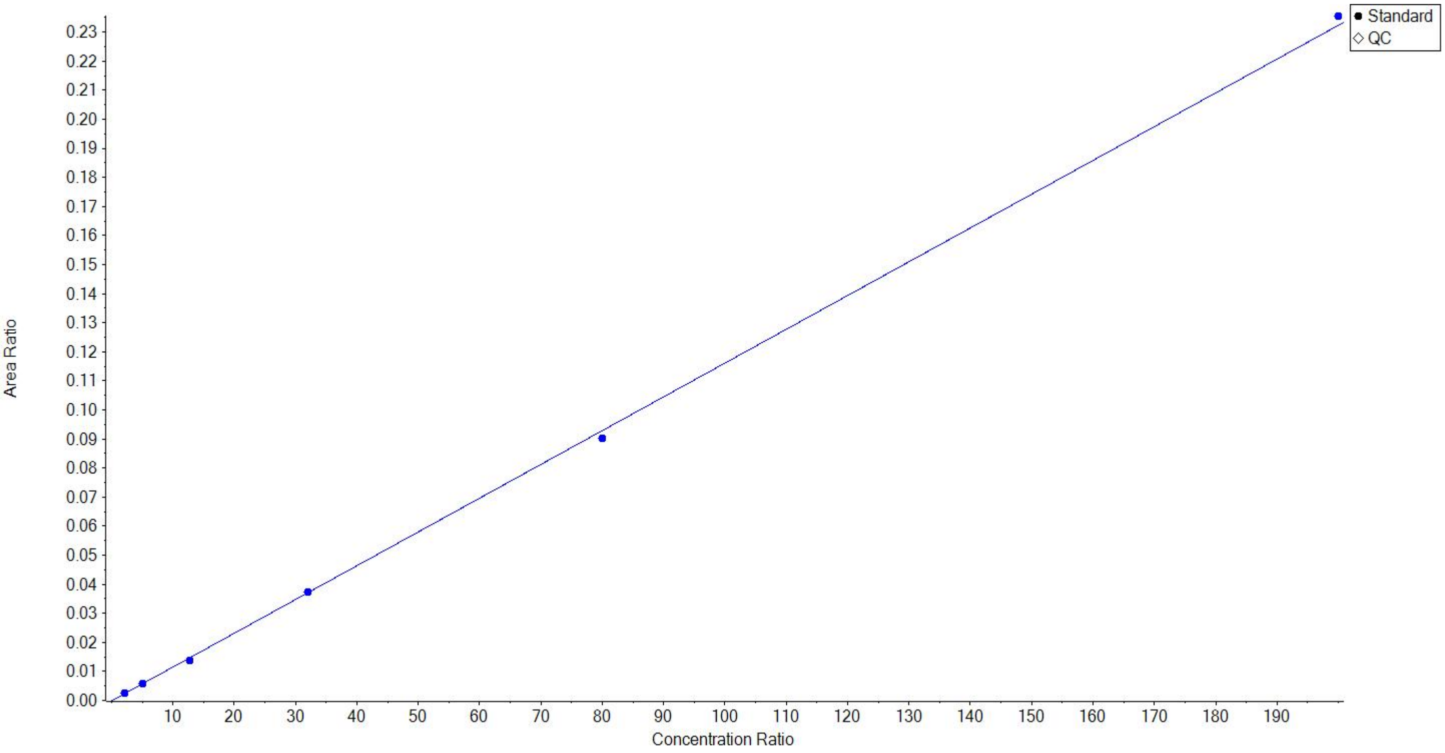
Standard
QC
Area Ratio
0.23
0.22
0.21
0.20
0.19
0.18
0.17
0.16
0.15
0.14
0.13
0.12
0.11
0.10
0.09
0.08
0.07
0.06
0.05
0.04
0.03
0.02
0.01
0.00
10
20
30
40
50
60
70
80
90
100
110
120
130
140
150
160
170
180
190
Concentration Ratio

Created with SCIEX OS 1.6.1
Printed:9/18/2023 5:27:01 PM

Analyte Name: LM-flavones-89_1
Internal Standard: LM-flavones-IS02_1

**Data File**	flavones-STD-20230908.wiff	**Result Table**	DZLM2023082419-results-20230913-5500
**Acquisition Date**	9/8/2023 5:37:08 PM	**Algorithm Used**	MQ4
**Acquisition Method**	20230908-flavones-(mix130-T3)-15min.dam	**Instrument Name**	QTRAP 6500+ Low Mass
**Project**	N/A	**Processing Method**	20230412-flavones-(mix130-T3)-15min.qmethod

Regression Equation: y = 0.00112 x + -4.22913e-4 (r = 0.99853, r² = 0.99707) (weighting: 1 / x)

Expected Concentration	Number of Values	Mean Calculated Concentration (No data for Analyte Unit)	% Accuracy	Std. Deviation	%CV
0.01	0 of 1	N/A	N/A	N/A	N/A
0.02	0 of 1	N/A	N/A	N/A	N/A
0.05	0 of 1	N/A	N/A	N/A	N/A
0.13	0 of 1	N/A	N/A	N/A	N/A
0.33	0 of 1	N/A	N/A	N/A	N/A
0.82	0 of 1	N/A	N/A	N/A	N/A
2.05	1 of 1	1.770e0	86.4	N/A	N/A
5.12	1 of 1	5.625e0	109.9	N/A	N/A
12.80	1 of 1	1.292e1	100.9	N/A	N/A
32.00	1 of 1	3.488e1	109.0	N/A	N/A
80.00	1 of 1	7.396e1	92.4	N/A	N/A
200.00	1 of 1	2.028e2	101.4	N/A	N/A

Analyte Name: LM-flavones-89_1

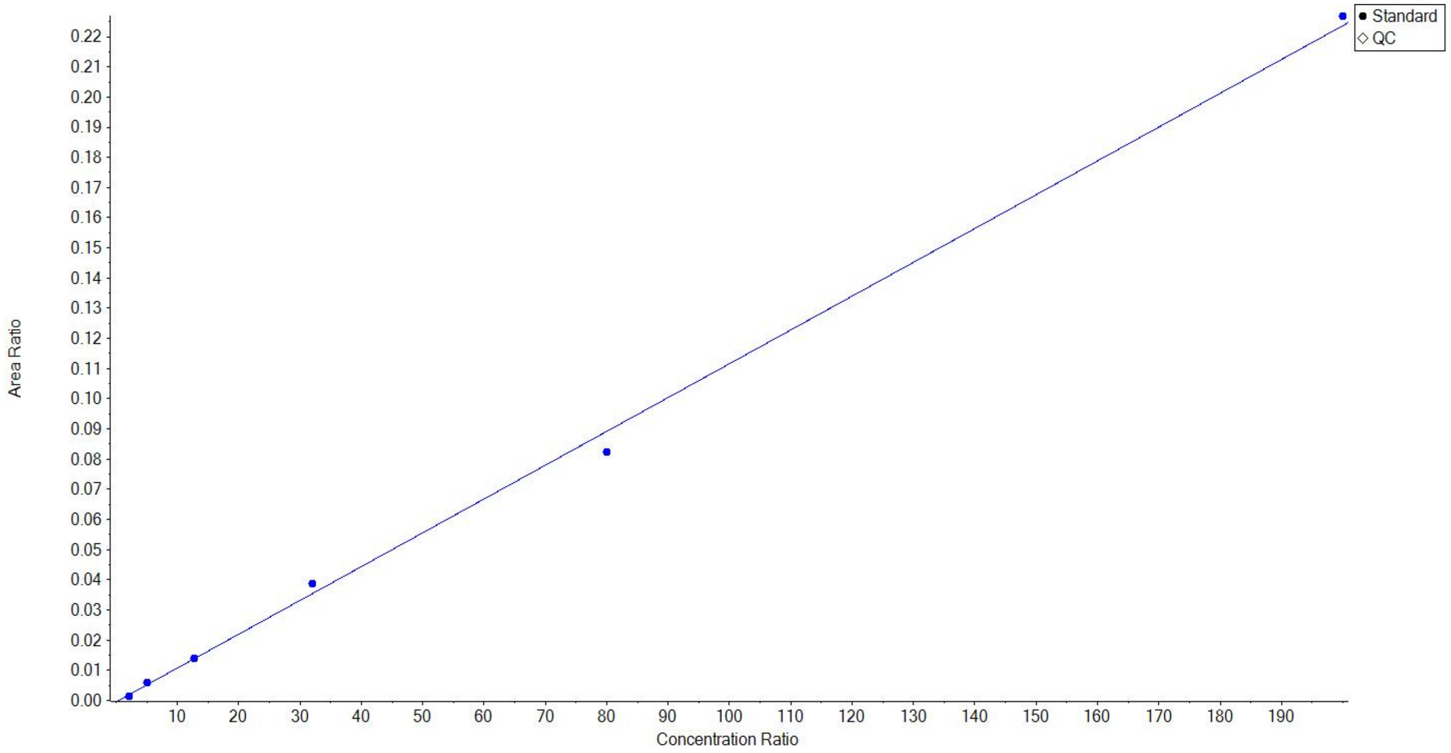
Standard
QC
Area Ratio
0.22
0.21
0.20
0.19
0.18
0.17
0.16
0.15
0.14
0.13
0.12
0.11
0.10
0.09
0.08
0.07
0.06
0.05
0.04
0.03
0.02
0.01
0.00
10
20
30
40
50
60
70
80
90
100
110
120
130
140
150
160
170
180
190
Concentration Ratio

Created with SCIEX OS 1.6.1
Printed:9/18/2023 5:27:01 PM

Analyte Name: LM-flavones-90_1
Internal Standard: LM-flavones-IS02_1

**Data File**	flavones-STD-20230908.wiff	**Result Table**	DZLM2023082419-results-20230913-5500
**Acquisition Date**	9/8/2023 5:37:08 PM	**Algorithm Used**	MQ4
**Acquisition Method**	20230908-flavones-(mix130-T3)-15min.dam	**Instrument Name**	QTRAP 6500+ Low Mass
**Project**	N/A	**Processing Method**	20230412-flavones-(mix130-T3)-15min.qmethod

Regression Equation: y = 0.00237 x + 2.11026e-4 (r = 0.99979, r^2 = 0.99958) (weighting: 1 / x)

Expected Concentration	Number of Values	Mean Calculated Concentration (No data for Analyte Unit)	% Accuracy	Std. Deviation	%CV
0.01	0 of 1	N/A	N/A	N/A	N/A
0.02	0 of 1	N/A	N/A	N/A	N/A
0.05	0 of 1	N/A	N/A	N/A	N/A
0.13	0 of 1	N/A	N/A	N/A	N/A
0.33	0 of 1	N/A	N/A	N/A	N/A
0.82	0 of 1	N/A	N/A	N/A	N/A
2.05	1 of 1	2.098e0	102.3	N/A	N/A
5.12	1 of 1	4.866e0	95.0	N/A	N/A
12.80	1 of 1	1.268e1	99.0	N/A	N/A
32.00	1 of 1	3.270e1	102.2	N/A	N/A
80.00	1 of 1	8.216e1	102.7	N/A	N/A
200.00	1 of 1	1.975e2	98.7	N/A	N/A

Analyte Name: LM-flavones-90_1

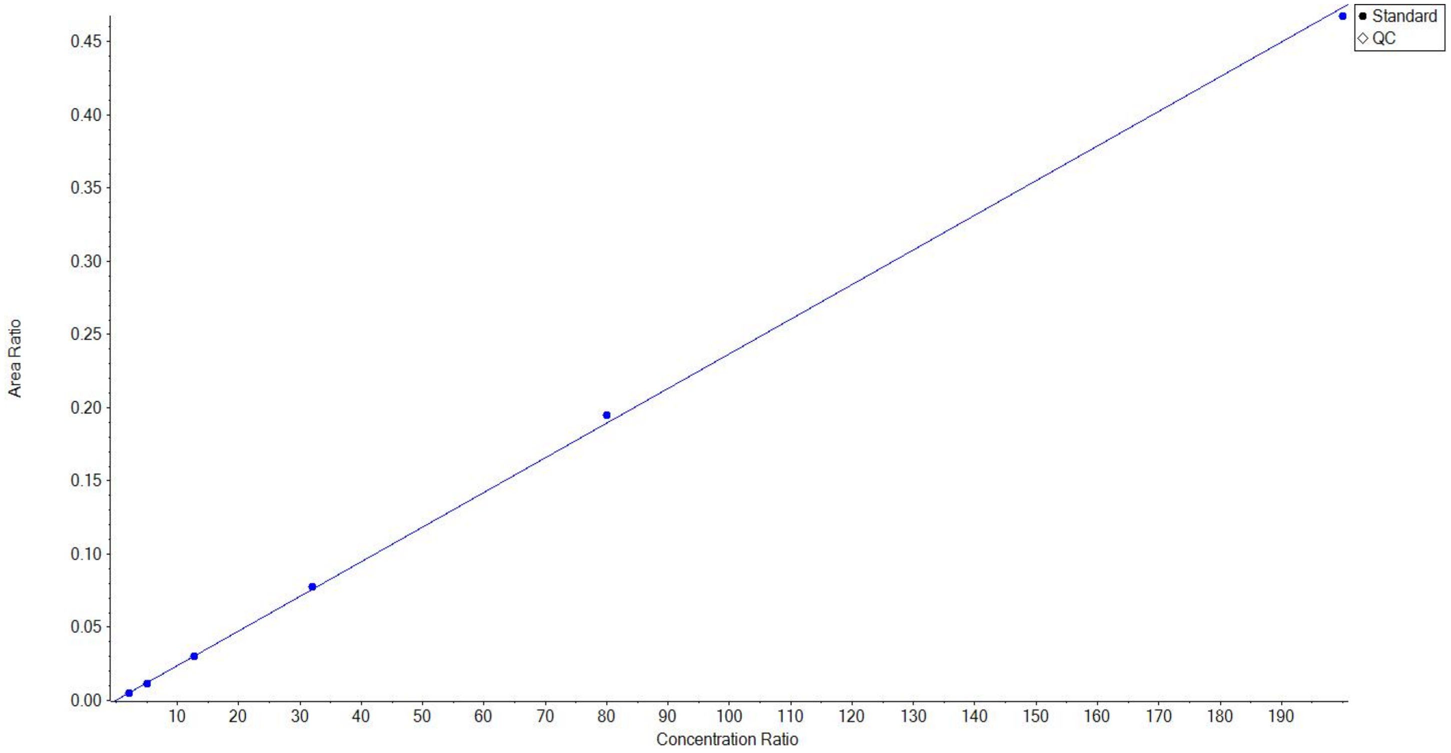
Standard
QC
Area Ratio
0.00
0.05
0.10
0.15
0.20
0.25
0.30
0.35
0.40
0.45
10
20
30
40
50
60
70
80
90
100
110
120
130
140
150
160
170
180
190
Concentration Ratio

Analyte Name: LM-flavones-93_1
Internal Standard: LM-flavones-IS02_1

**Data File**	flavones-STD-20230908.wiff	**Result Table**	DZLM2023082419-results-20230913-5500
**Acquisition Date**	9/8/2023 5:37:08 PM	**Algorithm Used**	MQ4
**Acquisition Method**	20230908-flavones-(mix130-T3)-15min.dam	**Instrument Name**	QTRAP 6500+ Low Mass
**Project**	N/A	**Processing Method**	20230412-flavones-(mix130-T3)-15min.qmethod

Regression Equation: y = 0.00111 x + 6.06145e-4 (r = 0.99863, r^2 = 0.99726) (weighting: 1 / x)

Expected Concentration	Number of Values	Mean Calculated Concentration (No data for Analyte Unit)	% Accuracy	Std. Deviation	%CV
0.01	0 of 1	N/A	N/A	N/A	N/A
0.02	0 of 1	N/A	N/A	N/A	N/A
0.05	0 of 1	N/A	N/A	N/A	N/A
0.13	0 of 1	N/A	N/A	N/A	N/A
0.33	0 of 1	N/A	N/A	N/A	N/A
0.82	0 of 1	N/A	N/A	N/A	N/A
2.05	1 of 1	2.004e0	97.7	N/A	N/A
5.12	1 of 1	4.805e0	93.9	N/A	N/A
12.80	1 of 1	1.443e1	112.7	N/A	N/A
32.00	1 of 1	2.925e1	91.4	N/A	N/A
80.00	1 of 1	8.470e1	105.9	N/A	N/A
200.00	1 of 1	1.968e2	98.4	N/A	N/A

Analyte Name: LM-flavones-93_1

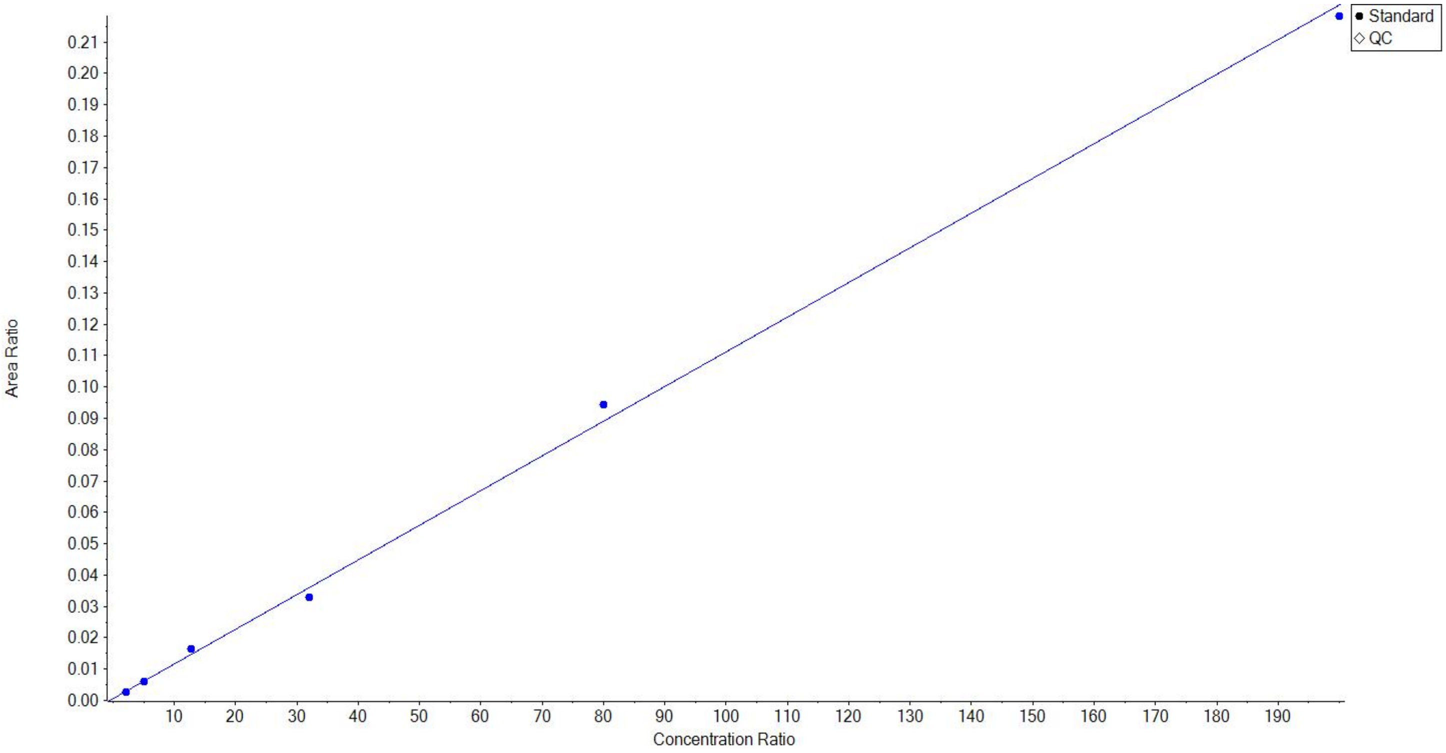
Standard
QC
Area Ratio
0.00
0.01
0.02
0.03
0.04
0.05
0.06
0.07
0.08
0.09
0.10
0.11
0.12
0.13
0.14
0.15
0.16
0.17
0.18
0.19
0.20
0.21
10
20
30
40
50
60
70
80
90
100
110
120
130
140
150
160
170
180
190
Concentration Ratio

Analyte Name: LM-flavones-96_1
Internal Standard: LM-flavones-IS02_1

**Data File**	flavones-STD-20230908.wiff	**Result Table**	DZLM2023082419-results-20230913-5500
**Acquisition Date**	9/8/2023 5:37:08 PM	**Algorithm Used**	MQ4
**Acquisition Method**	20230908-flavones-(mix130-T3)-15min.dam	**Instrument Name**	QTRAP 6500+ Low Mass
**Project**	N/A	**Processing Method**	20230412-flavones-(mix130-T3)-15min.qmethod

Regression Equation: y = 1.71665e-4 x + 4.99222e-5 (r = 0.99605, r² = 0.99212) (weighting: 1 / x)

Expected Concentration	Number of Values	Mean Calculated Concentration (No data for Analyte Unit)	% Accuracy	Std. Deviation	%CV
0.01	0 of 1	N/A	N/A	N/A	N/A
0.02	0 of 1	N/A	N/A	N/A	N/A
0.05	0 of 1	N/A	N/A	N/A	N/A
0.13	0 of 1	N/A	N/A	N/A	N/A
0.33	0 of 1	N/A	N/A	N/A	N/A
0.82	1 of 1	8.536e-1	104.2	N/A	N/A
2.05	1 of 1	2.304e0	112.5	N/A	N/A
5.12	1 of 1	5.249e0	102.5	N/A	N/A
12.80	1 of 1	1.101e1	86.0	N/A	N/A
32.00	1 of 1	2.828e1	88.4	N/A	N/A
80.00	1 of 1	8.513e1	106.4	N/A	N/A
200.00	0 of 1	N/A	N/A	N/A	N/A

Analyte Name: LM-flavones-96_1

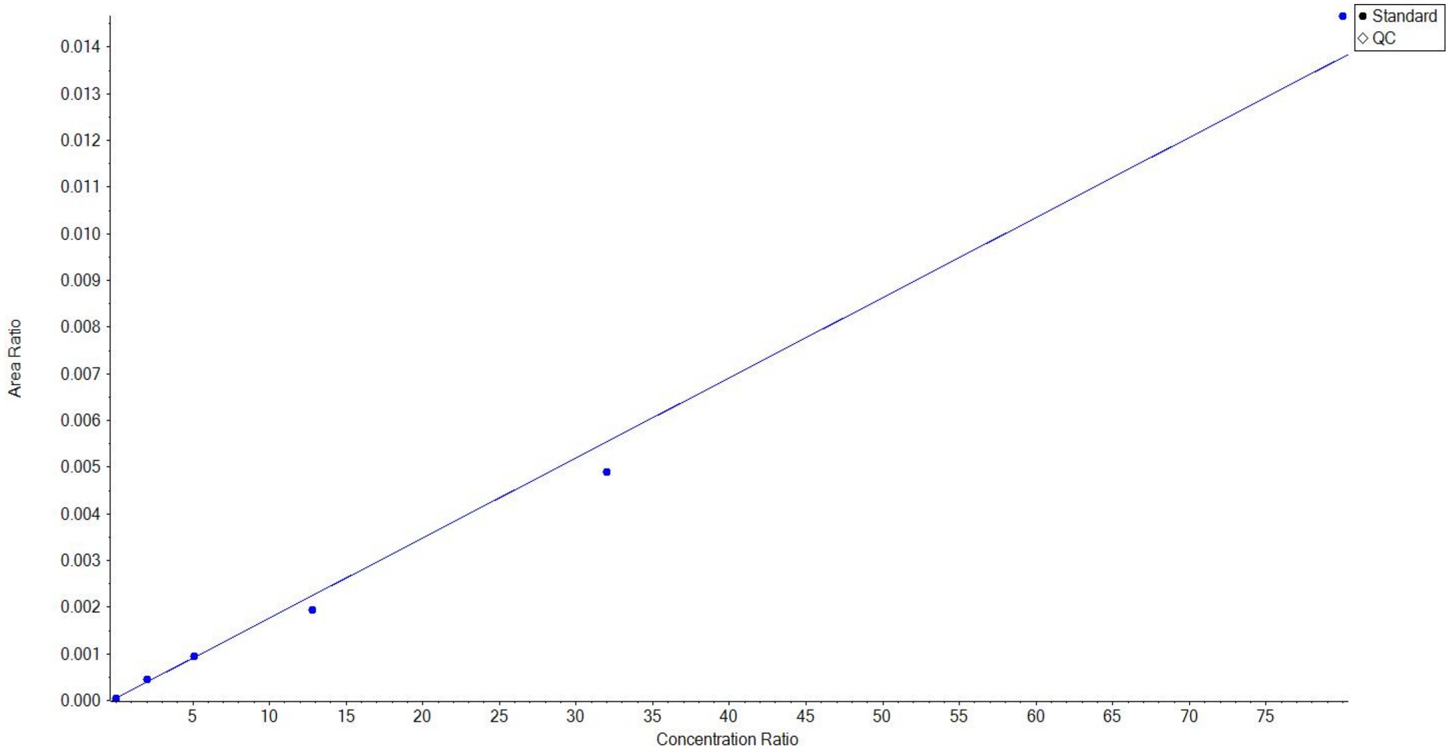
Standard
QC
0.014
0.013
0.012
0.011
0.010
0.009
0.008
0.007
0.006
0.005
0.004
0.003
0.002
0.001
0.000
Area Ratio
5
10
15
20
25
30
35
40
45
50
55
60
65
70
75
Concentration Ratio

Analyte Name: LM-flavones-97_1
Internal Standard: LM-flavones-IS02_1

**Data File**	flavones-STD-20230908.wiff	**Result Table**	DZLM2023082419-results-20230913-5500
**Acquisition Date**	9/8/2023 5:37:08 PM	**Algorithm Used**	MQ4
**Acquisition Method**	20230908-flavones-(mix130-T3)-15min.dam	**Instrument Name**	QTRAP 6500+ Low Mass
**Project**	N/A	**Processing Method**	20230412-flavones-(mix130-T3)-15min.qmethod

Regression Equation: y = 0.00804 x + -0.00563 (r = 0.99997, r² = 0.99994) (weighting: 1 / x)

Expected Concentration	Number of Values	Mean Calculated Concentration (No data for Analyte Unit)	% Accuracy	Std. Deviation	%CV
0.01	0 of 1	N/A	N/A	N/A	N/A
0.02	0 of 1	N/A	N/A	N/A	N/A
0.05	0 of 1	N/A	N/A	N/A	N/A
0.13	0 of 1	N/A	N/A	N/A	N/A
0.33	0 of 1	N/A	N/A	N/A	N/A
0.82	0 of 1	N/A	N/A	N/A	N/A
2.05	0 of 1	N/A	N/A	N/A	N/A
5.12	1 of 1	5.214e0	101.8	N/A	N/A
12.80	1 of 1	1.264e1	98.7	N/A	N/A
32.00	1 of 1	3.200e1	100.0	N/A	N/A
80.00	1 of 1	7.919e1	99.0	N/A	N/A
200.00	1 of 1	2.009e2	100.4	N/A	N/A

Analyte Name: LM-flavones-97_1

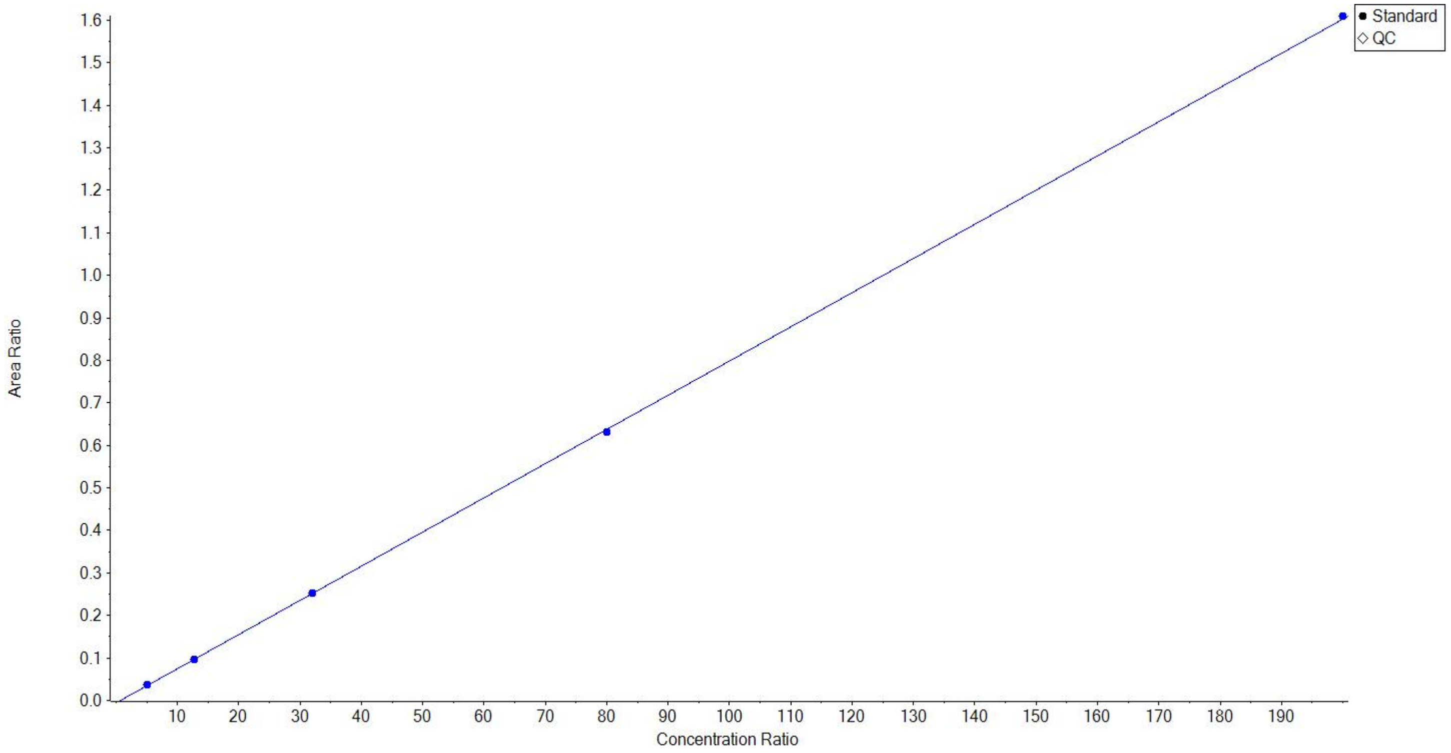
Standard
QC
Area Ratio
Concentration Ratio
1.6
1.5
1.4
1.3
1.2
1.1
1.0
0.9
0.8
0.7
0.6
0.5
0.4
0.3
0.2
0.1
0.0
10
20
30
40
50
60
70
80
90
100
110
120
130
140
150
160
170
180
190

Analyte Name: LM-flavones-98_1
Internal Standard: LM-flavones-IS02_1

**Data File**	flavones-STD-20230908.wiff	**Result Table**	DZLM2023082419-results-20230913-5500
**Acquisition Date**	9/8/2023 5:37:08 PM	**Algorithm Used**	MQ4
**Acquisition Method**	20230908-flavones-(mix130-T3)-15min.dam	**Instrument Name**	QTRAP 6500+ Low Mass
**Project**	N/A	**Processing Method**	20230412-flavones-(mix130-T3)-15min.qmethod

Regression Equation: y = 0.00552 x + -0.00166 (r = 0.99942, r² = 0.99883) (weighting: 1 / x)

Expected Concentration	Number of Values	Mean Calculated Concentration (No data for Analyte Unit)	% Accuracy	Std. Deviation	%CV
0.01	0 of 1	N/A	N/A	N/A	N/A
0.02	0 of 1	N/A	N/A	N/A	N/A
0.05	0 of 1	N/A	N/A	N/A	N/A
0.13	0 of 1	N/A	N/A	N/A	N/A
0.33	0 of 1	N/A	N/A	N/A	N/A
0.82	0 of 1	N/A	N/A	N/A	N/A
2.05	1 of 1	2.325e0	113.4	N/A	N/A
5.12	1 of 1	4.841e0	94.5	N/A	N/A
12.80	1 of 1	1.228e1	95.9	N/A	N/A
32.00	1 of 1	3.140e1	98.1	N/A	N/A
80.00	1 of 1	7.656e1	95.7	N/A	N/A
200.00	1 of 1	2.046e2	102.3	N/A	N/A

Analyte Name: LM-flavones-98_1

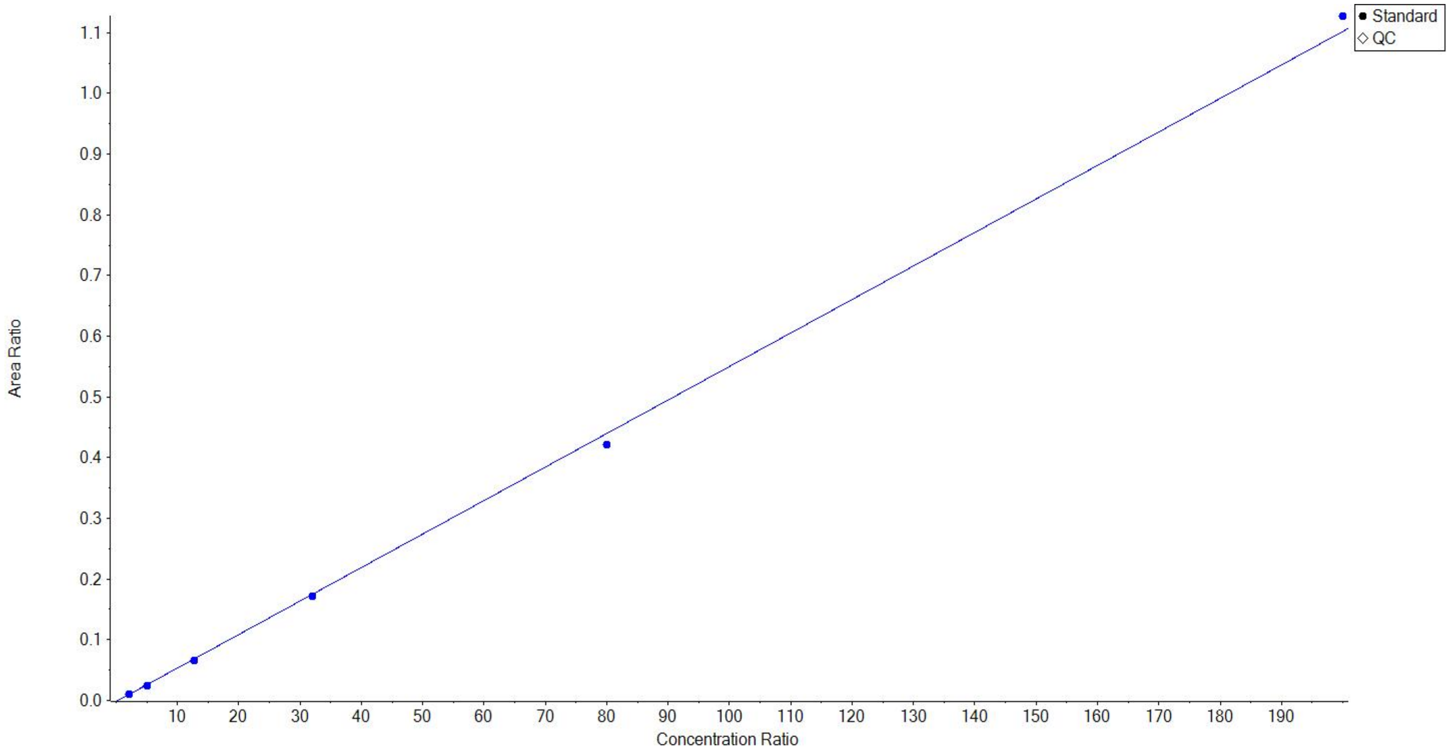
Standard
QC
Area Ratio
Concentration Ratio
1.1
1.0
0.9
0.8
0.7
0.6
0.5
0.4
0.3
0.2
0.1
0.0
10
20
30
40
50
60
70
80
90
100
110
120
130
140
150
160
170
180
190

Analyte Name: LM-flavones-99_1
Internal Standard: LM-flavones-IS02_1

**Data File**	flavones-STD-20230908.wiff	**Result Table**	DZLM2023082419-results-20230913-5500
**Acquisition Date**	9/8/2023 5:37:08 PM	**Algorithm Used**	MQ4
**Acquisition Method**	20230908-flavones-(mix130-T3)-15min.dam	**Instrument Name**	QTRAP 6500+ Low Mass
**Project**	N/A	**Processing Method**	20230412-flavones-(mix130-T3)-15min.qmethod

Regression Equation: y = 1.72083e-4 x + 3.17030e-7 (r = 0.99704, r^2 = 0.99409) (weighting: 1 / x)

Expected Concentration	Number of Values	Mean Calculated Concentration (No data for Analyte Unit)	% Accuracy	Std. Deviation	%CV
0.01	0 of 1	N/A	N/A	N/A	N/A
0.02	0 of 1	N/A	N/A	N/A	N/A
0.05	0 of 1	N/A	N/A	N/A	N/A
0.13	0 of 1	N/A	N/A	N/A	N/A
0.33	0 of 1	N/A	N/A	N/A	N/A
0.82	0 of 1	N/A	N/A	N/A	N/A
2.05	1 of 1	2.141e0	104.5	N/A	N/A
5.12	1 of 1	5.380e0	105.1	N/A	N/A
12.80	1 of 1	1.341e1	104.8	N/A	N/A
32.00	1 of 1	2.909e1	90.9	N/A	N/A
80.00	1 of 1	7.166e1	89.6	N/A	N/A
200.00	1 of 1	2.103e2	105.2	N/A	N/A

Analyte Name: LM-flavones-99_1

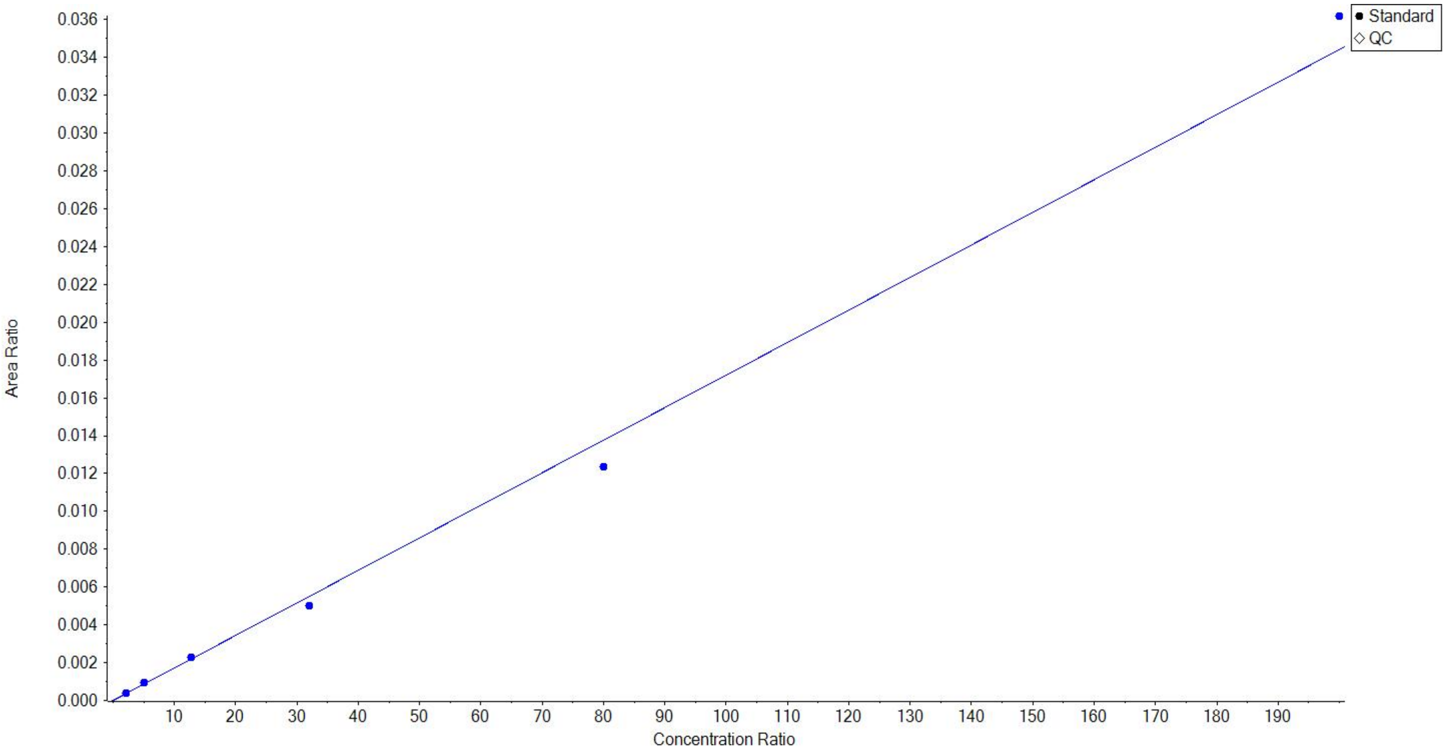
0.036
0.034
0.032
0.030
0.028
0.026
0.024
0.022
0.020
0.018
0.016
0.014
0.012
0.010
0.008
0.006
0.004
0.002
0.000
Area Ratio
10 20 30 40 50 60 70 80 90 100 110 120 130 140 150 160 170 180 190
Concentration Ratio
Standard
QC

Analyte Name: LM-flavones-100_1
Internal Standard: LM-flavones-IS02_1

**Data File**	flavones-STD-20230908.wiff	**Result Table**	DZLM2023082419-results-20230913-5500
**Acquisition Date**	9/8/2023 5:37:08 PM	**Algorithm Used**	MQ4
**Acquisition Method**	20230908-flavones-(mix130-T3)-15min.dam	**Instrument Name**	QTRAP 6500+ Low Mass
**Project**	N/A	**Processing Method**	20230412-flavones-(mix130-T3)-15min.qmethod

Regression Equation: y = 5.07483e-4 x + -8.33250e-5 (r = 0.99967, r^2 = 0.99934) (weighting: 1 / x)

Expected Concentration	Number of Values	Mean Calculated Concentration (No data for Analyte Unit)	% Accuracy	Std. Deviation	%CV
0.01	0 of 1	N/A	N/A	N/A	N/A
0.02	0 of 1	N/A	N/A	N/A	N/A
0.05	0 of 1	N/A	N/A	N/A	N/A
0.13	0 of 1	N/A	N/A	N/A	N/A
0.33	0 of 1	N/A	N/A	N/A	N/A
0.82	0 of 1	N/A	N/A	N/A	N/A
2.05	1 of 1	1.954e0	95.3	N/A	N/A
5.12	1 of 1	5.481e0	107.1	N/A	N/A
12.80	1 of 1	1.220e1	95.3	N/A	N/A
32.00	1 of 1	3.345e1	104.5	N/A	N/A
80.00	1 of 1	7.781e1	97.3	N/A	N/A
200.00	1 of 1	2.011e2	100.5	N/A	N/A

Analyte Name: LM-flavones-100_1

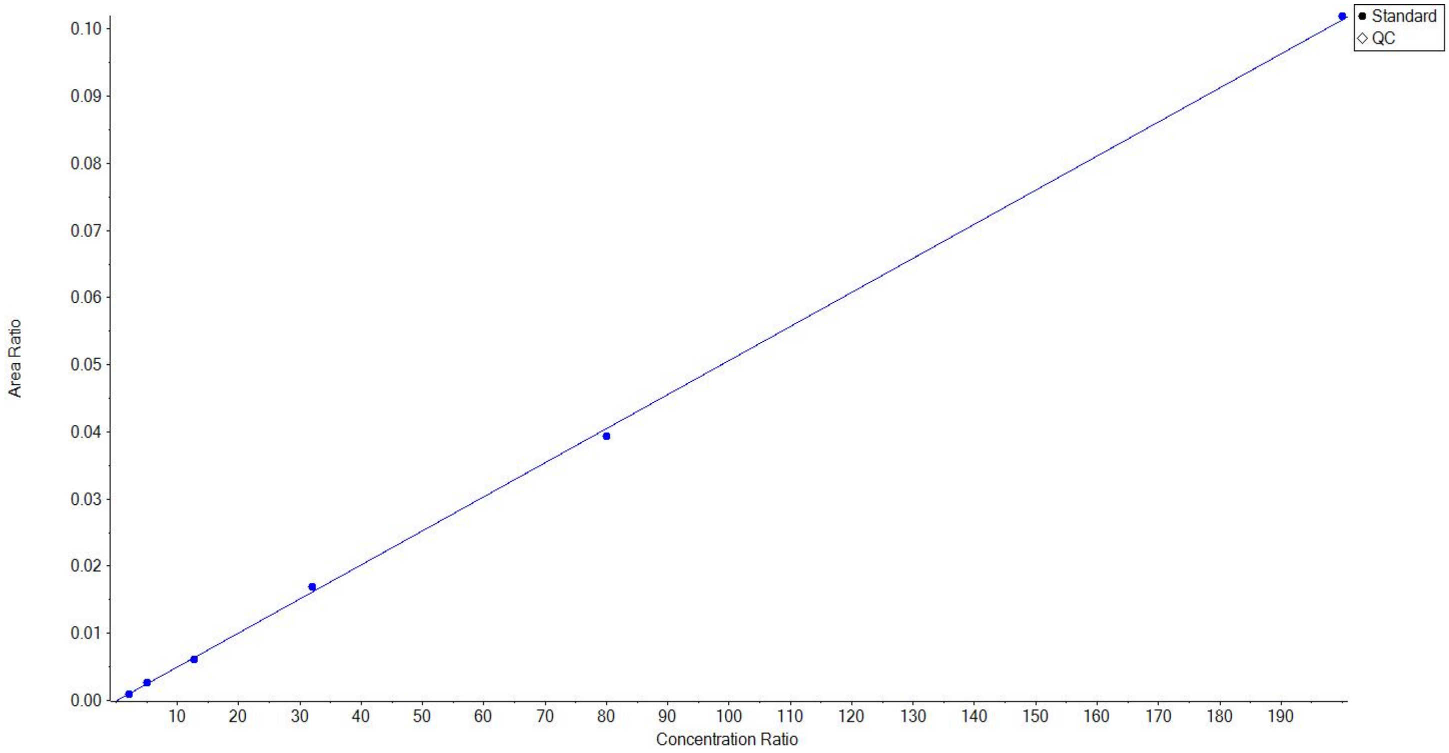
Standard
QC
Area Ratio
0.10
0.09
0.08
0.07
0.06
0.05
0.04
0.03
0.02
0.01
0.00
10
20
30
40
50
60
70
80
90
100
110
120
130
140
150
160
170
180
190
Concentration Ratio

Analyte Name: LM-flavones-101_1
Internal Standard: LM-flavones-IS02_1

**Data File**	flavones-STD-20230908.wiff	**Result Table**	DZLM2023082419-results-20230913-5500
**Acquisition Date**	9/8/2023 5:37:08 PM	**Algorithm Used**	MQ4
**Acquisition Method**	20230908-flavones-(mix130-T3)-15min.dam	**Instrument Name**	QTRAP 6500+ Low Mass
**Project**	N/A	**Processing Method**	20230412-flavones-(mix130-T3)-15min.qmethod

Regression Equation: y = 7.64703e-4 x + 8.36407e-5 (r = 0.99988, r^2 = 0.99977) (weighting: 1 / x)

Expected Concentration	Number of Values	Mean Calculated Concentration (No data for Analyte Unit)	% Accuracy	Std. Deviation	%CV
0.01	0 of 1	N/A	N/A	N/A	N/A
0.02	0 of 1	N/A	N/A	N/A	N/A
0.05	0 of 1	N/A	N/A	N/A	N/A
0.13	0 of 1	N/A	N/A	N/A	N/A
0.33	0 of 1	N/A	N/A	N/A	N/A
0.82	0 of 1	N/A	N/A	N/A	N/A
2.05	1 of 1	2.218e0	108.3	N/A	N/A
5.12	1 of 1	4.688e0	91.6	N/A	N/A
12.80	1 of 1	1.290e1	100.8	N/A	N/A
32.00	1 of 1	3.200e1	100.0	N/A	N/A
80.00	1 of 1	7.913e1	98.9	N/A	N/A
200.00	1 of 1	2.010e2	100.5	N/A	N/A

Analyte Name: LM-flavones-101_1

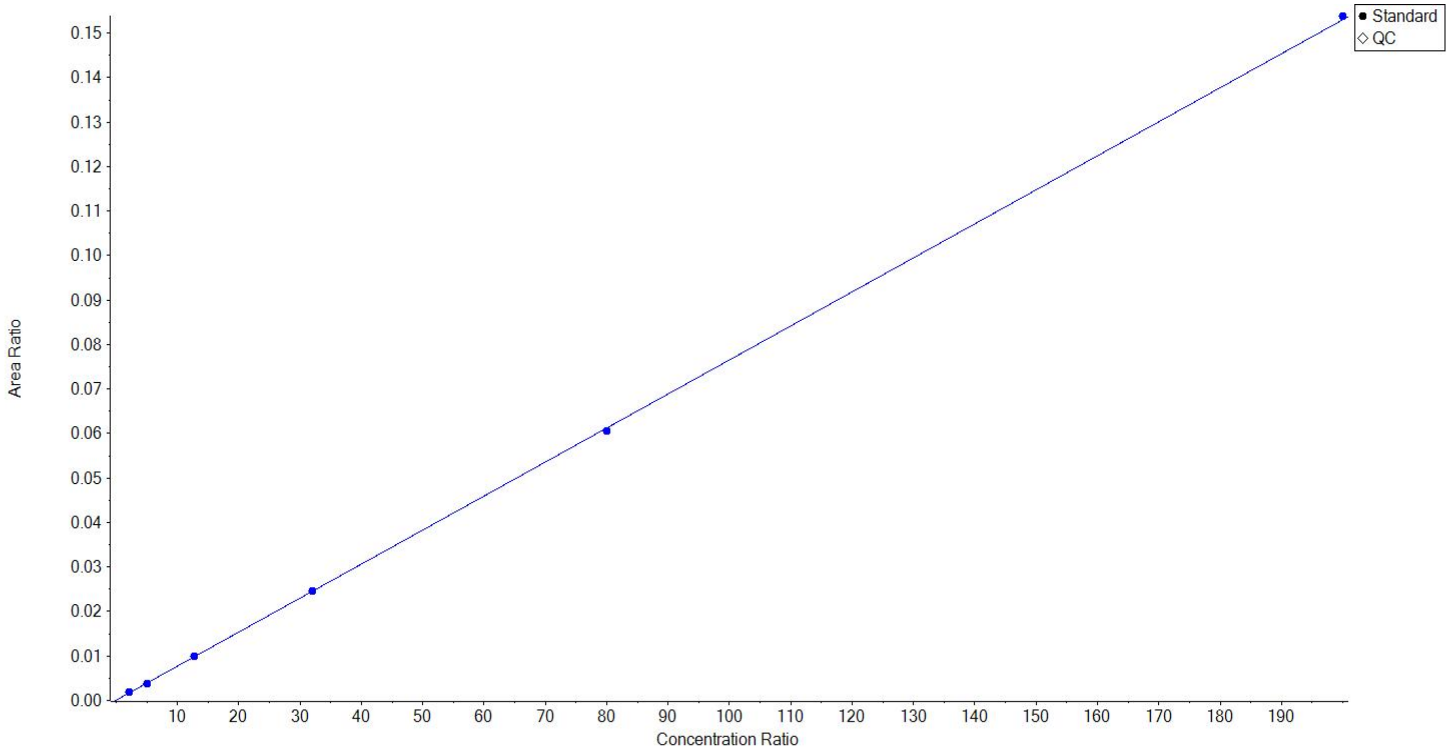
Area Ratio
0.15
0.14
0.13
0.12
0.11
0.10
0.09
0.08
0.07
0.06
0.05
0.04
0.03
0.02
0.01
0.00
10 20 30 40 50 60 70 80 90 100 110 120 130 140 150 160 170 180 190
Concentration Ratio
Standard
QC

Analyte Name: LM-flavones-102_1
Internal Standard: LM-flavones-IS02_1

**Data File**	flavones-STD-20230908.wiff	**Result Table**	DZLM2023082419-results-20230913-5500
**Acquisition Date**	9/8/2023 5:37:08 PM	**Algorithm Used**	MQ4
**Acquisition Method**	20230908-flavones-(mix130-T3)-15min.dam	**Instrument Name**	QTRAP 6500+ Low Mass
**Project**	N/A	**Processing Method**	20230412-flavones-(mix130-T3)-15min.qmethod

Regression Equation: y = 3.11269e-4 x + 2.64444e-4 (r = 0.99908, r² = 0.99816) (weighting: 1 / x)

Expected Concentration	Number of Values	Mean Calculated Concentration (No data for Analyte Unit)	% Accuracy	Std. Deviation	%CV
0.01	0 of 1	N/A	N/A	N/A	N/A
0.02	0 of 1	N/A	N/A	N/A	N/A
0.05	0 of 1	N/A	N/A	N/A	N/A
0.13	0 of 1	N/A	N/A	N/A	N/A
0.33	0 of 1	N/A	N/A	N/A	N/A
0.82	0 of 1	N/A	N/A	N/A	N/A
2.05	0 of 1	N/A	N/A	N/A	N/A
5.12	1 of 1	5.085e0	99.3	N/A	N/A
12.80	1 of 1	1.339e1	104.6	N/A	N/A
32.00	1 of 1	3.207e1	100.2	N/A	N/A
80.00	1 of 1	7.484e1	93.5	N/A	N/A
200.00	1 of 1	2.045e2	102.3	N/A	N/A

Analyte Name: LM-flavones-102_1

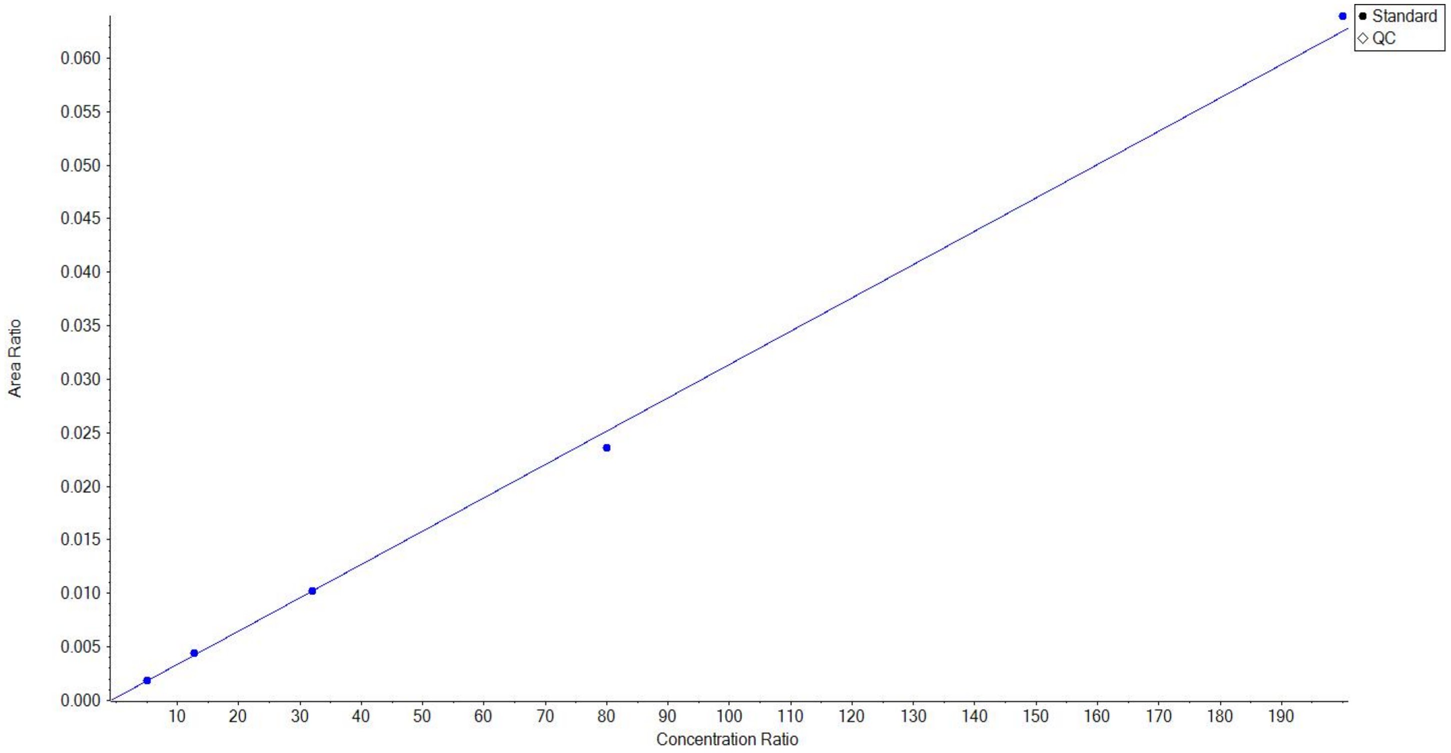
Standard
QC
Area Ratio
0.060
0.055
0.050
0.045
0.040
0.035
0.030
0.025
0.020
0.015
0.010
0.005
0.000
10
20
30
40
50
60
70
80
90
100
110
120
130
140
150
160
170
180
190
Concentration Ratio

Analyte Name: LM-flavones-103_1
Internal Standard: LM-flavones-IS02_1

**Data File**	flavones-STD-20230908.wiff	**Result Table**	DZLM2023082419-results-20230913-5500
**Acquisition Date**	9/8/2023 5:37:08 PM	**Algorithm Used**	MQ4
**Acquisition Method**	20230908-flavones-(mix130-T3)-15min.dam	**Instrument Name**	QTRAP 6500+ Low Mass
**Project**	N/A	**Processing Method**	20230412-flavones-(mix130-T3)-15min.qmethod

Regression Equation: y = 0.00843 x + -0.00104 (r = 0.99970, r^2 = 0.99939) (weighting: 1 / x)

Expected Concentration	Number of Values	Mean Calculated Concentration (No data for Analyte Unit)	% Accuracy	Std. Deviation	%CV
0.01	0 of 1	N/A	N/A	N/A	N/A
0.02	0 of 1	N/A	N/A	N/A	N/A
0.05	0 of 1	N/A	N/A	N/A	N/A
0.13	0 of 1	N/A	N/A	N/A	N/A
0.33	0 of 1	N/A	N/A	N/A	N/A
0.82	0 of 1	N/A	N/A	N/A	N/A
2.05	1 of 1	2.051e0	100.1	N/A	N/A
5.12	1 of 1	4.976e0	97.2	N/A	N/A
12.80	1 of 1	1.271e1	99.3	N/A	N/A
32.00	1 of 1	3.389e1	105.9	N/A	N/A
80.00	1 of 1	7.786e1	97.3	N/A	N/A
200.00	1 of 1	2.005e2	100.3	N/A	N/A

Analyte Name: LM-flavones-103_1

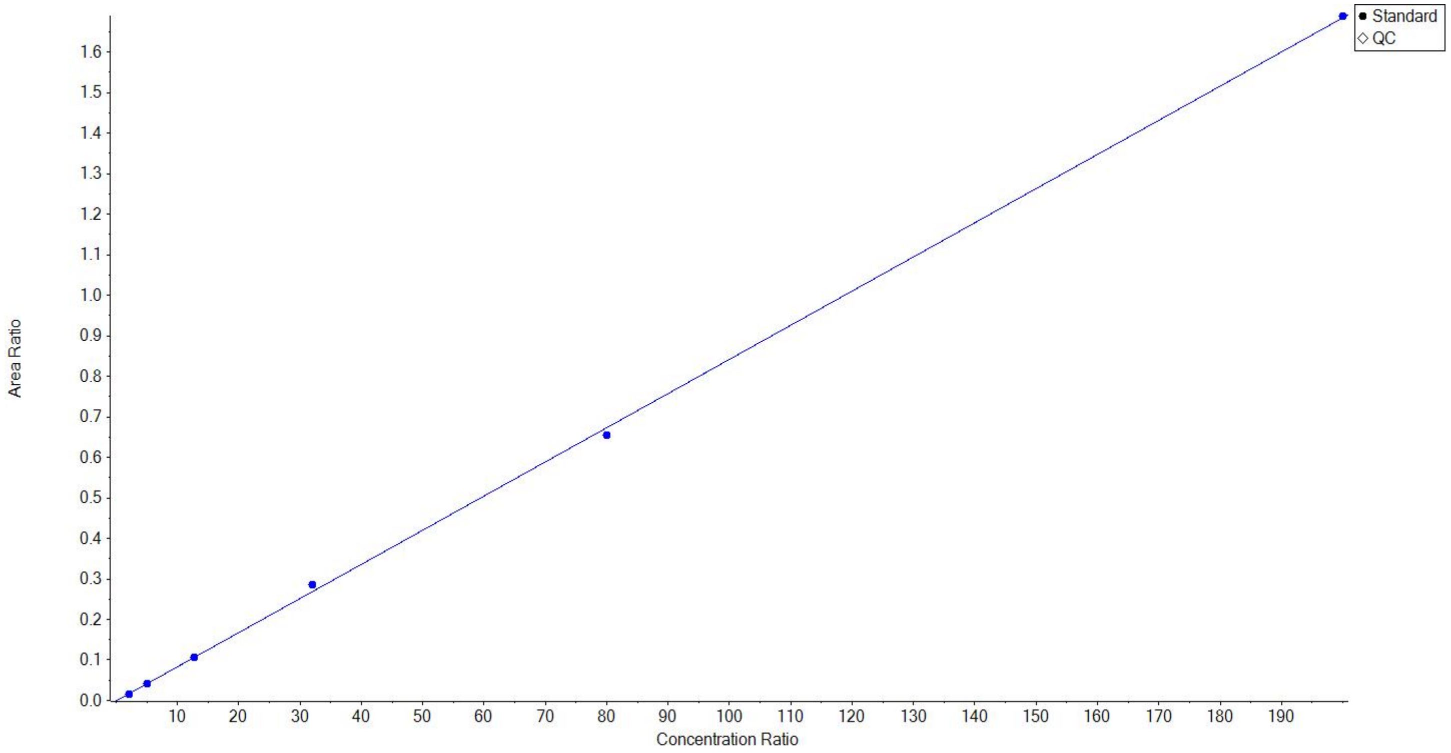
Standard
QC
Area Ratio
Concentration Ratio
0.0
0.1
0.2
0.3
0.4
0.5
0.6
0.7
0.8
0.9
1.0
1.1
1.2
1.3
1.4
1.5
1.6
10
20
30
40
50
60
70
80
90
100
110
120
130
140
150
160
170
180
190

Analyte Name: LM-flavones-104_1
Internal Standard: LM-flavones-IS02_1

**Data File**	flavones-STD-20230908.wiff	**Result Table**	DZLM2023082419-results-20230913-5500
**Acquisition Date**	9/8/2023 5:37:08 PM	**Algorithm Used**	MQ4
**Acquisition Method**	20230908-flavones-(mix130-T3)-15min.dam	**Instrument Name**	QTRAP 6500+ Low Mass
**Project**	N/A	**Processing Method**	20230412-flavones-(mix130-T3)-15min.qmethod

Regression Equation: y = 0.00489 x + -0.00122 (r = 0.99914, r² = 0.99829) (weighting: 1 / x)

Expected Concentration	Number of Values	Mean Calculated Concentration (No data for Analyte Unit)	% Accuracy	Std. Deviation	%CV
0.01	0 of 1	N/A	N/A	N/A	N/A
0.02	0 of 1	N/A	N/A	N/A	N/A
0.05	0 of 1	N/A	N/A	N/A	N/A
0.13	0 of 1	N/A	N/A	N/A	N/A
0.33	0 of 1	N/A	N/A	N/A	N/A
0.82	0 of 1	N/A	N/A	N/A	N/A
2.05	1 of 1	2.295e0	111.9	N/A	N/A
5.12	1 of 1	4.677e0	91.4	N/A	N/A
12.80	1 of 1	1.265e1	98.9	N/A	N/A
32.00	1 of 1	3.253e1	101.6	N/A	N/A
80.00	1 of 1	7.508e1	93.9	N/A	N/A
200.00	1 of 1	2.047e2	102.4	N/A	N/A

Analyte Name: LM-flavones-104_1

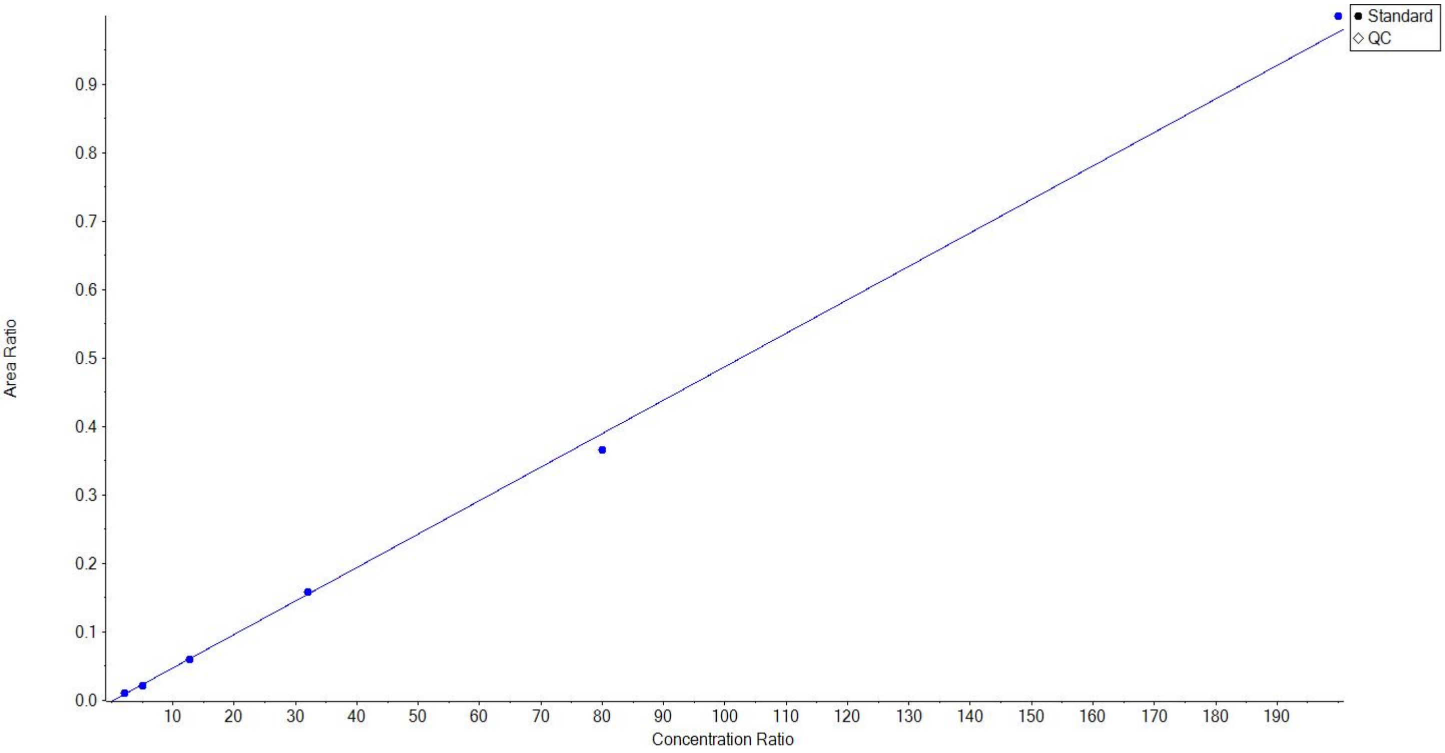
Standard
QC
Area Ratio
0.0
0.1
0.2
0.3
0.4
0.5
0.6
0.7
0.8
0.9
10
20
30
40
50
60
70
80
90
100
110
120
130
140
150
160
170
180
190
Concentration Ratio

Analyte Name: LM-flavones-106_1
Internal Standard: LM-flavones-IS02_1

**Data File**	flavones-STD-20230908.wiff	**Result Table**	DZLM2023082419-results-20230913-5500
**Acquisition Date**	9/8/2023 5:37:08 PM	**Algorithm Used**	MQ4
**Acquisition Method**	20230908-flavones-(mix130-T3)-15min.dam	**Instrument Name**	QTRAP 6500+ Low Mass
**Project**	N/A	**Processing Method**	20230412-flavones-(mix130-T3)-15min.qmethod

Regression Equation: y = 0.00209 x + -0.00375 (r = 0.99847, r² = 0.99694) (weighting: 1 / x)

Expected Concentration	Number of Values	Mean Calculated Concentration (No data for Analyte Unit)	% Accuracy	Std. Deviation	%CV
0.01	0 of 1	N/A	N/A	N/A	N/A
0.02	0 of 1	N/A	N/A	N/A	N/A
0.05	0 of 1	N/A	N/A	N/A	N/A
0.13	0 of 1	N/A	N/A	N/A	N/A
0.33	0 of 1	N/A	N/A	N/A	N/A
0.82	0 of 1	N/A	N/A	N/A	N/A
2.05	0 of 1	N/A	N/A	N/A	N/A
5.12	1 of 1	5.747e0	112.3	N/A	N/A
12.80	1 of 1	1.232e1	96.2	N/A	N/A
32.00	1 of 1	3.032e1	94.7	N/A	N/A
80.00	1 of 1	7.467e1	93.3	N/A	N/A
200.00	1 of 1	2.069e2	103.4	N/A	N/A

Analyte Name: LM-flavones-106_1

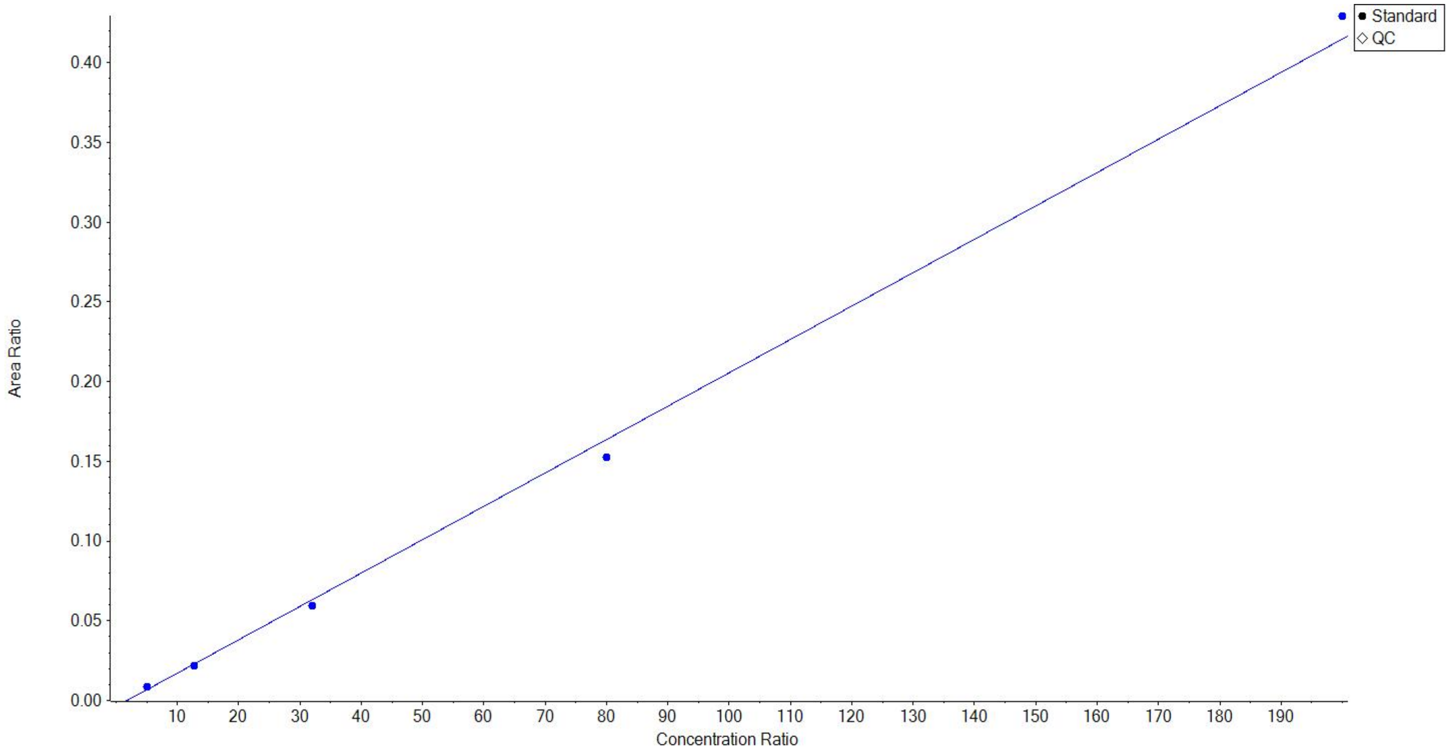
Standard
QC
Area Ratio
0.00
0.05
0.10
0.15
0.20
0.25
0.30
0.35
0.40
Concentration Ratio
10
20
30
40
50
60
70
80
90
100
110
120
130
140
150
160
170
180
190

Analyte Name: LM-flavones-108
Internal Standard: LM-flavones-IS02_1

**Data File**	flavones-STD-20230908.wiff	**Result Table**	DZLM2023082419-results-20230913-5500
**Acquisition Date**	9/8/2023 5:37:08 PM	**Algorithm Used**	MQ4
**Acquisition Method**	20230908-flavones-(mix130-T3)-15min.dam	**Instrument Name**	QTRAP 6500+ Low Mass
**Project**	N/A	**Processing Method**	20230412-flavones-(mix130-T3)-15min.qmethod

Regression Equation: y = 0.00152 x + 4.63242e-4 (r = 0.99968, r² = 0.99937) (weighting: 1 / x)

Expected Concentration	Number of Values	Mean Calculated Concentration (No data for Analyte Unit)	% Accuracy	Std. Deviation	%CV
0.01	0 of 1	N/A	N/A	N/A	N/A
0.02	0 of 1	N/A	N/A	N/A	N/A
0.05	0 of 1	N/A	N/A	N/A	N/A
0.13	0 of 1	N/A	N/A	N/A	N/A
0.33	0 of 1	N/A	N/A	N/A	N/A
0.82	0 of 1	N/A	N/A	N/A	N/A
2.05	1 of 1	2.102e0	102.5	N/A	N/A
5.12	1 of 1	4.501e0	87.9	N/A	N/A
12.80	1 of 1	1.377e1	107.6	N/A	N/A
32.00	1 of 1	3.293e1	102.9	N/A	N/A
80.00	1 of 1	7.970e1	99.6	N/A	N/A
200.00	1 of 1	1.990e2	99.5	N/A	N/A

Analyte Name: LM-flavones-108

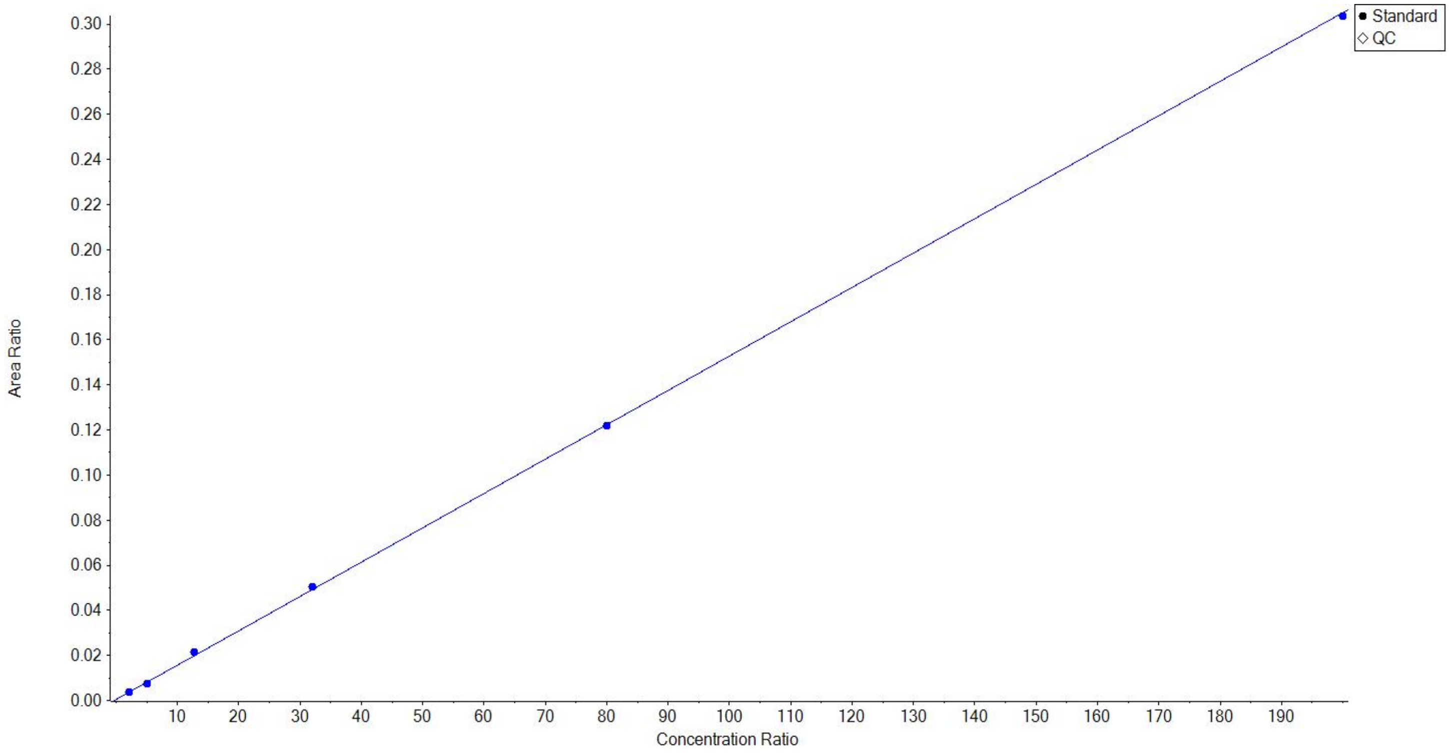
Standard
QC
Area Ratio
Concentration Ratio
0.00
0.02
0.04
0.06
0.08
0.10
0.12
0.14
0.16
0.18
0.20
0.22
0.24
0.26
0.28
0.30
10
20
30
40
50
60
70
80
90
100
110
120
130
140
150
160
170
180
190

Analyte Name: LM-flavones-109_1
Internal Standard: LM-flavones-IS02_1

**Data File**	flavones-STD-20230908.wiff	**Result Table**	DZLM2023082419-results-20230913-5500
**Acquisition Date**	9/8/2023 5:37:08 PM	**Algorithm Used**	MQ4
**Acquisition Method**	20230908-flavones-(mix130-T3)-15min.dam	**Instrument Name**	QTRAP 6500+ Low Mass
**Project**	N/A	**Processing Method**	20230412-flavones-(mix130-T3)-15min.qmethod

Regression Equation: y = 0.00355 x + -3.09113e-4 (r = 0.99965, r² = 0.99930) (weighting: 1 / x)

Expected Concentration	Number of Values	Mean Calculated Concentration (No data for Analyte Unit)	% Accuracy	Std. Deviation	%CV
0.01	0 of 1	N/A	N/A	N/A	N/A
0.02	0 of 1	N/A	N/A	N/A	N/A
0.05	0 of 1	N/A	N/A	N/A	N/A
0.13	0 of 1	N/A	N/A	N/A	N/A
0.33	0 of 1	N/A	N/A	N/A	N/A
0.82	0 of 1	N/A	N/A	N/A	N/A
2.05	1 of 1	2.010e0	98.1	N/A	N/A
5.12	1 of 1	5.360e0	104.7	N/A	N/A
12.80	1 of 1	1.180e1	92.2	N/A	N/A
32.00	1 of 1	3.386e1	105.8	N/A	N/A
80.00	1 of 1	7.969e1	99.6	N/A	N/A
200.00	1 of 1	1.992e2	99.6	N/A	N/A

Analyte Name: LM-flavones-109_1

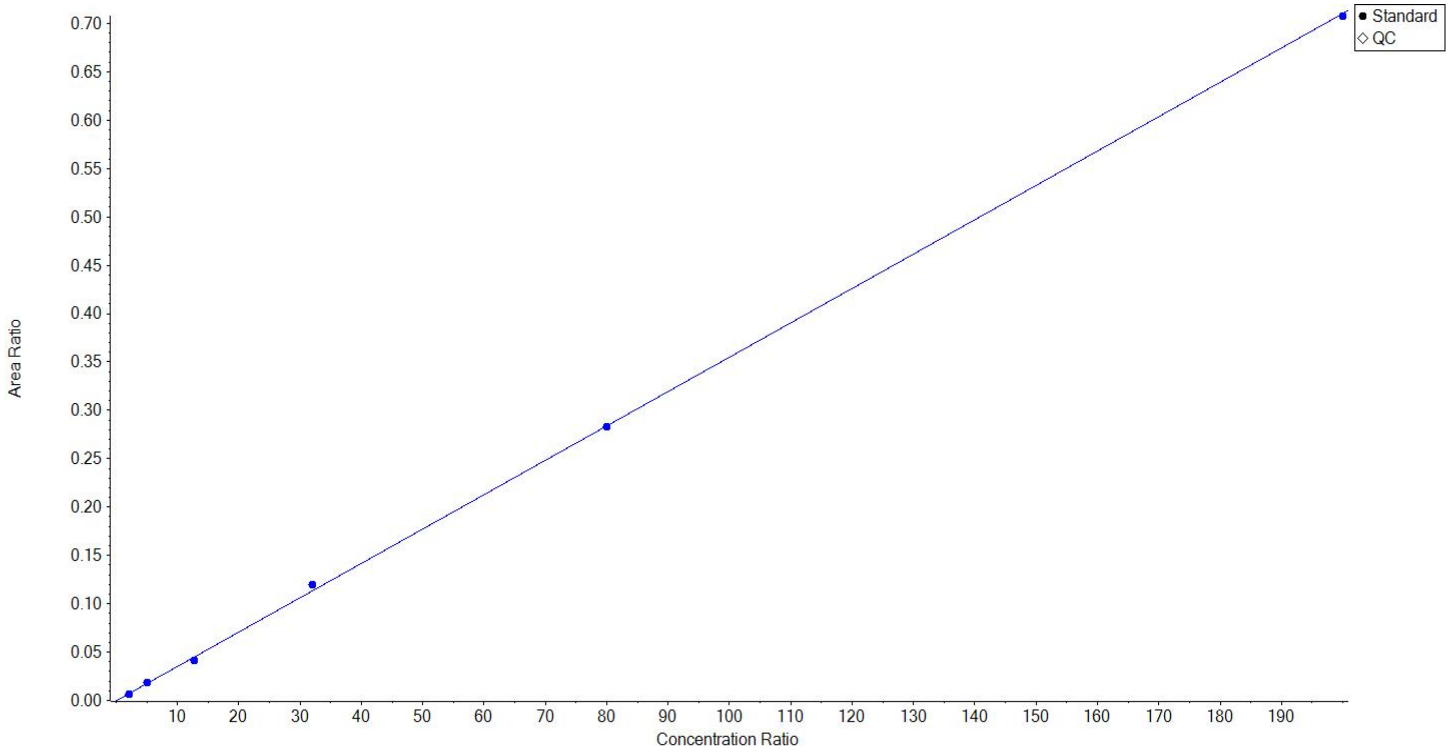
Standard
QC
Area Ratio
Concentration Ratio
0.70
0.65
0.60
0.55
0.50
0.45
0.40
0.35
0.30
0.25
0.20
0.15
0.10
0.05
0.00
10
20
30
40
50
60
70
80
90
100
110
120
130
140
150
160
170
180
190

Analyte Name: LM-flavones-107_1
Internal Standard: LM-flavones-IS02_1

**Data File**	flavones-STD-20230908.wiff	**Result Table**	DZLM2023082419-results-20230913-5500
**Acquisition Date**	9/8/2023 5:37:08 PM	**Algorithm Used**	MQ4
**Acquisition Method**	20230908-flavones-(mix130-T3)-15min.dam	**Instrument Name**	QTRAP 6500+ Low Mass
**Project**	N/A	**Processing Method**	20230412-flavones-(mix130-T3)-15min.qmethod

Regression Equation: y = 0.01092 x + -3.93249e-4 (r = 0.99991, r^2 = 0.99981) (weighting: 1 / x)

Expected Concentration	Number of Values	Mean Calculated Concentration (No data for Analyte Unit)	% Accuracy	Std. Deviation	%CV
0.01	0 of 1	N/A	N/A	N/A	N/A
0.02	0 of 1	N/A	N/A	N/A	N/A
0.05	0 of 1	N/A	N/A	N/A	N/A
0.13	0 of 1	N/A	N/A	N/A	N/A
0.33	0 of 1	N/A	N/A	N/A	N/A
0.82	0 of 1	N/A	N/A	N/A	N/A
2.05	1 of 1	2.012e0	98.1	N/A	N/A
5.12	1 of 1	5.072e0	99.1	N/A	N/A
12.80	1 of 1	1.275e1	99.6	N/A	N/A
32.00	1 of 1	3.324e1	103.9	N/A	N/A
80.00	1 of 1	7.980e1	99.8	N/A	N/A
200.00	1 of 1	1.991e2	99.6	N/A	N/A

Analyte Name: LM-flavones-107_1

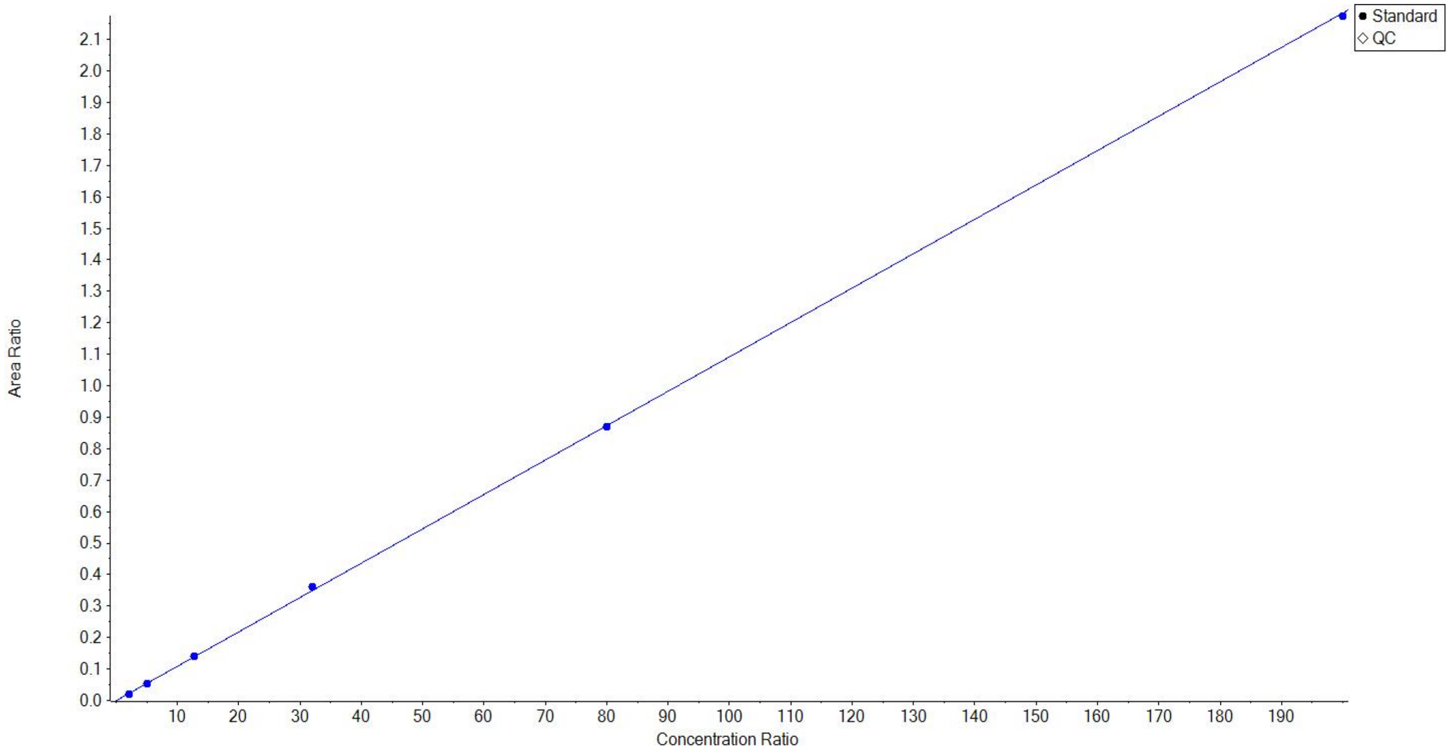
Standard
QC
Area Ratio
Concentration Ratio
0.0
0.1
0.2
0.3
0.4
0.5
0.6
0.7
0.8
0.9
1.0
1.1
1.2
1.3
1.4
1.5
1.6
1.7
1.8
1.9
2.0
2.1
10
20
30
40
50
60
70
80
90
100
110
120
130
140
150
160
170
180
190

Analyte Name: LM-flavones-110_1
Internal Standard: LM-flavones-IS02_1

**Data File**	flavones-STD-20230908.wiff	**Result Table**	DZLM2023082419-results-20230913-5500
**Acquisition Date**	9/8/2023 5:37:08 PM	**Algorithm Used**	MQ4
**Acquisition Method**	20230908-flavones-(mix130-T3)-15min.dam	**Instrument Name**	QTRAP 6500+ Low Mass
**Project**	N/A	**Processing Method**	20230412-flavones-(mix130-T3)-15min.qmethod

Regression Equation: y = 0.00315 x + -9.94657e-4 (r = 0.99977, r² = 0.99955) (weighting: 1 / x)

Expected Concentration	Number of Values	Mean Calculated Concentration (No data for Analyte Unit)	% Accuracy	Std. Deviation	%CV
0.01	0 of 1	N/A	N/A	N/A	N/A
0.02	0 of 1	N/A	N/A	N/A	N/A
0.05	0 of 1	N/A	N/A	N/A	N/A
0.13	0 of 1	N/A	N/A	N/A	N/A
0.33	0 of 1	N/A	N/A	N/A	N/A
0.82	0 of 1	N/A	N/A	N/A	N/A
2.05	1 of 1	2.020e0	98.5	N/A	N/A
5.12	1 of 1	5.232e0	102.2	N/A	N/A
12.80	1 of 1	1.215e1	94.9	N/A	N/A
32.00	1 of 1	3.369e1	105.3	N/A	N/A
80.00	1 of 1	7.956e1	99.5	N/A	N/A
200.00	1 of 1	1.993e2	99.7	N/A	N/A

Analyte Name: LM-flavones-110_1

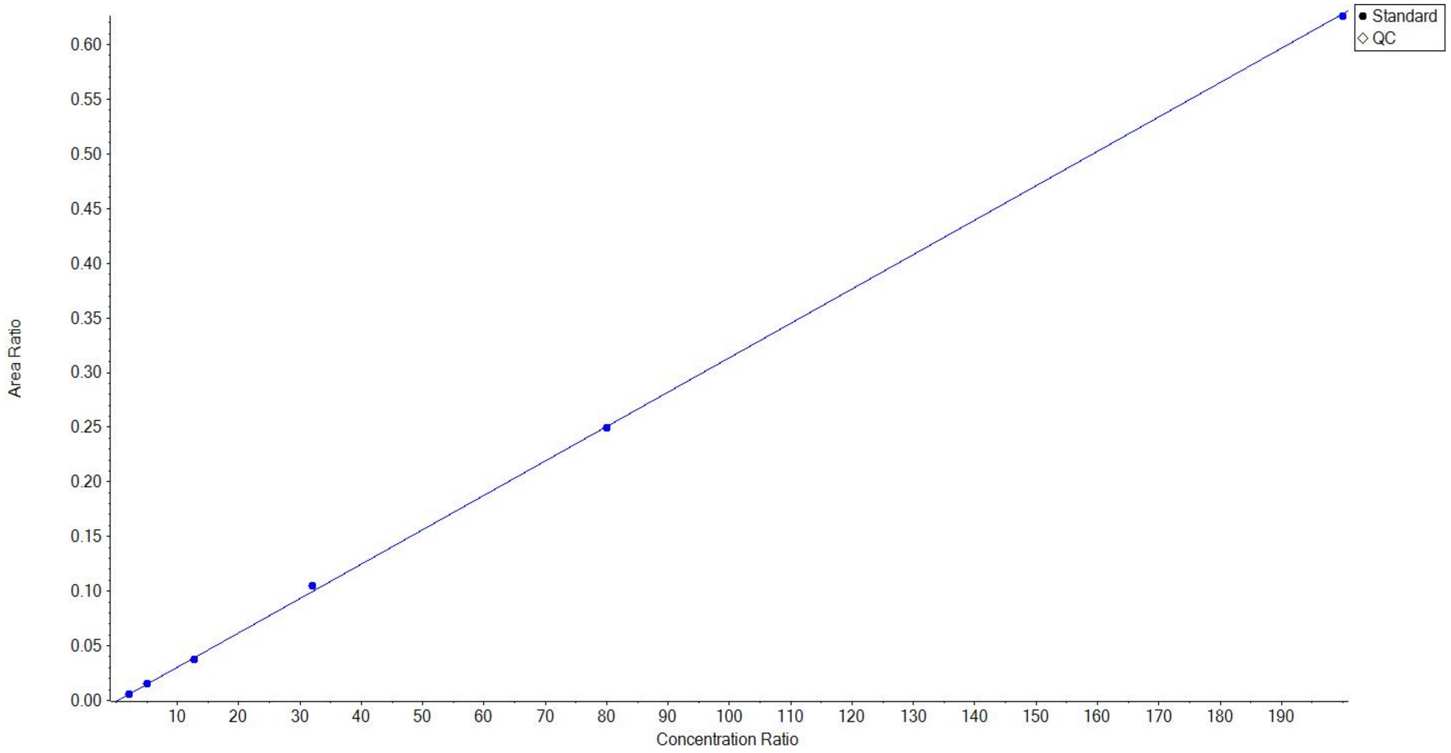
Standard
QC
Area Ratio
0.60
0.55
0.50
0.45
0.40
0.35
0.30
0.25
0.20
0.15
0.10
0.05
0.00
10
20
30
40
50
60
70
80
90
100
110
120
130
140
150
160
170
180
190
Concentration Ratio

Analyte Name: LM-flavones-111_1
Internal Standard: LM-flavones-IS02_1

**Data File**	flavones-STD-20230908.wiff	**Result Table**	DZLM2023082419-results-20230913-5500
**Acquisition Date**	9/8/2023 5:37:08 PM	**Algorithm Used**	MQ4
**Acquisition Method**	20230908-flavones-(mix130-T3)-15min.dam	**Instrument Name**	QTRAP 6500+ Low Mass
**Project**	N/A	**Processing Method**	20230412-flavones-(mix130-T3)-15min.qmethod

Regression Equation: y = 0.00297 x + -2.55911e-4 (r = 0.99994, r^2 = 0.99987) (weighting: 1 / x)

Expected Concentration	Number of Values	Mean Calculated Concentration (No data for Analyte Unit)	% Accuracy	Std. Deviation	%CV
0.01	0 of 1	N/A	N/A	N/A	N/A
0.02	0 of 1	N/A	N/A	N/A	N/A
0.05	0 of 1	N/A	N/A	N/A	N/A
0.13	0 of 1	N/A	N/A	N/A	N/A
0.33	0 of 1	N/A	N/A	N/A	N/A
0.82	0 of 1	N/A	N/A	N/A	N/A
2.05	1 of 1	2.088e0	101.9	N/A	N/A
5.12	1 of 1	5.186e0	101.3	N/A	N/A
12.80	1 of 1	1.239e1	96.8	N/A	N/A
32.00	1 of 1	3.227e1	100.9	N/A	N/A
80.00	1 of 1	7.897e1	98.7	N/A	N/A
200.00	1 of 1	2.011e2	100.5	N/A	N/A

Analyte Name: LM-flavones-111_1

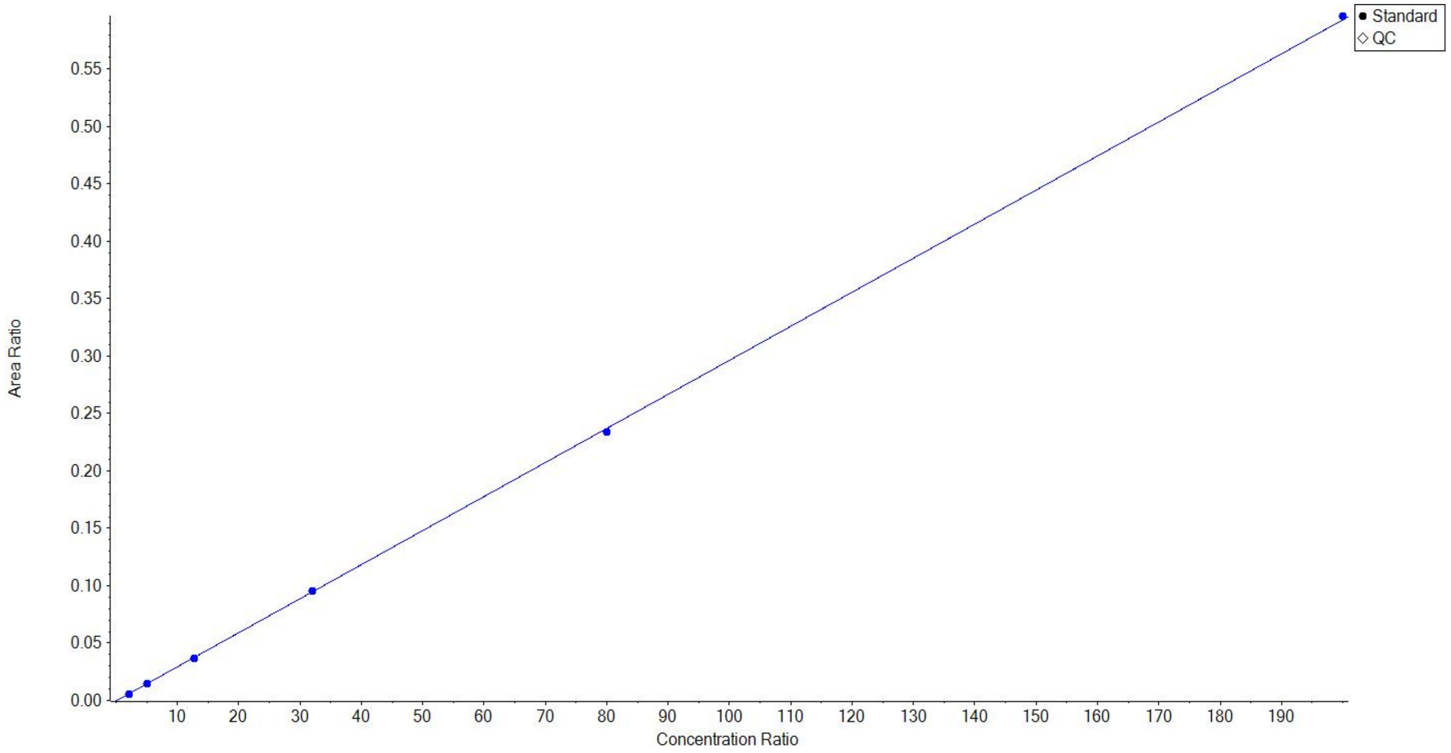
Standard
QC
Area Ratio
0.00
0.05
0.10
0.15
0.20
0.25
0.30
0.35
0.40
0.45
0.50
0.55
10
20
30
40
50
60
70
80
90
100
110
120
130
140
150
160
170
180
190
Concentration Ratio

Created with SCIEX OS 1.6.1
Printed:9/18/2023 5:27:01 PM

Analyte Name: LM-flavones-112_1
Internal Standard: LM-flavones-IS02_1

**Data File**	flavones-STD-20230908.wiff	**Result Table**	DZLM2023082419-results-20230913-5500
**Acquisition Date**	9/8/2023 5:37:08 PM	**Algorithm Used**	MQ4
**Acquisition Method**	20230908-flavones-(mix130-T3)-15min.dam	**Instrument Name**	QTRAP 6500+ Low Mass
**Project**	N/A	**Processing Method**	20230412-flavones-(mix130-T3)-15min.qmethod

Regression Equation: y = 0.00710 x + -0.00462 (r = 0.99945, r² = 0.99890) (weighting: 1 / x)

Expected Concentration	Number of Values	Mean Calculated Concentration (No data for Analyte Unit)	% Accuracy	Std. Deviation	%CV
0.01	0 of 1	N/A	N/A	N/A	N/A
0.02	0 of 1	N/A	N/A	N/A	N/A
0.05	0 of 1	N/A	N/A	N/A	N/A
0.13	0 of 1	N/A	N/A	N/A	N/A
0.33	0 of 1	N/A	N/A	N/A	N/A
0.82	0 of 1	N/A	N/A	N/A	N/A
2.05	1 of 1	2.325e0	113.4	N/A	N/A
5.12	1 of 1	4.913e0	96.0	N/A	N/A
12.80	1 of 1	1.201e1	93.8	N/A	N/A
32.00	1 of 1	3.154e1	98.6	N/A	N/A
80.00	1 of 1	7.686e1	96.1	N/A	N/A
200.00	1 of 1	2.043e2	102.2	N/A	N/A

Analyte Name: LM-flavones-112_1

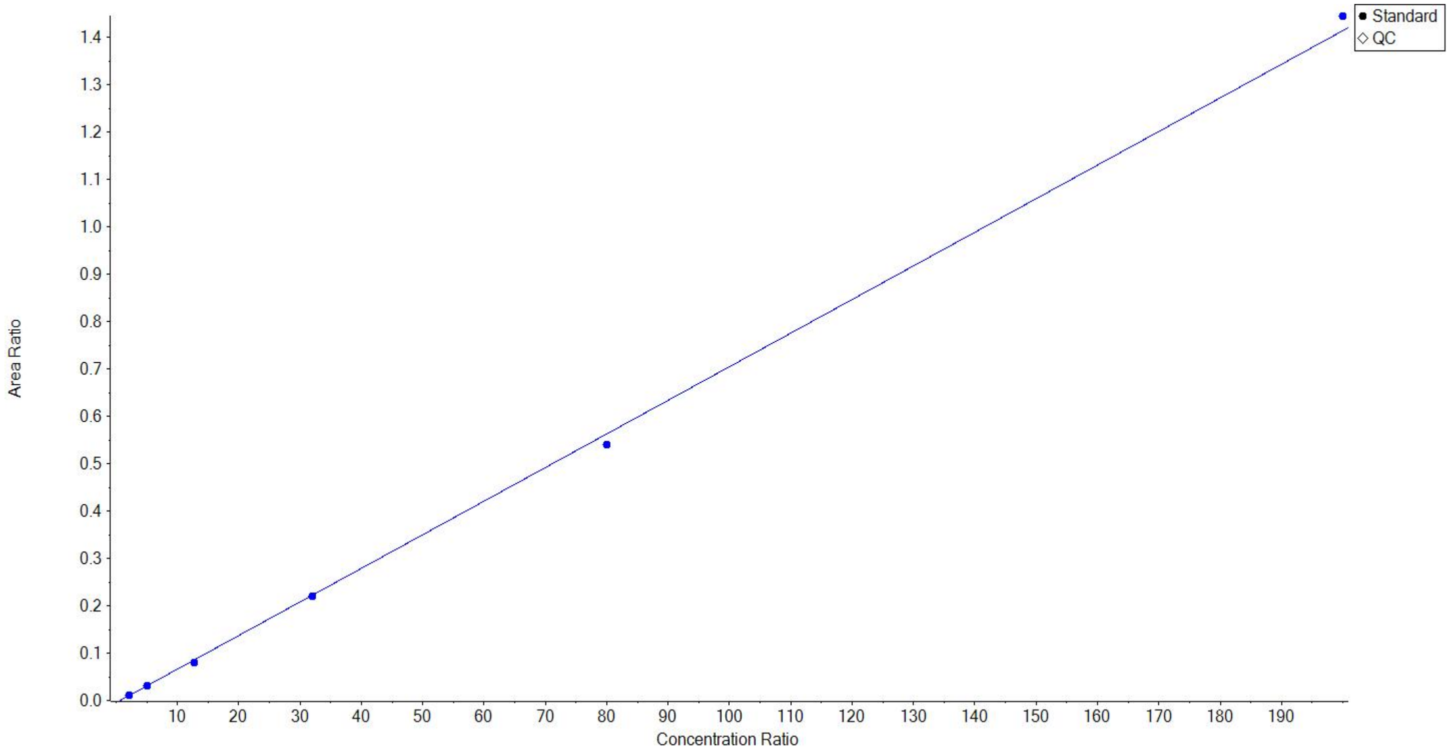
Standard
QC
Area Ratio
Concentration Ratio
1.4
1.3
1.2
1.1
1.0
0.9
0.8
0.7
0.6
0.5
0.4
0.3
0.2
0.1
0.0
10
20
30
40
50
60
70
80
90
100
110
120
130
140
150
160
170
180
190

Analyte Name: LM-flavones-113_1
Internal Standard: LM-flavones-IS02_1

**Data File**	flavones-STD-20230908.wiff	**Result Table**	DZLM2023082419-results-20230913-5500
**Acquisition Date**	9/8/2023 5:37:08 PM	**Algorithm Used**	MQ4
**Acquisition Method**	20230908-flavones-(mix130-T3)-15min.dam	**Instrument Name**	QTRAP 6500+ Low Mass
**Project**	N/A	**Processing Method**	20230412-flavones-(mix130-T3)-15min.qmethod

Regression Equation: $y = 0.00152 x + 9.56836e-5$ ($r = 0.99930$, $r^2 = 0.99860$) (weighting: 1 / x)

Expected Concentration	Number of Values	Mean Calculated Concentration (No data for Analyte Unit)	% Accuracy	Std. Deviation	%CV
0.01	0 of 1	N/A	N/A	N/A	N/A
0.02	0 of 1	N/A	N/A	N/A	N/A
0.05	0 of 1	N/A	N/A	N/A	N/A
0.13	0 of 1	N/A	N/A	N/A	N/A
0.33	0 of 1	N/A	N/A	N/A	N/A
0.82	0 of 1	N/A	N/A	N/A	N/A
2.05	1 of 1	1.981e0	96.7	N/A	N/A
5.12	1 of 1	5.558e0	108.6	N/A	N/A
12.80	1 of 1	1.161e1	90.7	N/A	N/A
32.00	1 of 1	3.419e1	106.8	N/A	N/A
80.00	1 of 1	7.729e1	96.6	N/A	N/A
200.00	1 of 1	2.014e2	100.7	N/A	N/A

Analyte Name: LM-flavones-113_1

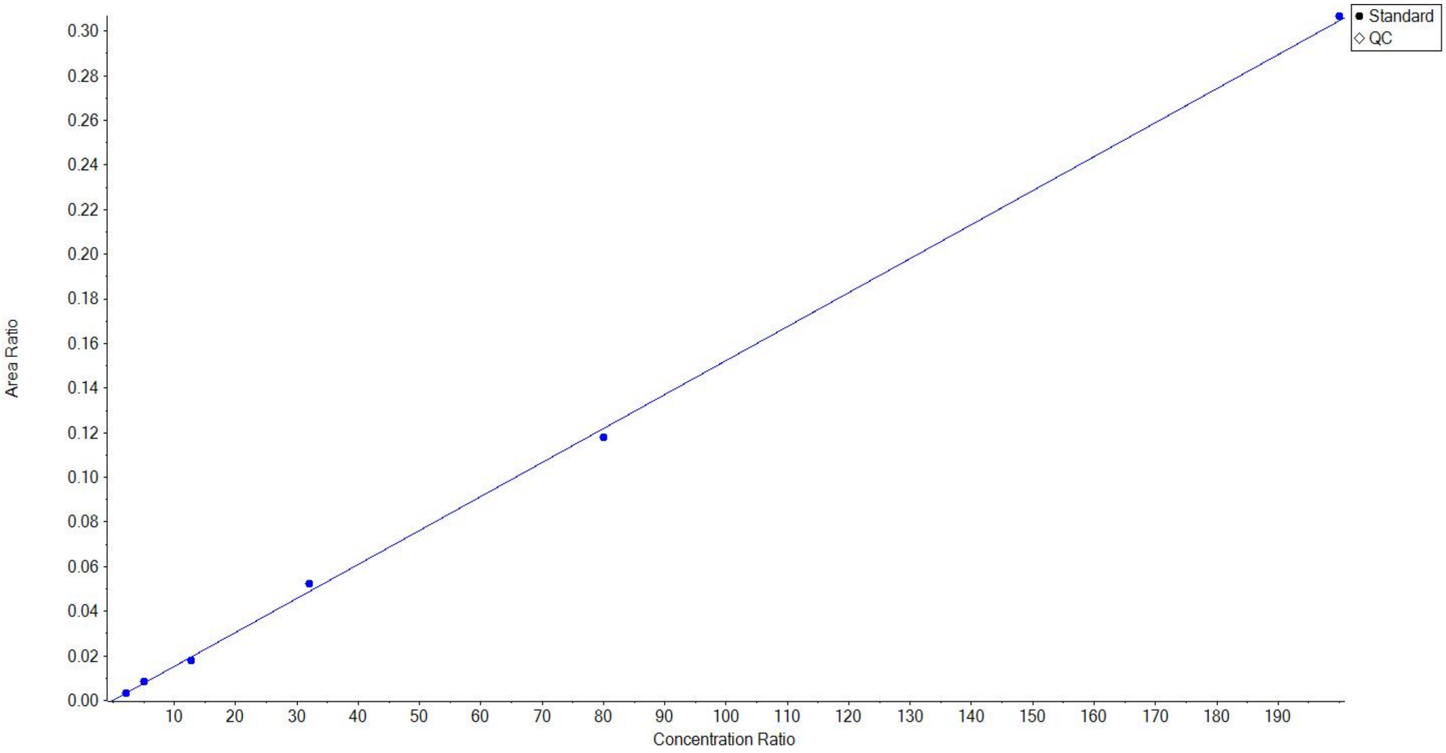
Standard
QC
Area Ratio
0.30
0.28
0.26
0.24
0.22
0.20
0.18
0.16
0.14
0.12
0.10
0.08
0.06
0.04
0.02
0.00
10
20
30
40
50
60
70
80
90
100
110
120
130
140
150
160
170
180
190
Concentration Ratio

Analyte Name: LM-flavones-114_1
Internal Standard: LM-flavones-IS02_1

**Data File**	flavones-STD-20230908.wiff	**Result Table**	DZLM2023082419-results-20230913-5500
**Acquisition Date**	9/8/2023 5:37:08 PM	**Algorithm Used**	MQ4
**Acquisition Method**	20230908-flavones-(mix130-T3)-15min.dam	**Instrument Name**	QTRAP 6500+ Low Mass
**Project**	N/A	**Processing Method**	20230412-flavones-(mix130-T3)-15min.qmethod

Regression Equation: y = 0.01223 x + -0.00253 (r = 0.99993, r² = 0.99986) (weighting: 1 / x)

Expected Concentration	Number of Values	Mean Calculated Concentration (No data for Analyte Unit)	% Accuracy	Std. Deviation	%CV
0.01	0 of 1	N/A	N/A	N/A	N/A
0.02	0 of 1	N/A	N/A	N/A	N/A
0.05	0 of 1	N/A	N/A	N/A	N/A
0.13	0 of 1	N/A	N/A	N/A	N/A
0.33	0 of 1	N/A	N/A	N/A	N/A
0.82	0 of 1	N/A	N/A	N/A	N/A
2.05	1 of 1	2.156e0	105.2	N/A	N/A
5.12	1 of 1	5.040e0	98.4	N/A	N/A
12.80	1 of 1	1.251e1	97.8	N/A	N/A
32.00	1 of 1	3.170e1	99.1	N/A	N/A
80.00	1 of 1	7.901e1	98.8	N/A	N/A
200.00	1 of 1	2.015e2	100.8	N/A	N/A

Analyte Name: LM-flavones-114_1

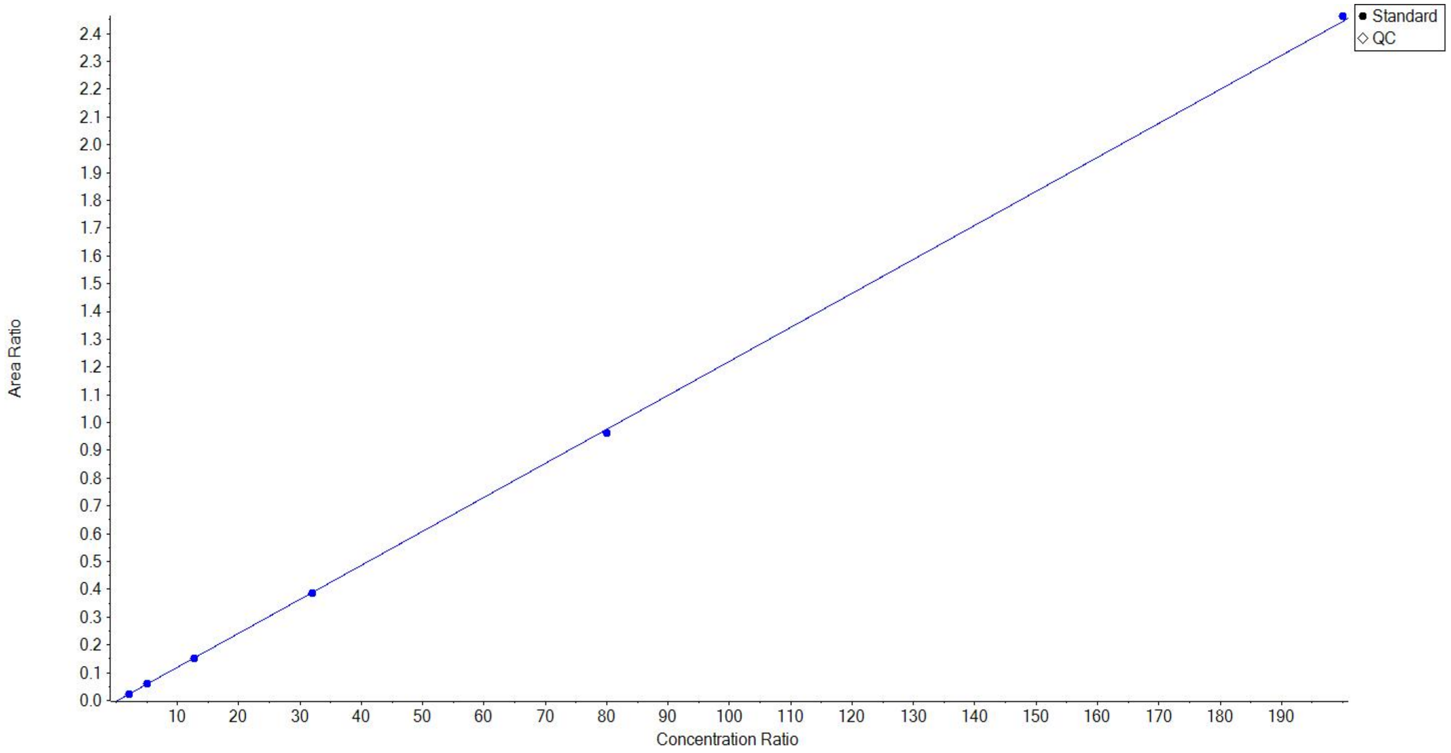
Standard
QC
Area Ratio
Concentration Ratio
2.4
2.3
2.2
2.1
2.0
1.9
1.8
1.7
1.6
1.5
1.4
1.3
1.2
1.1
1.0
0.9
0.8
0.7
0.6
0.5
0.4
0.3
0.2
0.1
0.0
10
20
30
40
50
60
70
80
90
100
110
120
130
140
150
160
170
180
190

Analyte Name: LM-flavones-115_1
Internal Standard: LM-flavones-IS02_1

**Data File**	flavones-STD-20230908.wiff	**Result Table**	DZLM2023082419-results-20230913-5500
**Acquisition Date**	9/8/2023 5:37:08 PM	**Algorithm Used**	MQ4
**Acquisition Method**	20230908-flavones-(mix130-T3)-15min.dam	**Instrument Name**	QTRAP 6500+ Low Mass
**Project**	N/A	**Processing Method**	20230412-flavones-(mix130-T3)-15min.qmethod

Regression Equation: y = 0.00126 x + -8.42298e-5 (r = 0.99972, r² = 0.99945) (weighting: 1 / x)

Expected Concentration	Number of Values	Mean Calculated Concentration (No data for Analyte Unit)	% Accuracy	Std. Deviation	%CV
0.01	0 of 1	N/A	N/A	N/A	N/A
0.02	0 of 1	N/A	N/A	N/A	N/A
0.05	0 of 1	N/A	N/A	N/A	N/A
0.13	0 of 1	N/A	N/A	N/A	N/A
0.33	0 of 1	N/A	N/A	N/A	N/A
0.82	0 of 1	N/A	N/A	N/A	N/A
2.05	1 of 1	2.012e0	98.2	N/A	N/A
5.12	1 of 1	5.091e0	99.4	N/A	N/A
12.80	1 of 1	1.237e1	96.6	N/A	N/A
32.00	1 of 1	3.379e1	105.6	N/A	N/A
80.00	1 of 1	8.109e1	101.4	N/A	N/A
200.00	1 of 1	1.976e2	98.8	N/A	N/A

Analyte Name: LM-flavones-115_1

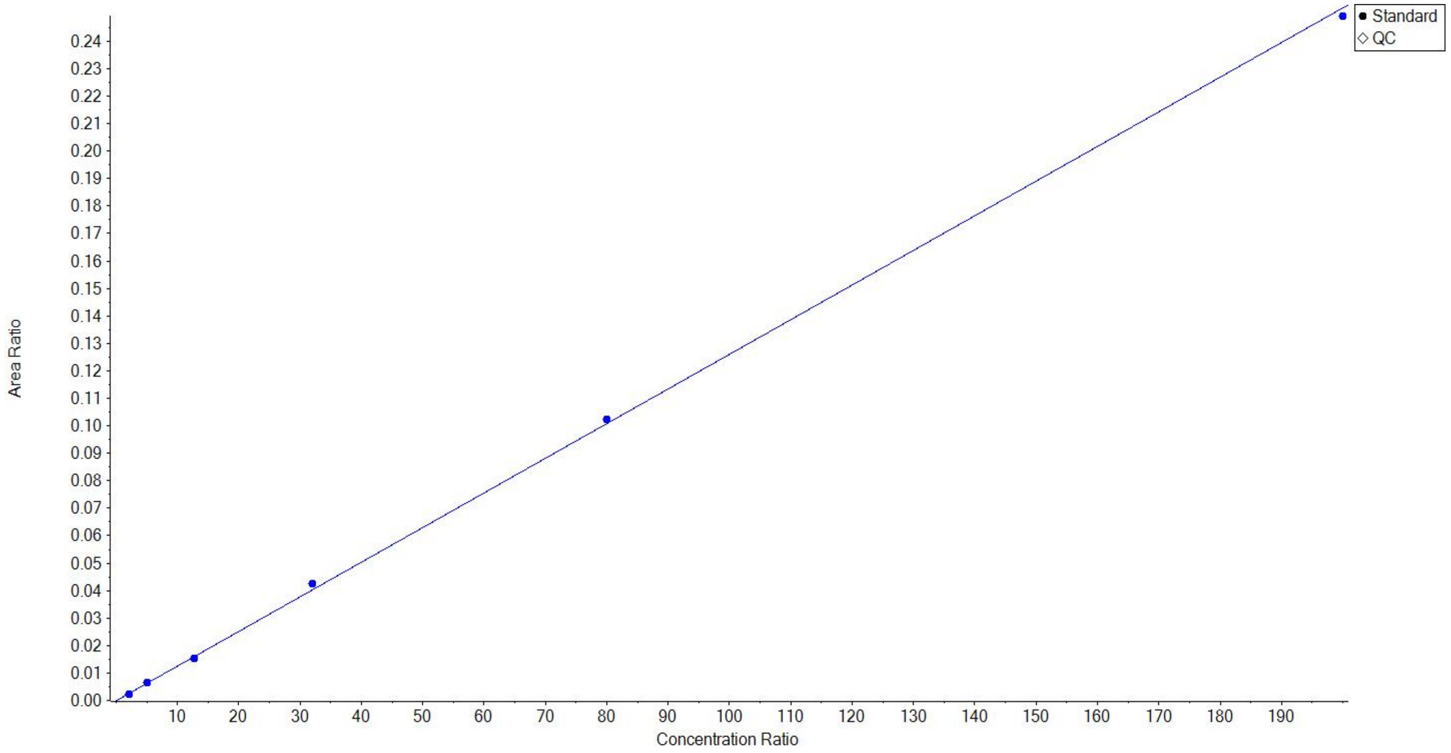
Standard
QC
Area Ratio
0.00
0.01
0.02
0.03
0.04
0.05
0.06
0.07
0.08
0.09
0.10
0.11
0.12
0.13
0.14
0.15
0.16
0.17
0.18
0.19
0.20
0.21
0.22
0.23
0.24
10
20
30
40
50
60
70
80
90
100
110
120
130
140
150
160
170
180
190
Concentration Ratio

Analyte Name: LM-flavones-116_1
Internal Standard: LM-flavones-IS02_1

**Data File**	flavones-STD-20230908.wiff	**Result Table**	DZLM2023082419-results-20230913-5500
**Acquisition Date**	9/8/2023 5:37:08 PM	**Algorithm Used**	MQ4
**Acquisition Method**	20230908-flavones-(mix130-T3)-15min.dam	**Instrument Name**	QTRAP 6500+ Low Mass
**Project**	N/A	**Processing Method**	20230412-flavones-(mix130-T3)-15min.qmethod

Regression Equation: y = 0.01279 x + 0.00117 (r = 0.99994, r² = 0.99988) (weighting: 1 / x)

Expected Concentration	Number of Values	Mean Calculated Concentration (No data for Analyte Unit)	% Accuracy	Std. Deviation	%CV
0.01	0 of 1	N/A	N/A	N/A	N/A
0.02	0 of 1	N/A	N/A	N/A	N/A
0.05	0 of 1	N/A	N/A	N/A	N/A
0.13	0 of 1	N/A	N/A	N/A	N/A
0.33	0 of 1	N/A	N/A	N/A	N/A
0.82	0 of 1	N/A	N/A	N/A	N/A
2.05	1 of 1	2.080e0	101.5	N/A	N/A
5.12	1 of 1	5.095e0	99.5	N/A	N/A
12.80	1 of 1	1.279e1	99.9	N/A	N/A
32.00	1 of 1	3.208e1	100.2	N/A	N/A
80.00	1 of 1	7.857e1	98.2	N/A	N/A
200.00	1 of 1	2.014e2	100.7	N/A	N/A

Analyte Name: LM-flavones-116_1

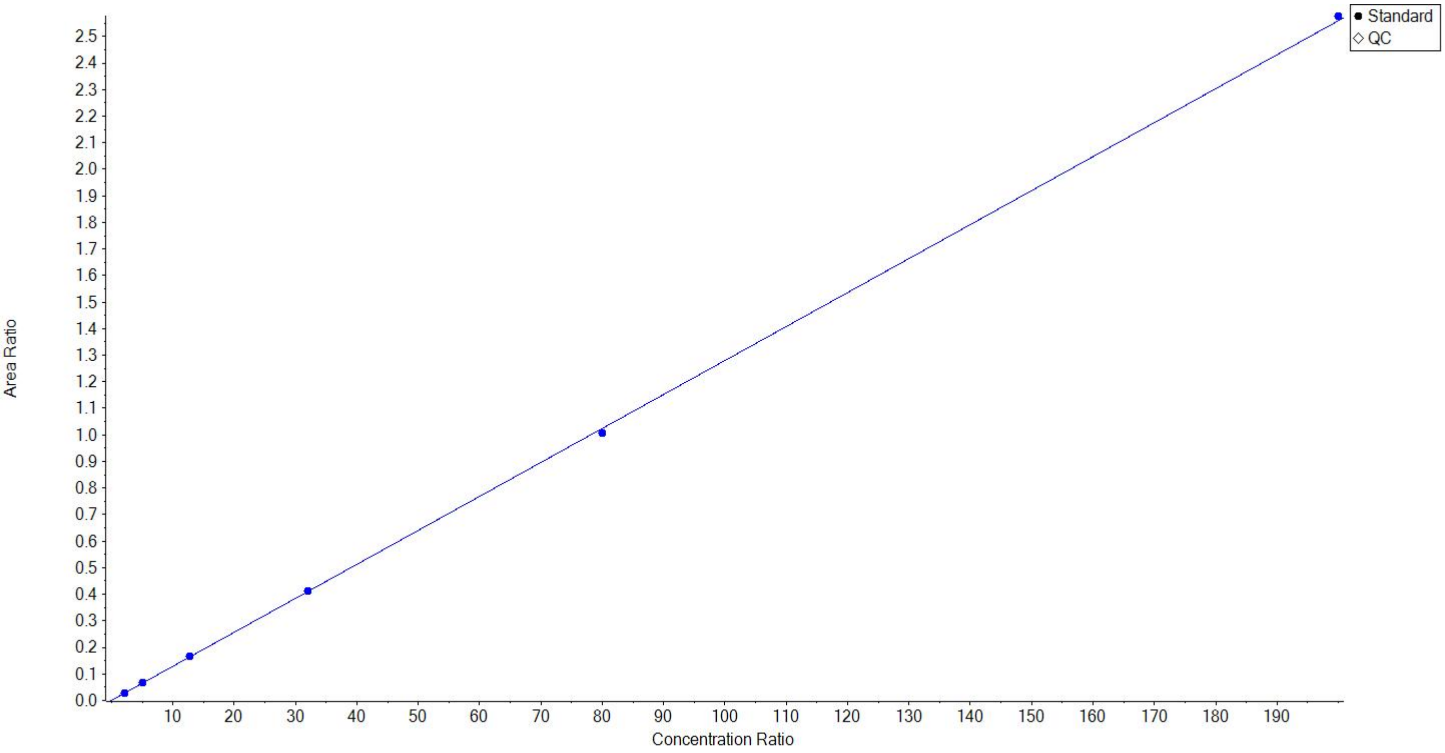
Standard
QC
Area Ratio
Concentration Ratio
0.0
0.1
0.2
0.3
0.4
0.5
0.6
0.7
0.8
0.9
1.0
1.1
1.2
1.3
1.4
1.5
1.6
1.7
1.8
1.9
2.0
2.1
2.2
2.3
2.4
2.5
10
20
30
40
50
60
70
80
90
100
110
120
130
140
150
160
170
180
190

Analyte Name: LM-flavones-118_1
Internal Standard: LM-flavones-IS02_1

**Data File**	flavones-STD-20230908.wiff	**Result Table**	DZLM2023082419-results-20230913-5500
**Acquisition Date**	9/8/2023 5:37:08 PM	**Algorithm Used**	MQ4
**Acquisition Method**	20230908-flavones-(mix130-T3)-15min.dam	**Instrument Name**	QTRAP 6500+ Low Mass
**Project**	N/A	**Processing Method**	20230412-flavones-(mix130-T3)-15min.qmethod

Regression Equation: y = 0.00598 x + -0.00150 (r = 0.99966, r^2 = 0.99931) (weighting: 1 / x)

Expected Concentration	Number of Values	Mean Calculated Concentration (No data for Analyte Unit)	% Accuracy	Std. Deviation	%CV
0.01	0 of 1	N/A	N/A	N/A	N/A
0.02	0 of 1	N/A	N/A	N/A	N/A
0.05	0 of 1	N/A	N/A	N/A	N/A
0.13	0 of 1	N/A	N/A	N/A	N/A
0.33	0 of 1	N/A	N/A	N/A	N/A
0.82	0 of 1	N/A	N/A	N/A	N/A
2.05	1 of 1	2.243e0	109.4	N/A	N/A
5.12	1 of 1	4.976e0	97.2	N/A	N/A
12.80	1 of 1	1.248e1	97.5	N/A	N/A
32.00	1 of 1	3.109e1	97.2	N/A	N/A
80.00	1 of 1	7.754e1	96.9	N/A	N/A
200.00	1 of 1	2.036e2	101.8	N/A	N/A

Analyte Name: LM-flavones-118_1

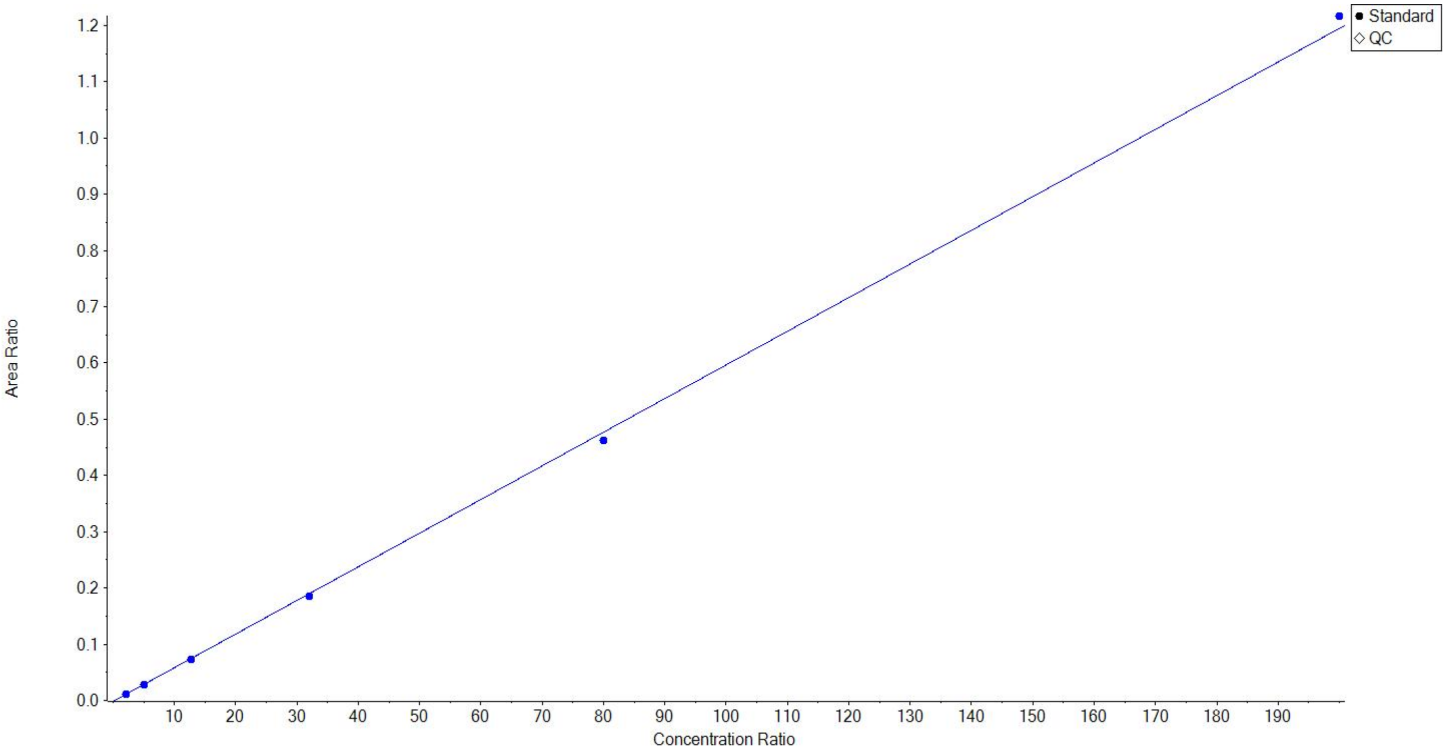
Standard
QC
Area Ratio
Concentration Ratio
1.2
1.1
1.0
0.9
0.8
0.7
0.6
0.5
0.4
0.3
0.2
0.1
0.0
10
20
30
40
50
60
70
80
90
100
110
120
130
140
150
160
170
180
190

Analyte Name: LM-flavones-119_1
Internal Standard: LM-flavones-IS02_1

**Data File**	flavones-STD-20230908.wiff	**Result Table**	DZLM2023082419-results-20230913-5500
**Acquisition Date**	9/8/2023 5:37:08 PM	**Algorithm Used**	MQ4
**Acquisition Method**	20230908-flavones-(mix130-T3)-15min.dam	**Instrument Name**	QTRAP 6500+ Low Mass
**Project**	N/A	**Processing Method**	20230412-flavones-(mix130-T3)-15min.qmethod

Regression Equation: y = 5.63163e-4 x + -6.33815e-4 (r = 0.99928, r² = 0.99855) (weighting: 1 / x)

Expected Concentration	Number of Values	Mean Calculated Concentration (No data for Analyte Unit)	% Accuracy	Std. Deviation	%CV
0.01	0 of 1	N/A	N/A	N/A	N/A
0.02	0 of 1	N/A	N/A	N/A	N/A
0.05	0 of 1	N/A	N/A	N/A	N/A
0.13	0 of 1	N/A	N/A	N/A	N/A
0.33	0 of 1	N/A	N/A	N/A	N/A
0.82	0 of 1	N/A	N/A	N/A	N/A
2.05	1 of 1	2.211e0	107.9	N/A	N/A
5.12	1 of 1	5.235e0	102.2	N/A	N/A
12.80	1 of 1	1.267e1	99.0	N/A	N/A
32.00	1 of 1	2.882e1	90.1	N/A	N/A
80.00	1 of 1	7.916e1	99.0	N/A	N/A
200.00	1 of 1	2.039e2	101.9	N/A	N/A

Analyte Name: LM-flavones-119_1

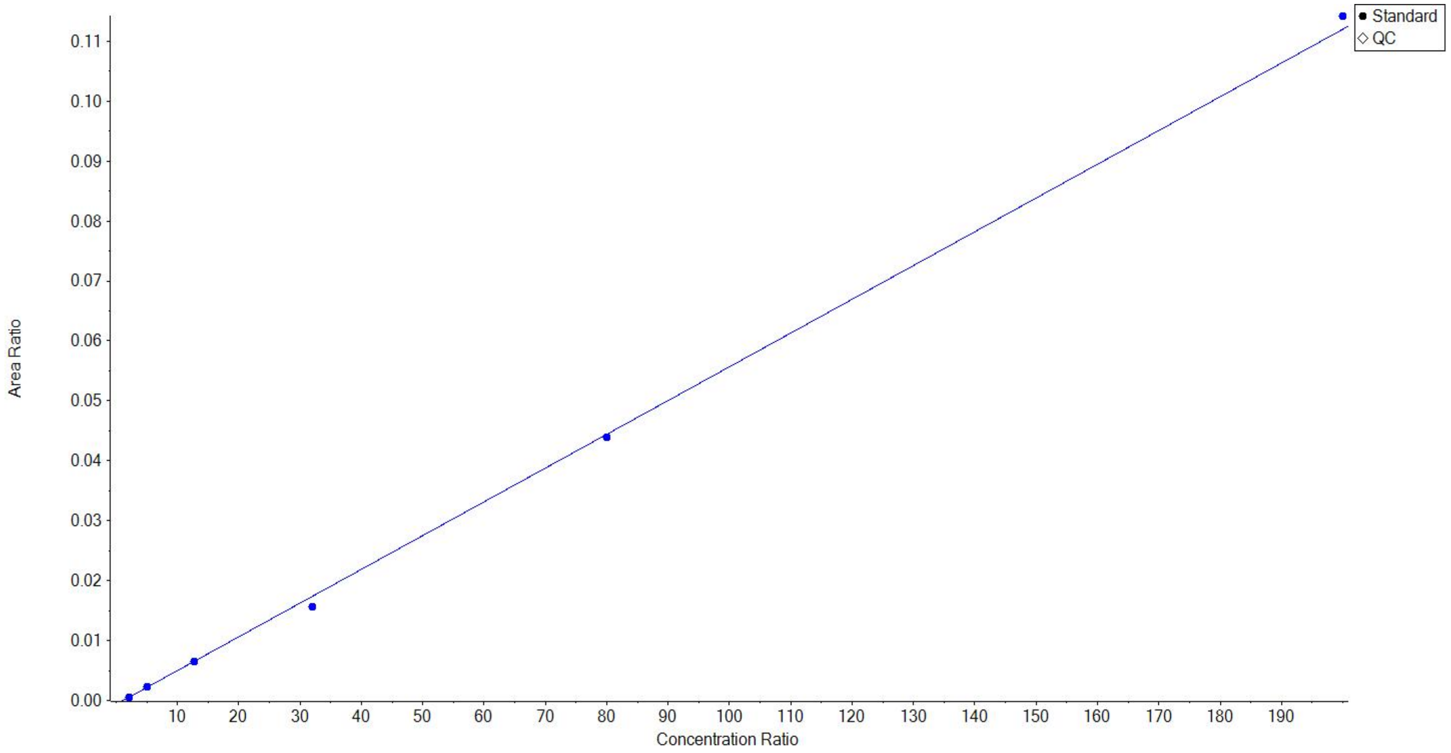
Standard
QC
Area Ratio
0.00
0.01
0.02
0.03
0.04
0.05
0.06
0.07
0.08
0.09
0.10
0.11
Concentration Ratio
10
20
30
40
50
60
70
80
90
100
110
120
130
140
150
160
170
180
190

Analyte Name: LM-flavones-122_1
Internal Standard: LM-flavones-IS02_1

**Data File**	flavones-STD-20230908.wiff	**Result Table**	DZLM2023082419-results-20230913-5500
**Acquisition Date**	9/8/2023 5:37:08 PM	**Algorithm Used**	MQ4
**Acquisition Method**	20230908-flavones-(mix130-T3)-15min.dam	**Instrument Name**	QTRAP 6500+ Low Mass
**Project**	N/A	**Processing Method**	20230412-flavones-(mix130-T3)-15min.qmethod

Regression Equation: y = 0.00238 x + 0.00142 (r = 0.99936, r² = 0.99872) (weighting: 1 / x)

Expected Concentration	Number of Values	Mean Calculated Concentration (No data for Analyte Unit)	% Accuracy	Std. Deviation	%CV
0.01	0 of 1	N/A	N/A	N/A	N/A
0.02	0 of 1	N/A	N/A	N/A	N/A
0.05	0 of 1	N/A	N/A	N/A	N/A
0.13	0 of 1	N/A	N/A	N/A	N/A
0.33	0 of 1	N/A	N/A	N/A	N/A
0.82	0 of 1	N/A	N/A	N/A	N/A
2.05	1 of 1	2.040e0	99.5	N/A	N/A
5.12	1 of 1	4.661e0	91.0	N/A	N/A
12.80	1 of 1	1.299e1	101.5	N/A	N/A
32.00	1 of 1	3.439e1	107.5	N/A	N/A
80.00	1 of 1	8.215e1	102.7	N/A	N/A
200.00	1 of 1	1.957e2	97.9	N/A	N/A

Analyte Name: LM-flavones-122_1

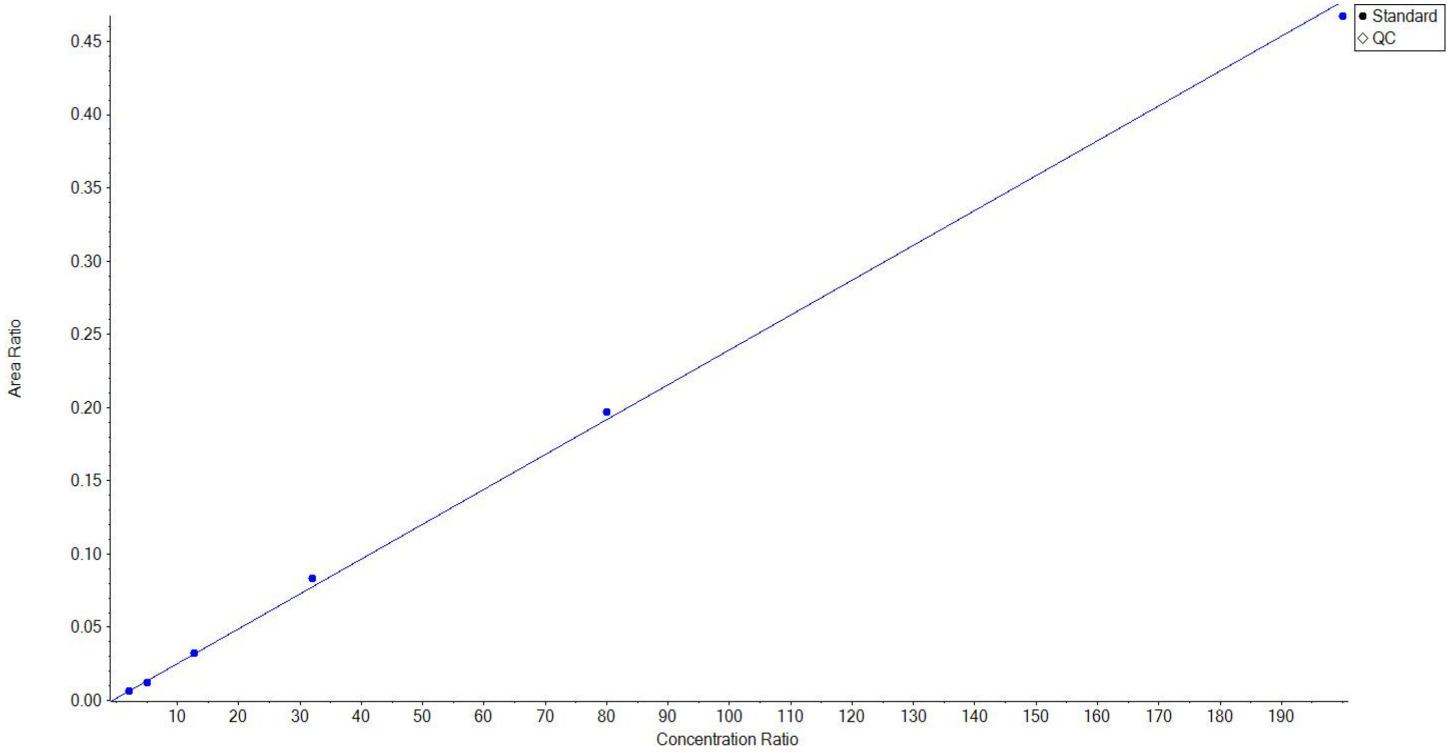
Standard
QC
Area Ratio
0.45
0.40
0.35
0.30
0.25
0.20
0.15
0.10
0.05
0.00
10
20
30
40
50
60
70
80
90
100
110
120
130
140
150
160
170
180
190
Concentration Ratio

Analyte Name: LM-flavones-123_1
Internal Standard: LM-flavones-IS02_1

**Data File**	flavones-STD-20230908.wiff	**Result Table**	DZLM2023082419-results-20230913-5500
**Acquisition Date**	9/8/2023 5:37:08 PM	**Algorithm Used**	MQ4
**Acquisition Method**	20230908-flavones-(mix130-T3)-15min.dam	**Instrument Name**	QTRAP 6500+ Low Mass
**Project**	N/A	**Processing Method**	20230412-flavones-(mix130-T3)-15min.qmethod

Regression Equation: y = 0.00344 x + 5.15819e-4 (r = 0.99994, r² = 0.99987) (weighting: 1 / x)

Expected Concentration	Number of Values	Mean Calculated Concentration (No data for Analyte Unit)	% Accuracy	Std. Deviation	%CV
0.01	0 of 1	N/A	N/A	N/A	N/A
0.02	0 of 1	N/A	N/A	N/A	N/A
0.05	0 of 1	N/A	N/A	N/A	N/A
0.13	0 of 1	N/A	N/A	N/A	N/A
0.33	0 of 1	N/A	N/A	N/A	N/A
0.82	0 of 1	N/A	N/A	N/A	N/A
2.05	1 of 1	2.126e0	103.7	N/A	N/A
5.12	1 of 1	4.875e0	95.2	N/A	N/A
12.80	1 of 1	1.271e1	99.3	N/A	N/A
32.00	1 of 1	3.277e1	102.4	N/A	N/A
80.00	1 of 1	7.949e1	99.4	N/A	N/A
200.00	1 of 1	2.000e2	100.0	N/A	N/A

Analyte Name: LM-flavones-123_1

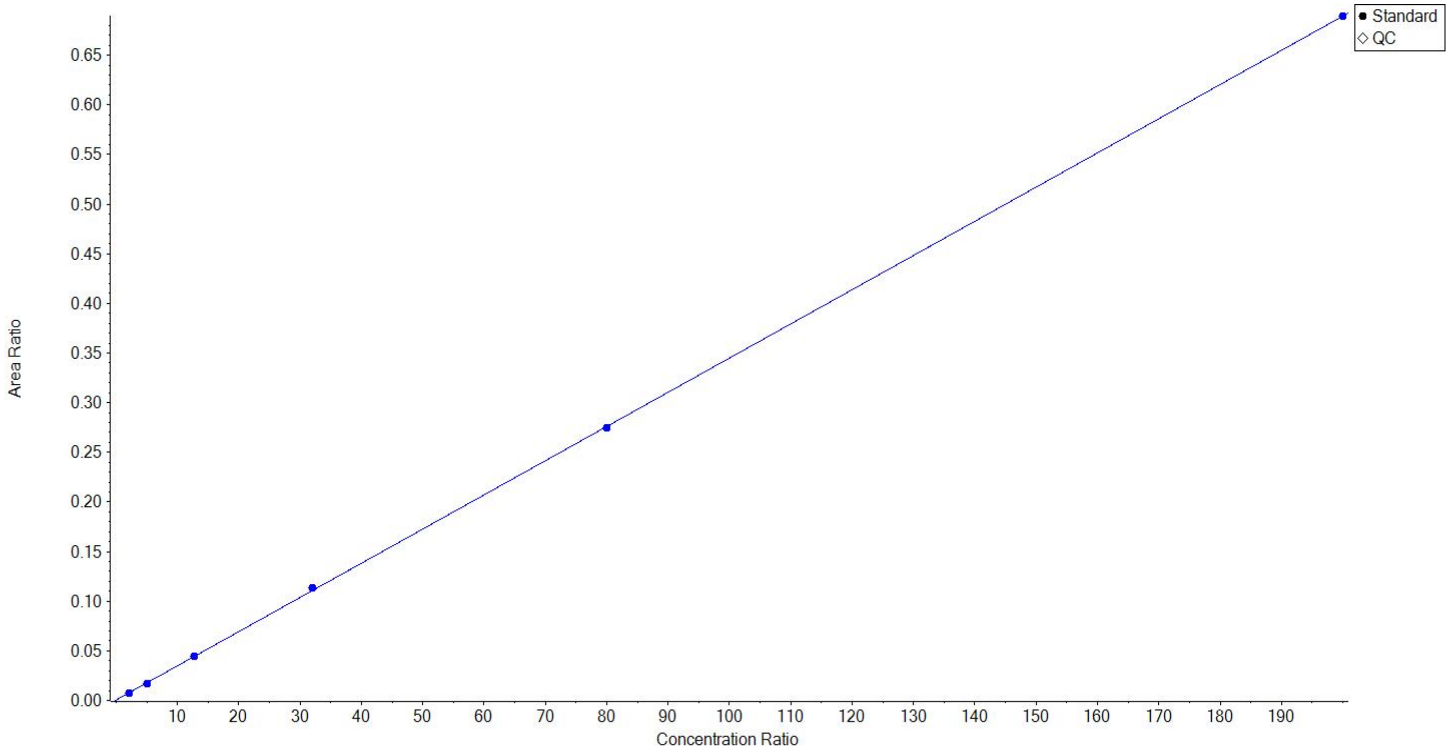
Standard
QC
Area Ratio
0.00
0.05
0.10
0.15
0.20
0.25
0.30
0.35
0.40
0.45
0.50
0.55
0.60
0.65
10
20
30
40
50
60
70
80
90
100
110
120
130
140
150
160
170
180
190
Concentration Ratio

Analyte Name: LM-flavones-125_1
Internal Standard: LM-flavones-IS02_1

**Data File**	flavones-STD-20230908.wiff	**Result Table**	DZLM2023082419-results-20230913-5500
**Acquisition Date**	9/8/2023 5:37:08 PM	**Algorithm Used**	MQ4
**Acquisition Method**	20230908-flavones-(mix130-T3)-15min.dam	**Instrument Name**	QTRAP 6500+ Low Mass
**Project**	N/A	**Processing Method**	20230412-flavones-(mix130-T3)-15min.qmethod

Regression Equation: y = 0.00310 x + -2.62863e-4 (r = 0.99996, r² = 0.99993) (weighting: 1 / x)

Expected Concentration	Number of Values	Mean Calculated Concentration (No data for Analyte Unit)	% Accuracy	Std. Deviation	%CV
0.01	0 of 1	N/A	N/A	N/A	N/A
0.02	0 of 1	N/A	N/A	N/A	N/A
0.05	0 of 1	N/A	N/A	N/A	N/A
0.13	0 of 1	N/A	N/A	N/A	N/A
0.33	0 of 1	N/A	N/A	N/A	N/A
0.82	0 of 1	N/A	N/A	N/A	N/A
2.05	1 of 1	2.029e0	99.0	N/A	N/A
5.12	1 of 1	5.274e0	103.0	N/A	N/A
12.80	1 of 1	1.251e1	97.7	N/A	N/A
32.00	1 of 1	3.227e1	100.8	N/A	N/A
80.00	1 of 1	7.935e1	99.2	N/A	N/A
200.00	1 of 1	2.005e2	100.3	N/A	N/A

Analyte Name: LM-flavones-125_1

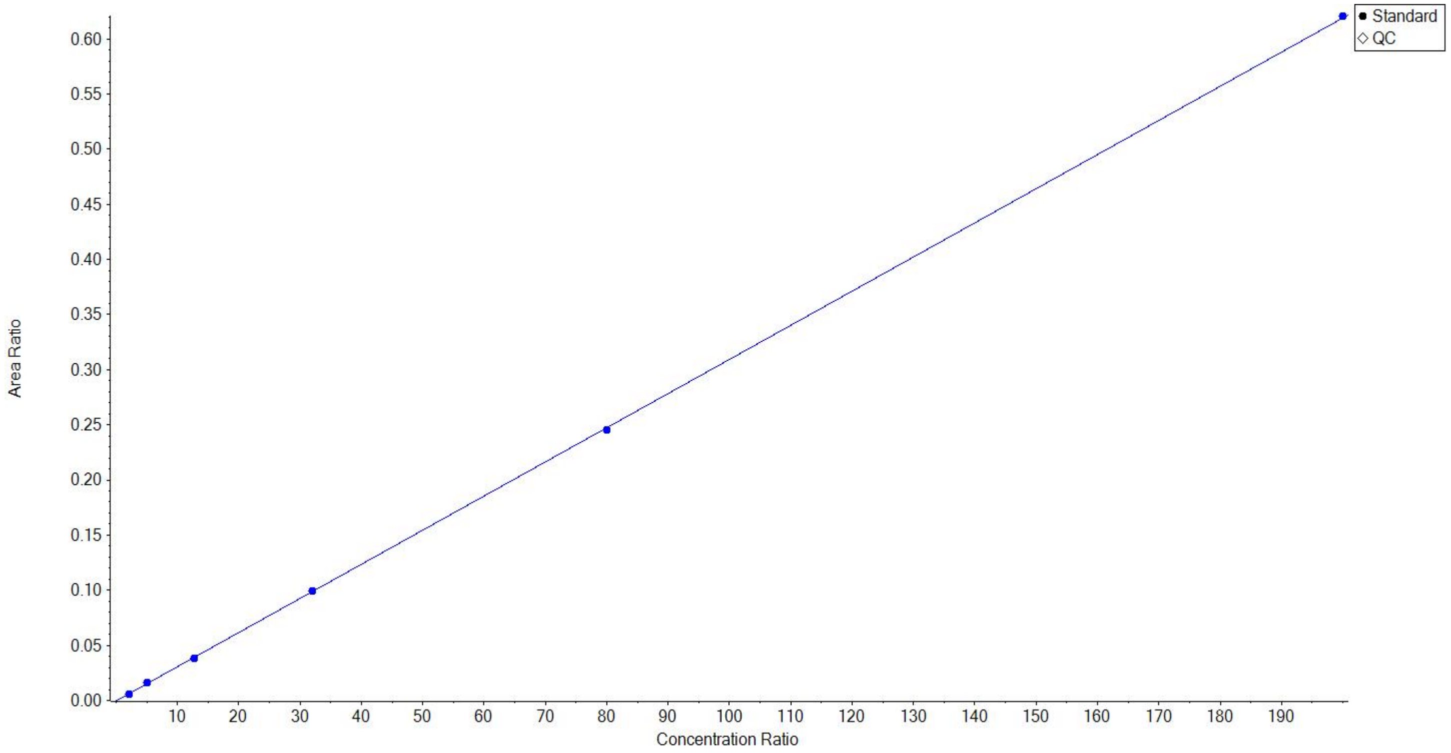
Standard
QC
Area Ratio
0.60
0.55
0.50
0.45
0.40
0.35
0.30
0.25
0.20
0.15
0.10
0.05
0.00
10
20
30
40
50
60
70
80
90
100
110
120
130
140
150
160
170
180
190
Concentration Ratio

Analyte Name: LM-flavones-126_1
Internal Standard: LM-flavones-IS02_1

**Data File**	flavones-STD-20230908.wiff	**Result Table**	DZLM2023082419-results-20230913-5500
**Acquisition Date**	9/8/2023 5:37:08 PM	**Algorithm Used**	MQ4
**Acquisition Method**	20230908-flavones-(mix130-T3)-15min.dam	**Instrument Name**	QTRAP 6500+ Low Mass
**Project**	N/A	**Processing Method**	20230412-flavones-(mix130-T3)-15min.qmethod

Regression Equation: y = 0.00366 x + -0.00104 (r = 0.99901, r² = 0.99802) (weighting: 1 / x)

Expected Concentration	Number of Values	Mean Calculated Concentration (No data for Analyte Unit)	% Accuracy	Std. Deviation	%CV
0.01	0 of 1	N/A	N/A	N/A	N/A
0.02	0 of 1	N/A	N/A	N/A	N/A
0.05	0 of 1	N/A	N/A	N/A	N/A
0.13	0 of 1	N/A	N/A	N/A	N/A
0.33	0 of 1	N/A	N/A	N/A	N/A
0.82	0 of 1	N/A	N/A	N/A	N/A
2.05	1 of 1	2.150e0	104.9	N/A	N/A
5.12	1 of 1	5.219e0	101.9	N/A	N/A
12.80	1 of 1	1.261e1	98.5	N/A	N/A
32.00	1 of 1	3.156e1	98.6	N/A	N/A
80.00	1 of 1	7.450e1	93.1	N/A	N/A
200.00	1 of 1	2.059e2	103.0	N/A	N/A

Analyte Name: LM-flavones-126_1

Created with SCIEX OS 1.6.1
Printed:9/18/2023 5:27:01 PM

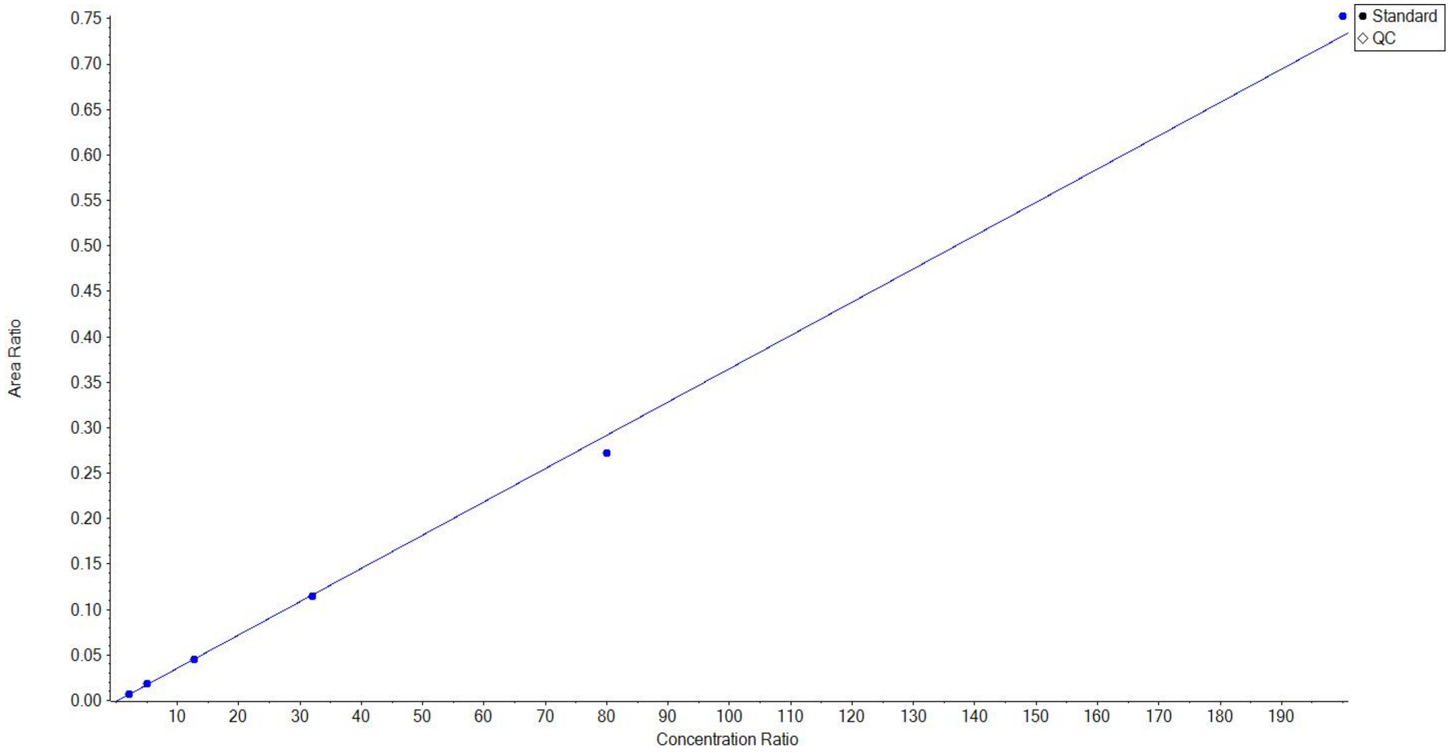

Analyte Name: LM-flavones-128_1
Internal Standard: LM-flavones-IS02_1

**Data File**	flavones-STD-20230908.wiff	**Result Table**	DZLM2023082419-results-20230913-5500
**Acquisition Date**	9/8/2023 5:37:08 PM	**Algorithm Used**	MQ4
**Acquisition Method**	20230908-flavones-(mix130-T3)-15min.dam	**Instrument Name**	QTRAP 6500+ Low Mass
**Project**	N/A	**Processing Method**	20230412-flavones-(mix130-T3)-15min.qmethod

Regression Equation: y = 1.16848e-4 x + 3.79756e-5 (r = 0.99914, r² = 0.99828) (weighting: 1 / x)

Expected Concentration	Number of Values	Mean Calculated Concentration (No data for Analyte Unit)	% Accuracy	Std. Deviation	%CV
0.01	0 of 1	N/A	N/A	N/A	N/A
0.02	0 of 1	N/A	N/A	N/A	N/A
0.05	0 of 1	N/A	N/A	N/A	N/A
0.13	0 of 1	N/A	N/A	N/A	N/A
0.33	0 of 1	N/A	N/A	N/A	N/A
0.82	0 of 1	N/A	N/A	N/A	N/A
2.05	1 of 1	2.019e0	98.6	N/A	N/A
5.12	1 of 1	5.885e0	114.9	N/A	N/A
12.80	1 of 1	1.132e1	88.4	N/A	N/A
32.00	1 of 1	3.205e1	100.2	N/A	N/A
80.00	1 of 1	7.675e1	95.9	N/A	N/A
200.00	1 of 1	2.039e2	102.0	N/A	N/A

Analyte Name: LM-flavones-128_1

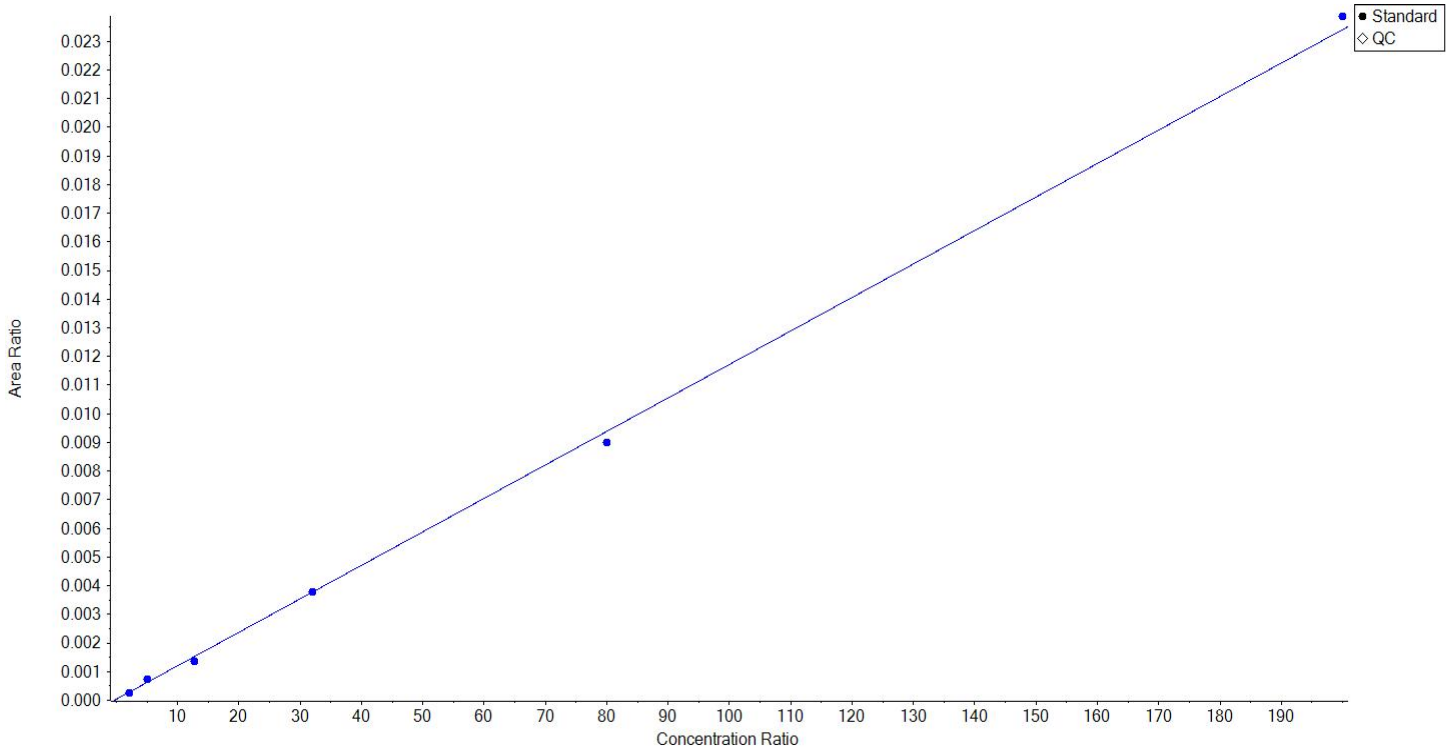
Standard
QC
Area Ratio
Concentration Ratio
0.023
0.022
0.021
0.020
0.019
0.018
0.017
0.016
0.015
0.014
0.013
0.012
0.011
0.010
0.009
0.008
0.007
0.006
0.005
0.004
0.003
0.002
0.001
0.000
10
20
30
40
50
60
70
80
90
100
110
120
130
140
150
160
170
180
190

Created with SCIEX OS 1.6.1
Printed:9/18/2023 5:27:01 PM

Analyte Name: LM-flavones-130_1
Internal Standard: LM-flavones-IS02_1

**Data File**	flavones-STD-20230908.wiff	**Result Table**	DZLM2023082419-results-20230913-5500
**Acquisition Date**	9/8/2023 5:37:08 PM	**Algorithm Used**	MQ4
**Acquisition Method**	20230908-flavones-(mix130-T3)-15min.dam	**Instrument Name**	QTRAP 6500+ Low Mass
**Project**	N/A	**Processing Method**	20230412-flavones-(mix130-T3)-15min.qmethod

Regression Equation: y = 0.00409 x + 1.26129e-5 (r = 0.99858, r² = 0.99716) (weighting: 1 / x)

Expected Concentration	Number of Values	Mean Calculated Concentration (No data for Analyte Unit)	% Accuracy	Std. Deviation	%CV
0.01	0 of 1	N/A	N/A	N/A	N/A
0.02	0 of 1	N/A	N/A	N/A	N/A
0.05	0 of 1	N/A	N/A	N/A	N/A
0.13	0 of 1	N/A	N/A	N/A	N/A
0.33	0 of 1	N/A	N/A	N/A	N/A
0.82	0 of 1	N/A	N/A	N/A	N/A
2.05	1 of 1	2.247e0	109.7	N/A	N/A
5.12	1 of 1	4.987e0	97.4	N/A	N/A
12.80	1 of 1	1.141e1	89.1	N/A	N/A
32.00	1 of 1	3.468e1	108.4	N/A	N/A
80.00	1 of 1	7.475e1	93.4	N/A	N/A
200.00	1 of 1	2.039e2	102.0	N/A	N/A

Analyte Name: LM-flavones-130_1

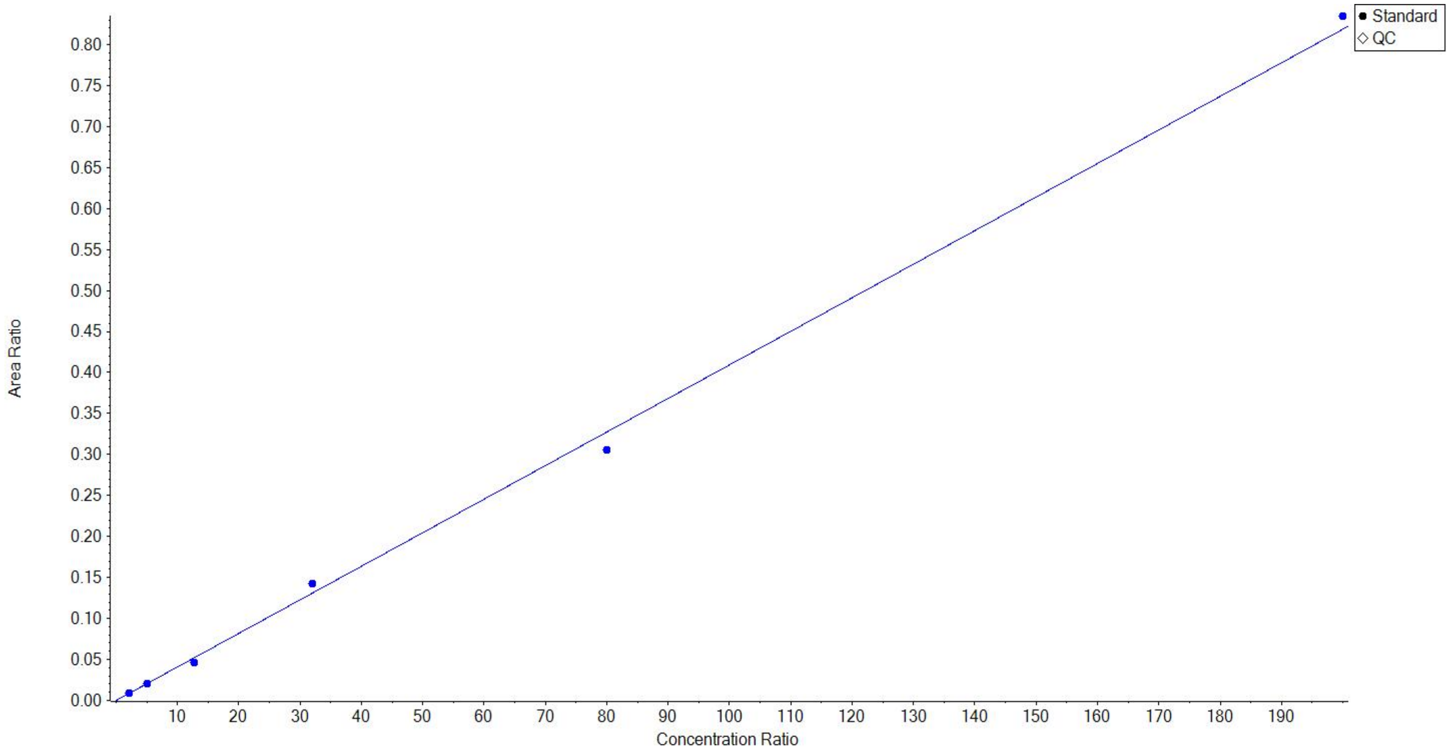
Standard
QC
Area Ratio
Concentration Ratio
0.00
0.05
0.10
0.15
0.20
0.25
0.30
0.35
0.40
0.45
0.50
0.55
0.60
0.65
0.70
0.75
0.80
10
20
30
40
50
60
70
80
90
100
110
120
130
140
150
160
170
180
190

Analyte Name: LM-flavones-16_1
Internal Standard: LM-flavones-IS02_1

**Data File**	flavones-STD-20230908.wiff	**Result Table**	DZLM2023082419-results-20230913-5500
**Acquisition Date**	9/8/2023 5:37:08 PM	**Algorithm Used**	MQ4
**Acquisition Method**	20230908-flavones-(mix130-T3)-15min.dam	**Instrument Name**	QTRAP 6500+ Low Mass
**Project**	N/A	**Processing Method**	20230412-flavones-(mix130-T3)-15min.qmethod

Regression Equation: y = 0.00833 x + -0.01213 (r = 0.99954, r² = 0.99908) (weighting: 1 / x)

Expected Concentration	Number of Values	Mean Calculated Concentration (No data for Analyte Unit)	% Accuracy	Std. Deviation	%CV
0.01	0 of 1	N/A	N/A	N/A	N/A
0.02	0 of 1	N/A	N/A	N/A	N/A
0.05	0 of 1	N/A	N/A	N/A	N/A
0.13	0 of 1	N/A	N/A	N/A	N/A
0.33	0 of 1	N/A	N/A	N/A	N/A
0.82	0 of 1	N/A	N/A	N/A	N/A
2.05	0 of 1	N/A	N/A	N/A	N/A
5.12	1 of 1	5.607e0	109.5	N/A	N/A
12.80	1 of 1	1.197e1	93.5	N/A	N/A
32.00	1 of 1	3.146e1	98.3	N/A	N/A
80.00	1 of 1	7.762e1	97.0	N/A	N/A
200.00	1 of 1	2.033e2	101.6	N/A	N/A

Analyte Name: LM-flavones-16_1

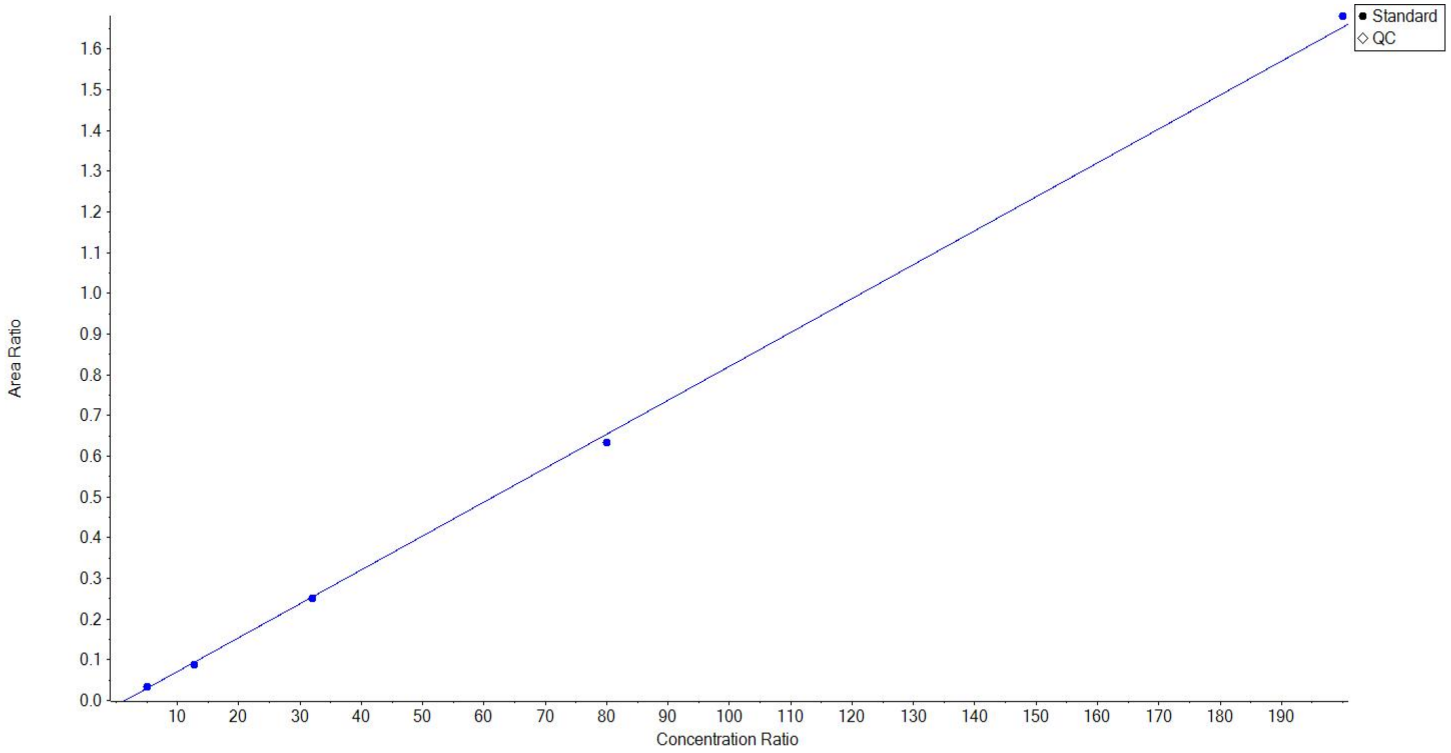
Standard
QC
Area Ratio
1.6
1.5
1.4
1.3
1.2
1.1
1.0
0.9
0.8
0.7
0.6
0.5
0.4
0.3
0.2
0.1
0.0
10
20
30
40
50
60
70
80
90
100
110
120
130
140
150
160
170
180
190
Concentration Ratio

Analyte Name: LM-flavones-17_1
Internal Standard: LM-flavones-IS02_1

**Data File**	flavones-STD-20230908.wiff	**Result Table**	DZLM2023082419-results-20230913-5500
**Acquisition Date**	9/8/2023 5:37:08 PM	**Algorithm Used**	MQ4
**Acquisition Method**	20230908-flavones-(mix130-T3)-15min.dam	**Instrument Name**	QTRAP 6500+ Low Mass
**Project**	N/A	**Processing Method**	20230412-flavones-(mix130-T3)-15min.qmethod

Regression Equation: y = 0.00570 x + -0.00398 (r = 0.99941, r² = 0.99882) (weighting: 1 / x)

Expected Concentration	Number of Values	Mean Calculated Concentration (No data for Analyte Unit)	% Accuracy	Std. Deviation	%CV
0.01	0 of 1	N/A	N/A	N/A	N/A
0.02	0 of 1	N/A	N/A	N/A	N/A
0.05	0 of 1	N/A	N/A	N/A	N/A
0.13	0 of 1	N/A	N/A	N/A	N/A
0.33	0 of 1	N/A	N/A	N/A	N/A
0.82	0 of 1	N/A	N/A	N/A	N/A
2.05	1 of 1	2.391e0	116.6	N/A	N/A
5.12	1 of 1	4.885e0	95.4	N/A	N/A
12.80	1 of 1	1.165e1	91.0	N/A	N/A
32.00	1 of 1	3.131e1	97.9	N/A	N/A
80.00	1 of 1	7.764e1	97.1	N/A	N/A
200.00	1 of 1	2.041e2	102.1	N/A	N/A

Analyte Name: LM-flavones-17_1

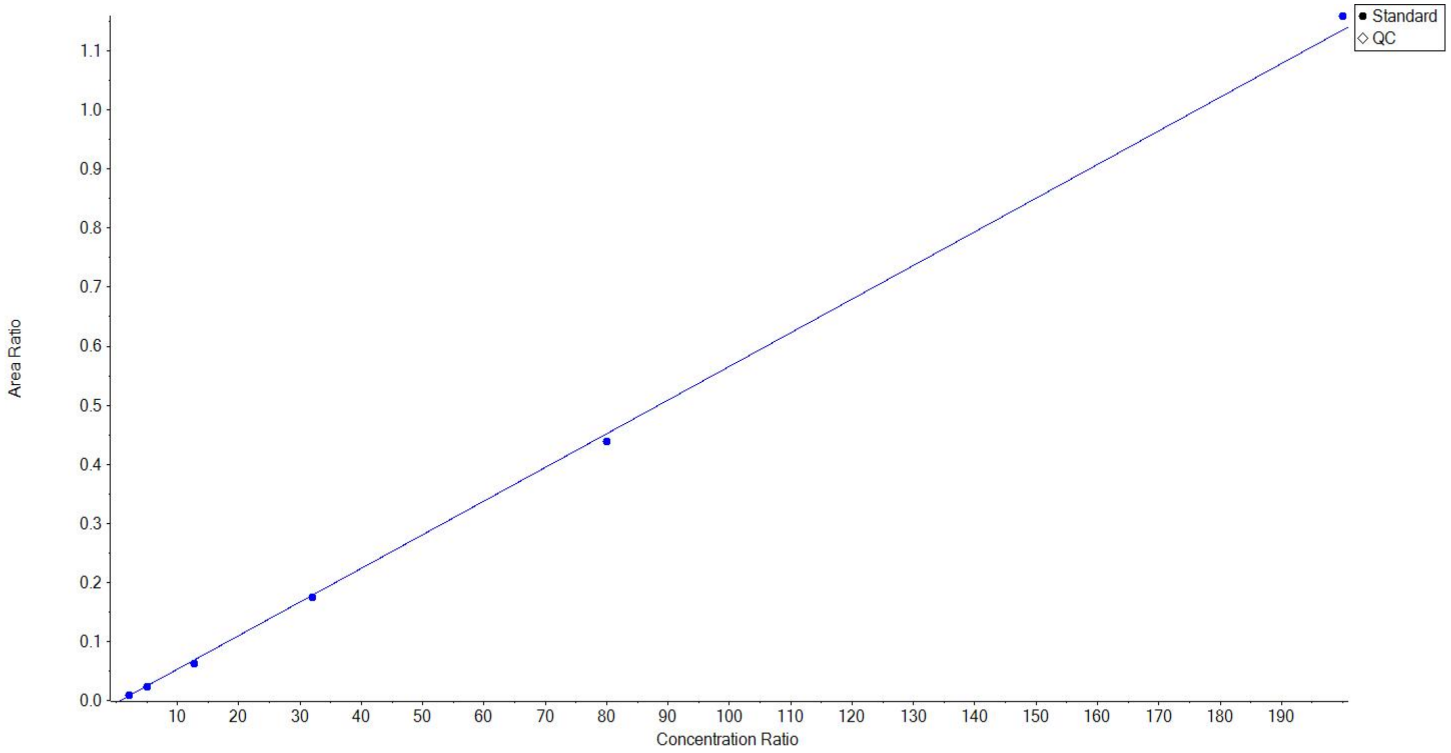
Standard
QC
Area Ratio
0.0
0.1
0.2
0.3
0.4
0.5
0.6
0.7
0.8
0.9
1.0
1.1
10
20
30
40
50
60
70
80
90
100
110
120
130
140
150
160
170
180
190
Concentration Ratio

Analyte Name: LM-flavones-21_1
Internal Standard: LM-flavones-IS02_1

**Data File**	flavones-STD-20230908.wiff	**Result Table**	DZLM2023082419-results-20230913-5500
**Acquisition Date**	9/8/2023 5:37:08 PM	**Algorithm Used**	MQ4
**Acquisition Method**	20230908-flavones-(mix130-T3)-15min.dam	**Instrument Name**	QTRAP 6500+ Low Mass
**Project**	N/A	**Processing Method**	20230412-flavones-(mix130-T3)-15min.qmethod

Regression Equation: y = 0.00527 x + 0.00319 (r = 0.99993, r² = 0.99986) (weighting: 1 / x)

Expected Concentration	Number of Values	Mean Calculated Concentration (No data for Analyte Unit)	% Accuracy	Std. Deviation	%CV
0.01	0 of 1	N/A	N/A	N/A	N/A
0.02	0 of 1	N/A	N/A	N/A	N/A
0.05	0 of 1	N/A	N/A	N/A	N/A
0.13	0 of 1	N/A	N/A	N/A	N/A
0.33	0 of 1	N/A	N/A	N/A	N/A
0.82	0 of 1	N/A	N/A	N/A	N/A
2.05	1 of 1	1.998e0	97.5	N/A	N/A
5.12	1 of 1	5.190e0	101.4	N/A	N/A
12.80	1 of 1	1.282e1	100.1	N/A	N/A
32.00	1 of 1	3.275e1	102.3	N/A	N/A
80.00	1 of 1	7.878e1	98.5	N/A	N/A
200.00	1 of 1	2.004e2	100.2	N/A	N/A

Analyte Name: LM-flavones-21_1

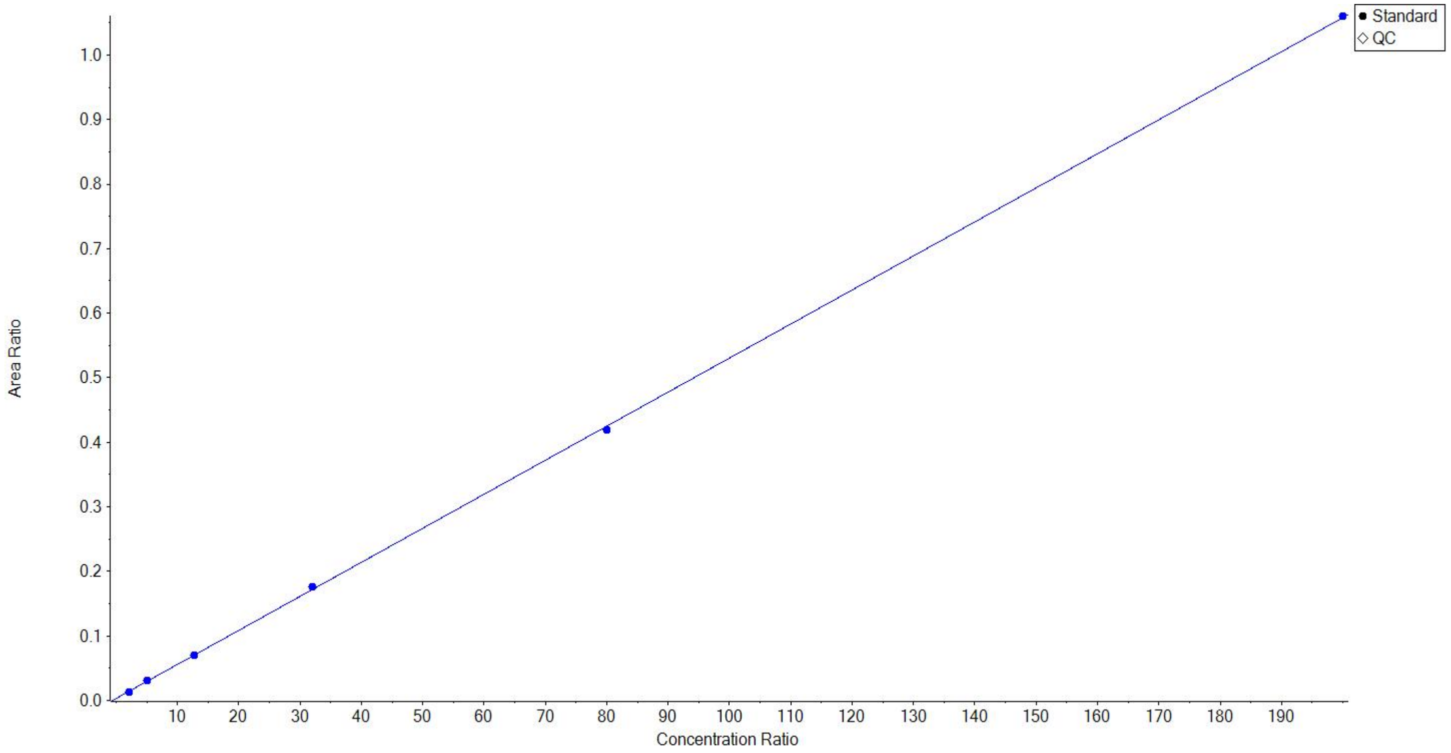
Standard
QC
Area Ratio
Concentration Ratio

Analyte Name: LM-flavones-23_1
Internal Standard: LM-flavones-IS02_1

**Data File**	flavones-STD-20230908.wiff	**Result Table**	DZLM2023082419-results-20230913-5500
**Acquisition Date**	9/8/2023 5:37:08 PM	**Algorithm Used**	MQ4
**Acquisition Method**	20230908-flavones-(mix130-T3)-15min.dam	**Instrument Name**	QTRAP 6500+ Low Mass
**Project**	N/A	**Processing Method**	20230412-flavones-(mix130-T3)-15min.qmethod

Regression Equation: y = 0.05525 x + -0.01593 (r = 0.99993, r² = 0.99985) (weighting: 1 / x)

Expected Concentration	Number of Values	Mean Calculated Concentration (No data for Analyte Unit)	% Accuracy	Std. Deviation	%CV
0.01	0 of 1	N/A	N/A	N/A	N/A
0.02	0 of 1	N/A	N/A	N/A	N/A
0.05	0 of 1	N/A	N/A	N/A	N/A
0.13	0 of 1	N/A	N/A	N/A	N/A
0.33	0 of 1	N/A	N/A	N/A	N/A
0.82	0 of 1	N/A	N/A	N/A	N/A
2.05	1 of 1	2.192e0	106.9	N/A	N/A
5.12	1 of 1	4.767e0	93.1	N/A	N/A
12.80	1 of 1	1.263e1	98.6	N/A	N/A
32.00	1 of 1	3.241e1	101.3	N/A	N/A
80.00	1 of 1	8.007e1	100.1	N/A	N/A
200.00	1 of 1	1.999e2	100.0	N/A	N/A

Analyte Name: LM-flavones-23_1

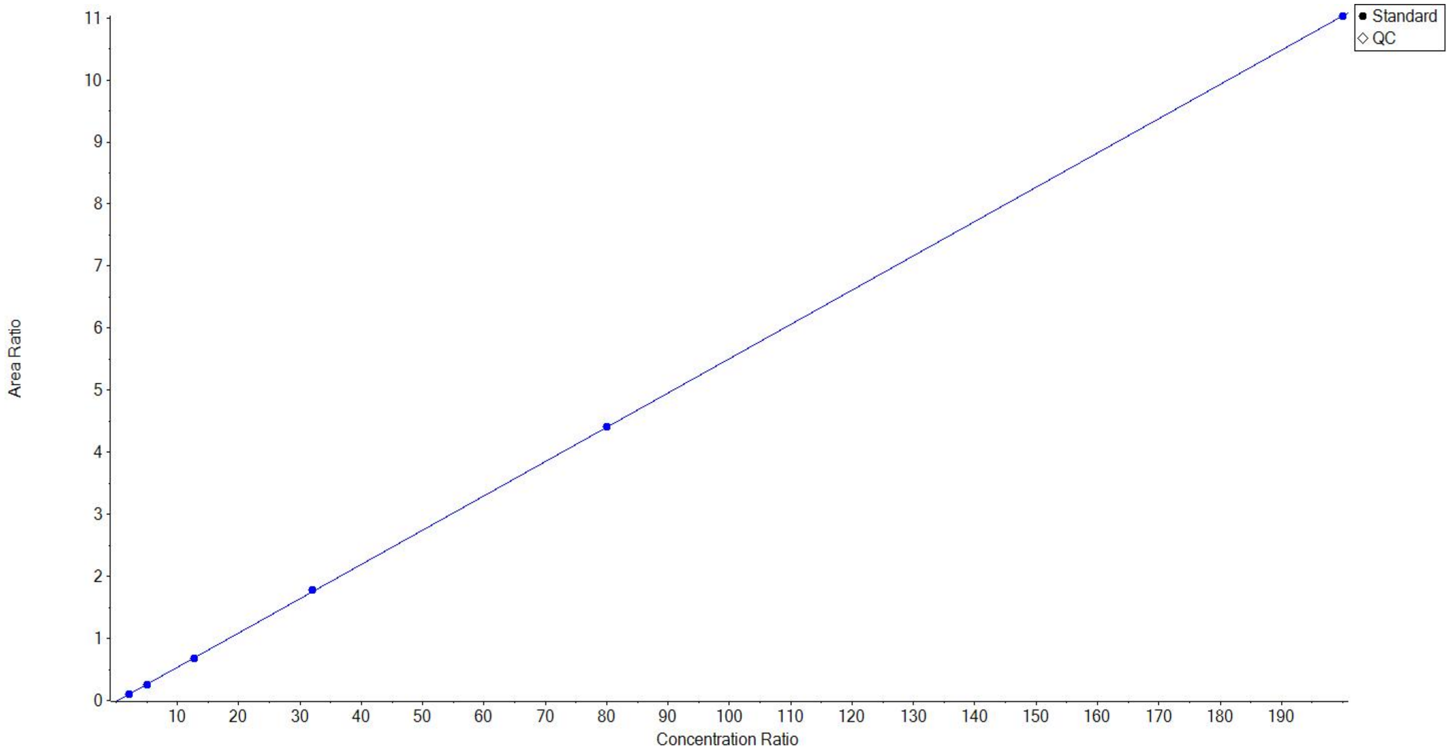
Standard
QC
Area Ratio
Concentration Ratio
0
1
2
3
4
5
6
7
8
9
10
11
10
20
30
40
50
60
70
80
90
100
110
120
130
140
150
160
170
180
190

Analyte Name: LM-flavones-24_1
Internal Standard: LM-flavones-IS02_1

**Data File**	flavones-STD-20230908.wiff	**Result Table**	DZLM2023082419-results-20230913-5500
**Acquisition Date**	9/8/2023 5:37:08 PM	**Algorithm Used**	MQ4
**Acquisition Method**	20230908-flavones-(mix130-T3)-15min.dam	**Instrument Name**	QTRAP 6500+ Low Mass
**Project**	N/A	**Processing Method**	20230412-flavones-(mix130-T3)-15min.qmethod

Regression Equation: y = 0.02260 x + -0.00744 (r = 0.99962, r² = 0.99925) (weighting: 1 / x)

Expected Concentration	Number of Values	Mean Calculated Concentration (No data for Analyte Unit)	% Accuracy	Std. Deviation	%CV
0.01	0 of 1	N/A	N/A	N/A	N/A
0.02	0 of 1	N/A	N/A	N/A	N/A
0.05	0 of 1	N/A	N/A	N/A	N/A
0.13	0 of 1	N/A	N/A	N/A	N/A
0.33	0 of 1	N/A	N/A	N/A	N/A
0.82	0 of 1	N/A	N/A	N/A	N/A
2.05	1 of 1	2.295e0	112.0	N/A	N/A
5.12	1 of 1	4.818e0	94.1	N/A	N/A
12.80	1 of 1	1.221e1	95.4	N/A	N/A
32.00	1 of 1	3.209e1	100.3	N/A	N/A
80.00	1 of 1	7.732e1	96.7	N/A	N/A
200.00	1 of 1	2.032e2	101.6	N/A	N/A

Analyte Name: LM-flavones-24_1

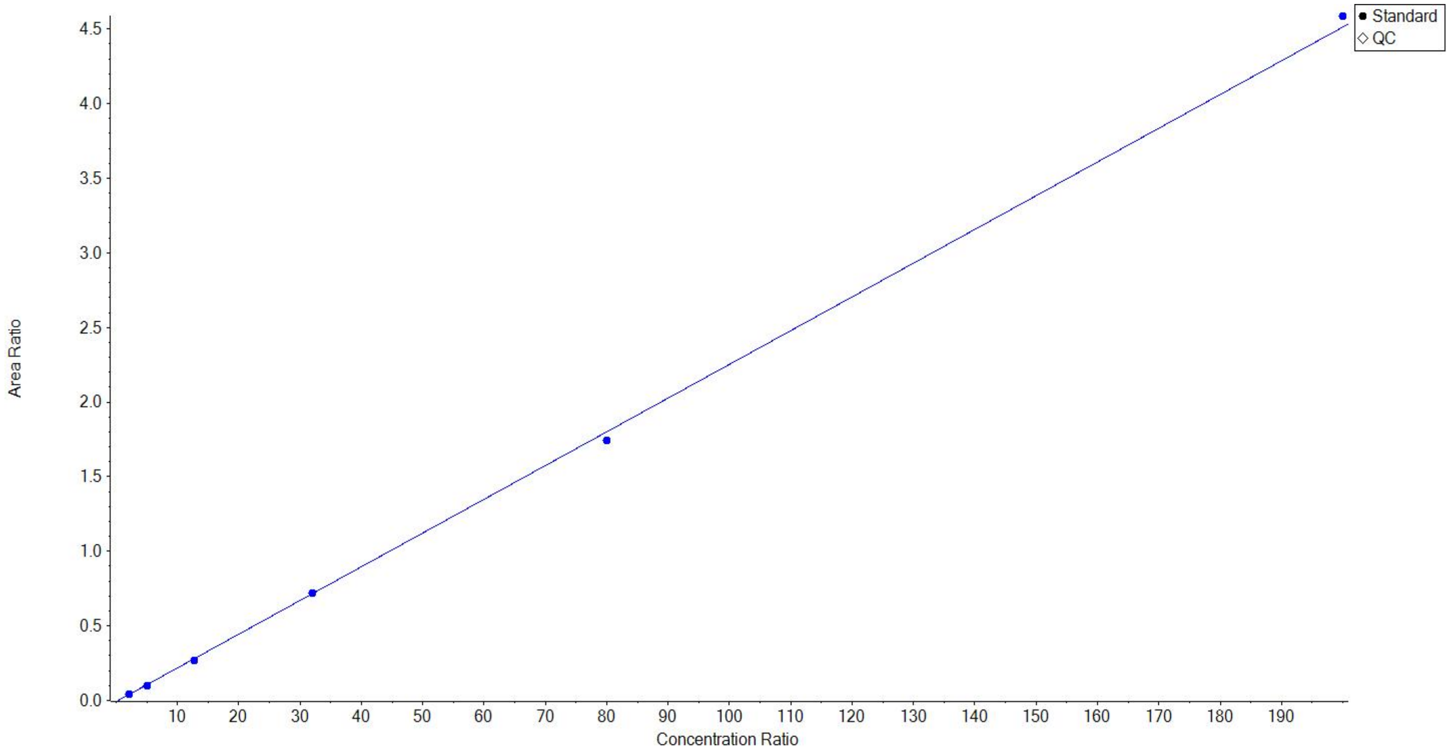
Standard
QC
Area Ratio
Concentration Ratio
4.5
4.0
3.5
3.0
2.5
2.0
1.5
1.0
0.5
0.0
10
20
30
40
50
60
70
80
90
100
110
120
130
140
150
160
170
180
190

Created with SCIEX OS 1.6.1
Printed:9/18/2023 5:27:01 PM

Analyte Name: LM-flavones-25_1
Internal Standard: LM-flavones-IS02_1

**Data File**	flavones-STD-20230908.wiff	**Result Table**	DZLM2023082419-results-20230913-5500
**Acquisition Date**	9/8/2023 5:37:08 PM	**Algorithm Used**	MQ4
**Acquisition Method**	20230908-flavones-(mix130-T3)-15min.dam	**Instrument Name**	QTRAP 6500+ Low Mass
**Project**	N/A	**Processing Method**	20230412-flavones-(mix130-T3)-15min.qmethod

Regression Equation: y = 0.00128 x + -1.72218e-4 (r = 0.99970, r² = 0.99941) (weighting: 1 / x)

Expected Concentration	Number of Values	Mean Calculated Concentration (No data for Analyte Unit)	% Accuracy	Std. Deviation	%CV
0.01	0 of 1	N/A	N/A	N/A	N/A
0.02	0 of 1	N/A	N/A	N/A	N/A
0.05	0 of 1	N/A	N/A	N/A	N/A
0.13	0 of 1	N/A	N/A	N/A	N/A
0.33	0 of 1	N/A	N/A	N/A	N/A
0.82	0 of 1	N/A	N/A	N/A	N/A
2.05	1 of 1	2.318e0	113.1	N/A	N/A
5.12	1 of 1	4.865e0	95.0	N/A	N/A
12.80	1 of 1	1.159e1	90.6	N/A	N/A
32.00	1 of 1	3.234e1	101.1	N/A	N/A
80.00	1 of 1	7.983e1	99.8	N/A	N/A
200.00	1 of 1	2.010e2	100.5	N/A	N/A

Analyte Name: LM-flavones-25_1

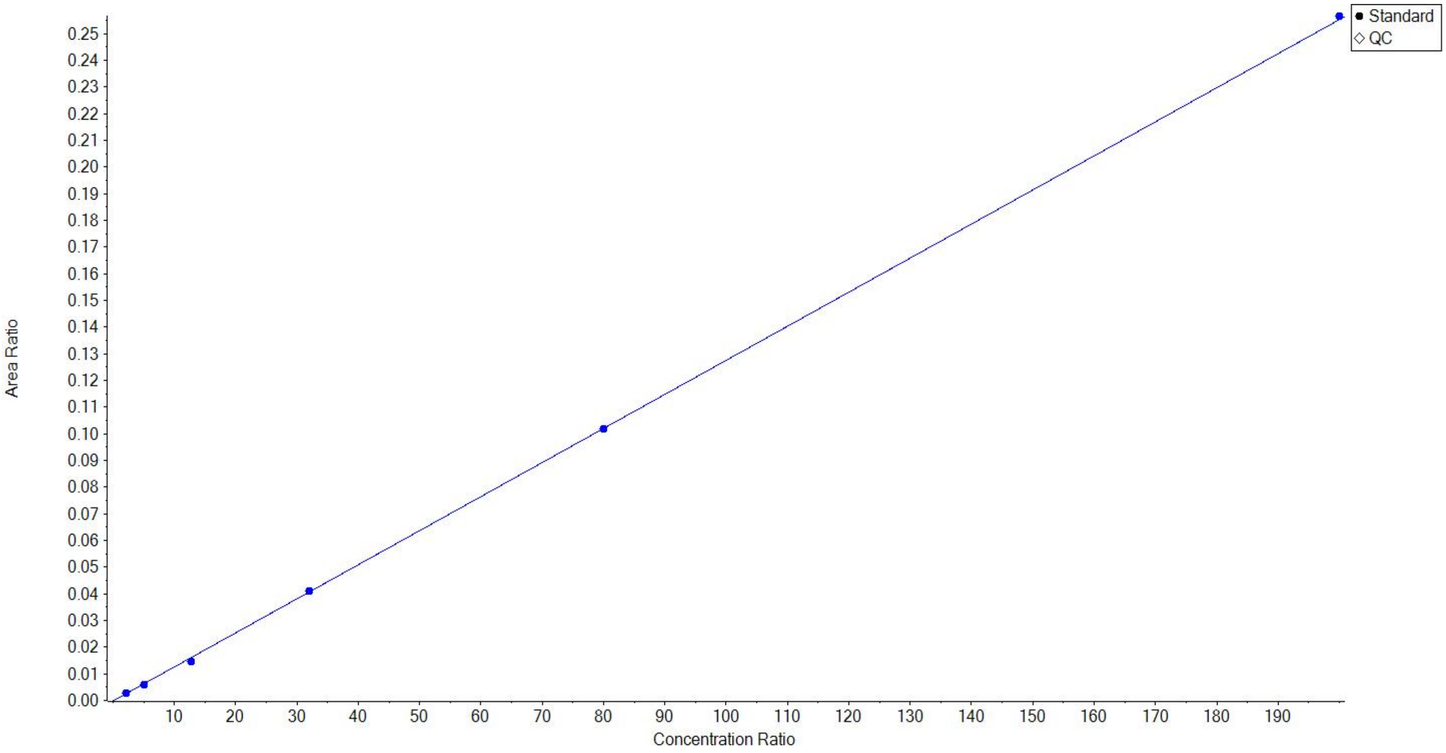
Standard
QC
Area Ratio
Concentration Ratio
0.00
0.01
0.02
0.03
0.04
0.05
0.06
0.07
0.08
0.09
0.10
0.11
0.12
0.13
0.14
0.15
0.16
0.17
0.18
0.19
0.20
0.21
0.22
0.23
0.24
0.25
10
20
30
40
50
60
70
80
90
100
110
120
130
140
150
160
170
180
190

Analyte Name: LM-flavones-27_1
Internal Standard: LM-flavones-IS02_1

**Data File**	flavones-STD-20230908.wiff	**Result Table**	DZLM2023082419-results-20230913-5500
**Acquisition Date**	9/8/2023 5:37:08 PM	**Algorithm Used**	MQ4
**Acquisition Method**	20230908-flavones-(mix130-T3)-15min.dam	**Instrument Name**	QTRAP 6500+ Low Mass
**Project**	N/A	**Processing Method**	20230412-flavones-(mix130-T3)-15min.qmethod

Regression Equation: y = 0.00666 x + 9.26026e-4 (r = 0.99983, r² = 0.99966) (weighting: 1 / x)

Expected Concentration	Number of Values	Mean Calculated Concentration (No data for Analyte Unit)	% Accuracy	Std. Deviation	%CV
0.01	0 of 1	N/A	N/A	N/A	N/A
0.02	0 of 1	N/A	N/A	N/A	N/A
0.05	0 of 1	N/A	N/A	N/A	N/A
0.13	0 of 1	N/A	N/A	N/A	N/A
0.33	0 of 1	N/A	N/A	N/A	N/A
0.82	0 of 1	N/A	N/A	N/A	N/A
2.05	1 of 1	2.028e0	98.9	N/A	N/A
5.12	1 of 1	5.036e0	98.4	N/A	N/A
12.80	1 of 1	1.303e1	101.8	N/A	N/A
32.00	1 of 1	3.298e1	103.1	N/A	N/A
80.00	1 of 1	7.785e1	97.3	N/A	N/A
200.00	1 of 1	2.010e2	100.5	N/A	N/A

Analyte Name: LM-flavones-27_1

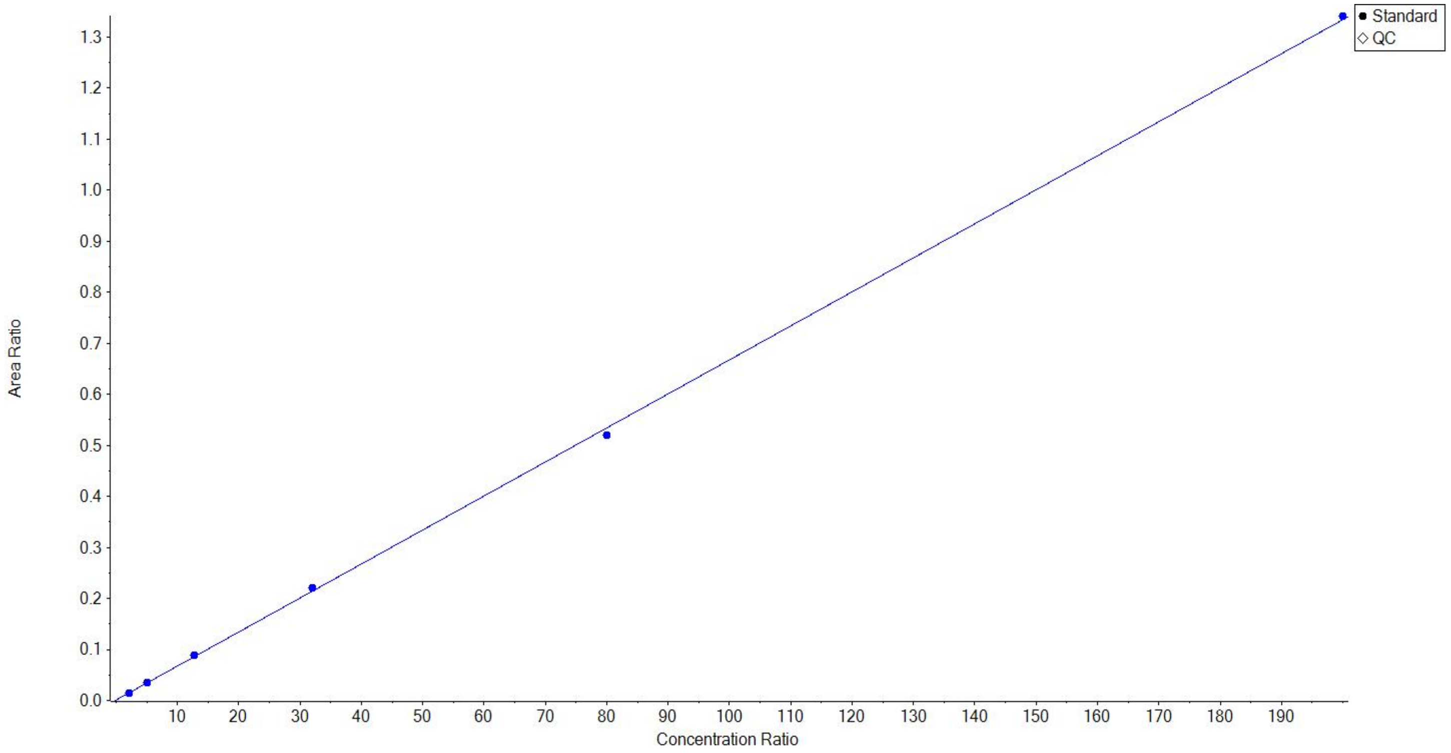
Standard
QC
Area Ratio
1.3
1.2
1.1
1.0
0.9
0.8
0.7
0.6
0.5
0.4
0.3
0.2
0.1
0.0
10
20
30
40
50
60
70
80
90
100
110
120
130
140
150
160
170
180
190
Concentration Ratio

Analyte Name: LM-flavones-41_1
Internal Standard: LM-flavones-IS02_1

**Data File**	flavones-STD-20230908.wiff	**Result Table**	DZLM2023082419-results-20230913-5500
**Acquisition Date**	9/8/2023 5:37:08 PM	**Algorithm Used**	MQ4
**Acquisition Method**	20230908-flavones-(mix130-T3)-15min.dam	**Instrument Name**	QTRAP 6500+ Low Mass
**Project**	N/A	**Processing Method**	20230412-flavones-(mix130-T3)-15min.qmethod

Regression Equation: y = 0.00369 x + 4.07096e-4 (r = 0.99985, r^2 = 0.99970) (weighting: 1 / x)

Expected Concentration	Number of Values	Mean Calculated Concentration (No data for Analyte Unit)	% Accuracy	Std. Deviation	%CV
0.01	0 of 1	N/A	N/A	N/A	N/A
0.02	0 of 1	N/A	N/A	N/A	N/A
0.05	0 of 1	N/A	N/A	N/A	N/A
0.13	0 of 1	N/A	N/A	N/A	N/A
0.33	0 of 1	N/A	N/A	N/A	N/A
0.82	0 of 1	N/A	N/A	N/A	N/A
2.05	1 of 1	2.046e0	99.8	N/A	N/A
5.12	1 of 1	5.015e0	98.0	N/A	N/A
12.80	1 of 1	1.269e1	99.1	N/A	N/A
32.00	1 of 1	3.347e1	104.6	N/A	N/A
80.00	1 of 1	7.888e1	98.6	N/A	N/A
200.00	1 of 1	1.999e2	99.9	N/A	N/A

Analyte Name: LM-flavones-41_1

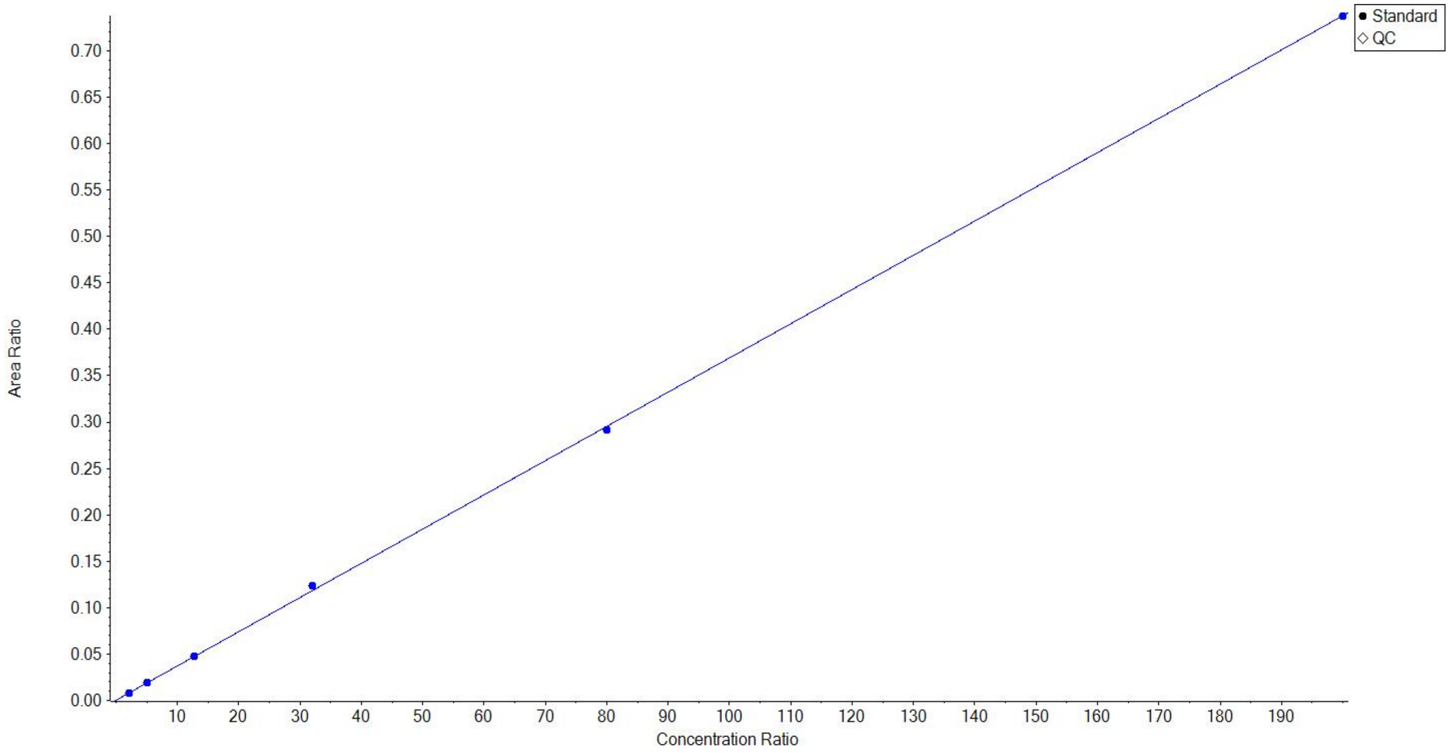
Standard
QC
Area Ratio
0.00
0.05
0.10
0.15
0.20
0.25
0.30
0.35
0.40
0.45
0.50
0.55
0.60
0.65
0.70
10
20
30
40
50
60
70
80
90
100
110
120
130
140
150
160
170
180
190
Concentration Ratio

Analyte Name: LM-flavones-44_1
Internal Standard: LM-flavones-IS02_1

**Data File**	flavones-STD-20230908.wiff	**Result Table**	DZLM2023082419-results-20230913-5500
**Acquisition Date**	9/8/2023 5:37:08 PM	**Algorithm Used**	MQ4
**Acquisition Method**	20230908-flavones-(mix130-T3)-15min.dam	**Instrument Name**	QTRAP 6500+ Low Mass
**Project**	N/A	**Processing Method**	20230412-flavones-(mix130-T3)-15min.qmethod

Regression Equation: y = 0.00714 x + -0.00110 (r = 0.99887, r² = 0.99775) (weighting: 1 / x)

Expected Concentration	Number of Values	Mean Calculated Concentration (No data for Analyte Unit)	% Accuracy	Std. Deviation	%CV
0.01	0 of 1	N/A	N/A	N/A	N/A
0.02	0 of 1	N/A	N/A	N/A	N/A
0.05	0 of 1	N/A	N/A	N/A	N/A
0.13	0 of 1	N/A	N/A	N/A	N/A
0.33	0 of 1	N/A	N/A	N/A	N/A
0.82	0 of 1	N/A	N/A	N/A	N/A
2.05	1 of 1	2.023e0	98.8	N/A	N/A
5.12	1 of 1	5.358e0	104.7	N/A	N/A
12.80	1 of 1	1.240e1	96.9	N/A	N/A
32.00	1 of 1	3.355e1	104.8	N/A	N/A
80.00	1 of 1	7.404e1	92.6	N/A	N/A
200.00	1 of 1	2.046e2	102.3	N/A	N/A

Analyte Name: LM-flavones-44_1

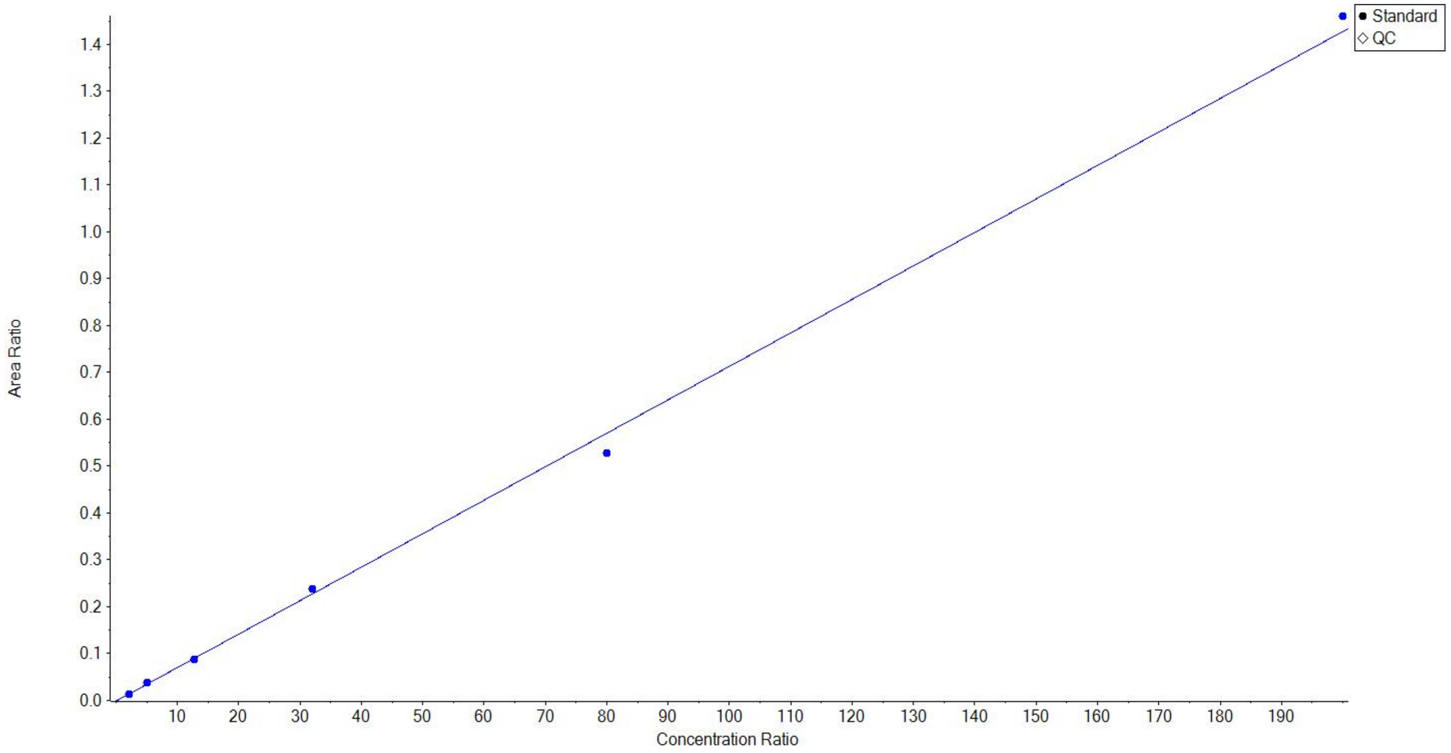
Standard
QC
Area Ratio
0.0
0.1
0.2
0.3
0.4
0.5
0.6
0.7
0.8
0.9
1.0
1.1
1.2
1.3
1.4
10
20
30
40
50
60
70
80
90
100
110
120
130
140
150
160
170
180
190
Concentration Ratio

Analyte Name: LM-flavones-47_1
Internal Standard: LM-flavones-IS02_1

**Data File**	flavones-STD-20230908.wiff	**Result Table**	DZLM2023082419-results-20230913-5500
**Acquisition Date**	9/8/2023 5:37:08 PM	**Algorithm Used**	MQ4
**Acquisition Method**	20230908-flavones-(mix130-T3)-15min.dam	**Instrument Name**	QTRAP 6500+ Low Mass
**Project**	N/A	**Processing Method**	20230412-flavones-(mix130-T3)-15min.qmethod

Regression Equation: y = 0.00229 x + -0.00103 (r = 0.99934, r² = 0.99869) (weighting: 1 / x)

Expected Concentration	Number of Values	Mean Calculated Concentration (No data for Analyte Unit)	% Accuracy	Std. Deviation	%CV
0.01	0 of 1	N/A	N/A	N/A	N/A
0.02	0 of 1	N/A	N/A	N/A	N/A
0.05	0 of 1	N/A	N/A	N/A	N/A
0.13	0 of 1	N/A	N/A	N/A	N/A
0.33	0 of 1	N/A	N/A	N/A	N/A
0.82	0 of 1	N/A	N/A	N/A	N/A
2.05	1 of 1	2.341e0	114.2	N/A	N/A
5.12	1 of 1	4.890e0	95.5	N/A	N/A
12.80	1 of 1	1.170e1	91.4	N/A	N/A
32.00	1 of 1	3.235e1	101.1	N/A	N/A
80.00	1 of 1	7.661e1	95.8	N/A	N/A
200.00	1 of 1	2.041e2	102.0	N/A	N/A

Analyte Name: LM-flavones-47_1

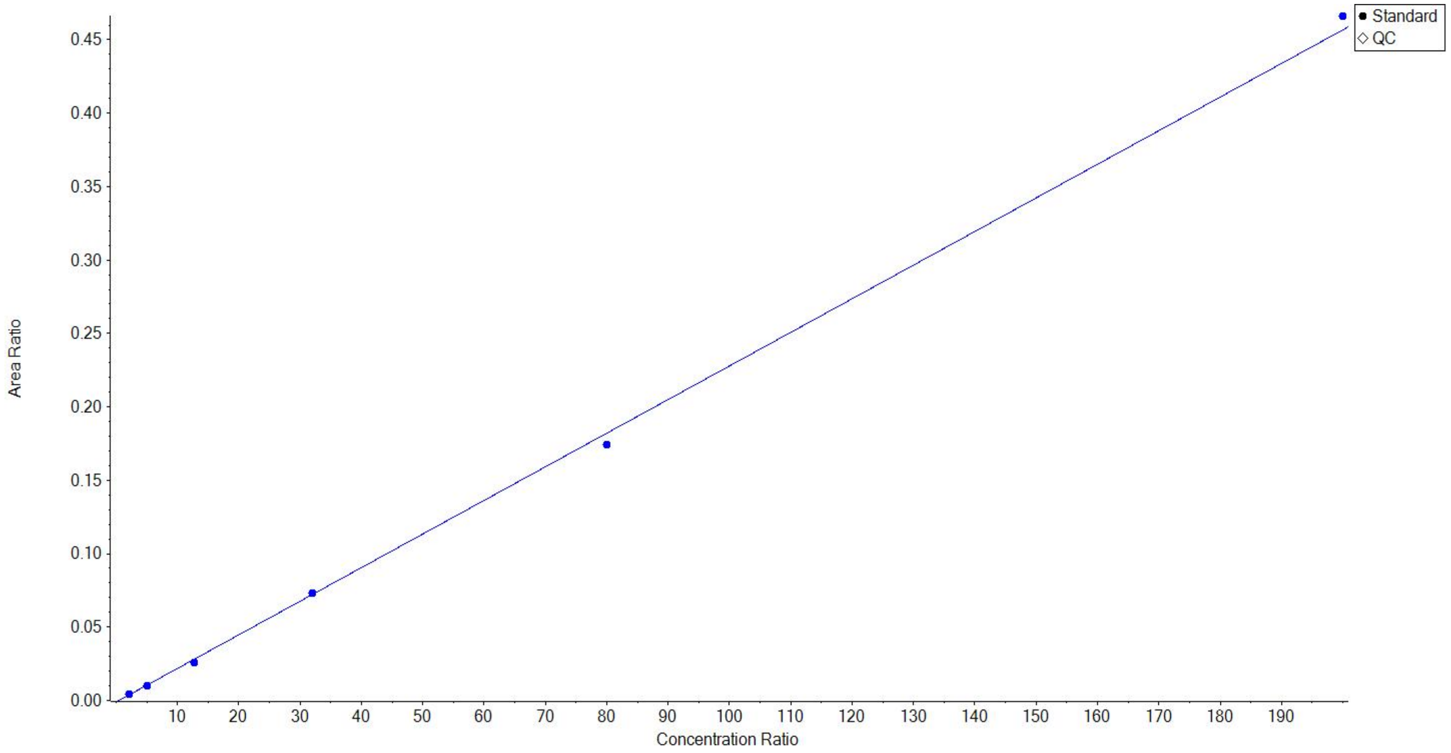
Standard
QC
Area Ratio
0.45
0.40
0.35
0.30
0.25
0.20
0.15
0.10
0.05
0.00
10 20 30 40 50 60 70 80 90 100 110 120 130 140 150 160 170 180 190
Concentration Ratio

Analyte Name: LM-flavones-48_1
Internal Standard: LM-flavones-IS02_1

**Data File**	flavones-STD-20230908.wiff	**Result Table**	DZLM2023082419-results-20230913-5500
**Acquisition Date**	9/8/2023 5:37:08 PM	**Algorithm Used**	MQ4
**Acquisition Method**	20230908-flavones-(mix130-T3)-15min.dam	**Instrument Name**	QTRAP 6500+ Low Mass
**Project**	N/A	**Processing Method**	20230412-flavones-(mix130-T3)-15min.qmethod

Regression Equation: y = 0.00898 x + -0.00132 (r = 0.99986, r² = 0.99973) (weighting: 1 / x)

Expected Concentration	Number of Values	Mean Calculated Concentration (No data for Analyte Unit)	% Accuracy	Std. Deviation	%CV
0.01	0 of 1	N/A	N/A	N/A	N/A
0.02	0 of 1	N/A	N/A	N/A	N/A
0.05	0 of 1	N/A	N/A	N/A	N/A
0.13	0 of 1	N/A	N/A	N/A	N/A
0.33	0 of 1	N/A	N/A	N/A	N/A
0.82	0 of 1	N/A	N/A	N/A	N/A
2.05	1 of 1	2.162e0	105.5	N/A	N/A
5.12	1 of 1	4.963e0	96.9	N/A	N/A
12.80	1 of 1	1.235e1	96.5	N/A	N/A
32.00	1 of 1	3.275e1	102.3	N/A	N/A
80.00	1 of 1	7.854e1	98.2	N/A	N/A
200.00	1 of 1	2.012e2	100.6	N/A	N/A

Analyte Name: LM-flavones-48_1

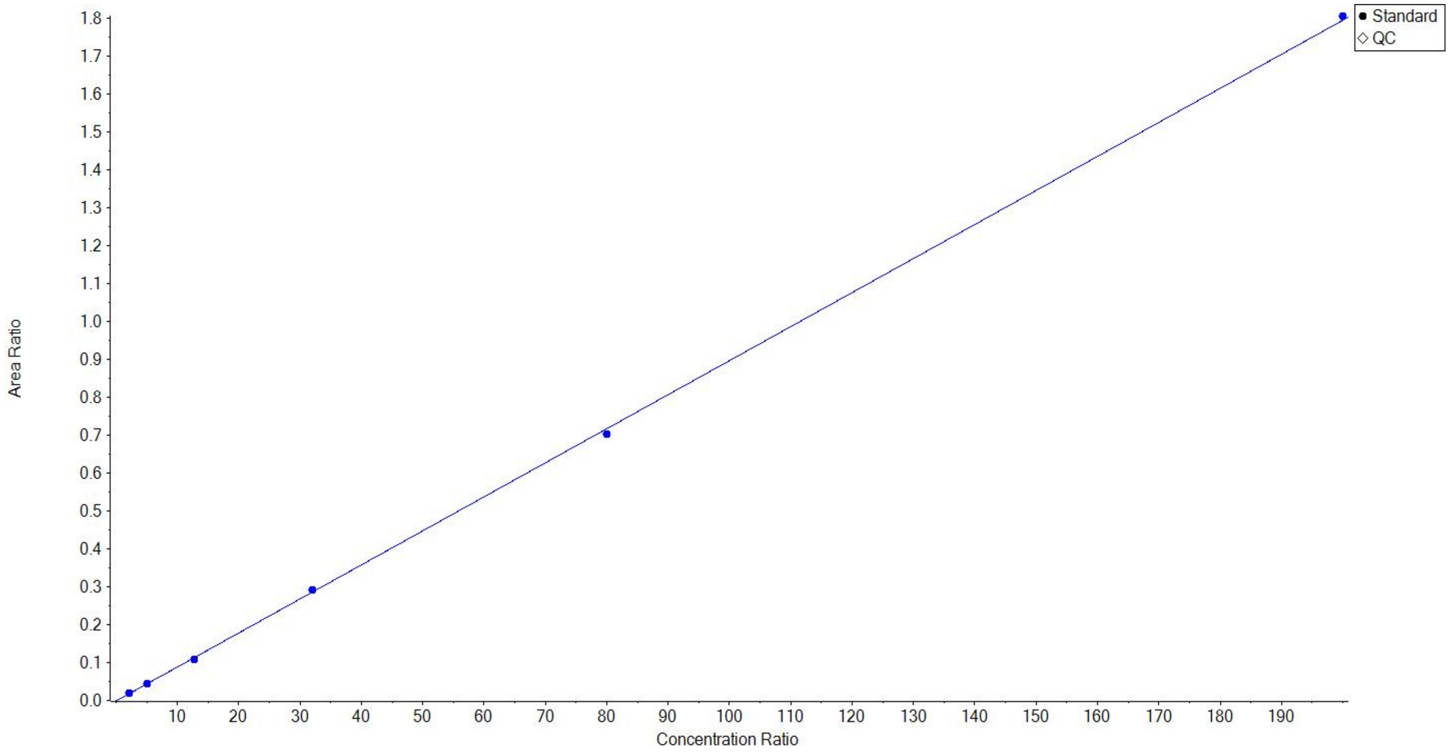
Standard
QC
Area Ratio
0.0
0.1
0.2
0.3
0.4
0.5
0.6
0.7
0.8
0.9
1.0
1.1
1.2
1.3
1.4
1.5
1.6
1.7
1.8
10
20
30
40
50
60
70
80
90
100
110
120
130
140
150
160
170
180
190
Concentration Ratio

Analyte Name: LM-flavones-49_1
Internal Standard: LM-flavones-IS02_1

**Data File**	flavones-STD-20230908.wiff	**Result Table**	DZLM2023082419-results-20230913-5500
**Acquisition Date**	9/8/2023 5:37:08 PM	**Algorithm Used**	MQ4
**Acquisition Method**	20230908-flavones-(mix130-T3)-15min.dam	**Instrument Name**	QTRAP 6500+ Low Mass
**Project**	N/A	**Processing Method**	20230412-flavones-(mix130-T3)-15min.qmethod

Regression Equation: y = 0.00196 x + 3.41516e-4 (r = 0.99991, r^2 = 0.99982) (weighting: 1 / x)

Expected Concentration	Number of Values	Mean Calculated Concentration (No data for Analyte Unit)	% Accuracy	Std. Deviation	%CV
0.01	0 of 1	N/A	N/A	N/A	N/A
0.02	0 of 1	N/A	N/A	N/A	N/A
0.05	0 of 1	N/A	N/A	N/A	N/A
0.13	0 of 1	N/A	N/A	N/A	N/A
0.33	0 of 1	N/A	N/A	N/A	N/A
0.82	1 of 1	8.898e-1	108.5	N/A	N/A
2.05	1 of 1	2.003e0	97.7	N/A	N/A
5.12	1 of 1	4.746e0	92.7	N/A	N/A
12.80	1 of 1	1.256e1	98.1	N/A	N/A
32.00	1 of 1	3.285e1	102.7	N/A	N/A
80.00	1 of 1	8.049e1	100.6	N/A	N/A
200.00	1 of 1	1.993e2	99.7	N/A	N/A

Analyte Name: LM-flavones-49_1

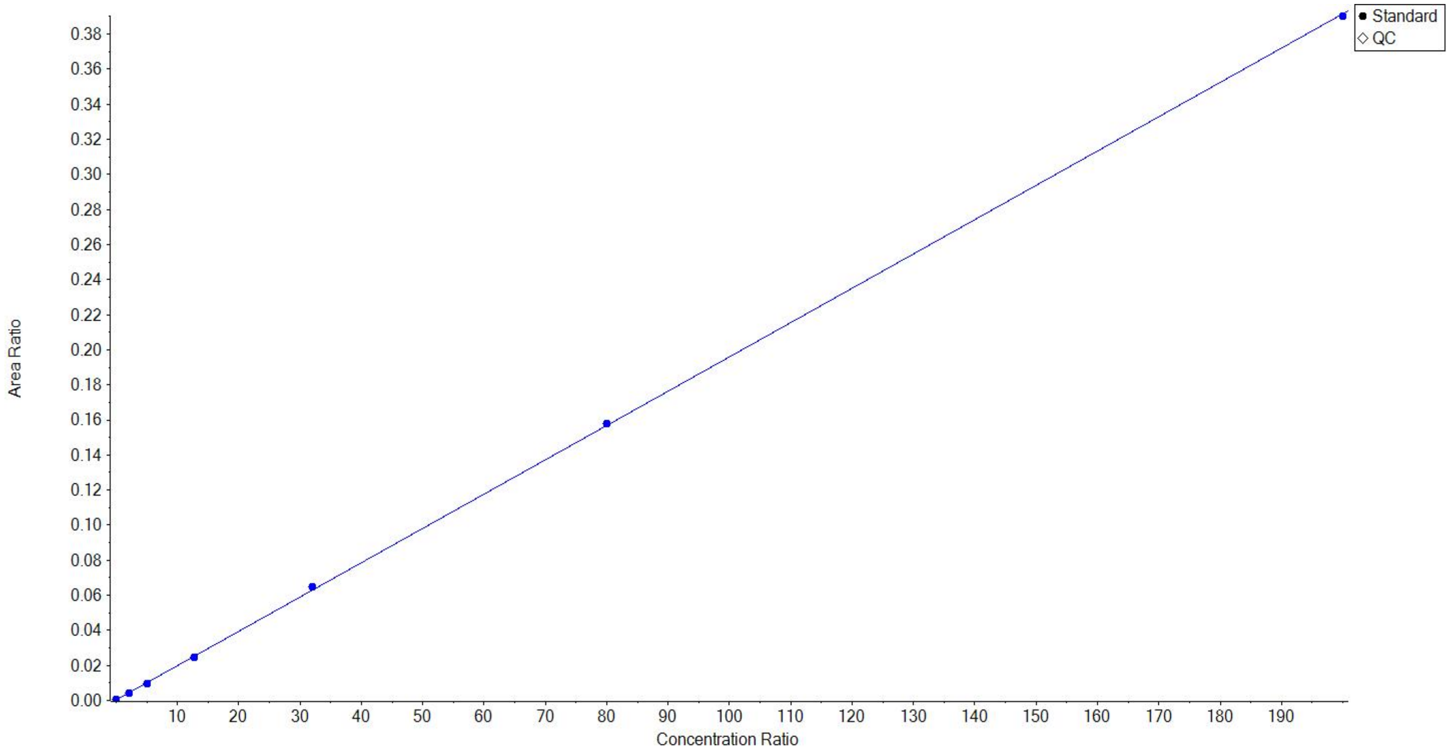
Standard
QC
Area Ratio
Concentration Ratio
0.00
0.02
0.04
0.06
0.08
0.10
0.12
0.14
0.16
0.18
0.20
0.22
0.24
0.26
0.28
0.30
0.32
0.34
0.36
0.38
10
20
30
40
50
60
70
80
90
100
110
120
130
140
150
160
170
180
190

Analyte Name: LM-flavones-50_1
Internal Standard: LM-flavones-IS02_1

**Data File**	flavones-STD-20230908.wiff	**Result Table**	DZLM2023082419-results-20230913-5500
**Acquisition Date**	9/8/2023 5:37:08 PM	**Algorithm Used**	MQ4
**Acquisition Method**	20230908-flavones-(mix130-T3)-15min.dam	**Instrument Name**	QTRAP 6500+ Low Mass
**Project**	N/A	**Processing Method**	20230412-flavones-(mix130-T3)-15min.qmethod

Regression Equation: y = 0.00366 x + 0.00139 (r = 0.99970, r² = 0.99940) (weighting: 1 / x)

Expected Concentration	Number of Values	Mean Calculated Concentration (No data for Analyte Unit)	% Accuracy	Std. Deviation	%CV
0.01	0 of 1	N/A	N/A	N/A	N/A
0.02	0 of 1	N/A	N/A	N/A	N/A
0.05	0 of 1	N/A	N/A	N/A	N/A
0.13	0 of 1	N/A	N/A	N/A	N/A
0.33	0 of 1	N/A	N/A	N/A	N/A
0.82	0 of 1	N/A	N/A	N/A	N/A
2.05	1 of 1	1.771e0	86.4	N/A	N/A
5.12	1 of 1	5.602e0	109.4	N/A	N/A
12.80	1 of 1	1.297e1	101.3	N/A	N/A
32.00	1 of 1	3.348e1	104.6	N/A	N/A
80.00	1 of 1	7.891e1	98.6	N/A	N/A
200.00	1 of 1	1.992e2	99.6	N/A	N/A

Analyte Name: LM-flavones-50_1

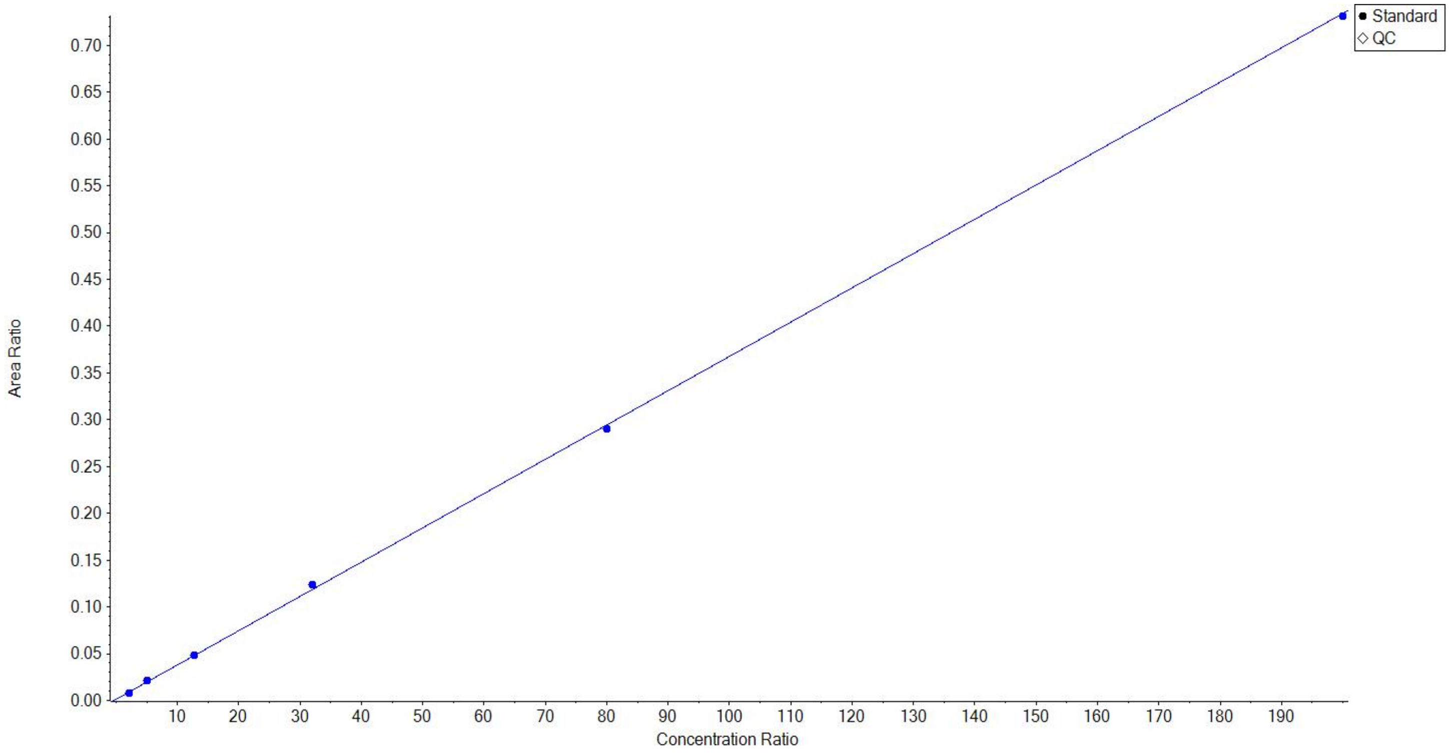
Standard
QC
Area Ratio
0.70
0.65
0.60
0.55
0.50
0.45
0.40
0.35
0.30
0.25
0.20
0.15
0.10
0.05
0.00
10
20
30
40
50
60
70
80
90
100
110
120
130
140
150
160
170
180
190
Concentration Ratio

Analyte Name: LM-flavones-51
Internal Standard: LM-flavones-IS02_1

**Data File**	flavones-STD-20230908.wiff	**Result Table**	DZLM2023082419-results-20230913-5500
**Acquisition Date**	9/8/2023 5:37:08 PM	**Algorithm Used**	MQ4
**Acquisition Method**	20230908-flavones-(mix130-T3)-15min.dam	**Instrument Name**	QTRAP 6500+ Low Mass
**Project**	N/A	**Processing Method**	20230412-flavones-(mix130-T3)-15min.qmethod

Regression Equation: y = 0.01071 x + 0.00234 (r = 0.99973, r^2 = 0.99946) (weighting: 1 / x)

Expected Concentration	Number of Values	Mean Calculated Concentration (No data for Analyte Unit)	% Accuracy	Std. Deviation	%CV
0.01	0 of 1	N/A	N/A	N/A	N/A
0.02	0 of 1	N/A	N/A	N/A	N/A
0.05	0 of 1	N/A	N/A	N/A	N/A
0.13	0 of 1	N/A	N/A	N/A	N/A
0.33	0 of 1	N/A	N/A	N/A	N/A
0.82	0 of 1	N/A	N/A	N/A	N/A
2.05	1 of 1	2.052e0	100.1	N/A	N/A
5.12	1 of 1	4.913e0	96.0	N/A	N/A
12.80	1 of 1	1.276e1	99.7	N/A	N/A
32.00	1 of 1	3.397e1	106.2	N/A	N/A
80.00	1 of 1	7.863e1	98.3	N/A	N/A
200.00	1 of 1	1.996e2	99.8	N/A	N/A

Analyte Name: LM-flavones-51

Created with SCIEX OS 1.6.1
Printed:9/18/2023 5:27:01 PM

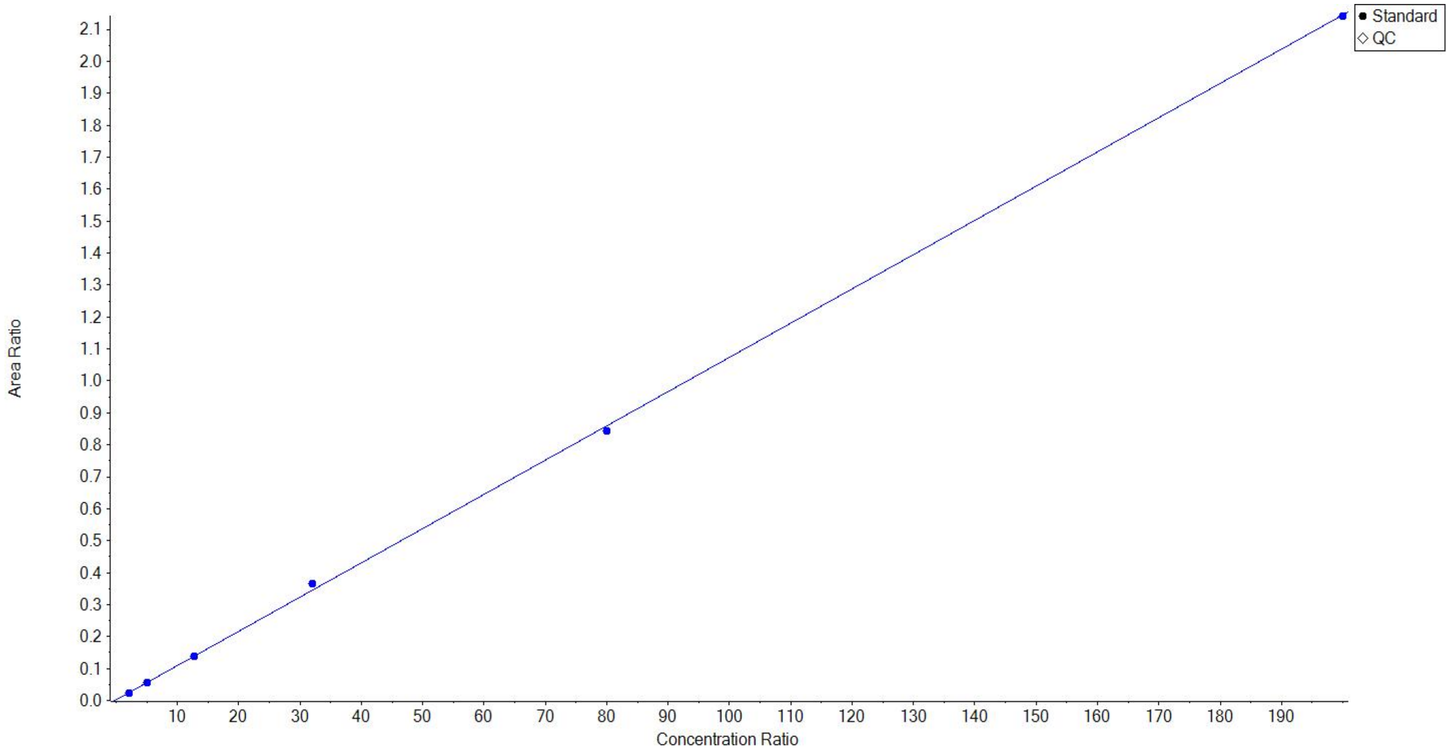

Analyte Name: LM-flavones-53
Internal Standard: LM-flavones-IS02_1

**Data File**	flavones-STD-20230908.wiff	**Result Table**	DZLM2023082419-results-20230913-5500
**Acquisition Date**	9/8/2023 5:37:08 PM	**Algorithm Used**	MQ4
**Acquisition Method**	20230908-flavones-(mix130-T3)-15min.dam	**Instrument Name**	QTRAP 6500+ Low Mass
**Project**	N/A	**Processing Method**	20230412-flavones-(mix130-T3)-15min.qmethod

Regression Equation: y = 0.01114 x + 0.00978 (r = 0.99691, r² = 0.99383) (weighting: 1 / x)

Expected Concentration	Number of Values	Mean Calculated Concentration (No data for Analyte Unit)	% Accuracy	Std. Deviation	%CV
0.01	0 of 1	N/A	N/A	N/A	N/A
0.02	0 of 1	N/A	N/A	N/A	N/A
0.05	0 of 1	N/A	N/A	N/A	N/A
0.13	0 of 1	N/A	N/A	N/A	N/A
0.33	0 of 1	N/A	N/A	N/A	N/A
0.82	1 of 1	1.339e0	163.3	N/A	N/A
2.05	1 of 1	1.185e0	57.8	N/A	N/A
5.12	1 of 1	4.082e0	79.7	N/A	N/A
12.80	1 of 1	1.171e1	91.5	N/A	N/A
32.00	1 of 1	3.411e1	106.6	N/A	N/A
80.00	1 of 1	8.088e1	101.1	N/A	N/A
200.00	0 of 1	N/A	N/A	N/A	N/A

Analyte Name: LM-flavones-53

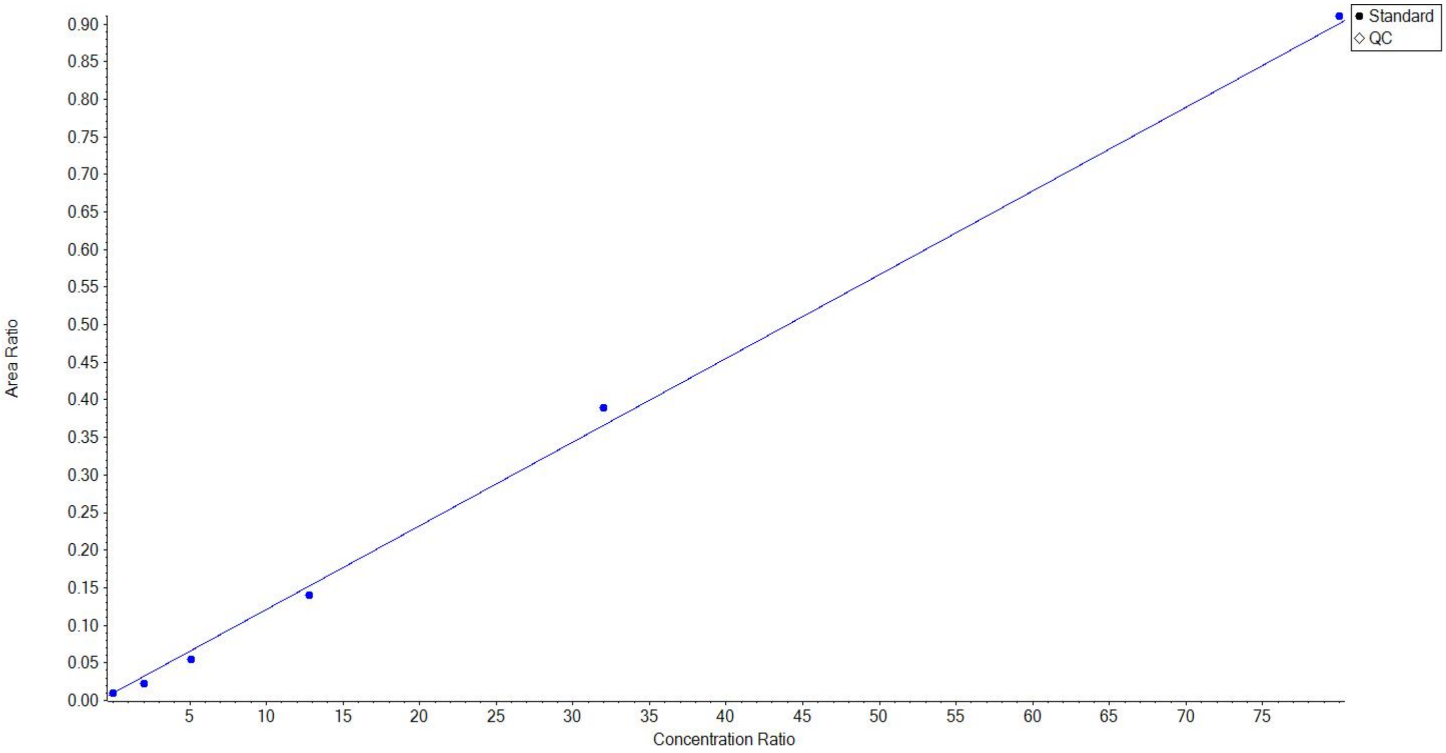
Standard
QC
Area Ratio
Concentration Ratio
0.00
0.05
0.10
0.15
0.20
0.25
0.30
0.35
0.40
0.45
0.50
0.55
0.60
0.65
0.70
0.75
0.80
0.85
0.90
5
10
15
20
25
30
35
40
45
50
55
60
65
70
75

Analyte Name: LM-flavones-61_1
Internal Standard: LM-flavones-IS02_1

**Data File**	flavones-STD-20230908.wiff	**Result Table**	DZLM2023082419-results-20230913-5500
**Acquisition Date**	9/8/2023 5:37:08 PM	**Algorithm Used**	MQ4
**Acquisition Method**	20230908-flavones-(mix130-T3)-15min.dam	**Instrument Name**	QTRAP 6500+ Low Mass
**Project**	N/A	**Processing Method**	20230412-flavones-(mix130-T3)-15min.qmethod

Regression Equation: y = 0.00193 x + -7.81768e-4 (r = 0.99977, r² = 0.99953) (weighting: 1 / x)

Expected Concentration	Number of Values	Mean Calculated Concentration (No data for Analyte Unit)	% Accuracy	Std. Deviation	%CV
0.01	0 of 1	N/A	N/A	N/A	N/A
0.02	0 of 1	N/A	N/A	N/A	N/A
0.05	0 of 1	N/A	N/A	N/A	N/A
0.13	0 of 1	N/A	N/A	N/A	N/A
0.33	0 of 1	N/A	N/A	N/A	N/A
0.82	0 of 1	N/A	N/A	N/A	N/A
2.05	0 of 1	N/A	N/A	N/A	N/A
5.12	1 of 1	4.689e0	91.6	N/A	N/A
12.80	1 of 1	1.362e1	106.4	N/A	N/A
32.00	1 of 1	3.283e1	102.6	N/A	N/A
80.00	1 of 1	8.003e1	100.0	N/A	N/A
200.00	1 of 1	1.987e2	99.4	N/A	N/A

Analyte Name: LM-flavones-61_1

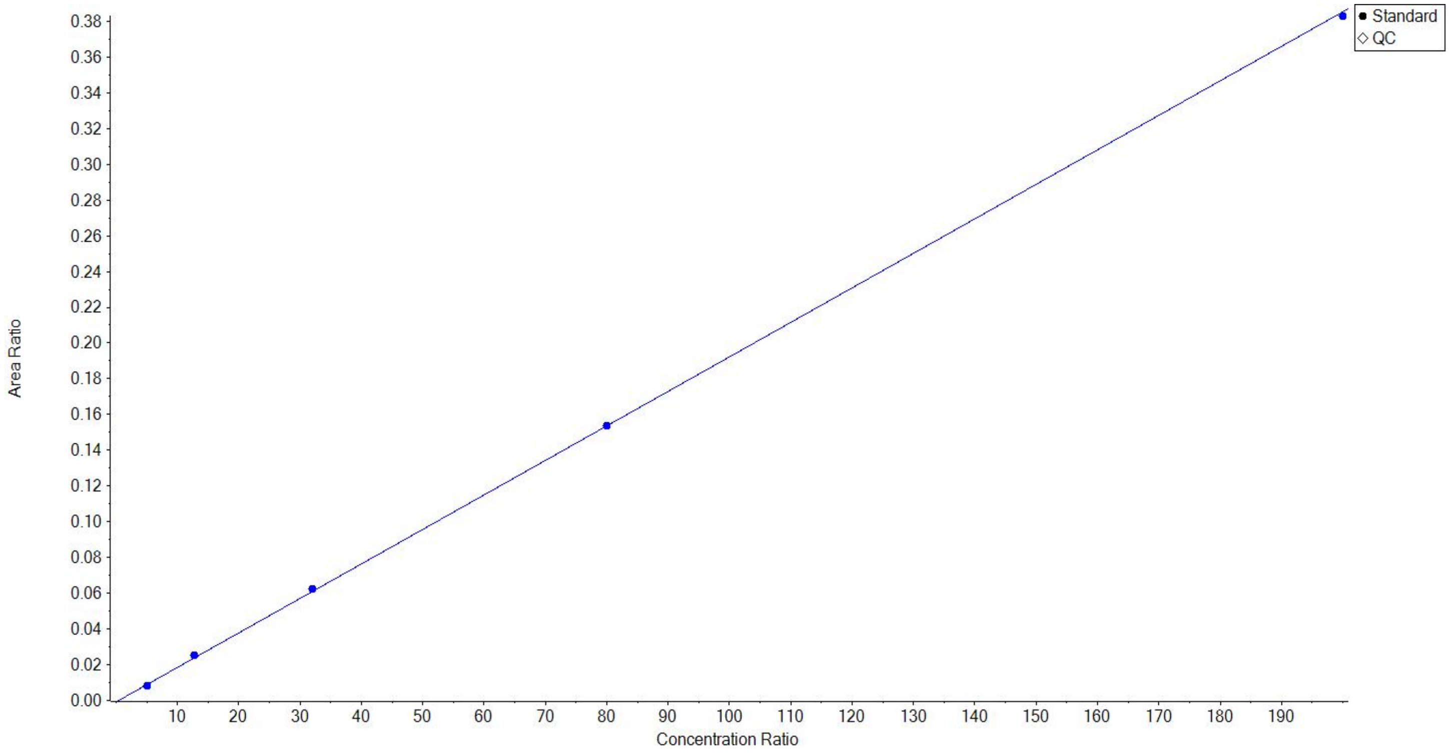
Standard
QC
Area Ratio
Concentration Ratio
0.00
0.02
0.04
0.06
0.08
0.10
0.12
0.14
0.16
0.18
0.20
0.22
0.24
0.26
0.28
0.30
0.32
0.34
0.36
0.38
10
20
30
40
50
60
70
80
90
100
110
120
130
140
150
160
170
180
190

Analyte Name: LM-flavones-06_1
Internal Standard: LM-flavones-IS02_1

**Data File**	flavones-STD-20230908.wiff	**Result Table**	DZLM2023082419-results-20230913-5500
**Acquisition Date**	9/8/2023 5:37:08 PM	**Algorithm Used**	MQ4
**Acquisition Method**	20230908-flavones-(mix130-T3)-15min.dam	**Instrument Name**	QTRAP 6500+ Low Mass
**Project**	N/A	**Processing Method**	20230412-flavones-(mix130-T3)-15min.qmethod

Regression Equation: y = 0.00170 x + 1.10928e-5 (r = 0.99896, r^2 = 0.99791) (weighting: 1 / x)

Expected Concentration	Number of Values	Mean Calculated Concentration (No data for Analyte Unit)	% Accuracy	Std. Deviation	%CV
0.01	0 of 1	N/A	N/A	N/A	N/A
0.02	0 of 1	N/A	N/A	N/A	N/A
0.05	0 of 1	N/A	N/A	N/A	N/A
0.13	0 of 1	N/A	N/A	N/A	N/A
0.33	0 of 1	N/A	N/A	N/A	N/A
0.82	0 of 1	N/A	N/A	N/A	N/A
2.05	1 of 1	2.237e0	109.1	N/A	N/A
5.12	1 of 1	5.089e0	99.4	N/A	N/A
12.80	1 of 1	1.208e1	94.4	N/A	N/A
32.00	1 of 1	3.237e1	101.2	N/A	N/A
80.00	1 of 1	7.451e1	93.1	N/A	N/A
200.00	1 of 1	2.057e2	102.8	N/A	N/A

Analyte Name: LM-flavones-06_1

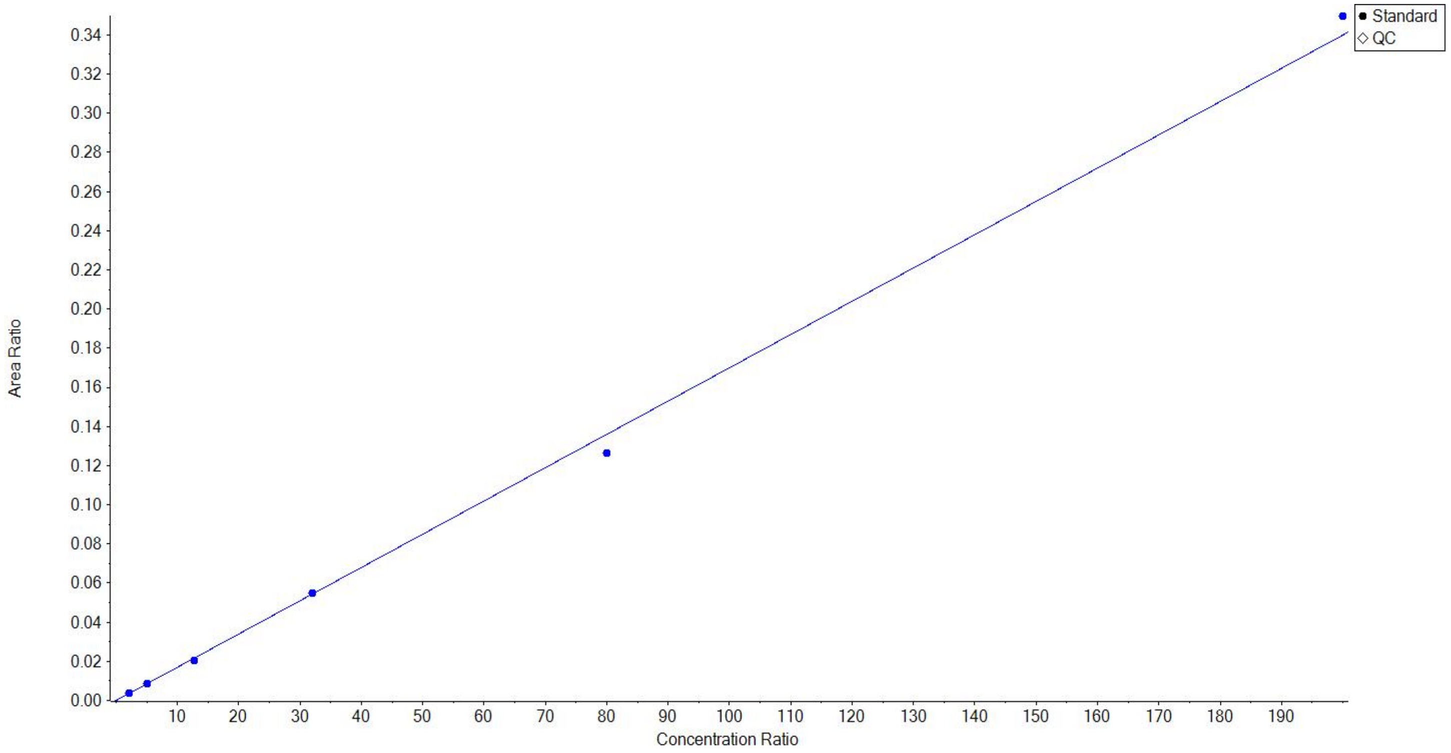
Standard
QC
Area Ratio
0.34
0.32
0.30
0.28
0.26
0.24
0.22
0.20
0.18
0.16
0.14
0.12
0.10
0.08
0.06
0.04
0.02
0.00
10
20
30
40
50
60
70
80
90
100
110
120
130
140
150
160
170
180
190
Concentration Ratio

Analyte Name: LM-flavones-65_1
Internal Standard: LM-flavones-IS02_1

**Data File**	flavones-STD-20230908.wiff	**Result Table**	DZLM2023082419-results-20230913-5500
**Acquisition Date**	9/8/2023 5:37:08 PM	**Algorithm Used**	MQ4
**Acquisition Method**	20230908-flavones-(mix130-T3)-15min.dam	**Instrument Name**	QTRAP 6500+ Low Mass
**Project**	N/A	**Processing Method**	20230412-flavones-(mix130-T3)-15min.qmethod

Regression Equation: y = 0.00191 x + -0.00106 (r = 0.99981, r² = 0.99962) (weighting: 1 / x)

Expected Concentration	Number of Values	Mean Calculated Concentration (No data for Analyte Unit)	% Accuracy	Std. Deviation	%CV
0.01	0 of 1	N/A	N/A	N/A	N/A
0.02	0 of 1	N/A	N/A	N/A	N/A
0.05	0 of 1	N/A	N/A	N/A	N/A
0.13	0 of 1	N/A	N/A	N/A	N/A
0.33	0 of 1	N/A	N/A	N/A	N/A
0.82	0 of 1	N/A	N/A	N/A	N/A
2.05	1 of 1	2.124e0	103.6	N/A	N/A
5.12	1 of 1	4.902e0	95.8	N/A	N/A
12.80	1 of 1	1.297e1	101.3	N/A	N/A
32.00	1 of 1	3.242e1	101.3	N/A	N/A
80.00	1 of 1	7.763e1	97.0	N/A	N/A
200.00	1 of 1	2.019e2	101.0	N/A	N/A

Analyte Name: LM-flavones-65_1

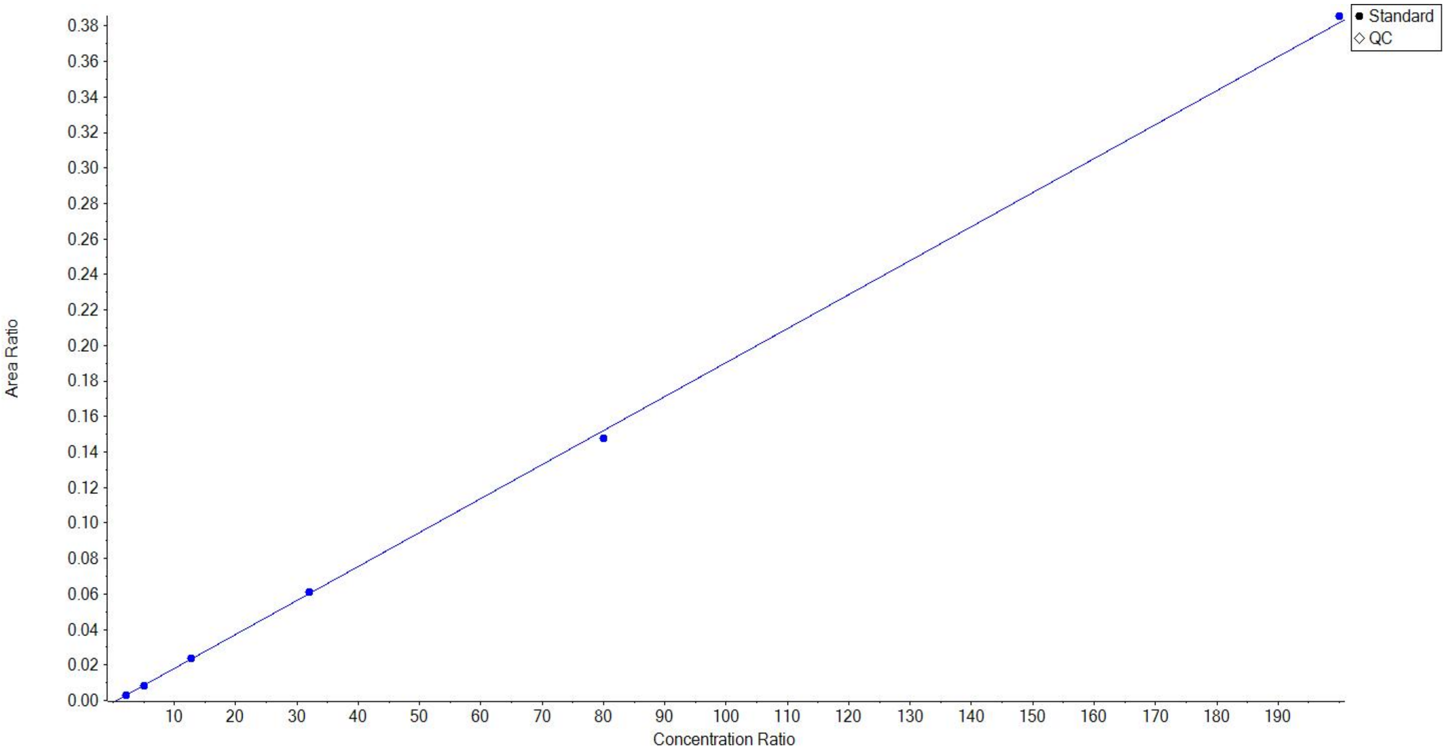
Standard
QC
Area Ratio
Concentration Ratio
0.00
0.02
0.04
0.06
0.08
0.10
0.12
0.14
0.16
0.18
0.20
0.22
0.24
0.26
0.28
0.30
0.32
0.34
0.36
0.38
10
20
30
40
50
60
70
80
90
100
110
120
130
140
150
160
170
180
190

Analyte Name: LM-flavones-68_1
Internal Standard: LM-flavones-IS02_1

**Data File**	flavones-STD-20230908.wiff	**Result Table**	DZLM2023082419-results-20230913-5500
**Acquisition Date**	9/8/2023 5:37:08 PM	**Algorithm Used**	MQ4
**Acquisition Method**	20230908-flavones-(mix130-T3)-15min.dam	**Instrument Name**	QTRAP 6500+ Low Mass
**Project**	N/A	**Processing Method**	20230412-flavones-(mix130-T3)-15min.qmethod

Regression Equation: y = 0.00971 x + -0.00285 (r = 0.99981, r² = 0.99962) (weighting: 1 / x)

Expected Concentration	Number of Values	Mean Calculated Concentration (No data for Analyte Unit)	% Accuracy	Std. Deviation	%CV
0.01	0 of 1	N/A	N/A	N/A	N/A
0.02	0 of 1	N/A	N/A	N/A	N/A
0.05	0 of 1	N/A	N/A	N/A	N/A
0.13	0 of 1	N/A	N/A	N/A	N/A
0.33	0 of 1	N/A	N/A	N/A	N/A
0.82	0 of 1	N/A	N/A	N/A	N/A
2.05	1 of 1	2.137e0	104.3	N/A	N/A
5.12	1 of 1	5.196e0	101.5	N/A	N/A
12.80	1 of 1	1.211e1	94.6	N/A	N/A
32.00	1 of 1	3.231e1	101.0	N/A	N/A
80.00	1 of 1	7.814e1	97.7	N/A	N/A
200.00	1 of 1	2.021e2	101.0	N/A	N/A

Analyte Name: LM-flavones-68_1

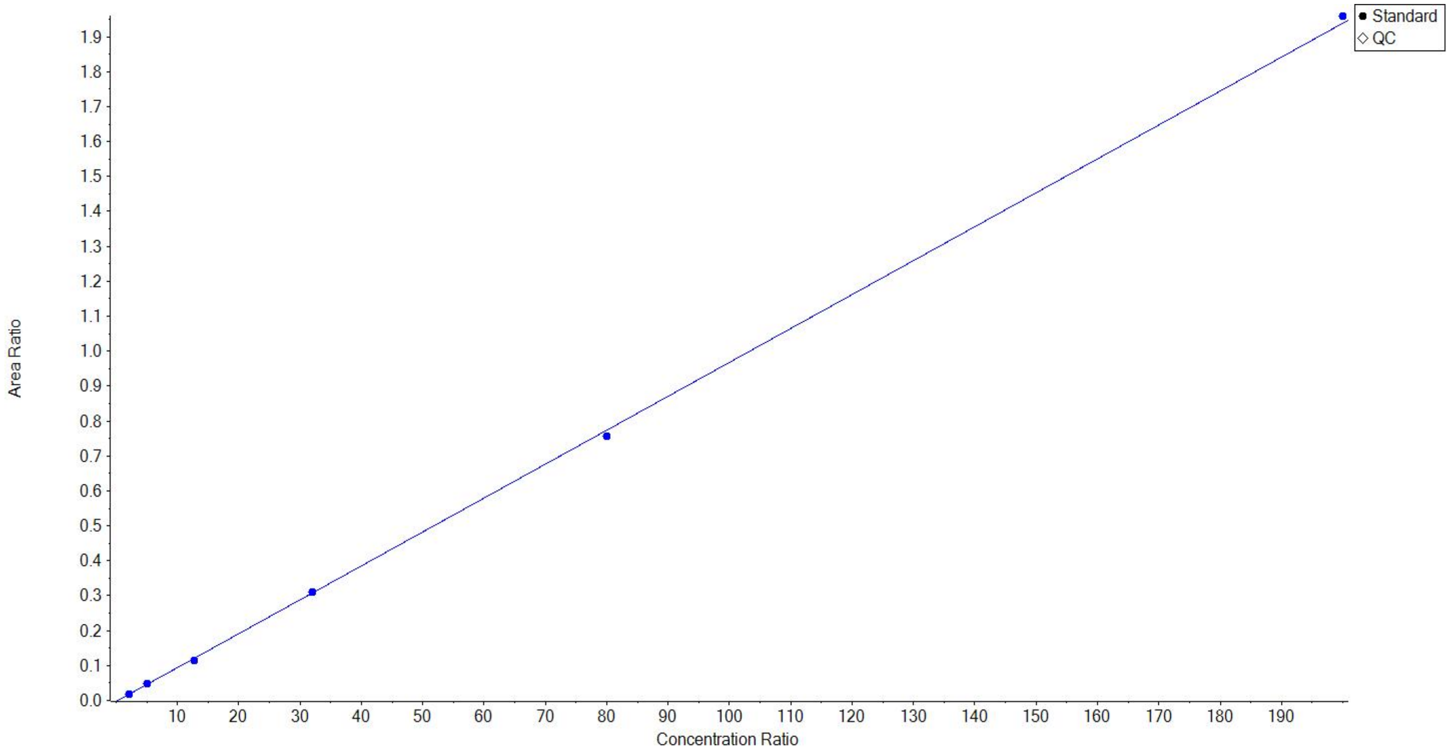
Standard
QC
Area Ratio
Concentration Ratio
0.0
0.1
0.2
0.3
0.4
0.5
0.6
0.7
0.8
0.9
1.0
1.1
1.2
1.3
1.4
1.5
1.6
1.7
1.8
1.9
10
20
30
40
50
60
70
80
90
100
110
120
130
140
150
160
170
180
190

Analyte Name: LM-flavones-80_1
Internal Standard: LM-flavones-IS02_1

**Data File**	flavones-STD-20230908.wiff	**Result Table**	DZLM2023082419-results-20230913-5500
**Acquisition Date**	9/8/2023 5:37:08 PM	**Algorithm Used**	MQ4
**Acquisition Method**	20230908-flavones-(mix130-T3)-15min.dam	**Instrument Name**	QTRAP 6500+ Low Mass
**Project**	N/A	**Processing Method**	20230412-flavones-(mix130-T3)-15min.qmethod

Regression Equation: y = 0.00315 x + -8.95684e-4 (r = 0.99934, r² = 0.99868) (weighting: 1 / x)

Expected Concentration	Number of Values	Mean Calculated Concentration (No data for Analyte Unit)	% Accuracy	Std. Deviation	%CV
0.01	0 of 1	N/A	N/A	N/A	N/A
0.02	0 of 1	N/A	N/A	N/A	N/A
0.05	0 of 1	N/A	N/A	N/A	N/A
0.13	0 of 1	N/A	N/A	N/A	N/A
0.33	0 of 1	N/A	N/A	N/A	N/A
0.82	0 of 1	N/A	N/A	N/A	N/A
2.05	1 of 1	2.272e0	110.8	N/A	N/A
5.12	1 of 1	4.992e0	97.5	N/A	N/A
12.80	1 of 1	1.221e1	95.4	N/A	N/A
32.00	1 of 1	3.164e1	98.9	N/A	N/A
80.00	1 of 1	7.596e1	95.0	N/A	N/A
200.00	1 of 1	2.049e2	102.4	N/A	N/A

Analyte Name: LM-flavones-80_1

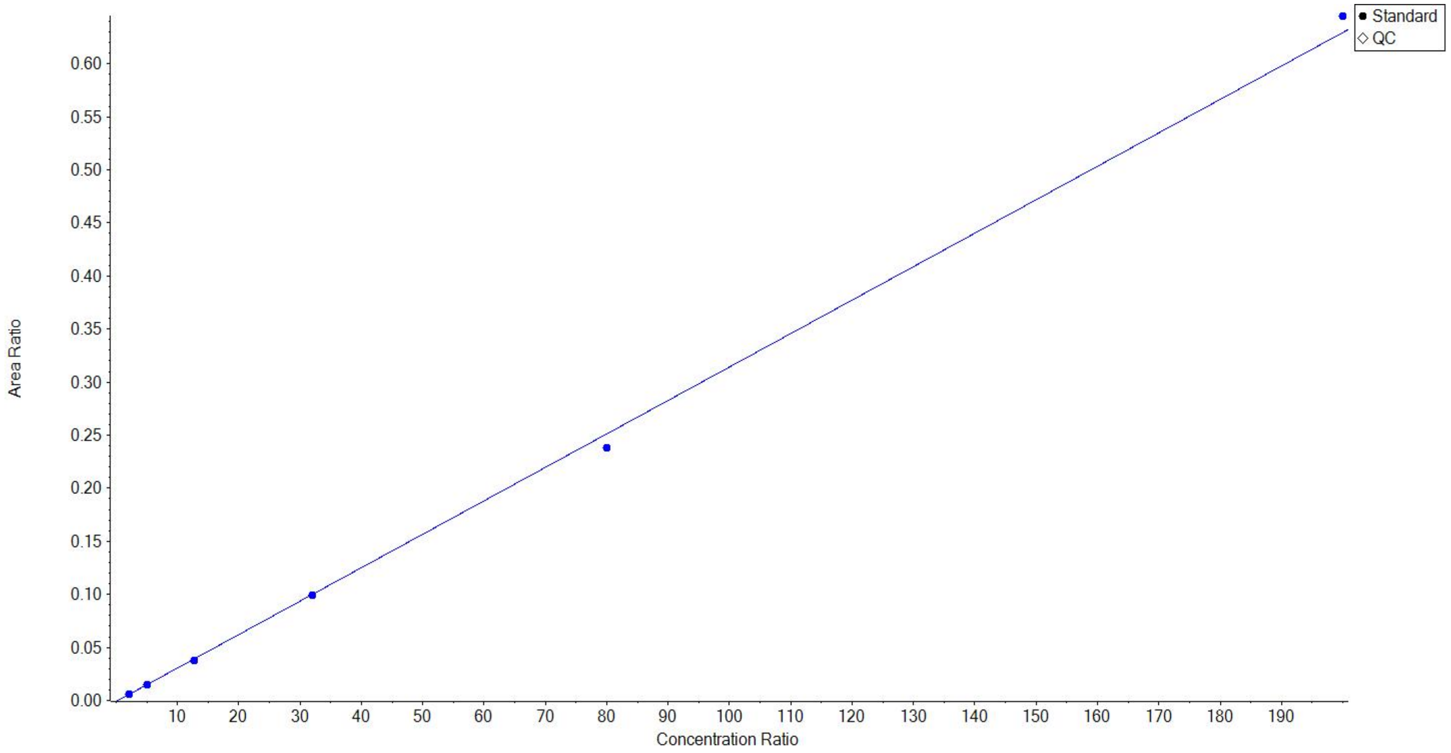
Standard
QC
0.60
0.55
0.50
0.45
0.40
0.35
0.30
0.25
0.20
0.15
0.10
0.05
0.00
Area Ratio
10
20
30
40
50
60
70
80
90
100
110
120
130
140
150
160
170
180
190
Concentration Ratio

Analyte Name: LM-flavones-85_1
Internal Standard: LM-flavones-IS02_1

**Data File**	flavones-STD-20230908.wiff	**Result Table**	DZLM2023082419-results-20230913-5500
**Acquisition Date**	9/8/2023 5:37:08 PM	**Algorithm Used**	MQ4
**Acquisition Method**	20230908-flavones-(mix130-T3)-15min.dam	**Instrument Name**	QTRAP 6500+ Low Mass
**Project**	N/A	**Processing Method**	20230412-flavones-(mix130-T3)-15min.qmethod

Regression Equation: $y = 0.00759 x + 0.00236$ ($r = 0.99974$, $r^2 = 0.99947$) (weighting: 1 / x)

Expected Concentration	Number of Values	Mean Calculated Concentration (No data for Analyte Unit)	% Accuracy	Std. Deviation	%CV
0.01	0 of 1	N/A	N/A	N/A	N/A
0.02	0 of 1	N/A	N/A	N/A	N/A
0.05	0 of 1	N/A	N/A	N/A	N/A
0.13	0 of 1	N/A	N/A	N/A	N/A
0.33	0 of 1	N/A	N/A	N/A	N/A
0.82	0 of 1	N/A	N/A	N/A	N/A
2.05	1 of 1	2.172e0	105.9	N/A	N/A
5.12	1 of 1	4.789e0	93.5	N/A	N/A
12.80	1 of 1	1.228e1	96.0	N/A	N/A
32.00	1 of 1	3.376e1	105.5	N/A	N/A
80.00	1 of 1	7.944e1	99.3	N/A	N/A
200.00	1 of 1	1.995e2	99.8	N/A	N/A

Analyte Name: LM-flavones-85_1

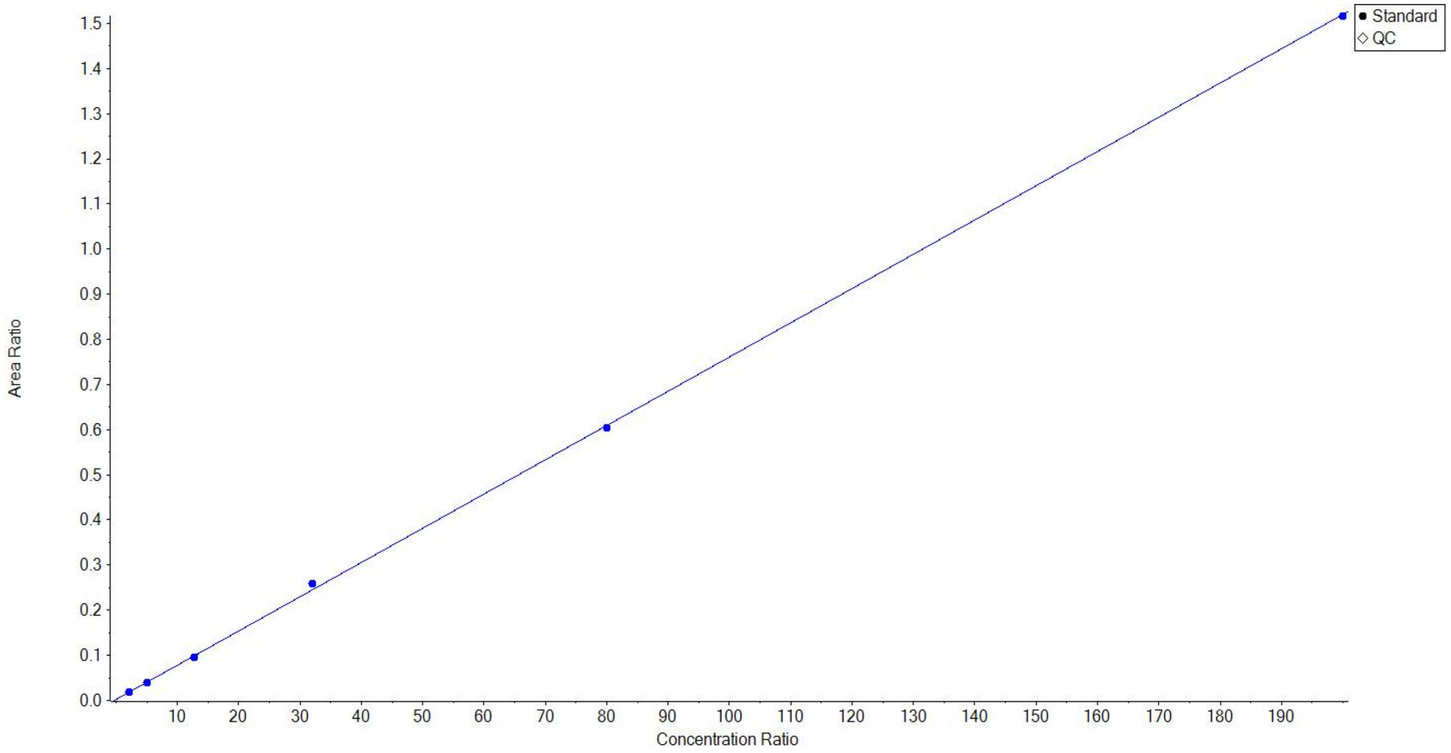
Area Ratio
1.5
1.4
1.3
1.2
1.1
1.0
0.9
0.8
0.7
0.6
0.5
0.4
0.3
0.2
0.1
0.0
10
20
30
40
50
60
70
80
90
100
110
120
130
140
150
160
170
180
190
Concentration Ratio
Standard
QC

Created with SCIEX OS 1.6.1
Printed:9/18/2023 5:27:01 PM

Analyte Name: LM-flavones-91_1
Internal Standard: LM-flavones-IS02_1

**Data File**	flavones-STD-20230908.wiff	**Result Table**	DZLM2023082419-results-20230913-5500
**Acquisition Date**	9/8/2023 5:37:08 PM	**Algorithm Used**	MQ4
**Acquisition Method**	20230908-flavones-(mix130-T3)-15min.dam	**Instrument Name**	QTRAP 6500+ Low Mass
**Project**	N/A	**Processing Method**	20230412-flavones-(mix130-T3)-15min.qmethod

Regression Equation: y = 0.10938 x + -0.00212 (r = 0.99991, r² = 0.99982) (weighting: 1 / x)

Expected Concentration	Number of Values	Mean Calculated Concentration (No data for Analyte Unit)	% Accuracy	Std. Deviation	%CV
0.01	0 of 1	N/A	N/A	N/A	N/A
0.02	0 of 1	N/A	N/A	N/A	N/A
0.05	0 of 1	N/A	N/A	N/A	N/A
0.13	0 of 1	N/A	N/A	N/A	N/A
0.33	0 of 1	N/A	N/A	N/A	N/A
0.82	0 of 1	N/A	N/A	N/A	N/A
2.05	1 of 1	2.149e0	104.8	N/A	N/A
5.12	1 of 1	5.031e0	98.3	N/A	N/A
12.80	1 of 1	1.227e1	95.8	N/A	N/A
32.00	1 of 1	3.256e1	101.7	N/A	N/A
80.00	1 of 1	7.914e1	98.9	N/A	N/A
200.00	1 of 1	2.008e2	100.4	N/A	N/A

Analyte Name: LM-flavones-91_1

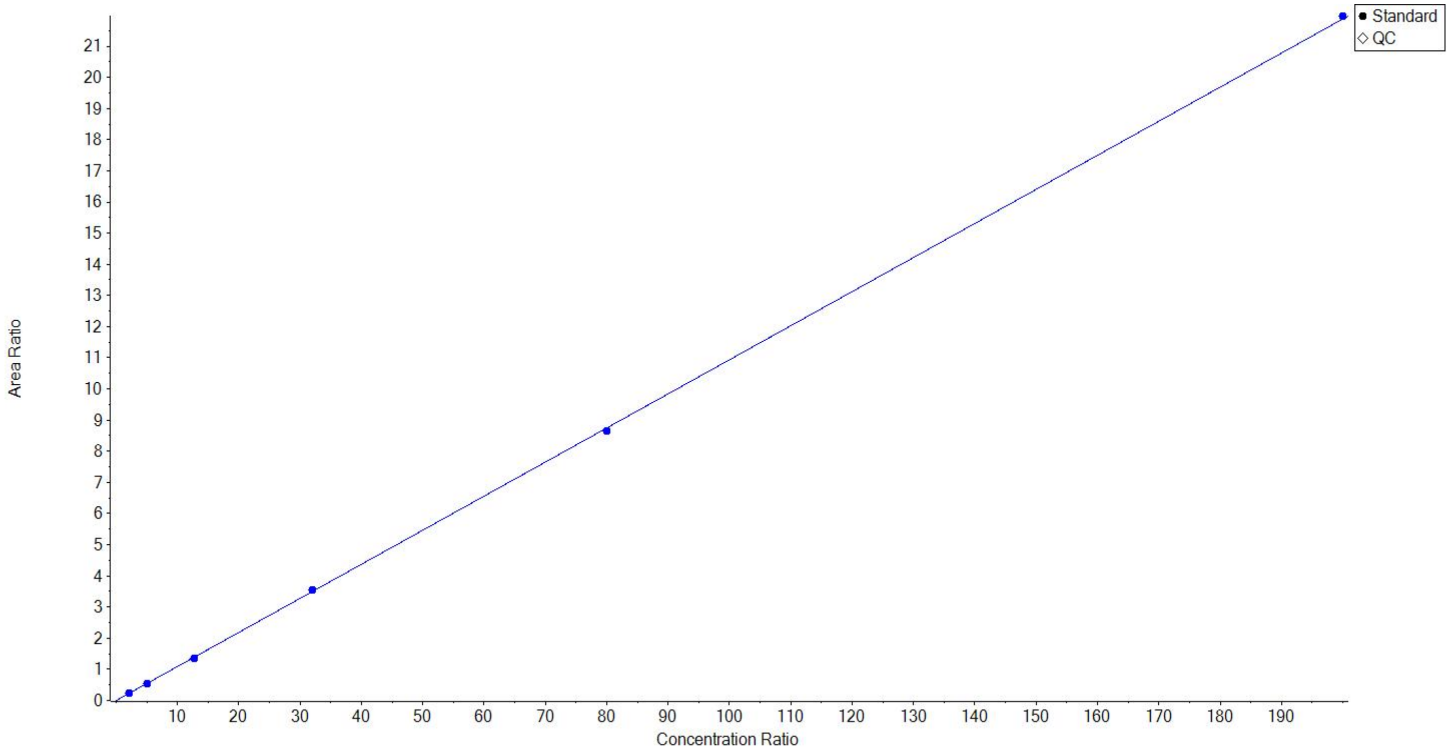
Standard
QC
Area Ratio
Concentration Ratio

Analyte Name: LM-flavones-92
Internal Standard: LM-flavones-IS02_1

**Data File**	flavones-STD-20230908.wiff	**Result Table**	DZLM2023082419-results-20230913-5500
**Acquisition Date**	9/8/2023 5:37:08 PM	**Algorithm Used**	MQ4
**Acquisition Method**	20230908-flavones-(mix130-T3)-15min.dam	**Instrument Name**	QTRAP 6500+ Low Mass
**Project**	N/A	**Processing Method**	20230412-flavones-(mix130-T3)-15min.qmethod

Regression Equation: y = 0.02768 x + 0.00162 (r = 0.99991, r² = 0.99983) (weighting: 1 / x)

Expected Concentration	Number of Values	Mean Calculated Concentration (No data for Analyte Unit)	% Accuracy	Std. Deviation	%CV
0.01	0 of 1	N/A	N/A	N/A	N/A
0.02	0 of 1	N/A	N/A	N/A	N/A
0.05	0 of 1	N/A	N/A	N/A	N/A
0.13	0 of 1	N/A	N/A	N/A	N/A
0.33	0 of 1	N/A	N/A	N/A	N/A
0.82	0 of 1	N/A	N/A	N/A	N/A
2.05	1 of 1	2.074e0	101.2	N/A	N/A
5.12	1 of 1	5.141e0	100.4	N/A	N/A
12.80	1 of 1	1.270e1	99.2	N/A	N/A
32.00	1 of 1	3.217e1	100.5	N/A	N/A
80.00	1 of 1	7.833e1	97.9	N/A	N/A
200.00	1 of 1	2.016e2	100.8	N/A	N/A

Analyte Name: LM-flavones-92

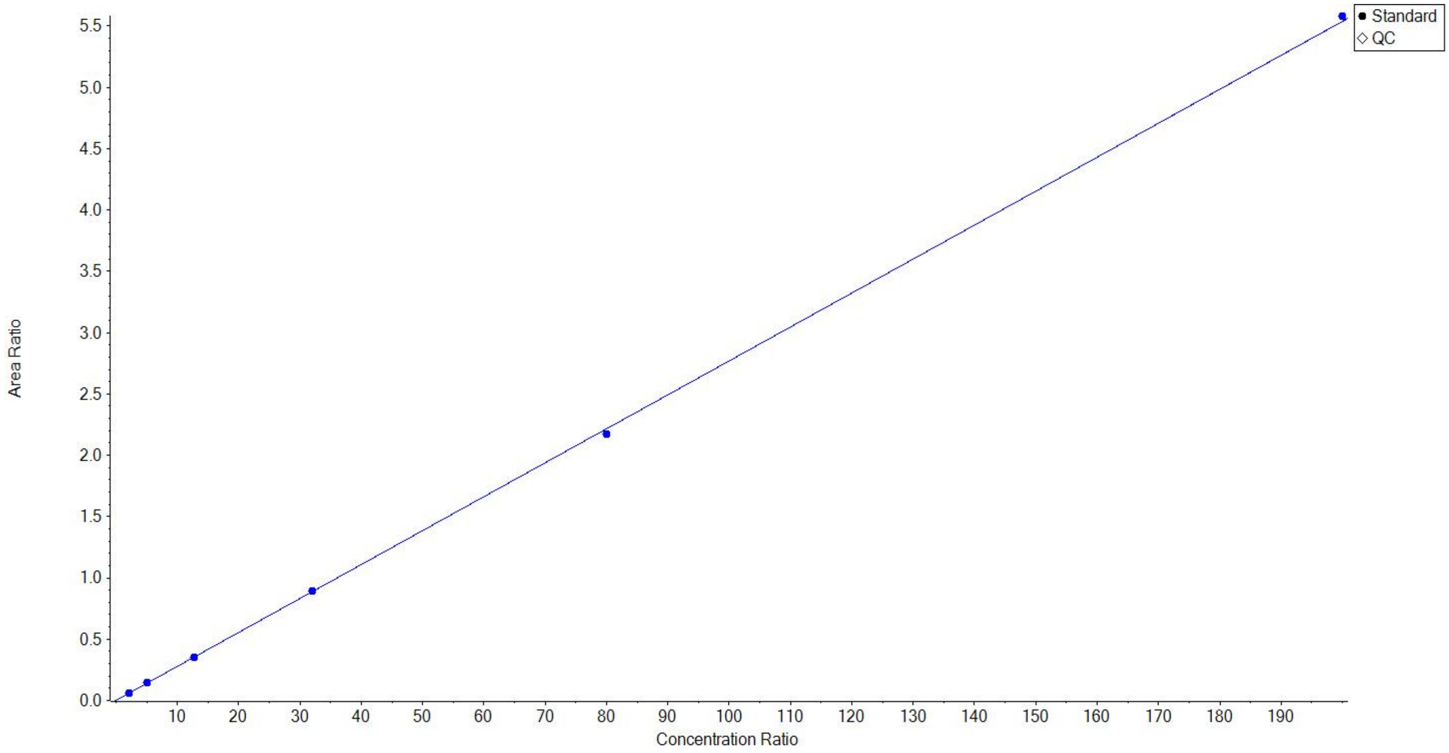
Standard
QC
Area Ratio
0.0
0.5
1.0
1.5
2.0
2.5
3.0
3.5
4.0
4.5
5.0
5.5
10
20
30
40
50
60
70
80
90
100
110
120
130
140
150
160
170
180
190
Concentration Ratio

Analyte Name: LM-flavones-95
Internal Standard: LM-flavones-IS02_1

**Data File**	flavones-STD-20230908.wiff	**Result Table**	DZLM2023082419-results-20230913-5500
**Acquisition Date**	9/8/2023 5:37:08 PM	**Algorithm Used**	MQ4
**Acquisition Method**	20230908-flavones-(mix130-T3)-15min.dam	**Instrument Name**	QTRAP 6500+ Low Mass
**Project**	N/A	**Processing Method**	20230412-flavones-(mix130-T3)-15min.qmethod

Regression Equation: y = 0.01340 x + 0.00820 (r = 0.99937, r² = 0.99874) (weighting: 1 / x)

Expected Concentration	Number of Values	Mean Calculated Concentration (No data for Analyte Unit)	% Accuracy	Std. Deviation	%CV
0.01	0 of 1	N/A	N/A	N/A	N/A
0.02	0 of 1	N/A	N/A	N/A	N/A
0.05	0 of 1	N/A	N/A	N/A	N/A
0.13	0 of 1	N/A	N/A	N/A	N/A
0.33	0 of 1	N/A	N/A	N/A	N/A
0.82	0 of 1	N/A	N/A	N/A	N/A
2.05	1 of 1	1.882e0	91.9	N/A	N/A
5.12	1 of 1	4.971e0	97.1	N/A	N/A
12.80	1 of 1	1.319e1	103.0	N/A	N/A
32.00	1 of 1	3.498e1	109.3	N/A	N/A
80.00	1 of 1	8.026e1	100.3	N/A	N/A
200.00	1 of 1	1.967e2	98.3	N/A	N/A

Analyte Name: LM-flavones-95

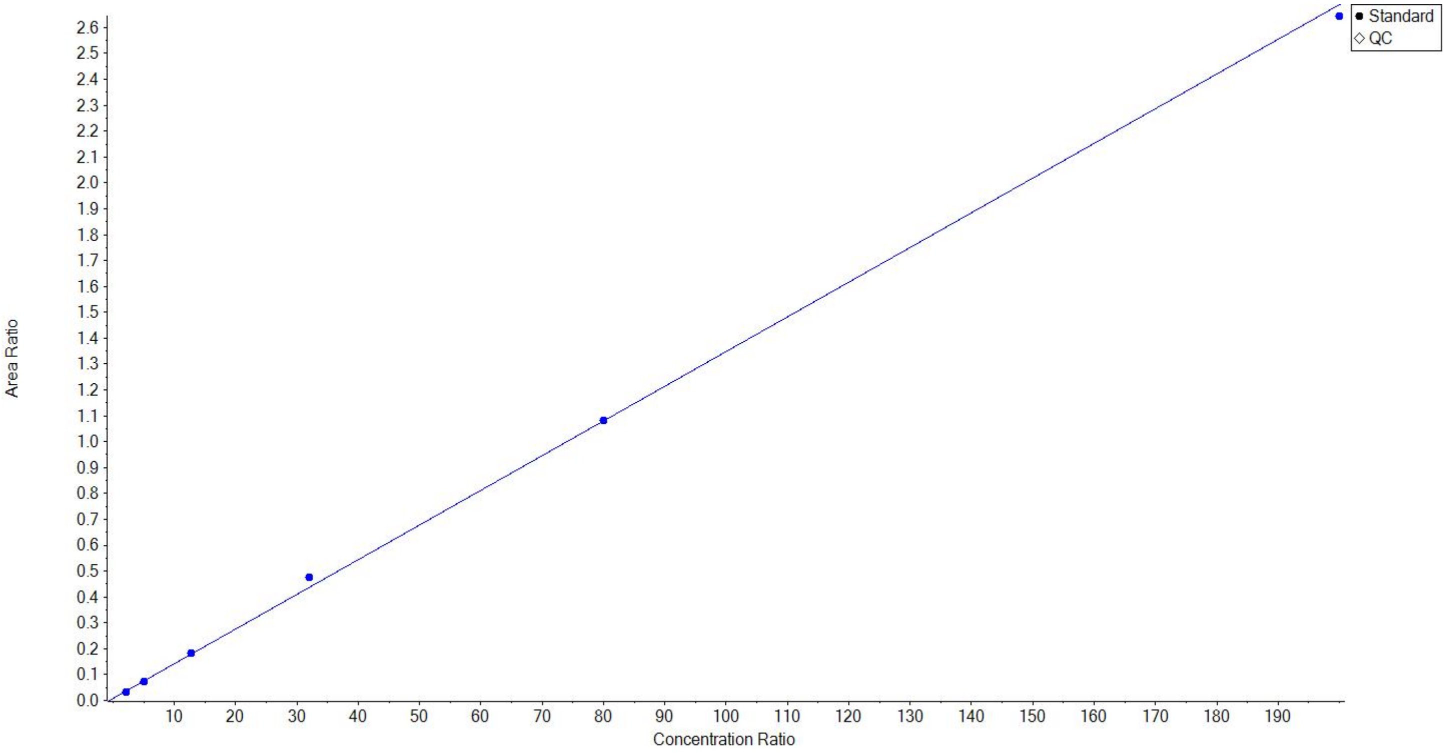
Standard
QC
Area Ratio
Concentration Ratio
0.0
0.1
0.2
0.3
0.4
0.5
0.6
0.7
0.8
0.9
1.0
1.1
1.2
1.3
1.4
1.5
1.6
1.7
1.8
1.9
2.0
2.1
2.2
2.3
2.4
2.5
2.6
10
20
30
40
50
60
70
80
90
100
110
120
130
140
150
160
170
180
190

Analyte Name: LM-flavones-105_1
Internal Standard: LM-flavones-IS02_1

**Data File**	flavones-STD-20230908.wiff	**Result Table**	DZLM2023082419-results-20230913-5500
**Acquisition Date**	9/8/2023 5:37:08 PM	**Algorithm Used**	MQ4
**Acquisition Method**	20230908-flavones-(mix130-T3)-15min.dam	**Instrument Name**	QTRAP 6500+ Low Mass
**Project**	N/A	**Processing Method**	20230412-flavones-(mix130-T3)-15min.qmethod

Regression Equation: y = 0.02621 x + -0.00718 (r = 0.99973, r² = 0.99945) (weighting: 1 / x)

Expected Concentration	Number of Values	Mean Calculated Concentration (No data for Analyte Unit)	% Accuracy	Std. Deviation	%CV
0.01	0 of 1	N/A	N/A	N/A	N/A
0.02	0 of 1	N/A	N/A	N/A	N/A
0.05	0 of 1	N/A	N/A	N/A	N/A
0.13	0 of 1	N/A	N/A	N/A	N/A
0.33	0 of 1	N/A	N/A	N/A	N/A
0.82	0 of 1	N/A	N/A	N/A	N/A
2.05	1 of 1	2.238e0	109.3	N/A	N/A
5.12	1 of 1	5.028e0	98.2	N/A	N/A
12.80	1 of 1	1.220e1	95.3	N/A	N/A
32.00	1 of 1	3.141e1	98.2	N/A	N/A
80.00	1 of 1	7.797e1	97.5	N/A	N/A
200.00	1 of 1	2.031e2	101.6	N/A	N/A

Analyte Name: LM-flavones-105_1

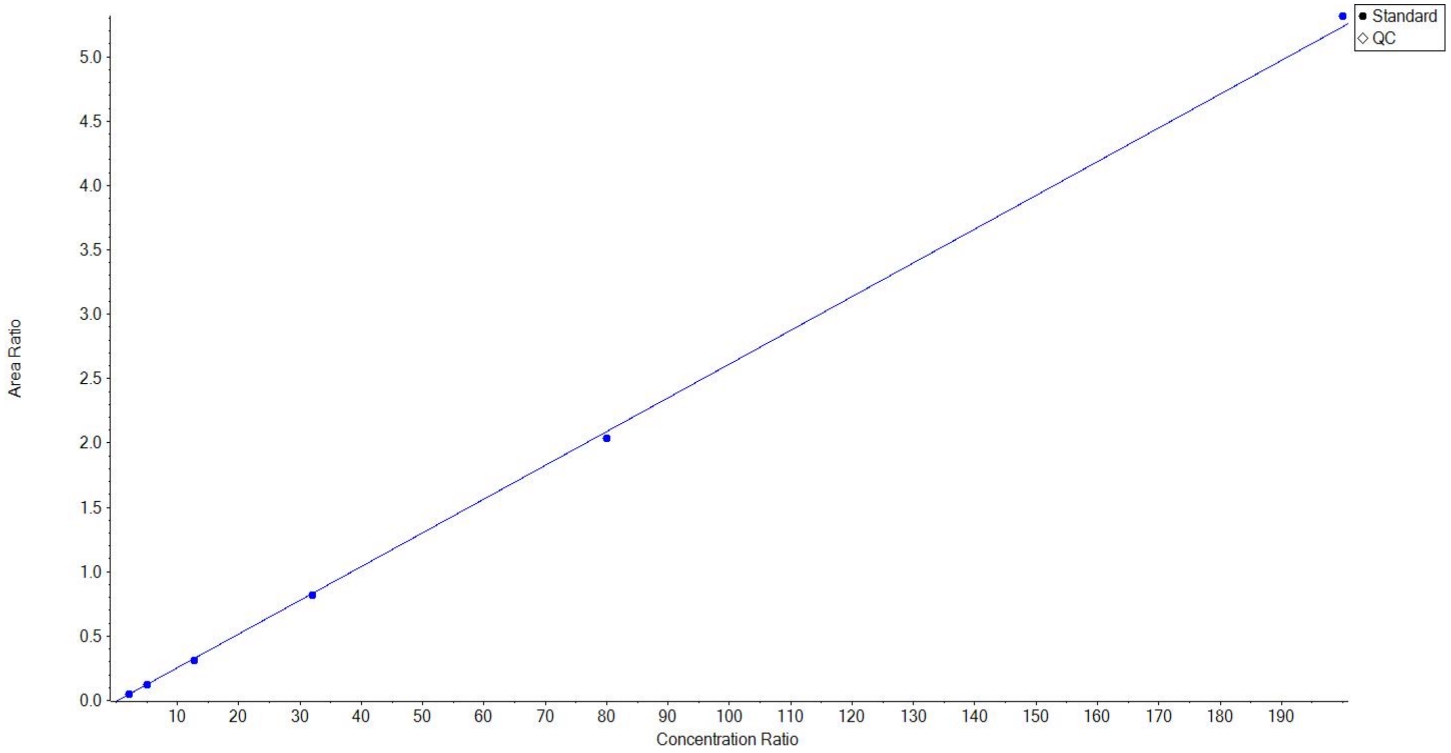
Standard
QC
Area Ratio
Concentration Ratio
0.0
0.5
1.0
1.5
2.0
2.5
3.0
3.5
4.0
4.5
5.0
10
20
30
40
50
60
70
80
90
100
110
120
130
140
150
160
170
180
190

Analyte Name: LM-flavones-117_1
Internal Standard: LM-flavones-IS02_1

**Data File**	flavones-STD-20230908.wiff	**Result Table**	DZLM2023082419-results-20230913-5500
**Acquisition Date**	9/8/2023 5:37:08 PM	**Algorithm Used**	MQ4
**Acquisition Method**	20230908-flavones-(mix130-T3)-15min.dam	**Instrument Name**	QTRAP 6500+ Low Mass
**Project**	N/A	**Processing Method**	20230412-flavones-(mix130-T3)-15min.qmethod

Regression Equation: y = 0.01114 x + 0.00455 (r = 0.99956, r² = 0.99913) (weighting: 1 / x)

Expected Concentration	Number of Values	Mean Calculated Concentration (No data for Analyte Unit)	% Accuracy	Std. Deviation	%CV
0.01	0 of 1	N/A	N/A	N/A	N/A
0.02	0 of 1	N/A	N/A	N/A	N/A
0.05	0 of 1	N/A	N/A	N/A	N/A
0.13	0 of 1	N/A	N/A	N/A	N/A
0.33	0 of 1	N/A	N/A	N/A	N/A
0.82	0 of 1	N/A	N/A	N/A	N/A
2.05	1 of 1	1.917e0	93.6	N/A	N/A
5.12	1 of 1	5.090e0	99.4	N/A	N/A
12.80	1 of 1	1.278e1	99.8	N/A	N/A
32.00	1 of 1	3.441e1	107.5	N/A	N/A
80.00	1 of 1	8.095e1	101.2	N/A	N/A
200.00	1 of 1	1.968e2	98.4	N/A	N/A

Analyte Name: LM-flavones-117_1

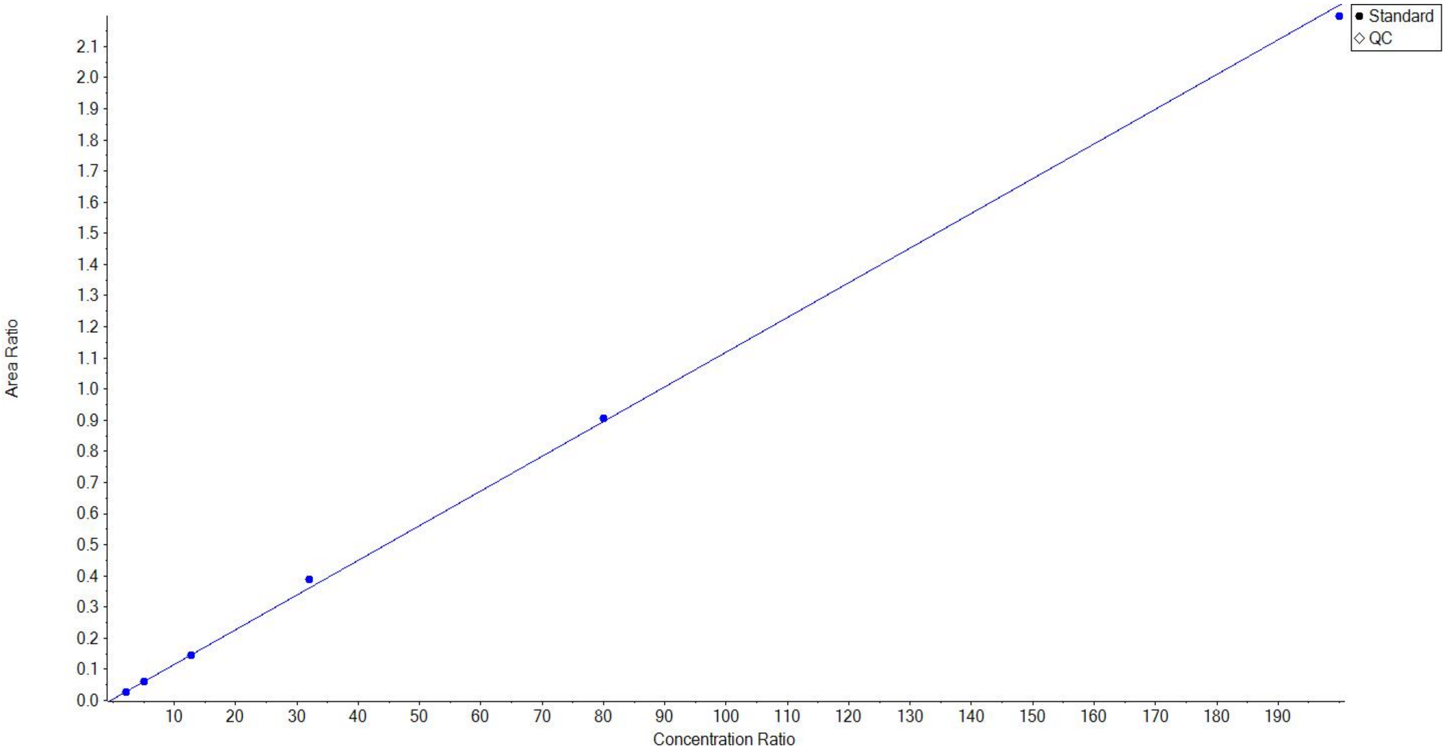
Standard
QC
Area Ratio
Concentration Ratio
0.0
0.1
0.2
0.3
0.4
0.5
0.6
0.7
0.8
0.9
1.0
1.1
1.2
1.3
1.4
1.5
1.6
1.7
1.8
1.9
2.0
2.1
10
20
30
40
50
60
70
80
90
100
110
120
130
140
150
160
170
180
190

Analyte Name: LM-flavones-120_1
Internal Standard: LM-flavones-IS02_1

**Data File**	flavones-STD-20230908.wiff	**Result Table**	DZLM2023082419-results-20230913-5500
**Acquisition Date**	9/8/2023 5:37:08 PM	**Algorithm Used**	MQ4
**Acquisition Method**	20230908-flavones-(mix130-T3)-15min.dam	**Instrument Name**	QTRAP 6500+ Low Mass
**Project**	N/A	**Processing Method**	20230412-flavones-(mix130-T3)-15min.qmethod

Regression Equation: y = 0.00220 x + 1.57314e-4 (r = 0.99964, r^2 = 0.99928) (weighting: 1 / x)

Expected Concentration	Number of Values	Mean Calculated Concentration (No data for Analyte Unit)	% Accuracy	Std. Deviation	%CV
0.01	0 of 1	N/A	N/A	N/A	N/A
0.02	0 of 1	N/A	N/A	N/A	N/A
0.05	0 of 1	N/A	N/A	N/A	N/A
0.13	0 of 1	N/A	N/A	N/A	N/A
0.33	0 of 1	N/A	N/A	N/A	N/A
0.82	0 of 1	N/A	N/A	N/A	N/A
2.05	1 of 1	2.149e0	104.9	N/A	N/A
5.12	1 of 1	4.530e0	88.5	N/A	N/A
12.80	1 of 1	1.296e1	101.3	N/A	N/A
32.00	1 of 1	3.392e1	106.0	N/A	N/A
80.00	1 of 1	8.019e1	100.2	N/A	N/A
200.00	1 of 1	1.982e2	99.1	N/A	N/A

Analyte Name: LM-flavones-120_1

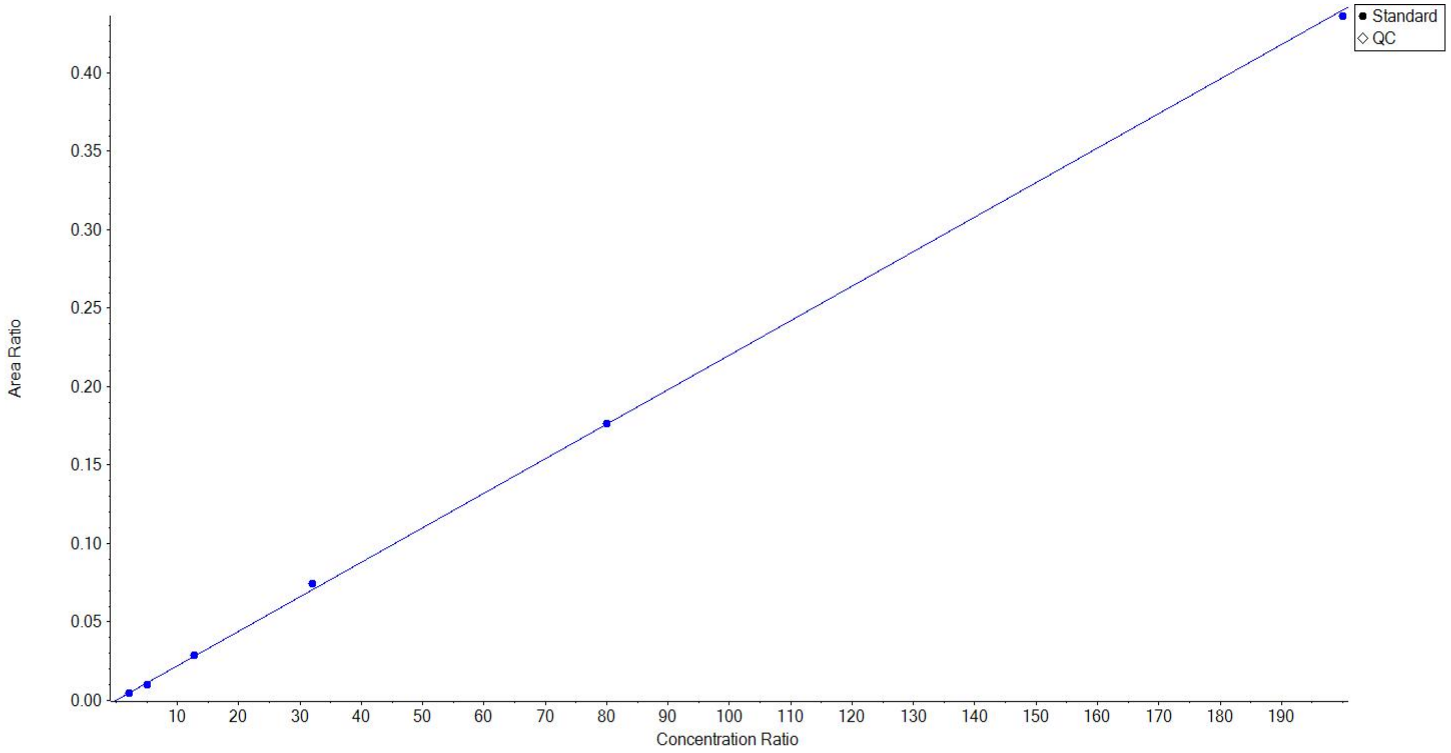
Standard
QC
Area Ratio
0.40
0.35
0.30
0.25
0.20
0.15
0.10
0.05
0.00
10
20
30
40
50
60
70
80
90
100
110
120
130
140
150
160
170
180
190
Concentration Ratio

Analyte Name: LM-flavones-121_1
Internal Standard: LM-flavones-IS02_1

**Data File**	flavones-STD-20230908.wiff	**Result Table**	DZLM2023082419-results-20230913-5500
**Acquisition Date**	9/8/2023 5:37:08 PM	**Algorithm Used**	MQ4
**Acquisition Method**	20230908-flavones-(mix130-T3)-15min.dam	**Instrument Name**	QTRAP 6500+ Low Mass
**Project**	N/A	**Processing Method**	20230412-flavones-(mix130-T3)-15min.qmethod

Regression Equation: y = 0.00193 x + 8.34795e-4 (r = 0.99681, r² = 0.99363) (weighting: 1 / x)

Expected Concentration	Number of Values	Mean Calculated Concentration (No data for Analyte Unit)	% Accuracy	Std. Deviation	%CV
0.01	0 of 1	N/A	N/A	N/A	N/A
0.02	0 of 1	N/A	N/A	N/A	N/A
0.05	0 of 1	N/A	N/A	N/A	N/A
0.13	0 of 1	N/A	N/A	N/A	N/A
0.33	0 of 1	N/A	N/A	N/A	N/A
0.82	0 of 1	N/A	N/A	N/A	N/A
2.05	1 of 1	2.165e0	105.7	N/A	N/A
5.12	1 of 1	4.870e0	95.1	N/A	N/A
12.80	1 of 1	1.279e1	99.9	N/A	N/A
32.00	1 of 1	2.879e1	90.0	N/A	N/A
80.00	1 of 1	9.010e1	112.6	N/A	N/A
200.00	1 of 1	1.933e2	96.6	N/A	N/A

Analyte Name: LM-flavones-121_1

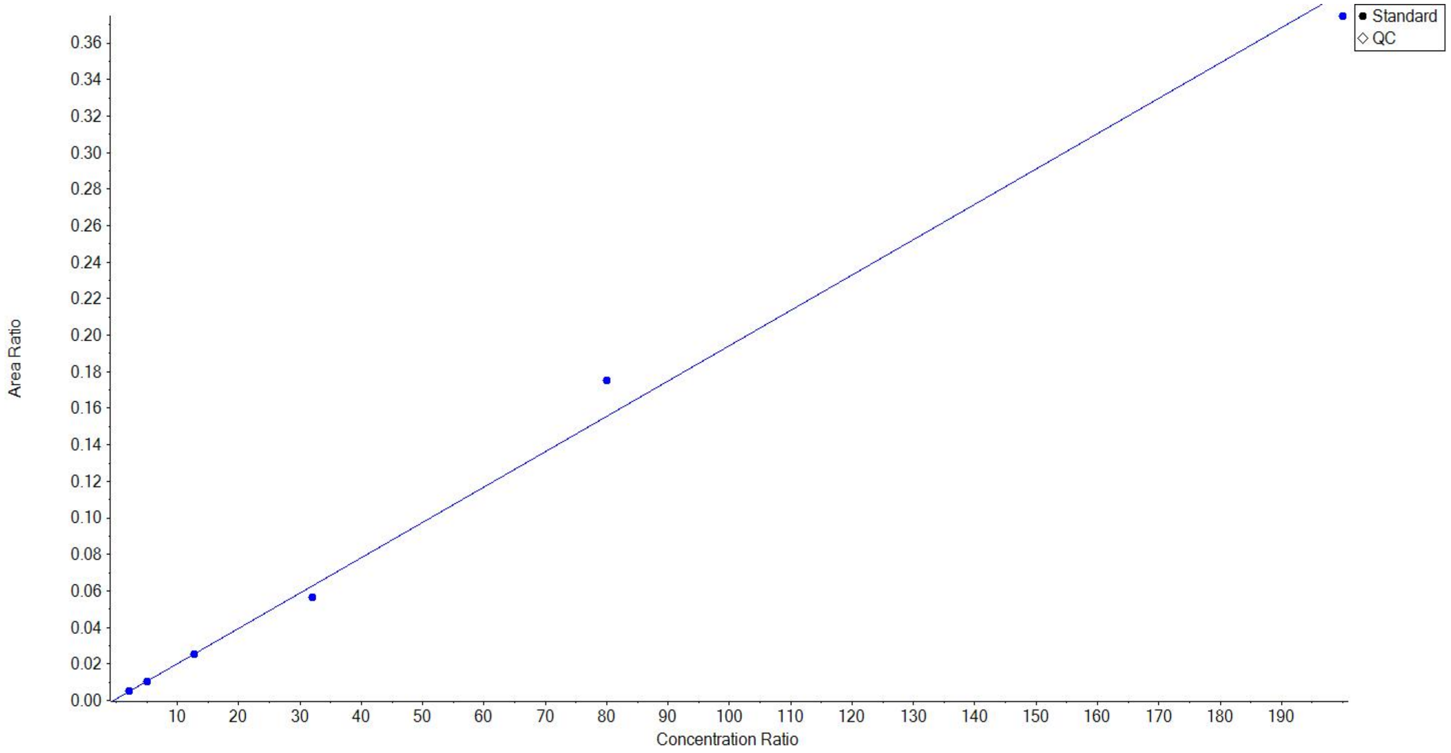
Standard
QC
Area Ratio
0.36
0.34
0.32
0.30
0.28
0.26
0.24
0.22
0.20
0.18
0.16
0.14
0.12
0.10
0.08
0.06
0.04
0.02
0.00
10 20 30 40 50 60 70 80 90 100 110 120 130 140 150 160 170 180 190
Concentration Ratio

Analyte Name: LM-flavones-124
Internal Standard: LM-flavones-IS02_1

**Data File**	flavones-STD-20230908.wiff	**Result Table**	DZLM2023082419-results-20230913-5500
**Acquisition Date**	9/8/2023 5:37:08 PM	**Algorithm Used**	MQ4
**Acquisition Method**	20230908-flavones-(mix130-T3)-15min.dam	**Instrument Name**	QTRAP 6500+ Low Mass
**Project**	N/A	**Processing Method**	20230412-flavones-(mix130-T3)-15min.qmethod

Regression Equation: y = 0.00200 x + -8.10886e-4 (r = 0.99941, r² = 0.99883) (weighting: 1 / x)

Expected Concentration	Number of Values	Mean Calculated Concentration (No data for Analyte Unit)	% Accuracy	Std. Deviation	%CV
0.01	0 of 1	N/A	N/A	N/A	N/A
0.02	0 of 1	N/A	N/A	N/A	N/A
0.05	0 of 1	N/A	N/A	N/A	N/A
0.13	0 of 1	N/A	N/A	N/A	N/A
0.33	0 of 1	N/A	N/A	N/A	N/A
0.82	0 of 1	N/A	N/A	N/A	N/A
2.05	1 of 1	2.261e0	110.4	N/A	N/A
5.12	1 of 1	5.151e0	100.6	N/A	N/A
12.80	1 of 1	1.214e1	94.8	N/A	N/A
32.00	1 of 1	2.970e1	92.8	N/A	N/A
80.00	0 of 1	N/A	N/A	N/A	N/A
200.00	1 of 1	2.027e2	101.4	N/A	N/A

Analyte Name: LM-flavones-124

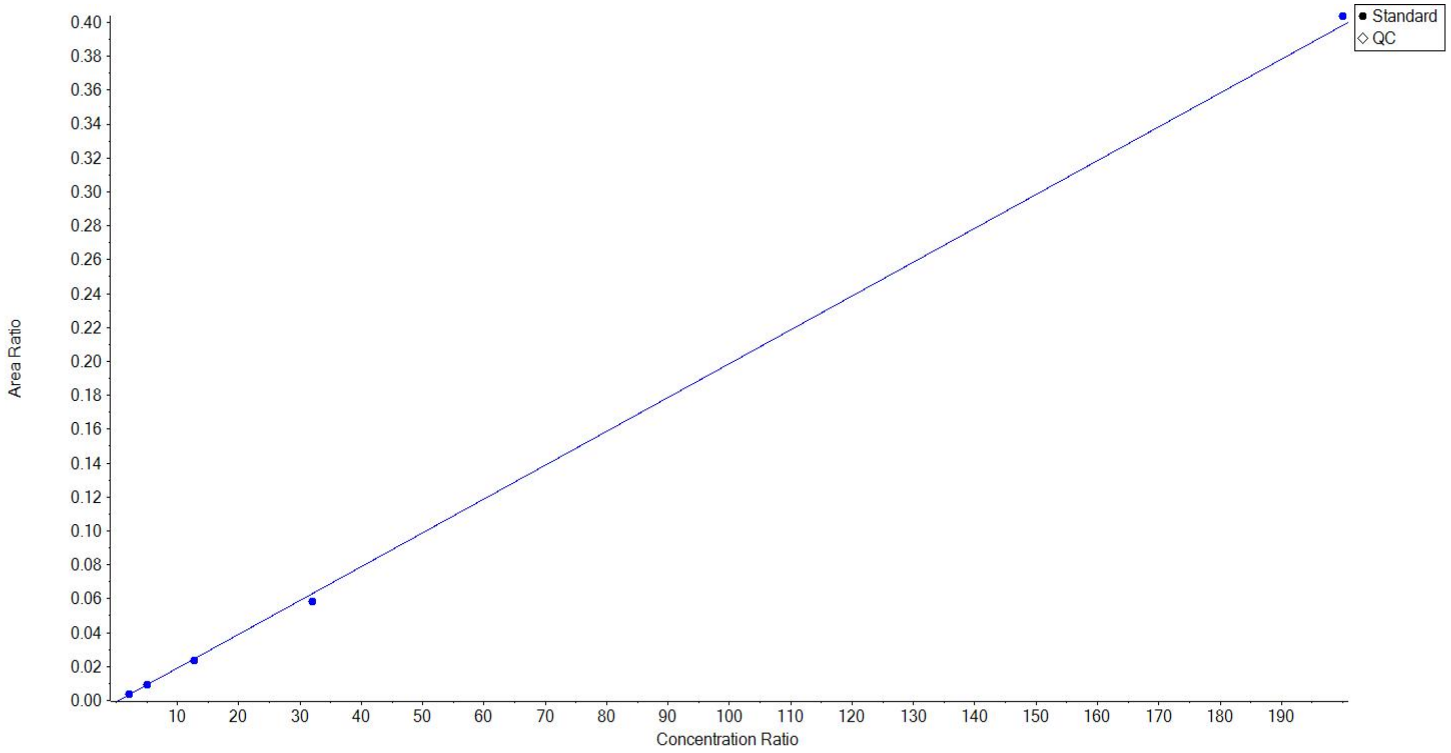
Standard
QC
Area Ratio
0.40
0.38
0.36
0.34
0.32
0.30
0.28
0.26
0.24
0.22
0.20
0.18
0.16
0.14
0.12
0.10
0.08
0.06
0.04
0.02
0.00
10
20
30
40
50
60
70
80
90
100
110
120
130
140
150
160
170
180
190
Concentration Ratio

Analyte Name: LM-flavones-127_1
Internal Standard: LM-flavones-IS02_1

**Data File**	flavones-STD-20230908.wiff	**Result Table**	DZLM2023082419-results-20230913-5500
**Acquisition Date**	9/8/2023 5:37:08 PM	**Algorithm Used**	MQ4
**Acquisition Method**	20230908-flavones-(mix130-T3)-15min.dam	**Instrument Name**	QTRAP 6500+ Low Mass
**Project**	N/A	**Processing Method**	20230412-flavones-(mix130-T3)-15min.qmethod

Regression Equation: $y = 0.00451\ x + 2.28470e-4$ ($r = 0.99979$, $r^2 = 0.99957$) (weighting: 1 / x)

Expected Concentration	Number of Values	Mean Calculated Concentration (No data for Analyte Unit)	% Accuracy	Std. Deviation	%CV
0.01	0 of 1	N/A	N/A	N/A	N/A
0.02	0 of 1	N/A	N/A	N/A	N/A
0.05	0 of 1	N/A	N/A	N/A	N/A
0.13	0 of 1	N/A	N/A	N/A	N/A
0.33	0 of 1	N/A	N/A	N/A	N/A
0.82	0 of 1	N/A	N/A	N/A	N/A
2.05	1 of 1	1.932e0	94.3	N/A	N/A
5.12	1 of 1	5.145e0	100.5	N/A	N/A
12.80	1 of 1	1.286e1	100.5	N/A	N/A
32.00	1 of 1	3.378e1	105.6	N/A	N/A
80.00	1 of 1	8.006e1	100.1	N/A	N/A
200.00	1 of 1	1.982e2	99.1	N/A	N/A

Analyte Name: LM-flavones-127_1

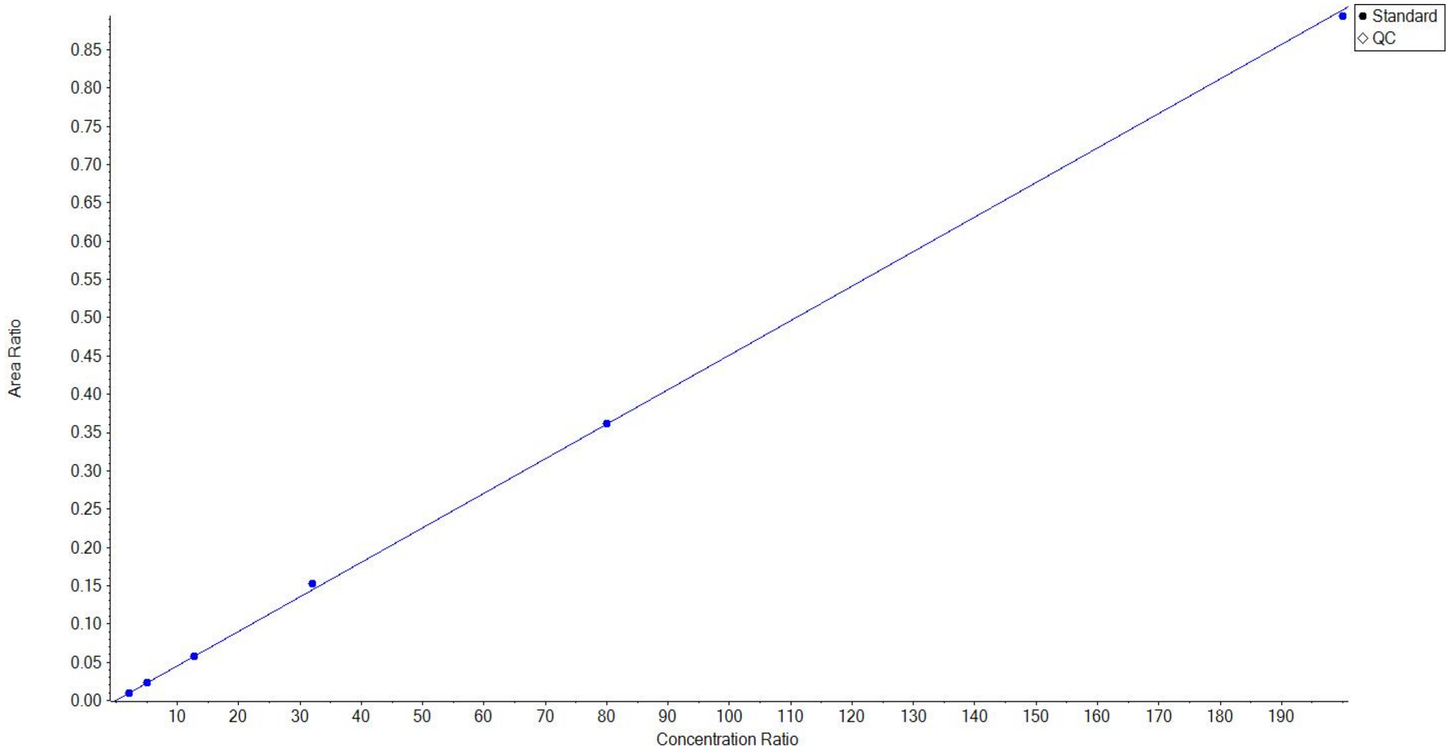
Standard
QC
Area Ratio
0.00
0.05
0.10
0.15
0.20
0.25
0.30
0.35
0.40
0.45
0.50
0.55
0.60
0.65
0.70
0.75
0.80
0.85
10
20
30
40
50
60
70
80
90
100
110
120
130
140
150
160
170
180
190
Concentration Ratio

Analyte Name: LM-flavones-129_1
Internal Standard: LM-flavones-IS02_1

**Data File**	flavones-STD-20230908.wiff	**Result Table**	DZLM2023082419-results-20230913-5500
**Acquisition Date**	9/8/2023 5:37:08 PM	**Algorithm Used**	MQ4
**Acquisition Method**	20230908-flavones-(mix130-T3)-15min.dam	**Instrument Name**	QTRAP 6500+ Low Mass
**Project**	N/A	**Processing Method**	20230412-flavones-(mix130-T3)-15min.qmethod

Regression Equation: $y = 0.00228\ x + 0.00134\ (r = 0.99762,\ r^2 = 0.99525)$ (weighting: 1 / x)

Expected Concentration	Number of Values	Mean Calculated Concentration (***No data for Analyte Unit***)	% Accuracy	Std. Deviation	%CV
0.01	0 of 1	N/A	N/A	N/A	N/A
0.02	0 of 1	N/A	N/A	N/A	N/A
0.05	0 of 1	N/A	N/A	N/A	N/A
0.13	0 of 1	N/A	N/A	N/A	N/A
0.33	0 of 1	N/A	N/A	N/A	N/A
0.82	0 of 1	N/A	N/A	N/A	N/A
2.05	1 of 1	1.646e0	80.4	N/A	N/A
5.12	1 of 1	4.968e0	97.0	N/A	N/A
12.80	1 of 1	1.465e1	114.5	N/A	N/A
32.00	1 of 1	3.676e1	114.9	N/A	N/A
80.00	1 of 1	7.505e1	93.8	N/A	N/A
200.00	1 of 1	1.989e2	99.5	N/A	N/A

Analyte Name: LM-flavones-129_1

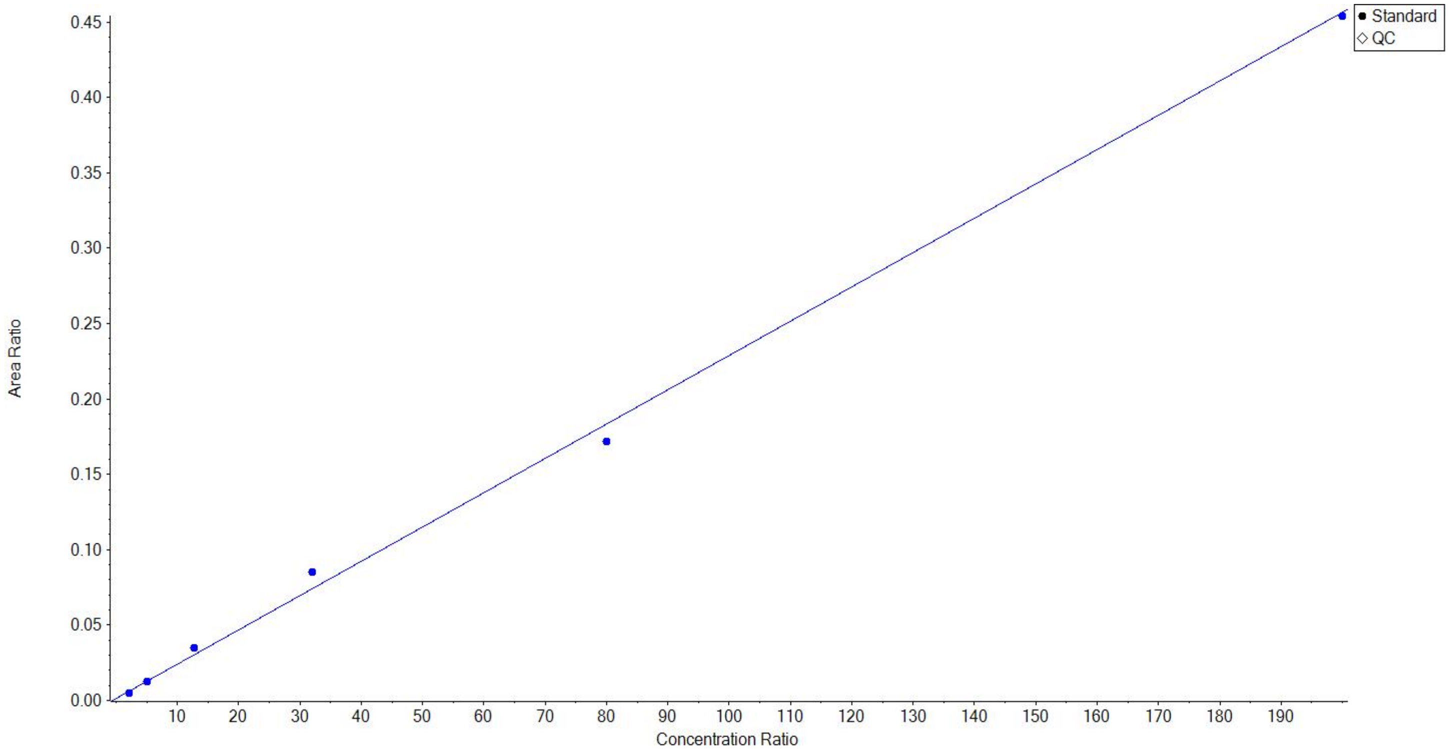
Standard
QC
Area Ratio
Concentration Ratio
0.00
0.05
0.10
0.15
0.20
0.25
0.30
0.35
0.40
0.45
10
20
30
40
50
60
70
80
90
100
110
120
130
140
150
160
170
180
190
